# Allelic Switching of *DLX5*, *GRB10*, and *SVOPL* during Colorectal Cancer Tumorigenesis

**DOI:** 10.1155/2019/1287671

**Published:** 2019-04-10

**Authors:** Arnoud Boot, Jan Oosting, Saskia Doorn, Sarah Ouahoud, Marina Ventayol Garcia, Dina Ruano, Hans Morreau, Tom van Wezel

**Affiliations:** Department of Pathology, Leiden University Medical Center, Leiden, Netherlands

## Abstract

Allele-specific expression (ASE) is found in approximately 20-30% of human genes. During tumorigenesis, ASE changes due to somatic alterations that change the regulatory landscape. In colorectal cancer (CRC), many chromosomes show frequent gains or losses while homozygosity of chromosome 7 is rare. We hypothesized that genes essential to survival show allele-specific expression (ASE) on both alleles of chromosome 7. Using a panel of 21 recently established low-passage CRC cell lines, we performed ASE analysis by hybridizing DNA and cDNA to Infinium HumanExome-12 v1 BeadChips containing cSNPs in 392 chromosome 7 genes. The results of this initial analysis were extended and validated in a set of 89 paired normal mucosa and CRC samples. We found that 14% of genes showed ASE in one or more cell lines and identified *allelic switching* of the potential cell survival genes *DLX5*, *GRB10*, and *SVOPL* on chromosome 7, whereby the most abundantly expressed allele in the normal tissue is the lowest expressed allele in the tumor and vice versa. We established that this allelic switch does not result from loss of imprinting. The allelic switching of *SVOPL* may be a result of transcriptional downregulation, while the exact mechanisms resulting in the allelic switching of *DLX5* and *GRB10* remain to be elucidated. In conclusion, our results show that profound changes take place in allelic transcriptional regulation during the tumorigenesis of CRC.

## 1. Introduction

**Figure 1:**
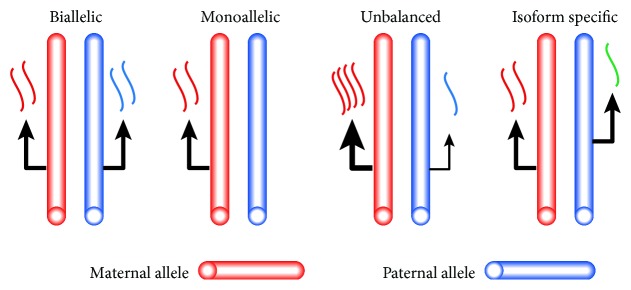
Subtypes of allele-specific expression patterns.

Approximately 20-30% of human genes show differential expression of parental alleles, commonly referred to as allele-specific expression (ASE) [1, 2]. The various subtypes of the ASE that can be distinguished include monoallelic expression, unbalanced expression, and isoform-specific ASE (Figure 1). Monoallelic expression is often studied in relation to X-chromosome inactivation or genomic imprinting [3–5]. Genes with an unbalanced expression have one allele that is more transcriptionally active than the other, a difference that may be caused by the presence of single nucleotide polymorphisms (SNPs) in regulatory elements. These are commonly referred to as expression quantitative trait loci (eQTLs) [6–8]. Isoform-specific ASE is defined as the monoallelic or unbalanced expression of one of the isoforms of a gene. ASE, and eQTLs in particular, has been proposed as a major factor in the predisposition to complex disease [9]. The contribution of eQTLs and ASE to tumor development and progression has been the subject of previous studies [10, 11]. ASE of *APC* and *TGFBR1*, for example, is known to be associated with colorectal cancer (CRC), with the frequency of ASE in both *APC* and *TGFBR1* reportedly increased up to 10-fold in CRC compared to controls [12, 13].

Recent studies have demonstrated that paired normal and tumor tissues show differences in ASE, indicating that ASE changes during tumorigenesis [14, 15]. In CRC, much of this difference has been attributed to copy number alterations or somatic alterations that change the regulatory landscape [14]. In a meta-analysis of CRC, chromosomes 7, 8q, 13, and 20 most frequently showed copy number gains, while 8p, 17, and 18 were the most frequently deleted chromosomes [16]. Interestingly, chromosome 7 is preferentially retained in a heterozygous state in a wide range of tumor types [17–22]. Oncocytic follicular thyroid carcinomas, for example, show homozygosity of nearly all chromosomes, with the notable exceptions of chromosomes 7 and to a lesser extent of 5 and 12, at which heterozygosity is nearly always retained [20, 23, 24]. Retention of at least one maternal and one paternal copy of chromosome 7 is also observed in lung, kidney, and skin cancers [17, 19, 25]. The high frequency of retention of heterozygosity on chromosome 7 suggests that the presence of a set of tumor cell survival genes subject to ASE leads to selective pressure on these cells to retain heterozygosity.

We recently described a large collection of novel low-passage CRC cell lines [26]. Starting with these cell lines, we employed an array-based ASE detection method using Infinium HumanExome-12 v1 arrays to identify ASE in CRC, in particular on chromosome 7. ASE was found in 14% of genes across all chromosomes in one or more informative samples. Validation was performed in a collection of 89 paired CRC and adjacent normal mucosa samples, producing similar results to those found in the cell lines. Interestingly, comparison of CRC and normal mucosa samples revealed the allelic switching of *DLX5*, *GRB10*, and *SVOPL* during tumorigenesis, possibly as a result of DNA conformational changes.

## 2. Results

### 2.1. ASE Discovery Using Coding SNP Arrays

We have previously shown that ASE of cell survival genes plays an essential role in the retention of heterozygosity on chromosome 7 in thyroid cancer [27]. Similarly, in CRC, heterozygosity of chromosome 7 is almost always retained. We therefore investigated the ASE in CRC using cSNP arrays in this study.

**Figure 2:**
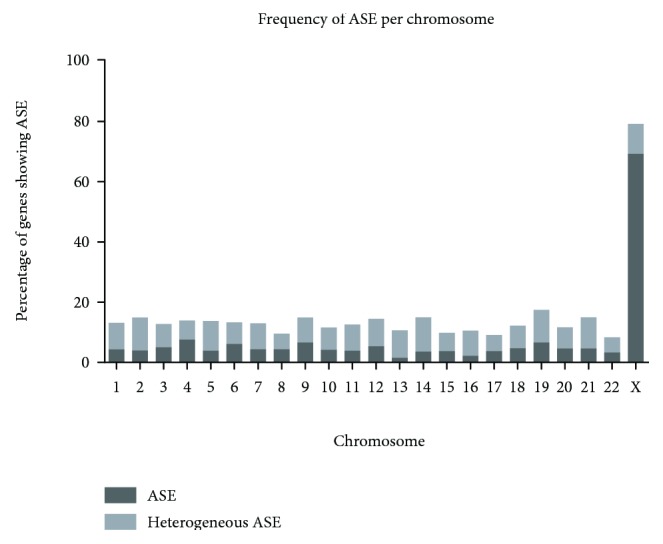
Percentage of genes displaying ASE per chromosome.

A total of 7,638 genes were suitable for ASE analysis due to the presence of at least one coding SNP (cSNP) in one or more of the colorectal cancer cell lines; 392 of these genes originate from chromosome 7. The ASE results for all genes, per cell line, are listed in Supplementary Table 1. For each gene, we classified the expression as either ASE, heterogeneous ASE (>25% of eligible samples showing ASE), or no ASE. Overall, 14.0% of genes showed either ASE or heterogeneous ASE. The frequency distribution of the ASE across the autosomes ranged from 1.5% to 7.5% (Figure 2), whereas 69.2% of genes on the X-chromosome displayed ASE (as a result of X-chromosome inactivation). The X-chromosomal genes that lacked ASE or showed heterogeneous expression either were located in the pseudoautosomal regions or had been previously reported to escape imprinting [28–30]. Both the high degree of ASE found on the X-chromosome and the concordance with reports of genes escaping X-inactivation indicated that coding SNP arrays can efficiently detect ASE.

Of all the genes on chromosome 7 for which probes are present on the array, 45 genes show either ASE or heterogeneous ASE and 347 genes do not show ASE (Supplemental Table 1). The strongest patterns of the ASE on chromosome 7 we found were for the *PRPS1L1* gene, with strong ASE in all samples heterozygous for the SNP rs3800962 (9/21 samples).

Fifteen genes located on chromosome 7 are known to be imprinted: *COPG2IT1*, *DLX5*, *GRB10*, *KLF14*, *MAGI2*, *MEST*, *MESTIT1*, *SGCE*, *TFPI2*, *CALCR*, *COPG2*, *CPA4*, *DDC*, *PEG10*, and *PPP1R9A* [31, 32]. Informative cSNPs located in the latter 6 genes were present on the arrays. Analysis of these cSNPs revealed a heterogeneous pattern of ASE for *CALCR* and *PEG10*, whereas *CPA4*, *COPG2*, *DDC*, and *PPP1R9A* lacked ASE (Supplementary Figure 1). As other imprinted genes were not covered by informative cSNPs on our arrays, we studied additional genes on chromosome 7. Recently, *GLI3* and *SVOPL* were reported to show ASE [33]. We detected ASE of *SVOPL* in 4 cell lines but could find no evidence for ASE of *GLI3*.

We then selected *CPED1*, *PEG10*, *SVOPL*, *DLX5*, *GRB10*, and *MEST* for further study in a collection of CRC and matching adjacent normal mucosa samples. *CPED1* was selected for validation as an example of heterogeneous ASE. *DLX5* is located in the chr7p21.3 imprinted gene cluster and is reportedly hypermethylated in CRC [34]. *GRB10* and *MEST* are known to undergo isoform-dependent imprinting. However, because the arrays were not designed to detect differential sharing of exons between different isoforms, this phenomenon is virtually impossible to detect using exon arrays.

*PEG10* showed monoallelic expression in all but one of the normal mucosa samples and in most CRC samples (Table 1). *CPED1* showed heterogeneous ASE in both normal mucosa and cancer samples. Five of the seven samples showing the ASE of *CPED1* in the normal mucosa also retained the ASE of *CPED1* in the paired tumor sample. In addition, seven samples showed the ASE of *CPED1* in the tumor sample but not in the paired normal mucosa, indicating that these cancers acquired ASE of *CPED1* during tumorigenesis.

### 2.2. Allelic Switching

**Figure 3:**
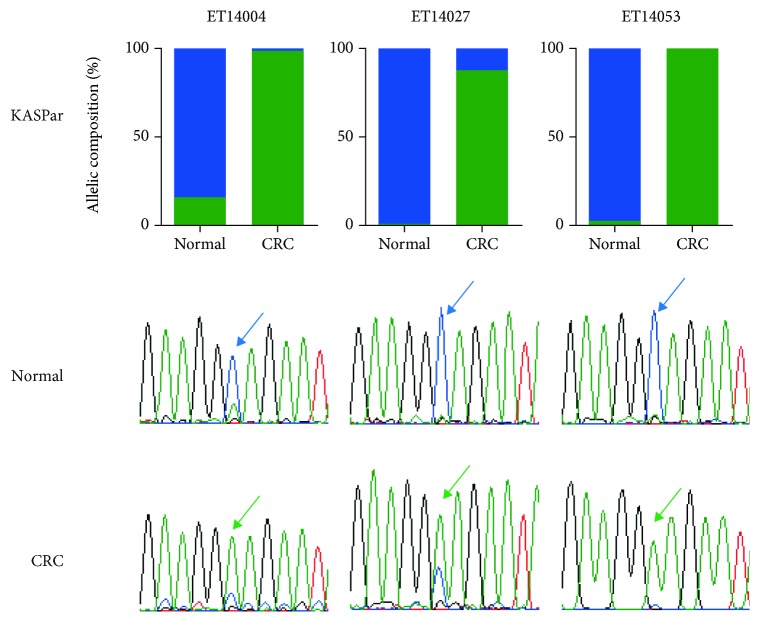
Allelic quantification of three samples displaying allelic switching of *SVOPL*. The allelic composition of *SVOPL* cDNA for both normal and colorectal cancer (CRC) cDNA was tested by KASPar analysis (a) (blue: C allele; green: A allele). Validation of allelic quantification using Sanger sequencing confirmed the C allele to be most abundant in the normal cDNA samples, while the A allele was most abundant in the CRC samples. The investigated cSNP, rs2305816, is indicated with arrows (b).

ASE analysis of *SVOPL* in paired normal and tumor CRC samples revealed 5 samples with expression of the opposite allele in the tumor as compared to the normal mucosa. This suggests *allelic switching* for this gene. To exclude the alternative possibility that sample swaps could have caused this pattern, we genotyped 7 cSNPs on both the DNA and the cDNA of each normal and CRC sample. All cSNPs showed identical genotypes for the paired DNA and cDNA, confirming sample identity. Allelic switching of *SVOPL* was further verified by Sanger sequencing (Figure 3). Most samples showed strong downregulation of *SVOPL*, with a median 8-fold lower expression in the tumor compared to the normal sample, irrespective of allelic switching or any other change in the ASE status (Supplementary Figure 2). We therefore concluded that in samples that display allelic switching, the most abundantly expressed allele is downregulated, possibly in combination with a slight upregulation of the other allele.

Allelic switching was also observed for *DLX5*. Three out of 31 informative samples were found to express different alleles in the cancer sample compared to normal mucosa. Additionally, 17 samples either acquired or lost the ASE during tumorigenesis, indicative of a high degree of variability in the regulation of *DLX5* (Supplementary Table 2). *DLX5* expression was upregulated in most CRCs compared to normal mucosa samples. Allelic switching and changes in the ASE status between normal and CRC samples were not found to affect gene expression ratios (Supplementary Figure 2) suggesting that one of the *DLX5* alleles is upregulated during tumorigenesis. In the samples displaying allelic switching, the upregulated allele is underrepresented in the normal mucosa, possibly in combination with downregulation of the opposite allele.

Changing patterns of ASE in normal and tumor tissues suggests that ASE of *DLX5* and *SVOPL* is not parent-of-origin-specific. Moreover, regulation of the expression of these genes is frequently altered during tumorigenesis, but this is not a result of or influenced by changes in ASE.

**Figure 4:**
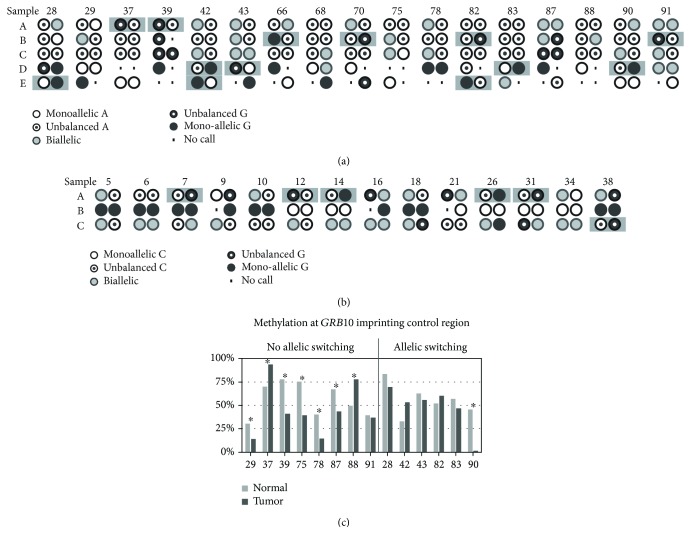
ASE results for *GRB10* and *MEST*. Isoform-specific ASE results for *GRB10* (a) and *MEST* (b). ASE calls for paired samples are displayed with the normal sample to the left and the CRC sample to the right. Assays displaying allelic switching are marked with a grey background. (c) Methylation status of the *GRB10* imprinting control region in samples with and without allelic switching. Asterisks mark samples with significant methylation difference between normal and tumor DNA.

In oncocytic thyroid carcinomas, ASE analysis of *GRB10* and *MEST* showed a high degree of variability of the distribution of isoforms expressed per allele [27]. To investigate whether allelic switching could explain this variability, we analyzed these genes using an isoform-specific nested PCR approach. Allelic distributions of the 5 different *GRB10* isoforms (Figure 4(a)) and the 3 different *MEST* isoforms (Figure 4(b)) in paired normal and tumor samples were compared. The allelic distribution of the different isoforms was highly variable between samples. Nonetheless, at least one of the isoforms was either monoallelically or predominantly monoallelically expressed in all normal and cancer samples. In the case of *GRB10*, 80.5% of the samples showed ASE, with the highest frequency of ASE detected with the *GRB10*-E assay (94.7%).

Interestingly, 11 of 17 samples displayed allelic switching of *GRB10* (Figure 4(a)). Notably, no allelic switching was observed for the *GRB10*-C assay. None of the paired samples showed the same ASE pattern in both the normal and tumor samples, highlighting the high degree of variability in the ASE patterns of *GRB10*.

Allelic switching of *MEST* was found in 6 of 14 samples but not for the *MEST*-B assay, which was exclusively monoallelically expressed (Figure 4(b)), in line with known parent-of-origin-dependent expression.

### 2.3. Imprinting Status of *GRB10*

Loss of imprinting has been shown to underlie the allelic switching of imprinted genes in renal cell and hepatocellular carcinoma [35, 36]. To investigate whether the allelic switching of *GRB10* in CRC is the result of loss of imprinting, we determined the methylation status of the *GRB10* imprinting control region on chr7p12.2. We found both hypomethylation and hypermethylation in CRC compared to paired normal mucosa (Figure 4(c)). Of the samples with the allelic switching of *GRB10*, only one showed a significant difference in the DNA methylation level, whereas all but one sample without allelic switching showed significant deregulation of the imprinting control region. This result indicates that there is no co-occurrence of deregulation of the chr7p12.2 imprinting control region and allelic switching of *GRB10*. We therefore conclude that while deregulation of the imprinting control region of *GRB10* is a frequent event in CRC, deregulation is unrelated to the allelic switching of this gene.

## 3. Discussion

This investigation of allele-specific expression (ASE) and the degree of deregulation of ASE during CRC tumorigenesis revealed extensive allelic switching, a pattern of transcriptional deregulation whereby paired normal and cancer tissues display the ASE but of opposite alleles. Allelic switching was observed for *DLX5*, *SVOPL*, and *GRB10*.

ASE has been suggested as a major contributor to a predisposition for and development of complex diseases such as CRC [10, 14, 37–39]. Both the regulation of gene expression and ASE patterns in different cell types show high variability [40, 41]. To understand the contribution of ASE to tumorigenesis, the ASE patterns of both the tumor cells and the originating cell type should be evaluated. Ideally, transcriptome sequencing should be performed, in combination with whole-exome or whole-genome sequencing, and several studies based on transcriptome sequencing have indeed reported the ASE [15, 42, 43]. However, transcriptome sequencing is still a very costly approach and analysis remains bioinformatically challenging [44]. We therefore opted for a coding SNP array approach, a proven technique for ASE analysis [45]. Although using coding SNP arrays for detection of ASE has limitations in terms of the number of genes and variants that can be detected, the technique does allow larger sample sizes to be analyzed at lower costs.

Our analysis identified the strongest patterns of ASE on chromosome 7 in the *PRPS1L1* gene, with a strong ASE in all samples heterozygous for the SNP rs3800962 (Supplementary Figure 1). Strikingly, all samples showed expression of the G allele, even 2 samples with DNA homozygous for the T allele. Using KASPar genotyping on the DNA and cDNA of the CRC cell lines, we found the obtained genotypes to be consistent with the microarray results. Also, in the cohort of 89 matched normal and CRC samples, all samples that are homozygous for the T allele also showed a strong expression of the G allele in the cDNA (Supplementary Table 3). This rather counterintuitive finding suggests that *PRPS1L1* is subject to RNA editing, a regulatory mechanism through which genes can be activated or inactivated [45]. RNA editing can both obscure ASE and create a false impression of ASE. This finding underlines the care that should be taken when performing ASE analysis, including ensuring that homozygous samples of both alleles are available and confirming a matching genotype in cDNA before drawing conclusions.

ASE of *GRB10* and *MEST* was recently shown to vary considerably between different tissue types, and ASE was reported in a small number of tissues [41]. Due to the high similarity of the various isoforms of both genes, the detection of ASE of single isoforms using transcription profiling is restricted to cSNPs in the exons and UTRs that are unique to a subset of isoforms. Assays based on cSNPs shared by all isoforms will tend to obscure patterns of ASE and create an appearance of biallelic expression. We therefore adopted a nested allele-specific amplification method to detect ASE of the various isoforms. Using this method, we were able to detect ASE of multiple *GRB10* and *MEST* isoforms at frequencies much higher than previously reported [41].

Our results indicate that extensive regulatory changes of genes displaying ASE occur during tumorigenesis. ASE of *DLX5*, *GRB10*, and *SVOPL* has been reported previously, but no published study has investigated the stability of ASE of these genes during tumorigenesis. While the regulatory mechanisms underlying extreme allelic switching during tumorigenesis remain elusive, our gene expression results suggest that the allelic switching of *SVOPL* could be the result of transcriptional downregulation during tumorigenesis, with downregulation of the most abundantly expressed allele and an unaffected or slightly upregulated second allele. One possible explanation for the silencing of the highly expressed allele would be a mutation in this allele resulting in nonsense-mediated decay. However, this scenario suggests frequent mutations of these genes, but there is currently no evidence for this in the CRC data that can be found in the Cancer Genome Atlas [46, 47]. Rather than up- or downregulation of one allele, an alternative explanation might be chromosomal conformational change such as chromosome looping, which could theoretically result in extreme allelic switching of transcriptional activity.

This study represents the first report of allelic switching during CRC tumorigenesis, although allelic switching has been reported in other tumor types. For example, *TP73* displayed loss of imprinting in 8 of 12 renal cell carcinomas, and two of these samples also displayed allelic switching [36]. Similarly, a study of the imprinting status of the *DLK1-MEG3* locus in hepatocellular carcinoma found expression of the imprinted allele in 20% of cases [35].

When we analyzed the methylation status of the imprinting control region of the imprinted gene *GRB10*, we found that allelic switching does not result from loss of imprinting. *DLX5*, regulated by the chr7q21.3 imprinting cluster, has also been reported to be imprinted [48]. As monoallelic or predominantly monoallelic expression of *DLX5* in the normal colon mucosa was observed in only 17 out of 30 cases, we concluded that there is a lack of evidence for imprinting of *DLX5* in the colonic epithelium, a finding in agreement with other studies that were also unable to confirm imprinting of *DLX5* in the colonic epithelium [41, 49].

*DLX5*, *GRB10*, and *SVOPL* have distinct functions. *DLX5* is a member of the DLX family of homeodomain transcription factors, which share sequence similarities with the Drosophila distal-less gene. *DLX5* has been shown to stimulate tumor cell proliferation through upregulation of the MYC expression [50, 51]. *GRB10* encodes a growth receptor bound protein that plays an important role in multiple key cancer signalling pathways such as the Wnt and Akt pathways [52, 53]. Consequently, both *DLX5* and *GRB10* are putative oncogenes, and allelic switching could serve as a mechanism to compensate for transcriptional silencing of the expressed allele or as a switch to the alternative allele when the expressing allele is mutated.

*SVOPL* (SVOP-like protein) is a paralog of the synaptic vesicle protein SVOP. SVOPL is a putative transporter, although no substrates have been identified to date [54]. The potential role of *SVOPL* in tumorigenesis is presently unclear. Akin to *SVOPL*, the lung cancer proapoptotic factor *BCL2L10* has been shown to display switching of the most abundantly expressed allele during tumorigenesis [15]. As the transcriptional downregulation of *SVOPL* was observed in all CRC samples, *SVOPL* allelic switching may occur primarily as a result of downregulation of the most abundantly expressed allele, unlike *DLX5* and *GRB10*. We therefore conclude that allelic switching of these genes is due to changes in transcriptional regulation that affect the individual alleles differently.

ASE analysis remains challenging due to the technical and analytical caveats mentioned above. Adding to the complexity of ASE analysis, we have shown that allelic switching can occur during tumorigenesis, indicative of the complexity of transcriptional deregulation occurring during tumorigenesis. These results underline the importance of further rigorous investigation of patterns of ASE, both in a variety of different normal tissues and during tumorigenesis.

## 4. Materials and Methods

### 4.1. Cohorts

This study used both a discovery and a validation cohort. Discovery was performed using exon SNP arrays in 21 CRC cell lines [26]. For 3 cell lines, normal DNA was isolated from fibroblasts derived from the same patient.

ASE validation was performed in a collection of 178 anonymized snap-frozen tissue samples. These samples were obtained from the biobank of the Pathology Department at Leiden University Medical Center (Leiden, the Netherlands). Surgical specimens were collected between 2005 and 2009. Samples were selected based on the availability of paired carcinoma and normal fresh frozen tissue.

A list of all samples used in the study can be found in Supplementary Table 4. Male and female samples were equally represented in both cell lines and the primary tissue collection, with 48% of the cell lines and 53% of the tissue samples of male origin.

The present study was approved by the Medical Ethics committee of the Leiden University Medical Center (protocol P01-019) and analyzed according to the Code for Adequate Secondary Use of Data and Tissue, provided by the Federation of Dutch Medical Scientific Societies (www.federa.org).

### 4.2. Nucleic Acid Isolation

DNA from the cell lines and fibroblasts was isolated from cells at 70-80% confluency, using the Wizard Genomic DNA Purification Kit (Promega, Madison, WI, USA). RNA was isolated from cells in the exponential growth phase, using the TRIzol® Reagent (Life Technologies). DNAse treatment was performed in suspension using rDNAse (MACHEREY-NAGEL GmbH & Co. KG, Düren, Germany). cDNA was synthesized as previously described [55].

Histological examination of the validation samples was performed prior to nucleic acid isolation. Normal samples were selected on the basis of at least 70% colon epithelium, without any neoplastic cells present. Carcinoma samples were selected on the basis of at least 70% neoplastic cells. Following sectioning prior to isolation, histological examination was repeated to check the same parameters. DNA and RNA were isolated using the NucleoSpin® Tissue and NucleoSpin® RNA kits (MACHEREY-NAGEL GmbH & Co. KG, Germany), respectively.

### 4.3. SNP Array Analysis

Genotyping and gene expression analysis were performed using Infinium HumanExome-12 v1 BeadChips (Illumina, Eindhoven, Netherlands). 500 ng RNA was converted to cDNA using the DyNAmo™ cDNA Synthesis Kit (Thermo Fisher Scientific, Waltham, MA, USA), and cDNA was purified using the QIAquick PCR Purification Kit (QIAGEN, Germantown, Maryland, USA). cDNA was eluted in 15 *μ*L MQ water and 5 *μ*L was used as input. In the case of DNA, 200 ng was used as the input.

HumanExome BeadChips were processed according to the manufacturer's instructions (Illumina, Eindhoven, Netherlands), and arrays were scanned using the iScan Microarray Scanner.

### 4.4. ASE Detection Method and Array Analysis QC

Detection of ASE was performed by hybridizing both DNA and cDNA to the Infinium HumanExome-12 v1 BeadChips. For JVE017, JVE044, and JVE367, normal DNA was also assayed. Raw IDAT files were imported into the Illumina GenomeStudio V2011.1 software, from which raw data was exported for further analysis. Paired cDNA and gDNA samples clustered together, as presented in Figure S3.

### 4.5. Intensity Threshold for ASE Determination

To define a minimal-intensity cut-off for reliable genotyping on the cDNA, we examined the effect of signal intensity on the genotypes. Density distribution of the cDNA samples revealed a high peak with a low intensity, which contained intergenic probes and probes located within genes that are not expressed (Figure S4A). Genotypes of the low-intensity probes showed skewing of the *β*-allele frequencies to 0.5, as a result of the signal background (Figure S4B). Also, in the cDNA, the total signal intensity showed influence on *β*-allele frequencies (Figure S4C). Based on this data, a minimum intensity cut-off of 2000 was implemented for reliable ASE detection.

### 4.6. ASE Detection Strategy

cSNPs with *β*-allele frequencies at the DNA level (DNA-BAF) between 0.2 and 0.8 were considered candidate cSNPs for ASE detection (Figure S5A). To minimize the effect of background signal inherent to microarray data, cSNPs with a total intensity below 2000 were excluded from the analysis (Figure S5B). For the remaining cSNPs, ASE detection was performed, by comparing the *β*-allele frequency between cDNA (cDNA-BAF) and DNA. The calculation method used for *β*-allele frequency-shift analysis was adapted from the LAIR-analysis method (Figure S5C) [56, 57]. cSNPs with an allelic contribution ratio in the cDNA lower than 0.5 were considered to show ASE, corresponding to an allelic contribution ratio of 1 : 2. cSNPs with an allelic contribution ratio higher than 0.5 were classified as biallelic (Figure S5D).

### 4.7. Consistency of ASE Calling in Genes with Multiple Candidate cSNPs

For 7.3% of genes with multiple candidate cSNPs, inconsistent ASE calls were found between candidate cSNPs. To further examine this, we examined the ASE results for MUC16. Eight cell lines had least 4 candidate cSNPs; Figure S6A shows the ASE score per candidate probe. Based on these results, we concluded that JVE192 and KP7038T are the only cell lines showing the ASE of MUC16. This was confirmed when plotting the *β*-allele frequencies of the DNA and cDNA (Figure S6B). We therefore chose to use the average ASE score for all candidate cSNPs per gene to determine the ASE.

### 4.8. Gene-Level ASE Classification

Gene-level ASE classification was performed based on the percentage of samples showing the ASE. Genes with 75% or more samples displaying either the ASE or no ASE were classified accordingly. The remaining genes were classified as displaying heterogeneous ASE.

### 4.9. KASPar Genotyping

SNP genotyping was performed using the competitive allele-specific PCR (KASPar) assay, following the manufacturer's protocol (LGC Genomics, Berlin, Germany). Primers were designed using Primerpicker (KBioscience, Hoddesdon, UK).

### 4.10. KASPar ASE Analysis

cDNA synthesis and qRT-PCR were performed as described previously. For ASE analysis, 2 *μ*L of 25x diluted cDNA was used as a template for the KASPar assay. Using the Cq values obtained for both alleles, the allelic dosage was calculated in a manner similar to the Pfaffl method for relative gene expression [58]. The allelic dosage observed in the cDNA was then adjusted for the amplification difference between the two alleles in the DNA sample, in order to compensate for genomic imbalance, if present.

Allelic expression ratios of 3 : 1 and 2 : 1 were considered to be the predominantly monoallelic expression or unbalanced expression, respectively [59]. Allelic contribution ratios exceeding 9 : 1 were classified as monoallelic (with some residual expression of the underexpressed allele).

### 4.11. Isoform-Specific ASE of *GRB10* and *MEST*

A nested KASPar approach was used for the isoform-specific ASE detection of *GRB10* and *MEST*. In short, multiple isoform-specific PCRs were designed for each gene. Specific PCRs were used to amplify cDNA from one of the unique exons of the different isoforms and included a high-frequency cSNP in one of the exons shared by all isoforms (rs1800504 for *GRB10* and rs1050582 for *MEST*) [27].

## Figures and Tables

**Table 1:** ASE results in normal colon mucosa and colorectal cancer samples. Numbers represent the amount of normal mucosa (N) and colorectal cancer (CRC) samples showing ASE and the total number of samples eligible for ASE detection heterozygous samples (hetSMP).

			Monoallelic		Predominantly monoallelic		Unbalanced		Biallelic	
Gene	cSNP	hetSMP	N	CRC	N	CRC	N	CRC	N	CRC
*CPED1*	rs1524498	26	—	1	2	7	6	4	17	14
*DLX5*	c.1241InsG	31	11	9	6	7	5	5	9	10
*PEG10*	rs3750105	21	20	17	—	1	—	—	1	3
*SVOPL*	rs2305816	24	16	21	3	—	3	1	2	2

## Data Availability

The ASE results used to support the findings of this study are included within the Supplementary Tables 1 and 2.

## Supplementary Materials

Supplementary Materials Supplementary Figure 1: ASE scores for selected chromosome 7 genes as determined by cSNP arrays. ASE scores were only calculated for cell lines with a heterozygous cSNP in the DNA. Supplementary Figure 2: gene expression of *SVOPL* and *DLX5.* Supplementary Table 1: ASE results per cell line for all genes with at least 1 heterozygous sample. Per sample, each gene was assigned an ASE score, based on the data from the heterozygous cSNPs, as described in the Supplementary methods. Samples displaying the ASE for that gene are marked 1, and samples not showing the ASE are marked 0. For samples where no heterozygous cSNPs were identified, the field was left empty. Genes for which none of the samples showed a heterozygous cSNP were removed, as the ASE could not be calculated. Supplementary Table 2: *DLX5* ASE results in paired normal and cancer samples. ASE scores for paired normal mucosa and CRC samples. The 4th column states the case-level conclusion concerning allelic switching during tumorigenesis. Supplementary Table 3: PRPS1L1 KASPar genotyping results in paired normal and cancer samples. Supplementary Table 4: samples used in this study. Supplementary methods: ASE detection method and array analysis QC.
