# Supplementary material for: Allelic Switching of DLX5, GRB10, and SVOPL during Colorectal Cancer Tumorigenesis
Source: Int J Genomics. 2019 Apr 10;2019:1287671. doi: 10.1155/2019/1287671 (PMC6481143; doi:10.1155/2019/1287671)
Supplement: Supplementary Materials — Supplementary Figure 1: ASE scores for selected chromosome 7 genes as determined by cSNP arrays. ASE scores were only calculated for cell lines with a heterozygous cSNP in the DNA. Supplementary Figure 2: gene expression of SVOPL and DLX5. Supplementary Table 1: ASE results per cell line for all genes with at least 1 heterozygous sample. Per sample, each gene was assigned an ASE score, based on the data from the heterozygous cSNPs, as described in the Supplementary methods. Samples displaying the ASE for that gene are marked 1, and samples not showing the ASE are marked 0. For samples where no heterozygous cSNPs were identified, the field was left empty. Genes for which none of the samples showed a heterozygous cSNP were removed, as the ASE could not be calculated. Supplementary Table 2: DLX5 ASE results in paired normal and cancer samples. ASE scores for paired normal mucosa and CRC samples. The 4th column states the case-level conclusion concerning allelic switching during tumorigenesis. Supplementary Table 3: PRPS1L1 KASPar genotyping results in paired normal and cancer samples. Supplementary Table 4: samples used in this study. Supplementary methods: ASE detection method and array analysis QC. [file 1287671.f1.pdf]

Supplementary files:to "Allelic switching of *DLX5*, *GRB10* and *SVOPL* during colorectal cancer tumorigenesis"

Arnoud Boot, Jan Oosting, Saskia Doorn, Sarah Ouahoud, Marina Ventayol Garcia, Dina Ruano, Hans Morreau, Tom van Wezel*

Supplementary figure 1: ASE scores for selected chromosome 7 genes as determined by cSNP arrays. ASE scores were only calculated for cell lines with a heterozygous cSNP in the DNA.

Supplementary figure 2: Gene expression of *SVOPL* and *DLX5.*

Supplementary table 1: ASE results per cell line for all genes with at least 1 heterozygous sample

Per sample, each gene was assigned an ASE score, based on the data from the heterozygous cSNPs, as described in the Supplementary methods. Samples displaying ASE for that gene are marked 1, samples not showing ASE are marked 0. For samples where no heterozygous cSNPs were identified, the field was left empty. Genes for which none of the samples showed a heterozygous cSNP were removed, as ASE could not be calculated.

Supplementary table 2: *DLX5* ASE results in paired normal and cancer samples.

ASE scores for paired normal mucosa and colorectal cancer samples. The 4th column states the case-level conclusion concerning allelic switching during tumorigenesis.

Supplementary table 3: PRPS1L1 KASPar genotyping results in paired normal and cancer samples.

Supplementary table 4: Samples used in this study.

Supplementary methods: ASE detection method and array analysis QC

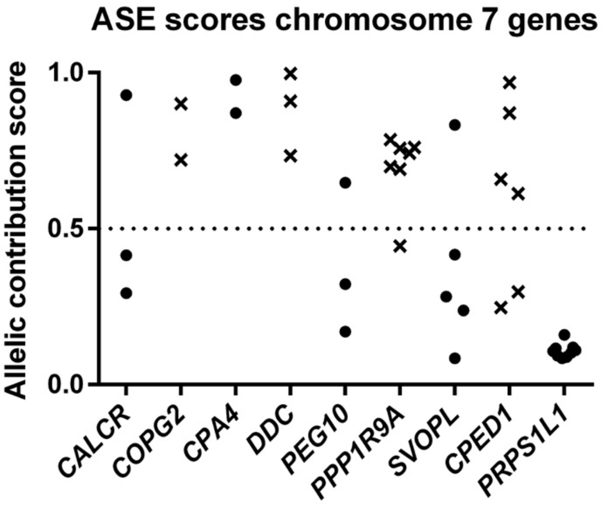

Figure S1: **ASE scores for selected chromosome 7 genes**

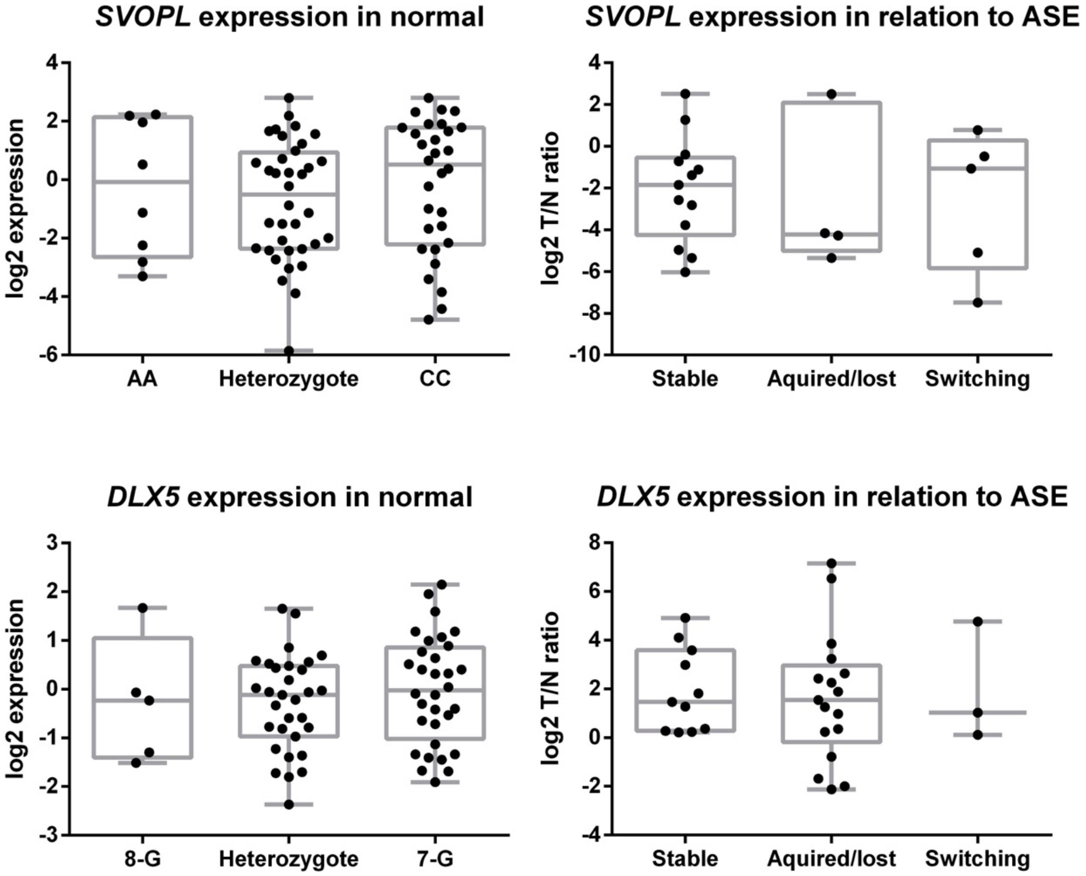

Figure S2: **Gene expression of *SVOPL* and *DLX5***

Supplementary methods: ASE detection method and array analysis QC

Detection of ASE was performed by hybridizing both DNA and cDNA to the Infinium HumanExome-12v1 beadchips. For JVE017, JVE044 and JVE367 also normal DNA was assayed. Raw IDAT files were imported into the Illumina GenomeStudio V2011.1 software, from which raw data was exported for further analysis. Paired samples showed to cluster together (Figure S3)

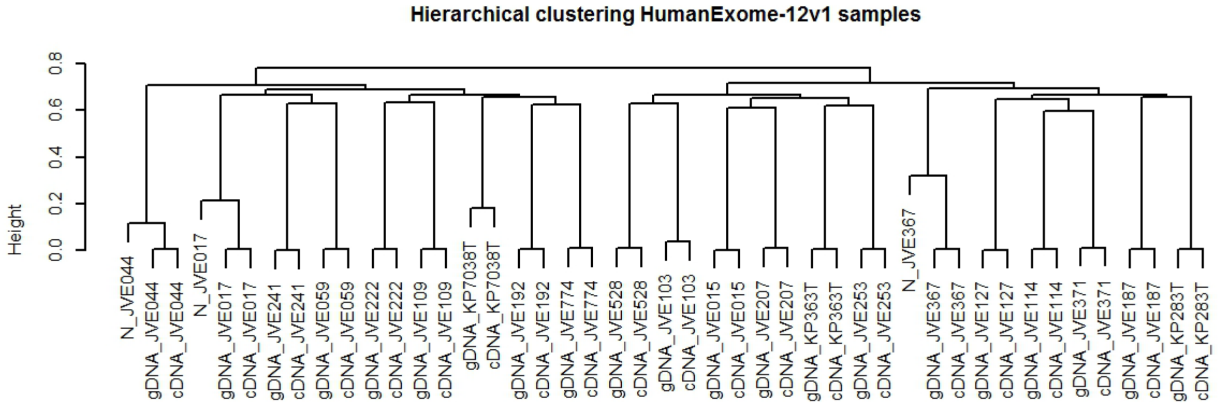

Figure S3: Hierarchical clustering of Infinium HumanExome12v1 beadchip genotypes on the 1000 most variable probes.

Intensity threshold for ASE determination

To define a minimal-intensity cut-off for reliable genotyping on the cDNA we examined the effect of signal intensity on the genotypes. Density distribution of the cDNA samples revealed a high peak with a low intensity, which contained intergenic probes and probes located within genes that are not expressed (Figure S4A). Genotypes of the low intensity probes showed skewing of the β-allele-frequencies to 0.5, as a result of signal background (Figure S4B). Also in the cDNA, total signal intensity showed to influence β-allele-frequencies (Figure S4C). Based on this data a minimum intensity cut-off of 2000 was implemented for reliable ASE detection.

ASE detection strategy

cSNPs with β-Allele-Frequencies at DNA level (DNA-BAF) between 0.2 and 0.8 were considered candidate cSNPs for ASE detection (**Figure S5A**). To minimize the effect of background signal inherent to micro-array data, cSNPs with a total intensity below 2000 were excluded from the analysis (**Figure S5B**). For the remaining cSNPs ASE detection was performed, by comparing the β-allele-frequency between cDNA (cDNA-BAF) and DNA. The calculation method used for β-allele-frequency-shift analysis was adapted from the LAIR-analysis method (**Figure S5C**) (Oosting et al., 2007; Corver et al., 2008). cSNPs with an allelic contribution ratio in the cDNA lower than 0.5 were considered to show ASE, corresponding to an allelic contribution ratio of 1:2. cSNPs with an allelic contribution ratio higher than 0.5 were classified as bi-allelic (**Figure S5D**).

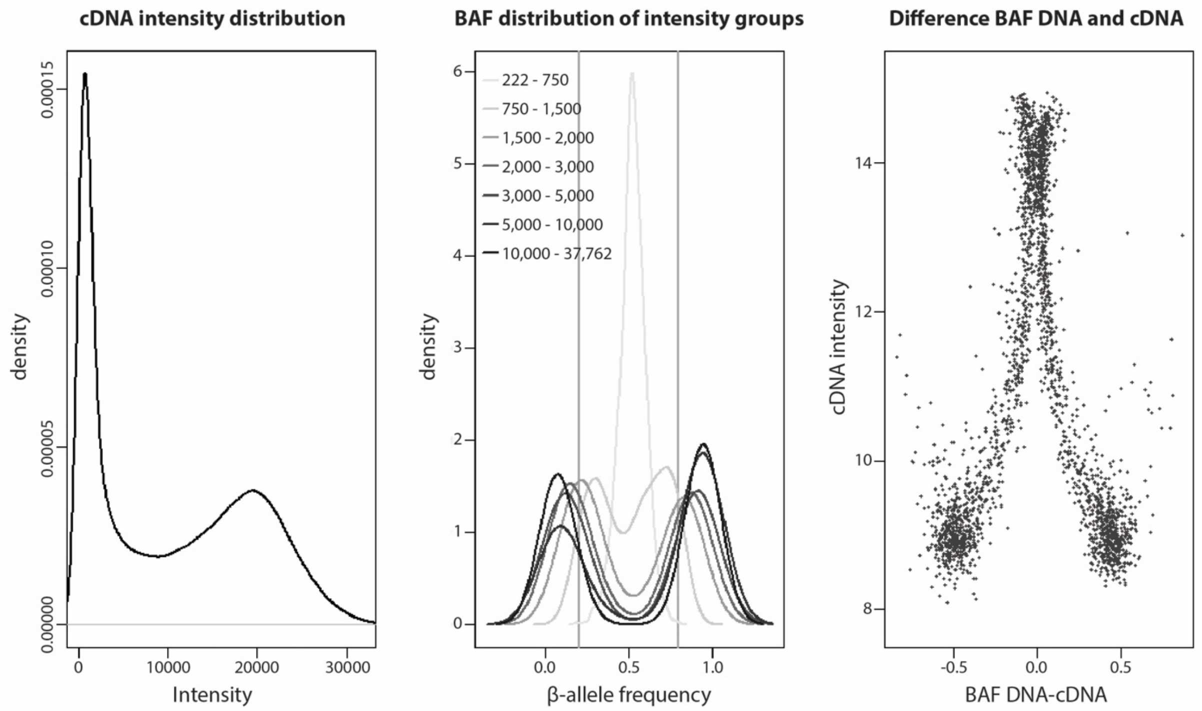

Figure S4: Intensity threshold determination for ASE detection. Approximately 100,000 SNPs in the cDNA samples showed low intensities (Figure S4A). The higher the intensity the more the β-allele-frequency distribution approaches the extremes. Low intensity SNPs show a β-allele-frequency distribution skewing towards 0.5, which is caused by the background signal of the array (Figure S4B). This is again seen when plotting the difference in β-allele-frequency between the DNA and cDNA for homozygous cSNPs. The higher the intensity, the less difference is observed between the DNA and cDNA β-allele-frequencies. A minimum intensity of 2,000 was chosen for reliable β-allele-frequency calculation in the cDNA (Figure S4C).

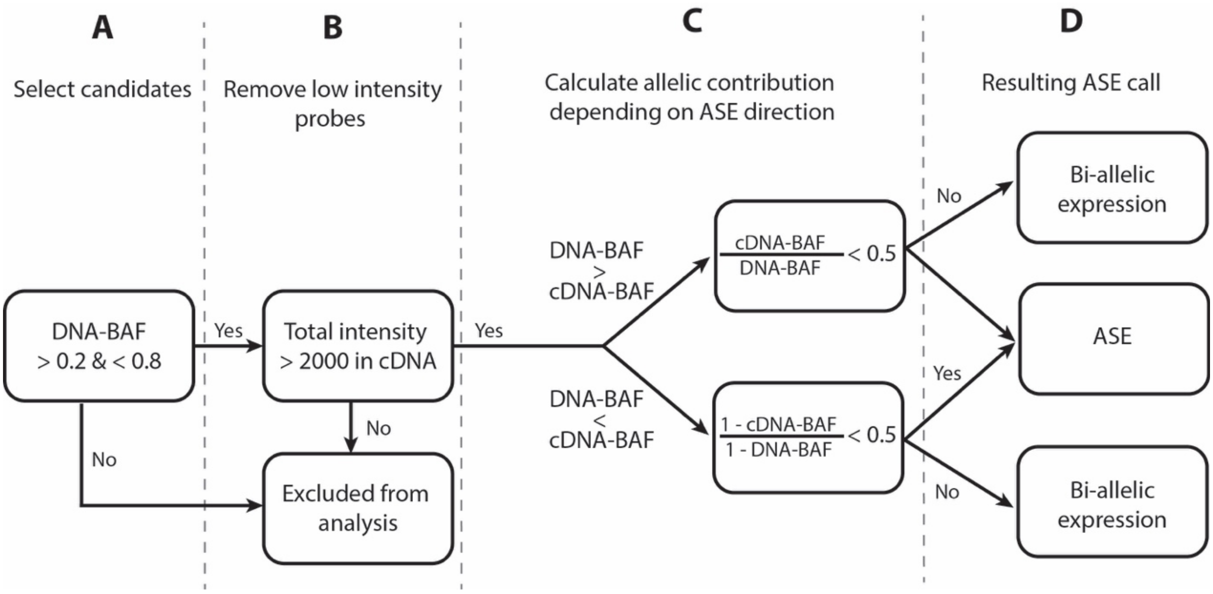

Figure S5: **ASE detection flowchart**.

Consistency of ASE calling in genes with multiple candidate cSNPs

For 7.3% of genes with multiple candidate cSNPs inconsistent ASE calls were found between candidate cSNPs. To further examine this we examined ASE results for *MUC16*. Eight cell lines had least 4 candidate cSNPs, Figure S6A shows the ASE score per candidate probe. Based on these results we concluded that for JVE192 and KP7038T are the only cell lines showing ASE of *MUC16*. This was confirmed when plotting the β-allele-frequencies of the DNA and cDNA (Figure S6B). We therefore chose to use the average ASE score for all candidate cSNPs per gene to determine ASE.

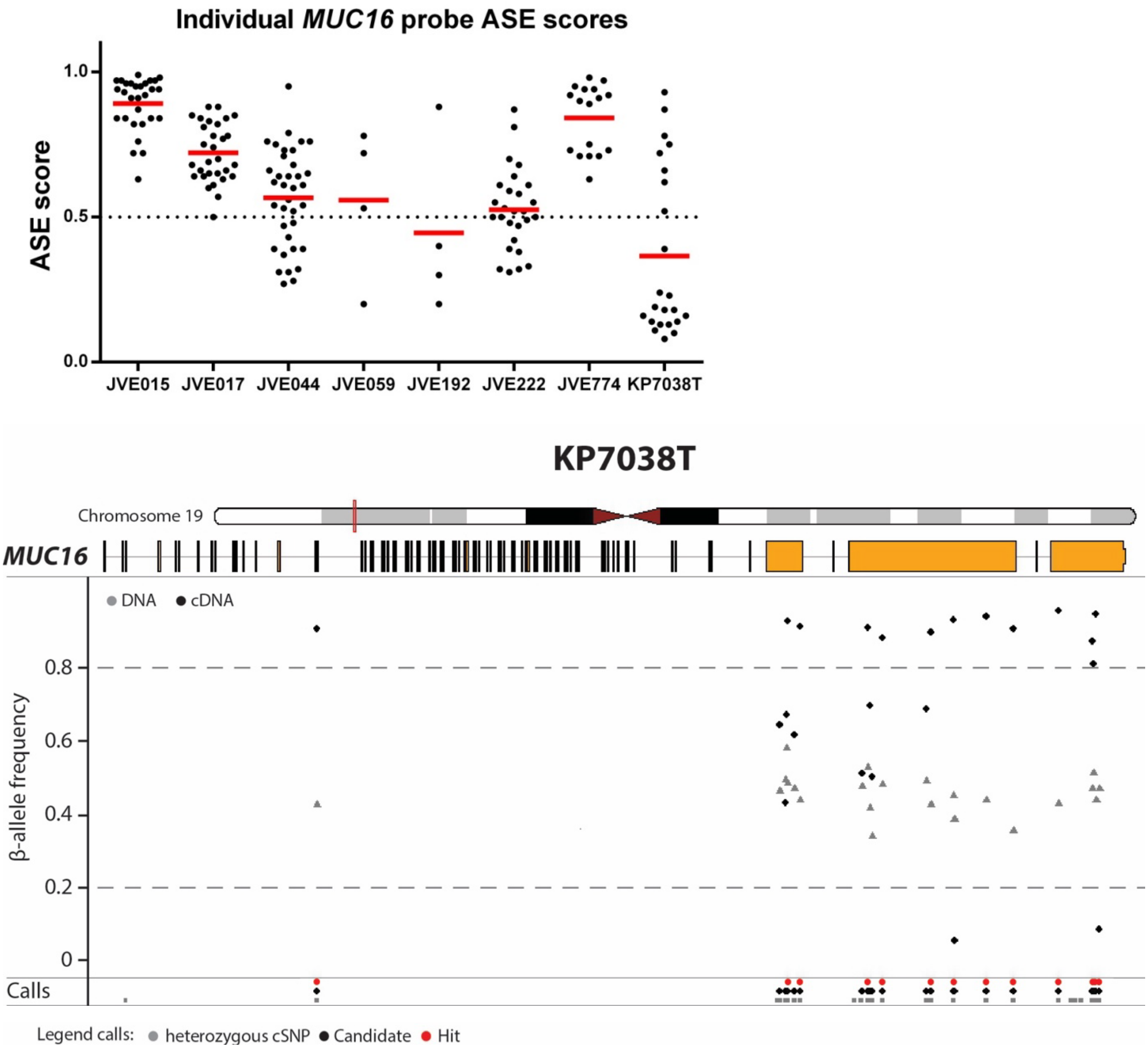

Figure S6: Inconsistent ASE results for *MUC16*. 8 samples with 4 or more candidate cSNPs in *MUC16* show high variability of ASE score between probes (Figure S6A). To further explore this we plotted the β-allele-frequencies of the DNA and cDNA samples (top panel) (Figure S6B). The bottom panel shows the distribution of heterozygous cSNPs (grey), candidate cSNPs (black) and ASE hits (red). The red line in S4A represents the average ASE score of all candidate cSNPs per sample, used in further analyses for all genes with multiple candidate cSNPs.

Supplementary table 1: ASE results per cell line for all genes with at least 1 heterozygous sample

Gene	CHR	MapInfo	JVE015	JVE017	JVE044	JVE059	JVE103	JVE109	JVE114	JVE127	JVE187	JVE192	JVE207	JVE222	JVE241	JVE253	JVE367	JVE371	JVE528	JVE774	KP283T	KP363T	KP7038T	Gene_ASE_conclusion
SAMD11	1	861349																	0					No-ASE
NOC2L	1	880160			0	0		0		0				0				0		0	0			No-ASE
PLEKHN1	1	901922			0	0		0		0		0			0	0		0		0	0		1	No-ASE
ISG15	1	949422			0	0						0						0		0			1	No-ASE
AGRN	1	970687		1		0		0										0		0				No-ASE
C1orf159	1	1021285														0								No-ASE
TTLL10	1	1114668			0					0	0			0				0						No-ASE
FAM132A	1	1177981														0								No-ASE
CPSF3L	1	1247454																	0					No-ASE
TAS1R3	1	1266738						0										0						No-ASE
DVL1	1	1271808				0																		No-ASE
CCNL2	1	1322654								0					0									No-ASE
ANKRD65	1	1354515																0						No-ASE
ATAD3C	1	1386089																	1					ASE
ATAD3B	1	1412659																0					0	No-ASE
MIB2	1	1550992			0						0				0			0	0	0				No-ASE
NADK	1	1686024																0		0				No-ASE
CALML6	1	1847886																	0					No-ASE
TMEM52	1	1849530						0								1			0					Heterogeneous_ASE
KIAA1751	1	1887019				0					0							0						No-ASE
PEX10	1	2337219									0													No-ASE
PANK4	1	2440415			0							0				0								No-ASE
LOC100133445	1	2488153				0				0								0	0					No-ASE
TNFRSF14	1	2491306								0		0			0									No-ASE
MMEL1	1	2523015				1						0			0			0						No-ASE
PRDM16	1	3102751				0		0										0					1	No-ASE
ARHGEF16	1	3380057																	0					No-ASE
MEGF6	1	3407112			0	1		0		0	0			0		0		0	0	0			0	No-ASE
CCDC27	1	3669100																0						No-ASE
CEP104	1	3732862						0		0		0				0			0	0				No-ASE
C1orf174	1	3806544																0						No-ASE
AJAP1	1	4772034																0						No-ASE
NPHP4	1	5923427																0	0					No-ASE
KCNAB2	1	6100645				0																		No-ASE
RNF207	1	6269069			0	0				0	0			0	0			0			0			No-ASE
TNFRSF25	1	6522218			0	0		0		0														No-ASE
PLEKHG5	1	6528069		1	0	0		0		0				0					0					No-ASE
NOL9	1	6585940						0						0	0			0	0	0				No-ASE
TAS1R1	1	6615444			1			1		1				1							1		1	ASE
ZBTB48	1	6641060				0												0		0				No-ASE
CAMTA1	1	6880304								0	0				0									No-ASE
PER3	1	7844989				0				0				1					0					No-ASE
UTS2	1	7907918								0	0			0	1						0		0	No-ASE
ERRFI1	1	8073398																0			0			No-ASE
SLC45A1	1	8384785								0					0									No-ASE
CA6	1	9009352						1										0			1		1	ASE
SLC2A7	1	9063478														1								ASE
GPR157	1	9164626			0			0				0						0		0	0			No-ASE
H6PD	1	9305309						0			0	0						0	0					No-ASE
TMEM201	1	9655965			0	0						0						0		0				No-ASE
PIK3CD	1	9770571																	1					ASE
CLSTN1	1	9790653			0						0			0		0		0	0				0	No-ASE
KIF1B	1	10327447																0						No-ASE
DFFA	1	10521629			0														0					No-ASE
C1orf127	1	11007724				1		0			0				0									No-ASE
MASP2	1	11086950									0							0						No-ASE
PTCHD2	1	11561057				0		1																Heterogeneous_ASE
DRAXIN	1	11766424																0						No-ASE
MTHFR	1	11850750			0	0		0										0			0			No-ASE
NPPA-AS1	1	11906068			0			0			0					0			0					No-ASE
PLOD1	1	12008093			0	0										1			0					No-ASE
MFN2	1	12049283																			0			No-ASE
MIIP	1	12081895		0	0			0			0	0		0	0	0			0		0		0	No-ASE
TNFRSF8	1	12164451										0												No-ASE
TNFRSF1B	1	12251141			0							0			0					0				No-ASE
VPS13D	1	12294420			0	0					0	0						0	0				0	No-ASE
PRAMEF2	1	12919081																0						No-ASE
PRDM2	1	14059374			0			0				0			0	0				0			0	No-ASE
KAZN	1	15287201				0		0			0							0		0				No-ASE
TMEM51	1	15541621			0													0		0			0	No-ASE
FHAD1	1	15578302			0	0		0			0	0			0	0			1	0	0		0	No-ASE
CELA2B	1	15808767						0			0	0			0			0						No-ASE
CASP9	1	15820448									0	0			0	0		0		0	0		0	No-ASE
AGMAT	1	15904318						0			0	0			0	0		0		0	0		0	No-ASE
PLEKHM2	1	16042766			0			0			0				0	0			0	0			0	No-ASE
FBLIM1	1	16091591										0												No-ASE
UQCRHL	1	16134072																	1					ASE
SPEN	1	16199526				0					0	0		0	0			0	0				0	No-ASE
HSPB7	1	16342103				0												0	1				1	Heterogeneous_ASE
CLCNKA	1	16349137																0						No-ASE
CLCNKB	1	16371067									0	0		0	0					0				No-ASE
EPHA2	1	16451722									0	0			0			0					0	No-ASE
ARHGEF19	1	16525657										0		0	0	0		0					0	No-ASE
RSG1	1	16558641																	0					No-ASE
FBXO42	1	16577377				0		0			0			0	0	0		0	0				0	No-ASE
SPATA21	1	16725270			0							0			0	0		0	0				0	No-ASE
NBPF1	1	16890470																0						No-ASE
CROCC	1	17248532												0									1	Heterogeneous_ASE
ATP13A2	1	17312485			0	0								0		0		0					0	No-ASE
PADI2	1	17395480			0											0		0			0		0	No-ASE
PADI3	1	17575699			0			1			0													Heterogeneous_ASE
PADI4	1	17634718				0					1	1		0	0			0						Heterogeneous_ASE
ARHGEF10L	1	17913964			0			0				0			0			0	0	0			0	No-ASE
KLHDC7A	1	18807521			0						0	0		0				0	1	0	0		1	No-ASE
TAS1R2	1	19166100																0						No-ASE
ALDH4A1	1	19199448				0		0				0		0	0	0		0	0		0		0	No-ASE
UBR4	1	19401331			0											0		0		0	0		0	No-ASE
EMC1	1	19550017			0			0			0				0	0		0	0		0		0	No-ASE
AKR7A3	1	19609262						0												0			1	Heterogeneous_ASE
AKR7A2	1	19633520																					0	No-ASE
PQLC2	1	19644218						0							0									No-ASE
TMCO4	1	20009745				0		0			0	0		0	0				0	0			0	No-ASE
RNF186	1	20140973												0		0								No-ASE
OTUD3	1	20216965				0																		No-ASE
PLA2G2D	1	20440684																		0				No-ASE
PLA2G2C	1	20490518																0		0				No-ASE
VWA5B1	1	20639370																	1					ASE
MUL1	1	20827377																			0			No-ASE
CDA	1	20915701				1												0			0		1	Heterogeneous_ASE
PINK1	1	20960259				0		0				0		0	0	0		0			0			No-ASE
KIF17	1	20992819			0	0								0	0	0		0	1	0	0		1	No-ASE
ECE1	1	21546459										0												No-ASE
NBPF3	1	21795375			0	0						0			0			0		0	0			No-ASE
RAP1GAP	1	21924217				0						0			0								0	No-ASE
LDLRAD2	1	22140931			0						0							0		0			0	No-ASE
HSPG2	1	22154365			0						0			0	0	0		0	0	0			0	No-ASE
WNT4	1	22446566												0										No-ASE

ZBTB40	1	22816686			0	0		0						0		0			0		0		0	No-ASE
EPHA8	1	22895820																0						No-ASE
KDM1A	1	23380259																	0					No-ASE
LUZP1	1	23415426														0			0				0	No-ASE
E2F2	1	23836407			0	0		0				0							0		0		0	No-ASE
ID3	1	23885480			0							0								0	0		0	No-ASE
TCEB3	1	24077388										0			0					0				No-ASE
FUCA1	1	24172332			0			0							0			0	0		0		0	No-ASE
CNR2	1	24201065						0							0	0		0	0				0	No-ASE
MYOM3	1	24383873			0	0		0			0			0				0	0	0	0		0	No-ASE
IL22RA1	1	24447468				0					0	0		0					0	0	0		0	No-ASE
IFNLR1	1	24483832		0																	0			No-ASE
GRHL3	1	24657930			0	0		0			0	0		0				0	0	0	0		0	No-ASE
STPG1	1	24696293			0						0	0		0				0	0	0				No-ASE
C1orf63	1	25570081				0					0			0		0		0			0		0	No-ASE
RHD	1	25627470				1						1		1						1			1	ASE
RHCE	1	25712227													0									No-ASE
LDLRAP1	1	25880437				0						0								0				No-ASE
SEPN1	1	26131638						0						0		0			0				0	No-ASE
AUNIP	1	26161593				0																		No-ASE
PAFAH2	1	26288523																		0				No-ASE
EXTL1	1	26349187													0									No-ASE
TRIM63	1	26380423																		0				No-ASE
PDIK1L	1	26450009						0						0	0	0			0		0			No-ASE
CNKSR1	1	26507075				0													0				0	No-ASE
CATSPER4	1	26517216				0						0			0			0						No-ASE
CEP85	1	26566299			0						0	0			0	0			0	0	0		0	No-ASE
UBXN11	1	26608935			0																			No-ASE
CD52	1	26646730			0						0				0						0		0	No-ASE
AIM1L	1	26648381			0	0		0			0	0		0	0	1		0	0	0	1		0	No-ASE
ZNF683	1	26688125																0						No-ASE
DHDDS	1	26764719				0		0							0	0			0		0			No-ASE
RPS6KA1	1	26863453										0								0				No-ASE
NR0B2	1	27238451				0																		No-ASE
C1orf172	1	27276604													0									No-ASE
FAM46B	1	27332466																			0			No-ASE
MAP3K6	1	27682530			0	0		0				0			0				0	0	0		0	No-ASE
CD164L2	1	27708996										0			0								0	No-ASE
AHDC1	1	27873895												0										No-ASE
THEMIS2	1	28206250				0		1				0			0			0	0		0			No-ASE
RPA2	1	28218100				0		0				0								0	0		0	No-ASE
SMPDL3B	1	28271744						0			0	0		0							0			No-ASE
ATPIF1	1	28562906			0	0								0				0	0					No-ASE
PHACTR4	1	28785658			0	0		0											0					No-ASE
SRSF4	1	29475296										0											0	No-ASE
MECR	1	29519778										0											0	No-ASE
PTPRU	1	29581894										0						0						No-ASE
LAPTM5	1	31206719			0	0		0						0	0						0			No-ASE
SDC3	1	31346102			0							0												No-ASE
FABP3	1	31838767														0								No-ASE
HCRTR1	1	32084861				0												0						No-ASE
COL16A1	1	32119265														1		0					1	Heterogeneous_ASE
BAI2	1	32193044			0			0																No-ASE
SPOCD1	1	32256329																0						No-ASE
CCDC28B	1	32667609												0									0	No-ASE
TMEM234	1	32682471			0																			No-ASE
ZBTB8A	1	33058596				0		0				0			0			0						No-ASE
KIAA1522	1	33233498						0			0	0							0		0			No-ASE
RNF19B	1	33402504		0							0													No-ASE
PHC2	1	33794564						0				0	0	0	0	0			0	0			0	No-ASE
ZSCAN20	1	33944993										0									0			No-ASE
CSMD2	1	33985177																0						No-ASE
C1orf94	1	34643413																0						No-ASE
GJB5	1	35222995				0																		No-ASE
GJB4	1	35226892		0															0					No-ASE
GJB3	1	35250457																	0					No-ASE
GJA4	1	35260202			1	0					0	0							0	0			1	Heterogeneous_ASE
ZMYM1	1	35559598																		0				No-ASE
TFAP2E	1	36040566																		0				No-ASE
PSMB2	1	36068872													0									No-ASE
CLSPN	1	36202585		0		0							0						0	0				No-ASE
MAP7D1	1	36636671										0												No-ASE
SH3D21	1	36773761																	0					No-ASE
STK40	1	36807416				0									0			0		0			0	No-ASE
OSCP1	1	36886095				0						0	0	0		0		0		0	0		0	No-ASE
MEAF6	1	37958813													0			0		0				No-ASE
DNALI1	1	38023316										1												ASE
EPHA10	1	38184055		0					0				0	0	0			0	0		0			No-ASE
MANEAL	1	38262417																	0					No-ASE
MTF1	1	38280866			0			0	0									0		0	0			No-ASE
INPP5B	1	38329999			0							0			0	0			0		0		0	No-ASE
FHL3	1	38463148											0						1					Heterogeneous_ASE
UTP11L	1	38478503																0						No-ASE
GJA9-MYCBP	1	39332586			0	0					0	0	0	0	0	0		0			0		0	No-ASE
RHBDL2	1	39352271			0	0					0	0	0	0	0	0		0			0		1	No-ASE
MACF1	1	39715702		0	0	0		0	0		0		0	0	0			0	0		0		0	No-ASE
PPIE	1	40209586		0																0				No-ASE
TRIT1	1	40307584				0														0				No-ASE
PPT1	1	40539750																0	0					No-ASE
RLF	1	40627183		0	0				0						0	0		0	0	0				No-ASE
COL9A2	1	40766942										0						0						No-ASE
ZFP69B	1	40923018						1										0			0			Heterogeneous_ASE
ZFP69	1	40945067									1					0								Heterogeneous_ASE
EXO5	1	40980559		0	0	0					0	0	1			0		0	1	0	0			No-ASE
ZNF684	1	41012533				0																		No-ASE
NFYC	1	41204569		0	0				0			0	0	0					0		0			No-ASE
KCNQ4	1	41282938		0								0												No-ASE
CITED4	1	41327281														0								No-ASE
SLFNL1-AS1	1	41482960		0	0			0			0				0	0		0						No-ASE
HIVEP3	1	41976132			0							0		0					0	0			0	No-ASE
GUCA2A	1	42628581			0			0			0							0			0		0	No-ASE
FOXJ3	1	42645389		0	0	0		0	0		0	0			0			0			0		0	No-ASE
RIMKLA	1	42865200		0	0						0	0				0		0	0	0	0		0	No-ASE
ZMYND12	1	42896432										0		0	0						0			No-ASE
CLDN19	1	43200774													0									No-ASE
LEPRE1	1	43212926		0				0			0										0			No-ASE
C1orf50	1	43232984														0								No-ASE
ERMAP	1	43296130			0	0			0									0						No-ASE
EBNA1BP2	1	43630315		0								0							0	0			0	No-ASE
WDR65	1	43638455		0	0	0			0			0						0		0			0	No-ASE
TMEM125	1	43738488																			0			No-ASE
SZT2	1	43868957		0	0	0		0										0	0		0		0	No-ASE
KDM4A	1	44118920										0			0								0	No-ASE
ST3GAL3	1	44202014		0	0	0		0	0			0	1	0		0		0	0		0			No-ASE
ARTN	1	44401353										0												No-ASE
B4GALT2	1	44446937										0							0					No-ASE
CCDC24	1	44457957										0												No-ASE
KLF17	1	44584638		1		0		0	1											0			1	Heterogeneous_ASE

KIF2C	1	45205606			0															0				No-ASE
UROD	1	45478832		0					0					0										No-ASE
ZSWIM5	1	45484448																			0			No-ASE
MUTYH	1	45795027			0	0		0							0					0				No-ASE
TOE1	1	45806750			0														0					No-ASE
TESK2	1	45810531				0		0																No-ASE
AKR1A1	1	46027477																	0					No-ASE
IPP	1	46160124			0		1								0				0	0	0			No-ASE
MAST2	1	46295147			0	0					0	0	0		0	0			0	0	0			No-ASE
PIK3R3	1	46509416			0								0		0	0			0	0	0			No-ASE
POMGNT1	1	46655130		0		1																		Heterogeneous_ASE
LRRC41	1	46744712				0										0				0				No-ASE
UQCRH	1	46774783				0					0									0				No-ASE
NSUN4	1	46810530		0	0	0		0	0		0	0			0					0			0	No-ASE
MKNK1-AS1	1	47013404						1			0				1									Heterogeneous_ASE
MOB3C	1	47075329			0			0	0		0	0	0			0		0		0				No-ASE
TEX38	1	47138637						0	0		0	0							0		0			No-ASE
EFCAB14-AS1	1	47149007																	0					No-ASE
CYP4B1	1	47264766									1								0					Heterogeneous_ASE
CYP4A22	1	47603188		0				0						0		0					0	0		No-ASE
STIL	1	47716980			0	0		0			0	0		0					0	0	0	0	0	No-ASE
CMPK1	1	47834152						0																No-ASE
SLC5A9	1	48688550													0				0					No-ASE
SPATA6	1	48764419			0						0	0	0		0	0					0			No-ASE
EPS15	1	51826853		0	0	0		0				0	0			0				0	0	0		No-ASE
NRD1	1	52257317		0																				No-ASE
TXNDC12	1	52492993																		0				No-ASE
ZFYVE9	1	52703169						0																No-ASE
CC2D1B	1	52820567						0																No-ASE
ORC1	1	52838992										0												No-ASE
SELRC1	1	53153432		0	0	0							0	0	0	0				0	0	0		No-ASE
ZYG11A	1	53308575										1												ASE
SLC1A7	1	53553754			0	0		0							0						0			No-ASE
CPT2	1	53666456		0	0	0									0	0				0	0	0		No-ASE
LRP8	1	53712727		0		0		0				0			0	0		0		0	0	0		No-ASE
GLIS1	1	53972360				0													1					Heterogeneous_ASE
YIPF1	1	54331901										0			0			0	0	0	0			No-ASE
LRRC42	1	54417796			0			0													0		0	No-ASE
CDCP2	1	54605242		0		0		0				0		0										No-ASE
CYB5RL	1	54640318		0	0	0								0	0	0				0	0	0	0	No-ASE
MRPL37	1	54666074				0		0				0						0			0	0		No-ASE
ACOT11	1	55014013			0	0		0				0	0		0			0		0		0	0	No-ASE
HEATR8-TTC4	1	55118790		0	0	0		0				0	0	0	0	0		0	0	0	0	0		No-ASE
PARS2	1	55223536						0							0			0						No-ASE
TTC22	1	55248059				0		0				0		0				0			0	0		No-ASE
C1orf177	1	55271845				0								0		0		0			0	0		No-ASE
DHCR24	1	55317935				0						0						0				0	0	No-ASE
PCSK9	1	55505647		1		0		0				0						0				0	0	No-ASE
USP24	1	55541174				0		0				0		0						0				No-ASE
C8A	1	57320444												0										No-ASE
C8B	1	57395114										1												ASE
TACSTD2	1	59041930																	1					ASE
MYSM1	1	59125683		0	0	0		0						0	0	0		0	0				0	No-ASE
FGGY	1	59787270		0	0	0		0								0			0	0	0		0	No-ASE
INADL	1	62228696		0	0	0		0				0		0	0	0		0	0	0	0		0	No-ASE
L1TD1	1	62672381						0				0												No-ASE
KANK4	1	62703977													0						1			Heterogeneous_ASE
ALG6	1	63872032		0		0								0				0		0	0			No-ASE
EFCAB7	1	63991322			0								0		0			0	0	0				No-ASE
PGM1	1	64059266		0		0										0		0	1					No-ASE
ROR1	1	64475021		0					0							0								No-ASE
CACHD1	1	65016278						0																No-ASE
LEPR	1	66036441		0	0	0													0	0			1	No-ASE
SGIP1	1	67091560																0						No-ASE
WDR78	1	67279835										0				0								No-ASE
MIER1	1	67424637											0								0			No-ASE
SLC35D1	1	67486092			0																			No-ASE
IL23R	1	67633812				0			0					0	0					0	0		1	No-ASE
GNG12-AS1	1	68512899		0	0	0									0						0		0	No-ASE
ANKRD13C	1	70742505						0								0								No-ASE
CTH	1	70877113											0											No-ASE
LRRIQ3	1	74492543							1									0						Heterogeneous_ASE
FPGT	1	74663985		0	0			0				0	0					0		0				No-ASE
C1orf173	1	75036783												1	0									Heterogeneous_ASE
CRYZ	1	75172042							0			0	0									0		No-ASE
TYW3	1	75204416						0	0			0	0	0					0	0	0			No-ASE
SLC44A5	1	75677192			1							0	1	1	1			1						ASE
ACADM	1	76198337		1		0																		Heterogeneous_ASE
MSH4	1	76262773						0				0		1										Heterogeneous_ASE
ASB17	1	76384659												0										No-ASE
ST6GALNAC3	1	76877767																					1	ASE
PIGK	1	77627018																	0					No-ASE
USP33	1	78178961		0	0				0							0								No-ASE
GIPC2	1	78511980										1	0								0			Heterogeneous_ASE
IFI44L	1	79093737		1		0									0					0	0			No-ASE
ELTD1	1	79357360														0								No-ASE
RPF1	1	84944975		0	0																			No-ASE
CTBS	1	85020683			0								0		0					0	0			No-ASE
SSX2IP	1	85113229		0	0	0			0	0		0	0	0	0				0	0			0	No-ASE
WDR63	1	85537661																					1	ASE
SYDE2	1	85624470		0				0								0								No-ASE
BCL10	1	85733374		0																				No-ASE
CYR61	1	86048526		0																	0			No-ASE
ZNHIT6	1	86123563				0			0															No-ASE
CLCA2	1	86889944										1			0						0			Heterogeneous_ASE
CLCA1	1	86934676										1												ASE
42248	1	87329165				0																		No-ASE
PKN2	1	89206740			0	0			0			0				0			0				0	No-ASE
GBP3	1	89473453		0		0		1	0				1						0	0				Heterogeneous_ASE
GBP2	1	89573902										0	0	0	0	0			0					No-ASE
GBP4	1	89650945			0																			No-ASE
GBP5	1	89726398												1										ASE
GBP6	1	89834182																			0			No-ASE
LRRC8C	1	90178335		0	0	0		0				0				1		0	0	0			0	No-ASE
ZNF644	1	91382455																			0			No-ASE
TGFBR3	1	92149303			0							1	0	0						0				No-ASE
BTBD8	1	92546144														0								No-ASE
KIAA1107	1	92633429			0			0	0			0	0	0	0	0		0	0				0	No-ASE
GFI1	1	92941657										0	0											No-ASE
EVI5	1	92979254		0	0							0	0	0	0	0		0					0	No-ASE
TMED5	1	93620318				0						0							0					No-ASE
CCDC18	1	93651937				0						0				0			0					No-ASE
DNTTIP2	1	94335432			0	0		0					0		0				0		0			No-ASE
ABCA4	1	94461717		0														0						No-ASE
ARHGAP29	1	94639856																		0				No-ASE
F3	1	94996062																	0					No-ASE
SLC44A3	1	95286518			0							0						0	0	0			0	No-ASE
CNN3	1	95363001		0										0									0	No-ASE

TMEM56-RWDD3	1	95699811			0	0						0				0								No-ASE
DPYD	1	97544543			0							1								1				Heterogeneous_ASE
SNX7	1	99150473				0																		No-ASE
MIR548AA1	1	100174455						0																No-ASE
FRRS1	1	100182997						0	0			0			0					0				No-ASE
AGL	1	100316589		0	0	0		0	0				0			0		0		0			0	No-ASE
HIAT1	1	100534070				0						0						0			0		0	No-ASE
SASS6	1	100550937				0			0			0	0	0				0			0		0	No-ASE
TRMT13	1	100598738			0			0											0					No-ASE
DBT	1	100661842			0			0											0					No-ASE
CDC14A	1	100818541																	0					No-ASE
DPH5	1	101456010				0																		No-ASE
PRMT6	1	107599482						1	0						0									Heterogeneous_ASE
VAV3	1	108116715			0									0		0							0	No-ASE
FNDC7	1	109255566		0	0	0		0	0			0		0	0			0			0		0	No-ASE
AKNAD1	1	109358897		0	0				0			1		0	0	0		0	0	0	0		1	No-ASE
GPSM2	1	109440129							0					0		0								No-ASE
CLCC1	1	109477313				0			0					0										No-ASE
C1orf194	1	109649162																0						No-ASE
KIAA1324	1	109656870		0	0	0		0				0		0		0		0	0		0			No-ASE
CELSR2	1	109792784		0		0						0						0	0	0	0		0	No-ASE
MYBPHL	1	109837810				0						0							1					Heterogeneous_ASE
SYPL2	1	110018267																					0	No-ASE
GNAT2	1	110146110		0																				No-ASE
GSTM1	1	110233104															1							ASE
GSTM3	1	110279701		0	1	0		0	0			0		1						1			0	Heterogeneous_ASE
EPS8L3	1	110293310		0	0	0		0							0	0			0	0	0		0	No-ASE
CSF1	1	110460068				0										0		0					0	No-ASE
SLC6A17	1	110709720																0						No-ASE
KCNC4	1	110765691							0			0								0				No-ASE
RBM15	1	110882052																		0			0	No-ASE
SLC16A4	1	110906406														0								No-ASE
LAMTOR5	1	110946580		0	0							0		0	0			0	0		0			No-ASE
PROK1	1	110993852																0						No-ASE
LRIF1	1	111490670													0						1			Heterogeneous_ASE
CHI3L2	1	111773462										0												No-ASE
CHIA	1	111854859																0						No-ASE
PIFO	1	111889283		1				0								0			1	0			0	Heterogeneous_ASE
OVGP1	1	111957097						0							0				0					No-ASE
C1orf162	1	112018657				0		0							0				0					No-ASE
ADORA3	1	112026370																					0	No-ASE
DDX20	1	112298581		0	0							0		0						0	0			No-ASE
CTTNBP2NL	1	112991675		0								0												No-ASE
ST7L	1	113068651			0	0			0			0		0					0		0		0	No-ASE
CAPZA1	1	113162482			0									0									0	No-ASE
MOV10	1	113232218														0								No-ASE
PPM1J	1	113252811						0																No-ASE
SLC16A1	1	113456546						0	0					0	0			0		0	0		0	No-ASE
MAGI3	1	113933675																					0	No-ASE
PTPN22	1	114367793													0									No-ASE
AP4B1-AS1	1	114423653		0	0	0			0						0						0		0	No-ASE
DCLRE1B	1	114448279				0																		No-ASE
HIPK1	1	114497331							0					0	0			0						No-ASE
TRIM33	1	114940386						0											0					No-ASE
DENND2C	1	115130363			0	0						0		0	0			0		0	0			No-ASE
VANGL1	1	116202263				0										0								No-ASE
SLC22A15	1	116519313			0																			No-ASE
ATP1A1	1	116937809																0						No-ASE
IGSF3	1	117120134				0		1				1			0						0	0		Heterogeneous_ASE
PTGFRN	1	117487567							0					0	0	0		0	0		0	0	0	No-ASE
CD101	1	117552529			0	0						0		0	0	0		0			0	0	0	No-ASE
TTF2	1	117603083		0		0		0	0			0		0	0	0			0			0	0	No-ASE
TRIM45	1	117656026			0				0			0		0					0			0		No-ASE
FAM46C	1	118165536														0								No-ASE
GDAP2	1	118420768																	0					No-ASE
WDR3	1	118476052																				0		No-ASE
SPAG17	1	118506432			1									0										Heterogeneous_ASE
TBX15	1	119427467												0						1			1	Heterogeneous_ASE
WARS2	1	119575538										0												No-ASE
HAO2	1	119923751																	1					ASE
NOTCH2	1	120458122						0				0												No-ASE
SEC22B	1	145103949																1						ASE
PEX11B	1	145517063		0												0								No-ASE
ITGA10	1	145525085		0		0											0			0				No-ASE
ANKRD35	1	145549328													0			0						No-ASE
PIAS3	1	145576091												0										No-ASE
NUDT17	1	145586596									0	0	0											No-ASE
CHD1L	1	146727553	0			0	0	0			0	0		0			0				0			No-ASE
LINC00624	1	146854836					0																	No-ASE
BCL9	1	147084683	0		0	0	0	0		0		0	0	0		0				0			0	No-ASE
ACP6	1	147119257	0	0		0	0	0			1	0	0	0	0	0			0	0			0	No-ASE
SF3B4	1	149897906												0										No-ASE
MTMR11	1	149901088		0		0		0		0			0	0	0			0		0		0		No-ASE
C1orf51	1	150255712									1													ASE
RPRD2	1	150416763																				0		No-ASE
TARS2	1	150459992																		0				No-ASE
ECM1	1	150482145		0	0	0	0	0			0	0			0									No-ASE
ADAMTSL4	1	150525425					0													0			1	Heterogeneous_ASE
ENSA	1	150598965			0	0	0	0						0										No-ASE
HORMAD1	1	150672619					0																	No-ASE
CTSS	1	150722511	0	0	0	0	0	0	0	0	0	0			0	0				0			0	No-ASE
SETDB1	1	150900260						0														0		No-ASE
CERS2	1	150939279						0	0		0	0	0							0			0	No-ASE
ANXA9	1	150955582						0	0		0	0	0										0	No-ASE
FAM63A	1	150969567	0																					No-ASE
BNIPL	1	151009208		0	0	0				0	0		0	0		0				0		0	0	No-ASE
GABPB2	1	151062922				0					0													No-ASE
SEMA6C	1	151105169				0	0	0									0	0				0	0	No-ASE
ZNF687	1	151259113				0	0		0		0	0												No-ASE
RFX5	1	151314683		0	0					0			0		0							0	0	No-ASE
PSMB4	1	151372065			0			0			0	0							0					No-ASE
CGN	1	151491026				0	0					0		0						0			0	No-ASE
TUFT1	1	151512879				0	0												0				0	No-ASE
SNX27	1	151633309		0	0		0		0	0		0				0	0					0	0	No-ASE
MRPL9	1	151732552	0	0	0	0	0			0		0				0	0		0			0	0	No-ASE
THEM5	1	151820298		0				0		0	0		0										0	No-ASE
TCHH	1	152079989		1	0		0	0		0			1	0	0	1			0				0	Heterogeneous_ASE
RPTN	1	152127348							1	0		0	1	0		0							0	Heterogeneous_ASE
HRNR	1	152185628	0						0		0									0			1	No-ASE
FLG	1	152275177	0	1							0								1	0				Heterogeneous_ASE
FLG2	1	152323087					0				0			0						0				No-ASE
CRCT1	1	152487917					0																	No-ASE
C1orf68	1	152692074					0																	No-ASE
KPRP	1	152732105					0																	No-ASE
IVL	1	152882302			0									0										No-ASE
SPRR3	1	152975555			0		0	0	1	0	0			0				0						No-ASE
SPRR1B	1	153004848			0		0	0		0				0	0			0		0				No-ASE
PGLYRP3	1	153270461	0			0	0							0				0					0	No-ASE

PGLYRP4	1	153303326		1	1		0							1								1		ASE
S100A7L2	1	153409674																0						No-ASE
S100A5	1	153509890						0			0										0			No-ASE
CHTOP	1	153614810												0								0		No-ASE
NPR1	1	153652129					0																	No-ASE
CREB3L4	1	153941005		0					0	0							0					0		No-ASE
RAB13	1	153954646					0								0		0							No-ASE
NUP210L	1	153965322		0													0					0		No-ASE
AQP10	1	154295592				1																		ASE
IL6R	1	154401780			0	0			0			0						0					1	No-ASE
TDRD10	1	154493878				1	0					0			0			0						No-ASE
ADAR	1	154557735	0	0	0							0		0	0								0	No-ASE
PBXIP1	1	154918182					0				0	0	0				0	0	0	0			0	No-ASE
SHC1	1	154936389																					0	No-ASE
DCST2	1	154991027												0										No-ASE
LOC100505666	1	155025186	0				0	0			0	0		0	0	0		0	0				0	No-ASE
EFNA3	1	155057650		0		0	1	0					0	0			0					0		No-ASE
EFNA1	1	155103819	0			0	0	0	0	0			0	0			0	0			0	0	0	No-ASE
MUC1	1	155158634	0						1					1			1	0						Heterogeneous_ASE
MTX1	1	155178993												0										No-ASE
GBA	1	155205047													0									No-ASE
SCAMP3	1	155226107						0		0			0	0						0				No-ASE
CLK2	1	155233763												0										No-ASE
HCN3	1	155247518			0																			No-ASE
PKLR	1	155260382					0															0		No-ASE
RUSC1-AS1	1	155290587																	0					No-ASE
RUSC1	1	155294666																	0					No-ASE
YY1AP1	1	155629930		0								0												No-ASE
GON4L	1	155723008		0	0			0		0	0		0											No-ASE
RIT1	1	155868625		0	0	0		0		1	0		0					0	1					No-ASE
UBQLN4	1	156011427		0	0	0		0	0	0			0		0			0					1	No-ASE
RAB25	1	156035717																	0					No-ASE
LMNA	1	156100408				0	0	0			0		0	0	0				0	0	0	0		No-ASE
SEMA4A	1	156127862				0																0		No-ASE
PMF1-BGLAP	1	156182816	0		0			0	0	0			0				0					0		No-ASE
TMEM79	1	156255022				0							0	0			0					0		No-ASE
C1orf85	1	156262905	0			0		0					0	0			0					0		No-ASE
CCT3	1	156280881																					0	No-ASE
TSACC	1	156309555						0																No-ASE
RHBG	1	156339047	0		0			0				0	1	0	0			0	1				0	No-ASE
IQGAP3	1	156496297	0		0		0		0	0	0	0		0		0		0	0	0		0		No-ASE
TTC24	1	156551248													0							1		Heterogeneous_ASE
BCAN	1	156615902		0	0	0			0	0	0	0		0				0		0		0	0	No-ASE
NES	1	156639215		1	0	0	0						0	0			0	0						No-ASE
CRABP2	1	156670334					0																	No-ASE
ISG20L2	1	156696974																		0				No-ASE
HDGF	1	156713234			0			0	0					0	0							0		No-ASE
SH2D2A	1	156776968					0																	No-ASE
NTRK1	1	156810763						0																No-ASE
PEAR1	1	156875126	0		0	0	0	0	0		0				0	0					0	0		No-ASE
LRRC71	1	156890672	0	0		0					0		0	0	0		0		0					No-ASE
ARHGEF11	1	156905823	0	0	0	0		0	0		0		0	0	0	0	0				0			No-ASE
ETV3L	1	157062575					0											0						No-ASE
FCRL5	1	157485429					0											0						No-ASE
FCRL3	1	157648543					0											0				1		Heterogeneous_ASE
CD1A	1	158224516													0									No-ASE
CD1E	1	158323779																0						No-ASE
OR10T2	1	158368321				1									1									ASE
OR10R2	1	158449749					0																	No-ASE
OR10X1	1	158548803		1	1				1						1									ASE
OR10Z1	1	158576394					0																	No-ASE
SPTA1	1	158581131					0																	No-ASE
OR6K3	1	158686980					0															1		Heterogeneous_ASE
OR6K6	1	158724634					0																	No-ASE
OR6N1	1	158735595		1		1	0	1			1	1		1	1	1	1	0						ASE
IFI16	1	158979950					0							0			0					0		No-ASE
DARC	1	159174683		0	0																			No-ASE
OR10J3	1	159283467												1										ASE
OR10J1	1	159409612					0																	No-ASE
OR10J5	1	159504942	1	0			1		0				1											Heterogeneous_ASE
DUSP23	1	159751104				0	1	0				0		1										Heterogeneous_ASE
FCRL6	1	159778115		0															1					Heterogeneous_ASE
SLAMF8	1	159796689					0																	No-ASE
C1orf204	1	159810633				0								0										No-ASE
CCDC19	1	159842304					0		0			0									0			No-ASE
IGSF9	1	159897251	0							0		0								0				No-ASE
SLAMF9	1	159921606	0									0								0				No-ASE
PIGM	1	160000380						0						0								0		No-ASE
KCNJ10	1	160011262												0										No-ASE
ATP1A2	1	160090708	1																					ASE
ATP1A4	1	160121869					0																	No-ASE
CASQ1	1	160160671	0																					No-ASE
SLAMF7	1	160709111																				0		No-ASE
LY9	1	160769567																0						No-ASE
ITLN2	1	160914970													0			0						No-ASE
F11R	1	160968609					0						0				0	0	0			0		No-ASE
USF1	1	161009523					0						0				0	0				0		No-ASE
ARHGAP30	1	161017761		0	0		0				0					0						0		No-ASE
PVRL4	1	161042515				0				0							0	0					0	No-ASE
KLHDC9	1	161068365	0	0			0	0		0			0			0	0	0		0			0	No-ASE
PPOX	1	161136705		0							0						0			0				No-ASE
ADAMTS4	1	161160956					0											0						No-ASE
NDUFS2	1	161172233		0					0		0						0			0				No-ASE
FCGR2A	1	161476205												0								1		Heterogeneous_ASE
FCRLA	1	161677004																				0		No-ASE
FCRLB	1	161692468	0			0						0						0	0					No-ASE
ATF6	1	161736167		0		0	0	0		0	0	0	0				0			0		0		No-ASE
OLFML2B	1	161953624										0							0					No-ASE
SH2D1B	1	162367103		0						0								0	1					No-ASE
DDR2	1	162724415				0																0		No-ASE
C1orf110	1	162824562								0					0	0								No-ASE
RGS5	1	163115321		0								0				0						0		No-ASE
NUF2	1	163298037					0							0						0	0		0	No-ASE
PBX1	1	164529120		0		0		0		0	0	0		0			0			0			0	No-ASE
ALDH9A1	1	165632360			0						0	0												No-ASE
POGK	1	166810206						0																No-ASE
TADA1	1	166826838										0												No-ASE
ILDR2	1	166890063								1						0	0						0	No-ASE
MAEL	1	166958710		0		0		1							0									No-ASE
GPA33	1	167023590				0		0						0										No-ASE
DUSP27	1	167064097		0	1		0	0	0		1		1		0			0		0	0		1	Heterogeneous_ASE
ADCY10	1	167778926					0					0					0	0						No-ASE
DCAF6	1	167935880				0					0			0					0				0	No-ASE
BLZF1	1	169345868				0							0											No-ASE
C1orf114	1	169366494								1				0			0	0						No-ASE
F5	1	169483561				0	0			0	0		0		1		0						0	No-ASE
SELP	1	169559396			0		0				0	0												No-ASE
SELL	1	169660939												0										No-ASE

METTL18	1	169761758					0								0			0		0				No-ASE
C1orf112	1	169770059	0						0	0	0				0									No-ASE
SCYL3	1	169823521	0				0		0	0	0			0	0		0	0						No-ASE
KIFAP3	1	169890903	0				0			0	0	0		0			0			0				No-ASE
METTL11B	1	170115300					0			1								0						Heterogeneous_ASE
GORAB	1	170508497	0				0		0	0		0	0			0	0	0						No-ASE
MROH9	1	170927615					0																	No-ASE
FMO3	1	171072965	0								0													No-ASE
FMO2	1	171162516					0		1					1	0		1	0						Heterogeneous_ASE
PRRC2C	1	171486912		0	0	0	0	0		0	0	0		0										No-ASE
METTL13	1	171753039	0	0	0	0		0			0	0	0	0			0	0	0	0	0			No-ASE
C1orf105	1	172410967		0	0		0			0	0									0			0	No-ASE
ZBTB37	1	173839374						0																No-ASE
RC3H1	1	173912593															0							No-ASE
TNN	1	175046557					0	0								0								No-ASE
TNR	1	175292499	0			0								0			0	0						No-ASE
RFWD2	1	176012946																					0	No-ASE
PAPPA2	1	176525585													1									ASE
FAM5B	1	177199021					0																	No-ASE
SEC16B	1	177899030	0	0	0	0	0	0			0	0		0	0	0		0	0	0	0			No-ASE
TEX35	1	178483205	1			0							0		1									Heterogeneous_ASE
C1orf220	1	178518008				0			0					0						0				No-ASE
RALGPS2	1	178777231											0											No-ASE
TOR3A	1	179051300		0											0								0	No-ASE
ABL2	1	179077109				0	0													0				No-ASE
SOAT1	1	179271826						0																No-ASE
AXDND1	1	179338046			0		1			0														Heterogeneous_ASE
TDRD5	1	179561901	1		0		0	0		0	0		0	0	0			0			0			No-ASE
TOR1AIP1	1	179851642	0	0	0				0		0	0	0		0	0	0	0		0			0	No-ASE
CEP350	1	179959724					0				0		0	0	0					0				No-ASE
QSOX1	1	180124061				0				0				0	0	0	0							No-ASE
LHX4	1	180199701	0		0				0	0	0	0		0		0	0	0						No-ASE
KIAA1614	1	180885290		0			0				0	0											0	No-ASE
CACNA1E	1	181701810									0							0						No-ASE
ZNF648	1	182025483					0	0			0	0												No-ASE
TEDDM1	1	182369039	0		0	0		0	0	0	0			0			0	0		0				No-ASE
RGSL1	1	182423094					0				1													Heterogeneous_ASE
RNASEL	1	182544687		0	0	0	0	0	0		0	0	0			0	0	0			0		0	No-ASE
RGS16	1	182569501								0				0				0					0	No-ASE
LAMC1	1	182993159	0	0		0	0	0	0	0		0			0	0				0				No-ASE
LAMC2	1	183177132			0	0	0			0				0	0	0			0	0				No-ASE
SMG7	1	183485010		0				0				0		0	0		0							No-ASE
NCF2	1	183525282	0		0	0	0			1	0		1	0	0	0	0	0		0				No-ASE
RGL1	1	183616866	0	1	0	0		1	0	0	0	0		0	0			0	0				0	No-ASE
TSEN15	1	184020945			0		0	0	0			0	0	0		0	0	0						No-ASE
EDEM3	1	184663288	0			0	0						0	0			0	0		0			0	No-ASE
FAM129A	1	184764167						0																No-ASE
RNF2	1	185069416					0																	No-ASE
SWT1	1	185130019		0	0		0					0			0	0	0			0			0	No-ASE
HMCN1	1	185703987		1	0	0					0	0		0		0	1	0	1	0			1	Heterogeneous_ASE
MIR548F1	1	186266014	0	0	0			0	0		0	0	0		0		0			0				No-ASE
PLA2G4A	1	186863287									0													No-ASE
RGS13	1	192617063		0																				No-ASE
GLRX2	1	193065799										0												No-ASE
CFH	1	196621252		1						0						0	0	0					1	Heterogeneous_ASE
ASPM	1	197056003			0	0	0				0		0	0			0		0	0				No-ASE
ZBTB41	1	197126649				0		0			0	0				0		0	0					No-ASE
DENND1B	1	197479723	0							0		0												No-ASE
KIF14	1	200522566	0	0	0		0	0		0		0	0				0	0		0	0		0	No-ASE
DDX59	1	200613555										0												No-ASE
CAMSAP2	1	200709059					0																	No-ASE
C1orf106	1	200867482																					0	No-ASE
KIF21B	1	200943291							0	0				0			0				0		0	No-ASE
CACNA1S	1	201009011			1	0							1	0		1								Heterogeneous_ASE
IGFN1	1	201163290	0	1	0	1	0	0		0	0			0	0			0		0			0	No-ASE
PKP1	1	201252984								0						1	0	0		0				No-ASE
LAD1	1	201351385	0	0		0		0	0			0		0	0	0	0	0	0	0				No-ASE
NAV1	1	201618040	0											0		0								No-ASE
SHISA4	1	201858713		0		1		0	0	0	0	0			0					0				No-ASE
LMOD1	1	201868510													0									No-ASE
TIMM17A	1	201926422			0						0	0												No-ASE
RNPEP	1	201958607				0		0						0	0	0	0		0					No-ASE
GPR37L1	1	202092293			0																			No-ASE
PTPN7	1	202119482								1													1	ASE
LGR6	1	202172988	0		0	0							1							0			0	No-ASE
PPP1R12B	1	202318143								0								0					1	Heterogeneous_ASE
KDM5B	1	202698838											0										0	No-ASE
CYB5R1	1	202932203									0													No-ASE
MYOG	1	203053787																0						No-ASE
MYBPH	1	203137795																0						No-ASE
CHI3L1	1	203148620									0							0					1	Heterogeneous_ASE
CHIT1	1	203186067																0						No-ASE
PRELP	1	203452341						0										0						No-ASE
ATP2B4	1	203652338																		0				No-ASE
LAX1	1	203734770					0							1				0						Heterogeneous_ASE
ZC3H11A	1	203766895		0		0	0		0	0		0		0		0	0	0					0	No-ASE
SOX13	1	204082173																0						No-ASE
ETNK2	1	204106316									0	0	0		0	0		0						No-ASE
KISS1	1	204159701																0						No-ASE
PLEKHA6	1	204197220	0							0	0		0		0	0			0	0	0		0	No-ASE
PPP1R15B	1	204378654	0					0		0	0		0				0							No-ASE
PIK3C2B	1	204394023													0						0			No-ASE
LRRN2	1	204587044	0				0		1					0			0						0	No-ASE
NFASC	1	204923406	0						0						0						0		0	No-ASE
CNTN2	1	205027156	0		0	0	0		0			0		0				0		0			0	No-ASE
TMEM81	1	205052685	0	0		0		0				0			0									No-ASE
DSTYK	1	205116700		0		0																		No-ASE
NUAK2	1	205272732																		0				No-ASE
LEMD1-AS1	1	205353492											0	0										No-ASE
CDK18	1	205492387															0							No-ASE
MFSD4	1	205548956											0	0										No-ASE
RAB7L1	1	205739558				0																		No-ASE
PM20D1	1	205797858	0	1	1		0	0		1	1	1	0	0	0	0	1	0	0	1			1	Heterogeneous_ASE
AVPR1B	1	206224453											0											No-ASE
SRGAP2	1	206603535				0	0	0	0	0	0	0		0	0		0	0	0	0	0		0	No-ASE
IKBKE	1	206647800		0				0					0											No-ASE
DYRK3	1	206811017													0									No-ASE
IL19	1	206972307					0								0			0						No-ASE
IL24	1	207071206		0		0	0							0		0			0					No-ASE
FAIM3	1	207078372			0												0							No-ASE
PIGR	1	207105843		0		1	0		0						0		1		1	0				Heterogeneous_ASE
FCAMR	1	207131878				0																	0	No-ASE
C1orf116	1	207195352											0											No-ASE
C4BPA	1	207286380					0																	No-ASE
CR2	1	207639979			0	1					0					0							1	Heterogeneous_ASE
CR1	1	207679398					0		1			1					1	1						ASE
CR1L	1	207850743					0																	No-ASE
PLXNA2	1	208200640	0		0	0	0	0	0	0	0	0	0	0	0	0	0	0	0				0	No-ASE

CAMK1G	1	209768356					0			0			1										Heterogeneous_ASE
LAMB3	1	209788656	0		0		0		0				0			0					0			No-ASE
TRAF3IP3	1	209933400	0											0				0						No-ASE
C1orf74	1	209956402					0	0					0				0							No-ASE
DIEXF	1	210001460	0				0	0					0	0			0	0						No-ASE
SERTAD4	1	210411339																					0	No-ASE
HHAT	1	210522387	0			0	0	0	0	0					0	0	0	0						No-ASE
NEK2	1	211836799					0						0											No-ASE
DTL	1	212209243								0					0									No-ASE
TMEM206	1	212553310		0																				No-ASE
NENF	1	212617692			0										0	0	0	0		0			0	No-ASE
FAM71A	1	212798251					0																	No-ASE
NSL1	1	212911779	0					0	0	0	0	0					0	0		0			0	No-ASE
FLVCR1	1	213032371	0					0	0		0		0				0		0				0	No-ASE
RPS6KC1	1	213244366				0																		No-ASE
SMYD2	1	214488172				0										0								No-ASE
PTPN14	1	214531313					0			0	0	0												No-ASE
CENPF	1	214791951		0		0	0	0	0			0	0	0	0	0		0	0	0				No-ASE
USH2A	1	215799170					0					1		0			0	1	0					Heterogeneous_ASE
GPATCH2	1	217604492		0								0												No-ASE
SPATA17	1	217804722						0				0												No-ASE
RRP15	1	218458672					0			0													0	No-ASE
RNU5F-1	1	220088801		0	0			0	0		0			0		0				0				No-ASE
IARS2	1	220298664		0																				No-ASE
RAB3GAP2	1	220324619															0							No-ASE
mrt-01	1	220964843							0					0			0			0			0	No-ASE
HLX	1	221053574					0			1		1		0						0				Heterogeneous_ASE
HHIPL2	1	222696004				0	0		0			0								1	0		1	Heterogeneous_ASE
TAF1A	1	222732027										0												No-ASE
MIA3	1	222800921	0												0		0							No-ASE
FAM177B	1	222919895	0				0										0							No-ASE
DISP1	1	223116452	0	0		1	0								0	0	0						0	No-ASE
TLR5	1	223283807	0			0	0	0		0			0	0	0	0		0		0				No-ASE
CAPN8	1	223716508		0						0	0	0				0	0			0				No-ASE
CAPN2	1	223900408						0	0		0							0						No-ASE
TP53BP2	1	223971861				0		0				0					0	0	0					No-ASE
NVL	1	224415342					0							0				0						No-ASE
DNAH14	1	225140459	0	0		0	0	0			0	0	0	0										No-ASE
LBR	1	225592184									0			0						0				No-ASE
EPHX1	1	226016551	0	0								0	0	0				0	0					No-ASE
TMEM63A	1	226034787	0			0				0	0	0		0	0					0				No-ASE
LEFTY1	1	226074563				0														1			0	Heterogeneous_ASE
ACBD3	1	226340046		0	0			0			0	0	0	1	0	0		0	0					No-ASE
PARP1	1	226550829	0	0		0				0	0								0				0	No-ASE
ITPKB	1	226822380		0	0		0	0	0	0	0	0	0	0	0	0		0		0			0	No-ASE
CDC42BPA	1	227178948	0		0	0		0			0			0	0	0				0	0		0	No-ASE
ZNF678	1	227842385													0									No-ASE
LOC100130093	1	227920196										0												No-ASE
SNAP47	1	227923195	0	0			0		0	0		0		0		0		0	0	0				No-ASE
IBA57	1	228353528	0	0		0				0	0			0				0		0			0	No-ASE
OBSCN	1	228401940	0	0	0	0	0	0	0	0	0	0	0	0	0	0	0	0	1	0	0		0	No-ASE
CCSAP	1	229462604					0																	No-ASE
NUP133	1	229577717		0										0	0		0		0	0				No-ASE
ABCB10	1	229654053							0															No-ASE
URB2	1	229771013		0		0		0		0		0		0	0	0					0		0	No-ASE
GALNT2	1	230314016			0						0		0											No-ASE
COG2	1	230795320								0														No-ASE
AGT	1	230838903	0				0				0					1			1					Heterogeneous_ASE
CAPN9	1	230883283	1		1	1	0	1	0	0		0		1				1		0			1	Heterogeneous_ASE
C1orf131	1	231362795																					0	No-ASE
GNPAT	1	231377150	0	0	0			0		0	0		0											No-ASE
EXOC8	1	231471540						0						0										No-ASE
SPRTN	1	231474181	0			0		0		0	0							0					0	No-ASE
TSNAX-DISC1	1	231665015	0	0	0	0	0			0	0	0	0	0	0			0	0	0				No-ASE
SIPA1L2	1	232534919		0		0	0	0		0	0	0		0	0	0	0	0		0	0			No-ASE
PCNXL2	1	233120167	0	0	0	0			0	0	0	0	0		0	0	0			0			0	No-ASE
KIAA1804	1	233464070		0		0	0	0							0	0		0	0	0				No-ASE
KCNK1	1	233750008													0									No-ASE
TARBP1	1	234529406		0	0	0	0	0	0		0	0	0		0			0	0		0			No-ASE
RBM34	1	235295240						0		0	0	0			0									No-ASE
ARID4B	1	235338517	0					0		0	0	0		0	0								0	No-ASE
NID1	1	236141174	0	1		1	0				1	1						0						Heterogeneous_ASE
ERO1LB	1	236381824	0					0	0	0				0	0					0				No-ASE
EDARADD	1	236557766														1		1						ASE
LGALS8	1	236700807		0	0						0		0	0				0						No-ASE
HEATR1	1	236716888		0	0		0			0	0		0	0		0		0	0				0	No-ASE
ACTN2	1	236849999					0																	No-ASE
MTR	1	236959016	0			0						0						0						No-ASE
RYR2	1	237550653						0						0										No-ASE
FMN2	1	240255940														0								No-ASE
KMO	1	241695758			0						0				0		1	1					1	Heterogeneous_ASE
EXO1	1	242012033	0	0	0	0	0	0		0		0	0	0	0	0	0	0	0	0			0	No-ASE
ADSS	1	244574657																		0				No-ASE
C1orf101	1	244627212													0	0	0							No-ASE
DESI2	1	244869045					0																	No-ASE
COX20	1	245005544	0																					No-ASE
EFCAB2	1	245180555		0																				No-ASE
SMYD3	1	245912881	0		0			0					1											No-ASE
CNST	1	246730090			0				0															No-ASE
AHCTF1	1	247006035		0				0				0	0										1	No-ASE
ZNF695	1	247150582	0							0	0			0	0		0	0					0	No-ASE
VN1R5	1	247419932																0						No-ASE
OR2B11	1	247614386			1	1	0	1				1	1					0	1					ASE
OR2C3	1	247694898					0											0						No-ASE
OR2G2	1	247751731					0																	No-ASE
OR2G3	1	247768948																0						No-ASE
OR13G1	1	247835493			1			1			1	1												ASE
OR6F1	1	247875276										1							1					ASE
TRIM58	1	248028032				0	0		1					0	1			0						Heterogeneous_ASE
OR2W3	1	248058983			0		0	1	1			0		0							0		0	No-ASE
OR2L13	1	248128722					0							0										No-ASE
OR2T12	1	248458760				0																		No-ASE
OR14C36	1	248512255					0																	No-ASE
OR2T6	1	248550916					0																	No-ASE
OR2T1	1	248569369					0																	No-ASE
OR2G6	1	248684976			0		0																	No-ASE
OR2T11	1	248789753	0																					No-ASE
OR14I1	1	248844695				1	0			0	0													No-ASE
FAM110C	2	38938						0				0												No-ASE
SH3YL1	2	218992		1		1		1													1	0		ASE
ACP1	2	264985		0		0		0								0					0	0		No-ASE
SNTG2	2	1079280		0							1	1						0				1		Heterogeneous_ASE
TPO	2	1418188	0					0					0					0		0				No-ASE
TRAPPC12	2	3391458	0	0		0			0		0		0							0	0	0	0	No-ASE
RNASEH1	2	3596275	0																0				0	No-ASE
ALLC	2	3726083																0					1	Heterogeneous_ASE
RNF144A	2	7137067							0									0						No-ASE
KIDINS220	2	8871219				0									0			0	0		0			No-ASE

ASAP2	2	9496439	0			0					0	0	0			0		0	0		0		0	No-ASE
ADAM17	2	9630344											0										0	No-ASE
TAF1B	2	10015994	0	0				0	0			0			0	0			0	0		0	0	No-ASE
GRHL1	2	10098921	0	0				0			0	0				0			0		0	1	0	No-ASE
RRM2	2	10262920	0	0		0									0			0		0				No-ASE
NOL10	2	10717775	0						0			0	0			0					0			No-ASE
ATP6V1C2	2	10863065	0	0		0		0	0				0		0	0				0		0		No-ASE
ROCK2	2	11323276	0	0		0		0	0		0	0	0		0			0				0	0	No-ASE
GREB1	2	11702661	0	0		0		0	0		0	0	0		0			0			0	0	0	No-ASE
LPIN1	2	11905710				0			0															No-ASE
TRIB2	2	12858444				0																		No-ASE
NBAS	2	15307240				0			0				0		0			0			0			No-ASE
RAD51AP2	2	17696573	0					0				0			0			0			0			No-ASE
SMC6	2	17846775		0				0	0			0			0					0		0	0	No-ASE
GEN1	2	17941352	0	0				0	0			0			0					0	0	0	0	No-ASE
KCNS3	2	18112301	0									1						0	0	1			0	Heterogeneous_ASE
NT5C1B-RDH14	2	18736630				0																		No-ASE
WDR35	2	20113444	0						0				0		0	0			0		0		0	No-ASE
MATN3	2	20197017		0																				No-ASE
HS1BP3	2	20818764	0			0		0				0	0		0			0		0	0			No-ASE
C2orf43	2	20939928	0																					No-ASE
APOB	2	21224624	0																					No-ASE
ATAD2B	2	23977086							0															No-ASE
C2orf44	2	24253941		0									0							0				No-ASE
TP53I3	2	24300517		0									0							0				No-ASE
FAM228B	2	24358001	0	1																				Heterogeneous_ASE
ITSN2	2	24426578							0		0		0	0	0			0	0	0			0	No-ASE
CENPO	2	25016350									0													No-ASE
ADCY3	2	25045383	0	0		0		0	0		0	0	0		0	0		0		0	0	0		No-ASE
EFR3B	2	25354712	0			0					0		1							0	0			No-ASE
ASXL2	2	25965095										0												No-ASE
KIF3C	2	26151206						0					0					0						No-ASE
GAREML	2	26399214	0	0		0		0	0							0		0		0	0	0		No-ASE
GPR113	2	26532442	0	1				1			0		1			0				0				Heterogeneous_ASE
CCDC164	2	26624955									0	0				0						0		No-ASE
OTOF	2	26682949		1														0				1		Heterogeneous_ASE
C2orf70	2	26798771	0	0									0		0			0	0		0			No-ASE
CIB4	2	26804247						0	0			0	1		0									No-ASE
DPYSL5	2	27150268														1								ASE
TMEM214	2	27258096	0					0							0								0	No-ASE
EMILIN1	2	27302085		0																				No-ASE
KHK	2	27315223	0			0		0			0				0				0	0			0	No-ASE
ABHD1	2	27346844							0															No-ASE
SLC5A6	2	27423371	0						0						0	0		0	0		0		0	No-ASE
ATRAID	2	27435250	0																					No-ASE
CAD	2	27444110	0																					No-ASE
DNAJC5G	2	27499607				0																		No-ASE
GTF3C2	2	27549562	0	0		0		0	0						0			0			0		0	No-ASE
EIF2B4	2	27587440				0															0			No-ASE
IFT172	2	27671847		0									0											No-ASE
GCKR	2	27720177				0									0					0				No-ASE
C2orf16	2	27799465	0	0		0									0								0	No-ASE
GPN1	2	27851918	0												0									No-ASE
SLC4A1AP	2	27886669		0		0									0							0	0	No-ASE
PLB1	2	28718991	0	0		1					0	0	0		0	0		0	0	0	1		0	No-ASE
FAM179A	2	29222070	0												1	0		0				0	1	Heterogeneous_ASE
C2orf71	2	29287776	0																					No-ASE
CLIP4	2	29344334	0																			0		No-ASE
ALK	2	29416157							0						0	0								No-ASE
LCLAT1	2	30748587				0													0			0		No-ASE
CAPN13	2	30957326				0		0			0		1									0	0	No-ASE
GALNT14	2	31135184	1								1				1			0						ASE
CAPN14	2	31397570													1									ASE
XDH	2	31560521	0		1																			Heterogeneous_ASE
SPAST	2	32289031																				0	0	No-ASE
BIRC6	2	32613882	0	0		0			0		0				0	0		0		0				No-ASE
TTC27	2	32855677	0										0									0		No-ASE
LTBP1	2	33172816		1									0							0				Heterogeneous_ASE
FAM98A	2	33809851				0																		No-ASE
FEZ2	2	36782886	0			0		0	0			0							0		0	0	0	No-ASE
VIT	2	36970296						0																No-ASE
STRN	2	37078233	0					0															0	No-ASE
HEATR5B	2	37215860									0											0	0	No-ASE
CEBPZ	2	37439091		0									0											No-ASE
PRKD3	2	37480347										0												No-ASE
QPCT	2	37579971						1																ASE
CYP1B1	2	38298139	0	0		0						0	0		0	0		0	0			0	1	No-ASE
ATL2	2	38525489	0	0											0			0	0		0	0	0	No-ASE
GALM	2	38893338				0																		No-ASE
GEMIN6	2	39008790				0																		No-ASE
DHX57	2	39025499	0					1				0	0							0			0	No-ASE
MORN2	2	39109558		0								0												No-ASE
SLC8A1-AS1	2	40342461				0																		No-ASE
PKDCC	2	42275914									0				0						0			No-ASE
EML4	2	42472672	0	0		0						0	0						0					No-ASE
OXER1	2	42990088	0	0											0			0		1			0	No-ASE
HAAO	2	42994581										1	0											Heterogeneous_ASE
ZFP36L2	2	43451498				0																		No-ASE
THADA	2	43458176						0	0				0		0			0	0	0	0			No-ASE
PLEKHH2	2	43871897																	1					ASE
DYNC2LI1	2	44004010	0			0			0		0				0	0			0	0			0	No-ASE
ABCG5	2	44040341									0													No-ASE
ABCG8	2	44066247	0								0	0			0				1	0			0	No-ASE
SLC3A1	2	44502696				0		0					0		0	0		0	0	0	0		0	No-ASE
SRBD1	2	45616486				0									0				0					No-ASE
PRKCE	2	46234762																	0					No-ASE
EPAS1	2	46574026									0										0			No-ASE
TTC7A	2	47177506		0																			1	Heterogeneous_ASE
C2orf61	2	47314198		0				0				0												No-ASE
EPCAM	2	47596694						0		0		0						0						No-ASE
MSH2	2	47630353									0							0						No-ASE
STON1-GTF2A1L	2	48807796	0			1					0	0			0			0		0	0		0	No-ASE
ASB3	2	53897765								0		0	0											No-ASE
GPR75-ASB3	2	54023086											0											No-ASE
PSME4	2	54093901	0	0				0		0		0			0	0							0	No-ASE
ACYP2	2	54342846	0	0					0	0	0													No-ASE
C2orf73	2	54558114	1						1	1		0				0		0						Heterogeneous_ASE
RTN4	2	55209766		0		0																		No-ASE
CLHC1	2	55403011	0	0		0		0								0		0	1		0			No-ASE
MTIF2	2	55463798	0	0	1	0							0						0					No-ASE
CCDC104	2	55746955																0						No-ASE
SMEK2	2	55777060				0																		No-ASE
CCDC85A	2	56420006																0						No-ASE
VRK2	2	58276070	0							0	0	0				0	0				0			No-ASE
BCL11A	2	60687959											0				0							No-ASE
PEX13	2	61258602							0				0											No-ASE
FAM161A	2	62053619		0				0	0				0			0	0		0				1	No-ASE
COMMD1	2	62132847						0			0													No-ASE

TMEM17	2	62728379	0			0				0	0					0	0						0	No-ASE
EHBP1	2	62998484								0							0							No-ASE
VPS54	2	64124722						0					0								0		0	No-ASE
PELI1	2	64323660	0			0		0	0	0		0	0				0		0		0		0	No-ASE
AFTPH	2	64778661		0															0					No-ASE
SLC1A4	2	65216817		0		0		0					0				0	0						No-ASE
CEP68	2	65296657	0	0		0		0	0	0		0	0		0	0	0	0					0	No-ASE
ACTR2	2	65496018				0		0				0												No-ASE
ETAA1	2	67624665		0		0		0		0		0	0		0	0	0			0				No-ASE
WDR92	2	68358474				0		0				0					0						0	No-ASE
PNO1	2	68385097	0	0		0		0		0		0	0		0		0			0	0		0	No-ASE
PLEK	2	68592496				1									0	1								Heterogeneous_ASE
APLF	2	68717322				0					0						0		0					No-ASE
PROKR1	2	68873059				0																		No-ASE
ARHGAP25	2	69015005	1	1				0		1					1		1		0	0			0	Heterogeneous_ASE
GFPT1	2	69555414									0		0											No-ASE
NFU1	2	69659049	0	0		0			0			0			0	0								No-ASE
ANXA4	2	70015272							0		0				0	0	0							No-ASE
ASPRV1	2	70187808				0					0	0					0	0	0				0	No-ASE
PCYOX1	2	70488470						0											0					No-ASE
FAM136A	2	70524142		0		0			0		0	0			0				0					No-ASE
CLEC4F	2	71036417						1										0						Heterogeneous_ASE
CD207	2	71058230	0	0				1				0						0	1					Heterogeneous_ASE
ATP6V1B1	2	71163086	0	1				1				0						0						Heterogeneous_ASE
ANKRD53	2	71205795	0	0		0			0															No-ASE
NAGK	2	71297695	0			0		0		0	0					0	0	0	0	0	0			No-ASE
MCEE	2	71337111		0				0		0	0					0								No-ASE
MPHOSPH10	2	71357884		0					0		0					0		0						No-ASE
PAIP2B	2	71415479						0																No-ASE
ZNF638	2	71576163				0		0			0				0		0	0	0		0			No-ASE
DYSF	2	71709066				0		0						0										No-ASE
CYP26B1	2	72359386		0																			0	No-ASE
EXOC6B	2	72406471								0		0						0	0		0			No-ASE
ALMS1	2	73613030	0			0			0	0			0		0			0	0	0			0	No-ASE
NAT8B	2	73927766	0			0				0			0		0		0	0					0	No-ASE
DUSP11	2	73989915								0			0					0		0			0	No-ASE
TET3	2	74273472							0															No-ASE
SLC4A5	2	74450058	0			0			0		0	0					0	0						No-ASE
C2orf81	2	74641512										0												No-ASE
MOGS	2	74688553		0					0								0		0	0				No-ASE
MRPL53	2	74699518		0													0			0				No-ASE
TTC31	2	74710469		0													0		0	0				No-ASE
PCGF1	2	74732316				0																		No-ASE
HK2	2	75099477													0		0							No-ASE
POLE4	2	75196731		0								0				0			0	0	0			No-ASE
TACR1	2	75276688										0												No-ASE
MRPL19	2	75874015											0		0									No-ASE
GCFC2	2	75891858			0	0		0	0	0		0	0	0	0		0	0						No-ASE
REG1B	2	79312725																					1	ASE
CTNNA2	2	80101372											1			0								Heterogeneous_ASE
DNAH6	2	84745113	0	0				0			0		0						0		0	1		No-ASE
TRABD2A	2	85049153		0																				No-ASE
TGOLN2	2	85549527	0	0		0		0		0	0		0	0	0	0				0	0	0	0	No-ASE
ELMOD3	2	85589319		0								0			0							0		No-ASE
CAPG	2	85622059				0					0	0			0						0	0	1	No-ASE
MAT2A	2	85766442	0	0					0	0	0		0							0		0	0	No-ASE
GGCX	2	85777080				0			0	0	0							0			0	0	0	No-ASE
RNF181	2	85823724							0												0		0	No-ASE
SFTPB	2	85890543										0						0				0		No-ASE
GNLY	2	85921546										0		0								0		No-ASE
ST3GAL5	2	86067277								0		0			1		0							No-ASE
POLR1A	2	86255790							0		0													No-ASE
PTCD3	2	86333420									0													No-ASE
MRPL35	2	86426629		0				0	0			0	0		0			0				0		No-ASE
KDM3A	2	86676932	0			0		0	0						0		0					0		No-ASE
KRCC1	2	88327431																	0					No-ASE
FABP1	2	88424066							0				0											No-ASE
THNSL2	2	88472737				0		1								1					0		1	Heterogeneous_ASE
EIF2AK3	2	88857275						0	0	0						0	0					0		No-ASE
LOC442028	2	95537418										1							1					ASE
MRPS5	2	95753139													0									No-ASE
PROM2	2	95940475						0							0	0				0		0	0	No-ASE
KCNIP3	2	95976119																	0					No-ASE
GPAT2	2	96689154				0												0						No-ASE
ASTL	2	96789747				0		0	0			0			0		0	0				0	0	No-ASE
STARD7	2	96852984																	0	0	0			No-ASE
SNRNP200	2	96940866	0	0		0		0			0								0	0			1	No-ASE
ITPRIPL1	2	96992520	0	1		1					0								0	0			0	Heterogeneous_ASE
NCAPH	2	97007416		0		0					0			0									0	No-ASE
FER1L5	2	97313565	0															0		0				No-ASE
SEMA4C	2	97526521																0						No-ASE
FAM178B	2	97543662																0						No-ASE
ACTR1B	2	98273912				0			0	0				0				0	0				0	No-ASE
LOC728537	2	98317685						0	0	0	0	0						0				0	0	No-ASE
TMEM131	2	98373584							0															No-ASE
VWA3B	2	98709673								0							0					0		No-ASE
INPP4A	2	99149864				0		0	0	0				0								0		No-ASE
MGAT4A	2	99239931								0														No-ASE
MRPL30	2	99802676	0			0				0							0							No-ASE
EIF5B	2	99977004				1			0			0		0		0						0		No-ASE
REV1	2	100019256	0	0		0		0	0	0		0	0		0	0	0	0	0	0	0	0		No-ASE
AFF3	2	100167950																1						ASE
LONRF2	2	100900836		1																				ASE
NPAS2	2	101541736		0		0			0							0	0	0				0	0	No-ASE
RPL31	2	101622522	0	0		0		0	0	0	0							0		0	0		0	No-ASE
TBC1D8	2	101638744	0			0		0		0	0							0	0	0	0		0	No-ASE
RNF149	2	101898412												0	0	0								No-ASE
MAP4K4	2	102440413	0							0	0		0	0		0			0				0	No-ASE
IL1R1	2	102781253		0					0			0			0									No-ASE
IL1RL2	2	102804330		1									1							0				Heterogeneous_ASE
IL1RL1	2	102954743																				0		No-ASE
SLC9A4	2	103090237						0			0			0	0					1	0			No-ASE
MFSD9	2	103334920															0	0				0		No-ASE
MRPS9	2	105654555	0	0		0			0	0				0			0			0	0	0		No-ASE
GPR45	2	105858434							0	0				0		0							1	No-ASE
TGFBRAP1	2	105885934						0		0	0		0					0		0			0	No-ASE
C2orf49	2	105954054		0														0				0		No-ASE
ST6GAL2	2	107446600	0						1									0			1			Heterogeneous_ASE
SULT1C3	2	108863652											0				0	0					0	No-ASE
SULT1C2	2	108910170																0				0		No-ASE
SULT1C4	2	108994808									0							0						No-ASE
GCC2	2	109067508										0												No-ASE
CCDC138	2	109403311								0									0					No-ASE
EDAR	2	109513401																					1	ASE
NPHP1	2	110881489				0													0					No-ASE
ACOXL	2	111542370						0				0										0		No-ASE
MERTK	2	112686742	0	0					0	0	0				0	0	0	0	0		0	0	0	No-ASE
ZC3H6	2	113067622											0											No-ASE

POLR1B	2	113300078						0		0		0		0			0	0	0	0	0		0	No-ASE
SLC20A1	2	113404460							0															No-ASE
CKAP2L	2	113496452	0			0		0	0	0		0			0	0	0		0			0		No-ASE
IL1A	2	113535640	0			0		0	0			0	0			0		0		0				No-ASE
IL37	2	113670627		0		0			0		0	0						0				0		No-ASE
IL36A	2	113763575														0		1						Heterogeneous_ASE
IL36RN	2	113818479																				0		No-ASE
IL1F10	2	113830347		1													1	1						ASE
IL1RN	2	113888688		1				1	0					0				0	0					Heterogeneous_ASE
PSD4	2	113940062		0							0	0			0	0		0		0	0	0	0	No-ASE
PAX8	2	113977678				0																1		Heterogeneous_ASE
DPP10	2	115822349				1																		ASE
DDX18	2	118575080		0		0		0						0	0	0		0			0			No-ASE
CCDC93	2	118693086										0	0	0										No-ASE
INSIG2	2	118864736							0			0						0						No-ASE
STEAP3	2	120003085						0			0	0		0									0	No-ASE
C2orf76	2	120060082		0						0		0						0		0		0	0	No-ASE
DBI	2	120125058		0		0		0					0	0				0		0		0		No-ASE
TMEM177	2	120438515										0	0						0					No-ASE
PTPN4	2	120567482		0				0						0										No-ASE
EPB41L5	2	120776731		0				0	0	0								0			0		0	No-ASE
TMEM185B	2	120980428	0					0		0	0				0					0			0	No-ASE
GLI2	2	121555015		0						1		0		0									0	No-ASE
CLASP1	2	122125187				0																		No-ASE
CNTNAP5	2	125175089											1									1		ASE
BIN1	2	127806143	0			0		0		0		0	0	0		0								No-ASE
MYO7B	2	128321770	0	0		0		0		0	0	0			0			1			0	0		No-ASE
WDR33	2	128467314						0		0	0	0				0			0					No-ASE
SAP130	2	128707965				0																		No-ASE
IMP4	2	131102232																	0					No-ASE
ARHGEF4	2	131688595		0										0										No-ASE
RNU6-81	2	132235740										0												No-ASE
GPR39	2	133174742		0							0		0	0					0		0		0	No-ASE
NCKAP5	2	133483265				0		1					0		0	0		0						No-ASE
RAB3GAP1	2	135810049				0			0	0		0			0						0			No-ASE
ZRANB3	2	135965204																			0			No-ASE
R3HDM1	2	136374311							0			0												No-ASE
LCT	2	136545910	0						1			1		0										Heterogeneous_ASE
LRP1B	2	140990868										0												No-ASE
GTDC1	2	144704655	0	0		0		0			0	0	0	0					0		0	0		No-ASE
ORC4	2	148695747								0		0		0		0		0					0	No-ASE
MBD5	2	149221327								0														No-ASE
RBM43	2	152107491								0														No-ASE
NMI	2	152127212										0	0				0					0	0	No-ASE
RIF1	2	152266992	0			0					0	0	0	0	0				0		0	0	0	No-ASE
NEB	2	152342288	0			0		0	0	0	0	0	0	0	0	1	0	0			0	0	0	No-ASE
CACNB4	2	152727089																0						No-ASE
FMNL2	2	153192170								0									0					No-ASE
GPD2	2	157352676			1													0						Heterogeneous_ASE
GALNT5	2	158114759		0				0	0	0			0	0			0							No-ASE
ACVR1C	2	158386938															0							No-ASE
ACVR1	2	158637039																				0		No-ASE
CCDC148	2	159166069																	0			0		No-ASE
DAPL1	2	159660805				0				0	0		1				0	0			0	0	0	No-ASE
TANC1	2	159954175	0	0					0	0	0		0	0	1	0	0		0	0	0	0	0	No-ASE
WDSUB1	2	160092692	0					0		0	0	0	0	0		0	0				0	0	0	No-ASE
BAZ2B	2	160181398				0		0		0				0	0		0				0		0	No-ASE
mrt-07	2	160585568						0	0			0				0						0		No-ASE
LY75-CD302	2	160628427	0	0				0	0	0		0	0	0	0	0		0		0	0	0	1	No-ASE
PLA2R1	2	160798408	0	0		0						0	0	0	0	0	0	0	0	0	0	0		No-ASE
TANK	2	162061198								0													0	No-ASE
IFIH1	2	163123826	0	0		0		0	0	0				0	0		0	0				0	0	No-ASE
GCA	2	163208877		0				0				0											0	No-ASE
GRB14	2	165349593						1									0	0	0		1			Heterogeneous_ASE
COBLL1	2	165550870				0		0					0	0	0						0			No-ASE
SCN3A	2	165946820		0																				No-ASE
GALNT3	2	166606326																	0					No-ASE
TTC21B	2	166731284						0		0	0		0	0		0	0				0		0	No-ASE
SCN9A	2	167055164											1			1								ASE
SCN7A	2	167262143															1							ASE
XIRP2	2	167759994		1															1					ASE
NOSTRIN	2	169659107				0			0				0		0							0	0	No-ASE
G6PC2	2	169757930				0									0							0		No-ASE
LRP2	2	169985220	0								1	1		0	0		1				0	1		Heterogeneous_ASE
KLHL41	2	170366296															0		0					No-ASE
FASTKD1	2	170386346				0			0		0			0	0			0			0		0	No-ASE
PPIG	2	170440900	0	0		0		0	0		0		0	0	0		0				0	0	0	No-ASE
CCDC173	2	170502355										0		0										No-ASE
MYO3B	2	171055752												0		0	0	0				0		No-ASE
GORASP2	2	171818244	0			0									0		0		0	0				No-ASE
DCAF17	2	172291650																0						No-ASE
CYBRD1	2	172379099	0							1	0	0			0	0	1	0	1		1			Heterogeneous_ASE
SLC25A12	2	172644389												0		0			0				0	No-ASE
ITGA6	2	173292571		0				0		0					0		0							No-ASE
ZAK	2	174047614	0	0				0	0	0			0		0									No-ASE
SP3	2	174774847	0											0							0		0	No-ASE
SCRN3	2	175263063	0			0		0		0			0			0	0	0		0	0			No-ASE
WIPF1	2	175436496										0												No-ASE
KIAA1715	2	176794846															0							No-ASE
HOXD4	2	177016425								0							0							No-ASE
HOXD1	2	177054115								0							0	1						Heterogeneous_ASE
PDE11A	2	178494181		1										1										ASE
OSBPL6	2	179170987										0												No-ASE
MIR548N	2	179300979	1	1				1	0	1	1	0	1	0	1	0	0	1	0		0	0	1	Heterogeneous_ASE
TTN	2	179543504										0		1			0		1					Heterogeneous_ASE
CCDC141	2	179698914								0													1	Heterogeneous_ASE
CWC22	2	180809970	0	0				0											0				0	No-ASE
ITGA4	2	182346352														1								ASE
NEUROD1	2	182542557										0			0			1						Heterogeneous_ASE
SSFA2	2	182756900																0	0			0		No-ASE
DNAJC10	2	183582868						0				0	0		0		0	0						No-ASE
FRZB	2	183699673								1					0		0		1	0			0	Heterogeneous_ASE
ZNF804A	2	185463700						1				1	1	1		0			1			0		Heterogeneous_ASE
FSIP2	2	186627943	0	0				0	0	0	0	0	0	0	0		0		0		0		0	No-ASE
TFPI	2	188331704		0													0					0		No-ASE
COL5A2	2	189898846																			0		1	Heterogeneous_ASE
WDR75	2	190313148				0			0		0							0	0		0	0		No-ASE
ASNSD1	2	190530959								0														No-ASE
ANKAR	2	190541497											0											No-ASE
OSGEPL1	2	190618698	0										0											No-ASE
C2orf88	2	191064591		0				0					0					1	0	0		0		No-ASE
HIBCH	2	191077756		0		0		0				0	0					0		0	0	0		No-ASE
MFSD6	2	191300916				0		0	0	0							0			0			0	No-ASE
MYO1B	2	192160875		0				0	0	0				0								0	0	No-ASE
SDPR	2	192700735																0				1		Heterogeneous_ASE
DNAH7	2	196602679	0	1					0	0			0				0	0		0		0		No-ASE
CCDC150	2	197511096				0																		No-ASE
GTF3C3	2	197641171		0		0																		No-ASE

ANKRD44	2	197870580		0								0		0		0								No-ASE
PLCL1	2	198948717	0	1																		0		Heterogeneous_ASE
TYW5	2	200797806		0																				No-ASE
C2orf47	2	200820546																				0		No-ASE
SPATS2L	2	201284090															0		0					No-ASE
KCTD18	2	201354866	0						0		0	0	0		0		0	0	0	0	0	0		No-ASE
SGOL2	2	201397724				0											0	0					0	No-ASE
PPIL3	2	201736166											0									0		No-ASE
NIF3L1	2	201756740									0		0						0			0	0	No-ASE
CASP10	2	202050562										0												No-ASE
CASP8	2	202122956				0							0	0	0		0		0		0		0	No-ASE
TRAK2	2	202245244								0				0					0	0				No-ASE
TMEM237	2	202490812				0																		No-ASE
MPP4	2	202509938												0										No-ASE
ALS2	2	202566584												0									0	No-ASE
BMPR2	2	203329541									0											0		No-ASE
ICA1L	2	203653587																0		0				No-ASE
WDR12	2	203748336								0														No-ASE
ALS2CR8	2	203806666								0														No-ASE
NBEAL1	2	203880992		0						0							0	0	0				0	No-ASE
CYP20A1	2	204111587		0		0			0			0	0	0	0	0		0	0	0	0	0	0	No-ASE
RAPH1	2	204304163						0											0					No-ASE
PARD3B	2	205829944				0				0		0	0				0		0					No-ASE
NRP2	2	206581033				0							0											No-ASE
INO80D	2	206869724		0		0						0					0	1			0		0	No-ASE
GPR1	2	207040927		1		0		0	1	0		1	1					0				0	0	Heterogeneous_ASE
ZDBF2	2	207162033		1		0				1														Heterogeneous_ASE
MDH1B	2	207603221	0																					No-ASE
FASTKD2	2	207631446						0						0			0					0	0	No-ASE
CPO	2	207804388									0	1		0					1	0	0	0	0	No-ASE
METTL21A	2	208477772		0						0		0	0				0						0	No-ASE
FZD5	2	208632817								0													0	No-ASE
PLEKHM3	2	208773279						0				0									0			No-ASE
LOC100507443	2	208986546															0							No-ASE
C2orf80	2	209035751																				0		No-ASE
IDH1	2	209106854		0																		0		No-ASE
PIKFYVE	2	209138313		0							0										0	0		No-ASE
MAP2	2	210517947	0									0		0				0		0				No-ASE
UNC80	2	210637818												0										No-ASE
RPE	2	210880810												0										No-ASE
CPS1	2	211421452	0			0		1		0	0		0	0		0			0			0	0	No-ASE
IKZF2	2	213872261											0		0									No-ASE
SPAG16	2	214160817																0						No-ASE
BARD1	2	215593452	0	0		0					0	0				0	0	0	0			0		No-ASE
ABCA12	2	215797431												0										No-ASE
FN1	2	216226759																	0			0	1	Heterogeneous_ASE
MREG	2	216811790	0	0		0			0	0								0	0					No-ASE
PECR	2	216903962																					0	No-ASE
SMARCAL1	2	217279551				0						0										0	0	No-ASE
TNS1	2	218669196								0	0	0	0	0		0		0	1	1		1	1	Heterogeneous_ASE
GPBAR1	2	219127905				0					0													No-ASE
PNKD	2	219137451															0							No-ASE
CTDSP1	2	219266385																					0	No-ASE
VIL1	2	219288517														0								No-ASE
USP37	2	219319657										0						0						No-ASE
ZNF142	2	219503220	0	0		0				0			0			0	0			0		0		No-ASE
STK36	2	219540127	0	0		0				0			0			0				0		0		No-ASE
PRKAG3	2	219688502		0							1			0										Heterogeneous_ASE
LOC100129175	2	219868693	0																1					Heterogeneous_ASE
CCDC108	2	219883882							0	0			0	0	0			1	0				1	No-ASE
IHH	2	219919996							0	0			0	0	0			0	0				0	No-ASE
CNPPD1	2	220037444	0						0	0			0	0	0	0		0	0			0	0	No-ASE
FAM134A	2	220046488	0						0	0			0	0	0	0		0	0			0		No-ASE
ZFAND2B	2	220072431																				0	0	No-ASE
ABCB6	2	220074953								0														No-ASE
ATG9A	2	220085847				0						0					0							No-ASE
ANKZF1	2	220095058																0					0	No-ASE
GLB1L	2	220101858				0			0					0			0							No-ASE
DNPEP	2	220239672																0						No-ASE
SPEG	2	220309664										0				0		0					1	No-ASE
ASIC4	2	220379117	0						0	0								0						No-ASE
TMEM198	2	220409614				0																		No-ASE
OBSL1	2	220417266		1				0	0	0		0	0	0	0			0	0			0	0	No-ASE
STK11IP	2	220462640				0			0	0		0				0		0	0		0	0	0	No-ASE
SLC4A3	2	220493280												0										No-ASE
PAX3	2	223084881																				0		No-ASE
MOGAT1	2	223536541						0			0	0										0	0	No-ASE
SCG2	2	224462310	0																					No-ASE
AP1S3	2	224630005								0														No-ASE
MRPL44	2	224822223		0																				No-ASE
FAM124B	2	225244352																				0		No-ASE
CUL3	2	225362478		0																		0		No-ASE
DOCK10	2	225630508													0									No-ASE
IRS1	2	227659785									0			0										No-ASE
COL4A4	2	227867719							0		0					0					0		0	No-ASE
COL4A3	2	228102723	0			0						0		0		0			0					No-ASE
MFF	2	228194481	0			0		0	0									0		0	0		0	No-ASE
TM4SF20	2	228228462							0	0				0		0								No-ASE
AGFG1	2	228388517																			0			No-ASE
C2orf83	2	228476140	0											0				0						No-ASE
DAW1	2	228750073				1										0						1		Heterogeneous_ASE
SPHKAP	2	228855866	0															1						Heterogeneous_ASE
DNER	2	230231632		1										0										Heterogeneous_ASE
SLC16A14	2	230902140															0							No-ASE
SP110	2	231033860	0			0		0		0		0	0	0			0	0	0			0	1	No-ASE
SP140	2	231101805							1				0					0						Heterogeneous_ASE
SP140L	2	231222543		0		0		0	0	0	0	0	0	0	0	0		0		0			1	No-ASE
SP100	2	231311570	0	0		0		0	0			0	0	0	0		0	0	0	0				No-ASE
ITM2C	2	231729802	0	0					0	0		0	0	0						0			0	No-ASE
ARMC9	2	232070961				0		0																No-ASE
B3GNT7	2	232262494										0		0			0							No-ASE
C2orf57	2	232457693												0				0				0		No-ASE
DIS3L2	2	232879671		0		0																		No-ASE
ALPP	2	233243586	0								0	0		0				0					0	No-ASE
ECEL1	2	233344871																					0	No-ASE
CHRNG	2	233404728											0	0										No-ASE
EFHD1	2	233527526				1							0	1										Heterogeneous_ASE
GIGYF2	2	233612427	0			0				0		0			0	0	0	0		0		0		No-ASE
NGEF	2	233745857	0			0							0	1						0		0	0	No-ASE
NEU2	2	233897414																0						No-ASE
INPP5D	2	233990575		1		0		0		1	0	0			0	0		0	0	0	0	1	1	Heterogeneous_ASE
ATG16L1	2	234164834		0		0			0				0		0		0				0			No-ASE
SAG	2	234217866	0															1	1					Heterogeneous_ASE
DGKD	2	234355413										0	0	0		0		1			0			No-ASE
USP40	2	234386091	0							0		0	0			0	0							No-ASE
UGT1A8	2	234526694	0	1					0	0	0	0		0	1	0	0						0	No-ASE
HJURP	2	234749258		0		0			0	0	0	0	0			0	0	0		0		0	0	No-ASE
SH3BP4	2	235943693								0														No-ASE

AGAP1	2	236617868		0		0						0	0	0	0	0	0	0	0		0	0	0	No-ASE
ASB18	2	237103616										0		0			1	1	1			1		Heterogeneous_ASE
IQCA1	2	237233420																0						No-ASE
COL6A3	2	238233427												0				1				0		Heterogeneous_ASE
MLPH	2	238402166	0							0		0	0		0				0					No-ASE
RAB17	2	238483729	0	0		0		0	0			0		0	0				0	0	0		0	No-ASE
LRRFIP1	2	238628188	0			0		0		0	0	0			0	0	0	0			0			No-ASE
UBE2F-SCLY	2	238973062	0	0				0		0	0	0		0		0							0	No-ASE
KLHL30	2	239049528																0						No-ASE
PER2	2	239155053				0				0			0				0		0	0				No-ASE
TRAF3IP1	2	239234520	0	0		0		0		0			0	0	0	0		0			0	0	0	No-ASE
PRR21	2	240981262																1						ASE
GPC1	2	241398543				0		0		0	0	0	0						0	0	0			No-ASE
ANKMY1	2	241439473				0		0		0			0				0	0			0		0	No-ASE
RNPEPL1	2	241513756				0												0						No-ASE
CAPN10	2	241526428				0						0			0		0	1				0		No-ASE
GPR35	2	241558397							0	0	0	0	0	0	0		0	0	0		0	0	0	No-ASE
AGXT	2	241808308																	1	0		1	0	Heterogeneous_ASE
C2orf54	2	241826521						0		0	0								0					No-ASE
SNED1	2	241969887								0					0				0			0		No-ASE
MTERFD2	2	242035466								0										0		0		No-ASE
PASK	2	242045997				0				0				0		0		0	0	0	0	0	0	No-ASE
ANO7	2	242128090	0							0		0	0		0	0		0			0		0	No-ASE
HDLBP	2	242168980									0		0										0	No-ASE
FARP2	2	242312572		0					0								0	0				0		No-ASE
THAP4	2	242541357		0							0		0	0	0			0		0	0			No-ASE
ATG4B	2	242592928		0				0	0	0		0	0					0	0	0				No-ASE
D2HGDH	2	242674803				0		0	0	0		0		0			0	0	0		0	0		No-ASE
GAL3ST2	2	242716380						0					0	0										No-ASE
CHL1	3	361487														1								ASE
CNTN4	3	2613232				0																		No-ASE
LRRN1	3	3886665			0																			No-ASE
SETMAR	3	4345184								0				0				0	0					No-ASE
ITPR1	3	4704816		0	0				0	0			0	0	0	0	0		0		0			No-ASE
GRM7	3	7188188												0										No-ASE
SSUH2	3	8661567				0												0	0					No-ASE
RAD18	3	8923099	0	0	0			0		0								0		0				No-ASE
SETD5	3	9470657	0																					No-ASE
ARPC4-TTLL3	3	9854630	0		0	0		0		0	0		0	0		0	0	0	0		0			No-ASE
CIDEC	3	9908888						0							0									No-ASE
JAGN1	3	9932407				0		0							0									No-ASE
IL17RE	3	9944636		0	0	0		0					0	0	0	0		0	0		0			No-ASE
IL17RC	3	9959033		0	0	0		0		0			0	0	0				0		0			No-ASE
CRELD1	3	9976159																	0					No-ASE
PRRT3	3	9988425														0			1		0			Heterogeneous_ASE
FANCD2	3	10070369												0							0			No-ASE
FANCD2OS	3	10146173																	0					No-ASE
IRAK2	3	10219555						0	0		0		0	0		0	0							No-ASE
TATDN2	3	10290952			0			0	0					0	0			0	0	0	0			No-ASE
GHRLOS	3	10328453		0							0		0					0		0				No-ASE
SLC6A11	3	10858071																0						No-ASE
HRH1	3	11300765							0	0			0				0		0					No-ASE
ATG7	3	11348425									0								0	0				No-ASE
VGLL4	3	11600231			0																			No-ASE
TAMM41	3	11851051							0		0		0		0	0				0				No-ASE
PPARG	3	12393125				0		0							0									No-ASE
TSEN2	3	12531421				0		0																No-ASE
CAND2	3	12845013		0		0		0	1				0		0	0	0	0	0	0	0			No-ASE
IQSEC1	3	12942710		0							0		0	0			0	0	0	0	0			No-ASE
NUP210	3	13360604		0	0			0		0	0		0	0	0	0		0	0	0	1			No-ASE
FBLN2	3	13611988									0		1	0	1	0								Heterogeneous_ASE
TMEM43	3	14170981			0	0		0	0	0			0					0	0					No-ASE
XPC	3	14187449		0	0	0		0		0						0		0	0					No-ASE
SLC6A6	3	14487358						0			0		0				0			0	0			No-ASE
GRIP2	3	14535183				0		0					1					0	0					No-ASE
C3orf20	3	14724264		1	0			0	1		1		0	0					1		1			Heterogeneous_ASE
FGD5	3	14860955			1	0													1					Heterogeneous_ASE
ZFYVE20	3	15115296		0					0				0	0					0					No-ASE
CAPN7	3	15248049				0				0														No-ASE
SH3BP5-AS1	3	15297719						0																No-ASE
HACL1	3	15604875							0		0													No-ASE
GALNT15	3	16216861												0				0						No-ASE
OXNAD1	3	16312496			0	0			0	0			0	0		0	0	0		0				No-ASE
RFTN1	3	16358342				0										0	0	0		0				No-ASE
PLCL2	3	16926648			0	0			0		0		0	0		0	0		1					No-ASE
TBC1D5	3	17202637		0	0	0					0		0					0		0	0			No-ASE
KCNH8	3	19384135									0								0					No-ASE
EFHB	3	19921149				0					0							0						No-ASE
SGOL1	3	20202616						0																No-ASE
UBE2E1	3	23848835		0													0							No-ASE
NEK10	3	27326094				0																		No-ASE
SLC4A7	3	27416013		0	0	0			0								0	0		0				No-ASE
OSBPL10	3	31725213			0	0			0							0				0				No-ASE
ZNF860	3	32030662		0				0		0						0	0		0					No-ASE
CMTM6	3	32533246		0																				No-ASE
DYNC1LI1	3	32576060		0															0					No-ASE
GLB1	3	33038787		0				0		0			0	0		0	0	0	0	0				No-ASE
SUSD5	3	33194241			0											0					0			No-ASE
UBP1	3	33434079		0							0								0					No-ASE
CLASP2	3	33543196		0																				No-ASE
PDCD6IP	3	33840234		0	0	0				0			0	0					0	0	0			No-ASE
TRANK1	3	36871070		0	0	0					0			0				0		0				No-ASE
MLH1	3	37042541				0					0			0				0		0				No-ASE
LRRFIP2	3	37096000																			0			No-ASE
GOLGA4	3	37323459						0	0	0	0		0	0		0	0			0				No-ASE
C3orf35	3	37458758							0											0				No-ASE
ITGA9	3	37512596			0					0	0			0		0	0	0	0					No-ASE
VILL	3	38035950		0				0	0	0	0						0		0	0				No-ASE
PLCD1	3	38049310																	0		0			No-ASE
DLEC1	3	38080763				0		1		0	0			1		0		0						Heterogeneous_ASE
ACAA1	3	38164585		0	0	0		0					0	0		0		0	0					No-ASE
OXSR1	3	38224533		0																	0			No-ASE
SLC22A14	3	38347632		0		0					0			0		0								No-ASE
XYLB	3	38390056		0		0		0	0				0	0				0		0	0			No-ASE
SCN5A	3	38591847			0	0			0		0								0	0	0		1	No-ASE
SCN10A	3	38739054		1					1															ASE
GORASP1	3	39139755																	0					No-ASE
TTC21A	3	39150586		0	0			0			0		0						0	0	0			No-ASE
CSRNP1	3	39184510		0	0														0					No-ASE
XIRP1	3	39225442				0																		No-ASE
CX3CR1	3	39306947		0					0					0		0					0			No-ASE
CCR8	3	39373902			0	0			0	0			0		0				1					No-ASE
SLC25A38	3	39425226					1																	ASE
RPSA	3	39448204		1	0				0		0		0			0								No-ASE
MOBP	3	39543574												0				0						No-ASE
MYRIP	3	40085614								0				0				0			0			No-ASE
ENTPD3	3	40429586			0	0		0		0	0									1				No-ASE
ZNF619	3	40519470								0								0	0					No-ASE

ZNF620	3	40547853							0									0					No-ASE
ULK4	3	41288466		0	0						0								0	0				No-ASE
TRAK1	3	42132983												0					0					No-ASE
LYZL4	3	42448377												0										No-ASE
NKTR	3	42660516		0	0	0							0				0							No-ASE
KLHL40	3	42727198							1	1			0	1			1							ASE
HHATL	3	42734343				0					0			0		0								No-ASE
CCDC13	3	42750516			0	1				0			0	0		1	0			0				No-ASE
CCBP2	3	42906116		1					0		0					0		0		0	0			No-ASE
FAM198A	3	43073796			0	0			0			0		1		0		0	1					No-ASE
GTDC2	3	43121258				0													0					No-ASE
SNRK	3	43388922											0											No-ASE
ANO10	3	43408427				0		0			0	0	0	0				0	0	0	0			No-ASE
ABHD5	3	43743718								0														No-ASE
ZNF445	3	44488293									0								0		0			No-ASE
ZKSCAN7	3	44598609										0									1			Heterogeneous_ASE
ZNF502	3	44762392		1	0	0		0	0	1			0	0		0		0	0	1	0			No-ASE
ZNF501	3	44775959		1	0	0		0	0				0	0		0		0			0			No-ASE
KIAA1143	3	44794955																	0					No-ASE
KIF15	3	44816862		0	0	0		0	0	0			0	0		0	0	0			0			No-ASE
TGM4	3	44926861			0			1	1			0				1								Heterogeneous_ASE
ZDHHC3	3	44974404								0														No-ASE
EXOSC7	3	45017782			0	0			0			0		0				0	0	0				No-ASE
CLEC3B	3	45077123				0						0		0				0		0				No-ASE
CDCP1	3	45127353				0		0		0				0			0		0	0	0			No-ASE
LARS2	3	45436093																	0					No-ASE
LIMD1	3	45636460									0													No-ASE
SACM1L	3	45744976			0				0							0	0	0	0	0				No-ASE
SLC6A20	3	45800505		0	0			1	0	1						0	0	0		0				No-ASE
LZTFL1	3	45869972																	0					No-ASE
FYCO1	3	45963300		0	0	0			0	0	0	0	0	0					0	0				No-ASE
CCR3	3	46306642												0						0				No-ASE
CCR5	3	46414529									0			0										No-ASE
CCRL2	3	46449164			0	0			0	0	0					0	0	0						No-ASE
LTF	3	46479431								0		0					0	0	0					No-ASE
LRRC2	3	46563069								0			0							0				No-ASE
TDGF1	3	46620614			0	0			0	0			0					0	0					No-ASE
LOC100132146	3	46654182			1																			ASE
ALS2CL	3	46712490		0								0							0		0			No-ASE
PRSS45	3	46783849								1		1							1					ASE
CCDC12	3	46963755										0												No-ASE
NBEAL2	3	47030865						0	0	0		0	0	0						0				No-ASE
SETD2	3	47079245						0	0	0		0	0	0		0		0	0	0				No-ASE
KIF9-AS1	3	47277065						0	0	0		0		0		0			0	0				No-ASE
PTPN23	3	47446239						0	0	0		0		0						0				No-ASE
CSPG5	3	47610572		0	0	0		0	0	0									0	0	0			No-ASE
MAP4	3	47894380		0	0			0	0	0	0					0		0	0	0	0			No-ASE
ZNF589	3	48282695							0	0	0						0	0	0	0	0			No-ASE
SPINK8	3	48360992																0						No-ASE
FBXW12	3	48414274		1		0			1		0				0			0	0					Heterogeneous_ASE
PLXNB1	3	48451889											0	0			0							No-ASE
CCDC51	3	48473953																	0					No-ASE
TREX1	3	48507894																		0				No-ASE
COL7A1	3	48602243							1								0	0						Heterogeneous_ASE
UQCRC1	3	48637071				0							0							0				No-ASE
TMEM89	3	48658315				0																		No-ASE
SLC26A6	3	48664473			0	0		0				0					0							No-ASE
CELSR3	3	48677114			0	0		0				0		0										No-ASE
IP6K2	3	48726170			0	0		0				0		0										No-ASE
SLC25A20	3	48896010																	0					No-ASE
ARIH2	3	48965078			0													0						No-ASE
WDR6	3	49044932										0												No-ASE
DALRD3	3	49053918				0		0		0	0		0						0		0			No-ASE
QRICH1	3	49067904				0								0										No-ASE
QARS	3	49135441							0							0	0							No-ASE
USP19	3	49145895				0								0										No-ASE
LAMB2	3	49158670		0	0			0	0	0		0		0		0		0			0			No-ASE
CCDC71	3	49200259						0		0	0		0	0				0	0	0	0			No-ASE
CCDC36	3	49249233				0							0	0										No-ASE
C3orf62	3	49308696				0							0	0										No-ASE
AMT	3	49455040		0	0	0		0	0		0	0		0							0			No-ASE
NICN1	3	49462805															0							No-ASE
DAG1	3	49548008																	0					No-ASE
BSN	3	49662500		0	0	0		0	0		0	0									0			No-ASE
MST1	3	49721532		0	0	0		0	0		0	0		0		0								No-ASE
RNF123	3	49728870				0					0			0									0	No-ASE
GMPPB	3	49759220																	0					No-ASE
CDHR4	3	49831032		1	1			1	1			1									1			ASE
MST1R	3	49924763		0	0	0				0	0	0	0	0					0	0				No-ASE
MON1A	3	49947766		0	0	0				0	0	0	0	0						0				No-ASE
SEMA3F	3	50197110		0	0						0		0	0						0	0			No-ASE
SEMA3B	3	50307645		0																				No-ASE
HYAL3	3	50330813		0	0	0		0					0			0	0							No-ASE
HYAL1	3	50337932		0	0																			No-ASE
RASSF1	3	50368091				0				0		0					0			0				No-ASE
CACNA2D2	3	50402850									1													ASE
C3orf18	3	50597092										0								0				No-ASE
CISH	3	50645081																	0					No-ASE
DOCK3	3	50816121										0												No-ASE
TEX264	3	51708349																	0					No-ASE
IQCF6	3	51812691												1				0		1				Heterogeneous_ASE
PARP3	3	51977385				0								0										No-ASE
PCBP4	3	51992141																	1					ASE
ABHD14A	3	52011906				0													0					No-ASE
ABHD14A-ACY1	3	52019928									0													No-ASE
PPM1M	3	52281713				0			0															No-ASE
DNAH1	3	52356504		0		0		0	0			0	0	0			0		0					No-ASE
PHF7	3	52455056																	1					ASE
NISCH	3	52505834						0																No-ASE
STAB1	3	52535264				0																		No-ASE
PBRM1	3	52582210							0	0				0	0					0	0			No-ASE
GNL3	3	52721305							0	0				0						0	0			No-ASE
SPCS1	3	52740182							0	0				0	0					0	0			No-ASE
NEK4	3	52745881							0	0				0						0	0			No-ASE
ITIH4	3	52848027												0				0		0				No-ASE
TMEM110-MUSTN1	3	52874288												0						0				No-ASE
TKT	3	53259854							0															No-ASE
CACNA1D	3	53700459						0																No-ASE
IL17RB	3	53882694			0							0	0	0					0					No-ASE
ACTR8	3	53905277															0				0			No-ASE
CCDC66	3	56597950				0		0		0		0	0				0	1		0	0			No-ASE
FAM208A	3	56657447		0		0		0		0		0	0				0	0	0	0	0			No-ASE
ARHGEF3	3	56763303		0	0	0		0		0			0		0	0	0		0	0				No-ASE
IL17RD	3	57131719		0	0	0		0	0		0			0		0					0			No-ASE
APPL1	3	57271566				0		0	0	0														No-ASE
DNAH12	3	57335876		0	0			0	0	0	0	0		0		0		0	1	0				No-ASE
PDE12	3	57542348												0										No-ASE
SLMAP	3	57743569				0			0	0	0	0	0	0		0	0	0						No-ASE

FLNB	3	57994565		0	0				0				0	0						0				No-ASE
PXK	3	58355183		0		0		0		0	0	0	0	0		0	0	0	0		0			No-ASE
ACOX2	3	58494664											0											No-ASE
FAM3D	3	58622914			1													0						Heterogeneous_ASE
C3orf67	3	58728270				0				0														No-ASE
PTPRG	3	61548010		1	0	0			0			0		0			0							No-ASE
SNTN	3	63645410								0														No-ASE
ATXN7	3	63898497		0	0	0				0		0					0	0	0				0	No-ASE
ADAMTS9	3	64507857												0										No-ASE
MAGI1	3	65342135						0									0							No-ASE
SLC25A26	3	66287056			0	0		0	0	0		0		0	0	0	0	0	0	0				No-ASE
LRIG1	3	66430695			0				0	0	0	0		0					0	0			0	No-ASE
KBTBD8	3	67053926		0					0				0				0							No-ASE
SUCLG2	3	67411103		0		0				0						0		0		0				No-ASE
TMF1	3	69073206											0	1					0					Heterogeneous_ASE
LMOD3	3	69158250												0										No-ASE
FRMD4B	3	69230061							0		0			0					0					No-ASE
SHQ1	3	72799481		0		0			0		0	0	0	0					0					No-ASE
PPP4R2	3	73047240		0	0	0			0				0	0				0	0	0				No-ASE
CNTN3	3	74315765			1						0													Heterogeneous_ASE
ZNF717	3	75786288							0															No-ASE
ROBO2	3	77147197								0									0					No-ASE
ROBO1	3	78649293										0					0							No-ASE
GBE1	3	81539593				0			0			0		0			0		0	0				No-ASE
CADM2	3	85775665																		1				ASE
CGGBP1	3	88188791						0		0				0										No-ASE
ARL13B	3	93714764										0	0								0			No-ASE
DHFRL1	3	93779860										0	0								0			No-ASE
MINA	3	97664725	0	0	0	0		0	0		0	0		0								0		No-ASE
OR5H6	3	97983231	1					1			1	1	1							1			1	ASE
GPR15	3	98250986								1														ASE
CPOX	3	98299553	0						0	0	0		0	0									0	No-ASE
ST3GAL6	3	98487361			0																			No-ASE
DCBLD2	3	98518223			0			0																No-ASE
MIR548G	3	99509683	0					0				0		0					0	0		0	0	No-ASE
CMSS1	3	99881173						0				0								0		0		No-ASE
LNP1	3	100148679																				0		No-ASE
GPR128	3	100328725	0		0	1					0		0								0		1	Heterogeneous_ASE
ABI3BP	3	100472692														1								ASE
SENP7	3	101044835																				0	0	No-ASE
TRMT10C	3	101283792		0		0				0				0									0	No-ASE
ZBTB11	3	101370015		0						0														No-ASE
NXPE3	3	101504505			0											0								No-ASE
NFKBIZ	3	101570944				0																		No-ASE
ZPLD1	3	102157365	0								0	0						0						No-ASE
ALCAM	3	105243209						0			0		0							0				No-ASE
CBLB	3	105377515	0	0									0								0			No-ASE
CCDC54	3	107096547	0							0						0								No-ASE
HHLA2	3	108021333				0					0		0					0	0	0				No-ASE
MYH15	3	108100406		0				0	1		0			0		1		0		0	0		0	No-ASE
KIAA1524	3	108271082		0							0							0			0			No-ASE
RETNLB	3	108474645		1							0		0					0				1	0	Heterogeneous_ASE
FLJ22763	3	108867128											0		0	0		0		0			1	No-ASE
CD96	3	111261097			0																			No-ASE
PLCXD2	3	111394179											0											No-ASE
PHLDB2	3	111603046		0	0								1											Heterogeneous_ASE
TMPRSS7	3	111760812													0							0		No-ASE
C3orf52	3	111812214																			0			No-ASE
SLC9C1	3	111870771	0	0	0	0		0		0	0		0	0		0		0	0	0		0	0	No-ASE
CD200	3	112059768	0								0										0			No-ASE
BTLA	3	112185025																		0				No-ASE
CD200R1L	3	112538691						1						1								1		ASE
CD200R1	3	112642568			1																	0		Heterogeneous_ASE
GTPBP8	3	112709932											0											No-ASE
C3orf17	3	112724471	0		0								0	0					0		0	0	0	No-ASE
BOC	3	112968708												0										No-ASE
WDR52	3	113010544		0	0						0								0	0				No-ASE
SPICE1	3	113164170		0							0								0					No-ASE
SIDT1	3	113251977				0				0		0												No-ASE
KIAA2018	3	113373930			0	0		0		0			0	0		0						0	0	No-ASE
NAA50	3	113440653						0																No-ASE
ZDHHC23	3	113667642	0	0				0	0	0	0	0		0						0	0		0	No-ASE
KIAA1407	3	113684061														0								No-ASE
ZNF80	3	113953480						1																ASE
IGSF11	3	118621387	0											0										No-ASE
C3orf30	3	118864997		1						0				0						0				No-ASE
B4GALT4	3	118931401										0		0										No-ASE
ARHGAP31	3	119099765									0			0								0		No-ASE
TMEM39A	3	119150836				0							0								0			No-ASE
TIMMDC1	3	119217756			0														0					No-ASE
PLA1A	3	119316828																	0	0				No-ASE
POPDC2	3	119361336	0	1		0			0	0	0	0	0	0						0		0		No-ASE
HGD	3	120352054								0						0				0				No-ASE
STXBP5L	3	120952486	0		0	0																		No-ASE
POLQ	3	121151240	0		0	0		0		0	0								0	0		0	0	No-ASE
ARGFX	3	121289594				1				1			1	1							1			ASE
HCLS1	3	121350748	0	1	0	0		0		1	0	0	1	0						1	0	0	1	Heterogeneous_ASE
GOLGB1	3	121383458		0		0		0	0	0	0	0	0	0		0				0	0	0	0	No-ASE
IQCB1	3	121489286							0		0		0	0						0	0	0		No-ASE
SLC15A2	3	121615340				0							0	0		0				0	0	0	0	No-ASE
CSTA	3	122060340							0		0		0							0		0	1	No-ASE
PARP9	3	122247284			0				0	0	0	0	0		0				0	0	0	0	0	No-ASE
DTX3L	3	122284865			0				0		0	0							0		0	0		No-ASE
PARP15	3	122296647							1					1										ASE
PARP14	3	122399915		0	0							0		0		0			0				0	No-ASE
LOC100129550	3	122607098	0									0												No-ASE
ADCY5	3	123003465										1												ASE
MYLK-AS1	3	123331143			0			0											0			0		No-ASE
MYLK	3	123356997	0																0	0				No-ASE
CCDC14	3	123633720	0		0			0				0		0			0		0				0	No-ASE
KALRN	3	124017713			0						0					0				0		0		No-ASE
UMPS	3	124449406														0						0		No-ASE
MUC13	3	124627024	0		0	0		0	0	0	0		0	0					0			0	0	No-ASE
HEG1	3	124692579	0	0					0			0	1	0				0	1	0				No-ASE
SLC12A8	3	124802888	0		0						0		0		0	1			0	0		0	1	No-ASE
SLC41A3	3	125725272				0		0		0	0								0		0		0	No-ASE
ALDH1L1	3	125824595	0	1		0		0			0	0							1		0		1	Heterogeneous_ASE
CCDC37	3	126132999	0																					No-ASE
CHST13	3	126255166		0				1										0		0			1	Heterogeneous_ASE
CHCHD6	3	126423192		0				0													0			No-ASE
PLXNA1	3	126707755			0				0				0	0					0	0		0		No-ASE
ABTB1	3	127393271				0																		No-ASE
MGLL	3	127410959			0			0					0						0			0		No-ASE
KBTBD12	3	127641968	0								1													Heterogeneous_ASE
EEFSEC	3	127872469																			0			No-ASE
ACAD9	3	128603497																		0				No-ASE
KIAA1257	3	128690076																					1	ASE
EFCC1	3	128722685				0				0														No-ASE

COPG1	3	128971197				0								0										No-ASE
C3orf37	3	129009622									0													No-ASE
EFCAB12	3	129120467								0	0									0			0	No-ASE
MBD4	3	129150385			0								0											No-ASE
PLXND1	3	129275166	0	0	0				0	0	0		0	0		0		0	1	0		0	0	No-ASE
TRH	3	129694666						0			0													No-ASE
COL6A5	3	130095179						0						1	1									Heterogeneous_ASE
COL6A6	3	130279229		0	0			0	0			0											1	No-ASE
NEK11	3	130748640			0			0	0				0					0				0		No-ASE
NUDT16	3	131100699			0																			No-ASE
ACPP	3	132036310												0										No-ASE
DNAJC13	3	132172268			0	0		0	0	0		0		0				0		0		0	0	No-ASE
ACAD11	3	132277849	0																	0				No-ASE
NPHP3-ACAD11	3	132379481																					0	No-ASE
TMEM108	3	133098661																0						No-ASE
TOPBP1	3	133320184	0	0	0	0		0	0	0				0								0	0	No-ASE
TF	3	133467337		0				0	0					1			0		0				1	Heterogeneous_ASE
SLCO2A1	3	133653573							0											0	0		1	No-ASE
AMOTL2	3	134077435			0	0		0		0		0								0	0			No-ASE
CEP63	3	134214206	0	0		0			0	0	0													No-ASE
EPHB1	3	134514510				0			0	0	0	0	1	0		0				0		0		No-ASE
PPP2R3A	3	135720404		0	0			0	0	0	0	0	0							0				No-ASE
PCCB	3	135969392												0										No-ASE
SLC35G2	3	136573358	0	0	0					0			0							0	0			No-ASE
DZIP1L	3	137781674		0	0			1		1		0						0						Heterogeneous_ASE
DBR1	3	137880785									0													No-ASE
MRAS	3	138122122	0		0			0				0		0										No-ASE
ESYT3	3	138153666	0	1	0			0		0	0	0	1								1		0	Heterogeneous_ASE
CEP70	3	138216947	0	0	0			0				0	0			0					0		0	No-ASE
FAIM	3	138329852	0	0				0				0												No-ASE
TRIM42	3	140397135	0																			1		Heterogeneous_ASE
SPSB4	3	140785128		1					1													1		ASE
ZBTB38	3	141162003								0	0													No-ASE
RASA2	3	141272706						0			0													No-ASE
XRN1	3	142030465																			0			No-ASE
ATR	3	142178115	0	0	0			0	0	0		0				0					0	0	0	No-ASE
PLS1	3	142389963		0	0				0				0						0		0	0	0	No-ASE
PCOLCE2	3	142537215												0										No-ASE
PLSCR4	3	145912919			0				0	0		0				0				0	0			No-ASE
PLSCR1	3	146234909										0									0			No-ASE
CPB1	3	148545841			0																			No-ASE
COMMD2	3	149459413			0			0		0	0		0						0	0	0		0	No-ASE
TSC22D2	3	150127576	0	0					0				0			0					0	0	0	No-ASE
EIF2A	3	150280445	0	0	0	0					0	0	0			0			0		0		0	No-ASE
FAM194A	3	150377771	0						1															Heterogeneous_ASE
MED12L	3	150874057	0		0	0					0	0									1			No-ASE
IGSF10	3	151154509	0			0								0									1	No-ASE
AADAC	3	151542533			1									0										Heterogeneous_ASE
MBNL1	3	152058344	0									0								0		0		No-ASE
C3orf79	3	153202348	0																					No-ASE
ARHGEF26	3	153839806									0	0								0		0	1	No-ASE
DHX36	3	153993964	0	0	0	0				0			0							0	0			No-ASE
PLCH1	3	155198866		0				0																No-ASE
C3orf33	3	155481319				0								0		0					0			No-ASE
SLC33A1	3	155547508	0																					No-ASE
LEKR1	3	156570680														0			1					Heterogeneous_ASE
VEPH1	3	156978981	0																				0	No-ASE
MLF1	3	158310353	0	1	1	0		1	0	1		0	0	0		0		1			0		0	Heterogeneous_ASE
GFM1	3	158363463	0		0	0		0	0			0	0	0		0			0		0		0	No-ASE
MFSD1	3	158519861	0		0	0		0		0		0		0									0	No-ASE
IFT80	3	159986266								0								0				0		No-ASE
SMC4	3	160119783												0										No-ASE
SI	3	164697211	0					1	0	0	0			0					0		0			No-ASE
ZBBX	3	166958644						0																No-ASE
SERPINI2	3	167167160	0											0										No-ASE
WDR49	3	167196724												0										No-ASE
MECOM	3	168802737																	0		0		0	No-ASE
MYNN	3	169492101				0		0		0		0		0								0		No-ASE
LRRC34	3	169511431						0		0		1								0		0		No-ASE
LRRC31	3	169557891											0											No-ASE
PHC3	3	169820366		0				0																No-ASE
PRKCI	3	169998015									0													No-ASE
SKIL	3	170078232		0						0					0	0		0						No-ASE
SLC7A14	3	170184949										0		1										Heterogeneous_ASE
TNIK	3	170802910		0	0	0			0	0				0					0		0			No-ASE
PLD1	3	171321023						0							0	0				0	0		0	No-ASE
FNDC3B	3	171965405	0		0			0		0	0	0	0		0	0				0				No-ASE
GHSR	3	172162980												0										No-ASE
NCEH1	3	172351370	0	0		0		0	0		0	0				0		0	0		0	0	0	No-ASE
SPATA16	3	172607362			1										1									ASE
NAALADL2	3	174814613	0		0	0			0	0	0	0	0	0	0				0	0	0		1	No-ASE
KCNMB3	3	178960744	0		0			0			0	0	0			0			0					No-ASE
MRPL47	3	179306748		0																				No-ASE
LAMP3	3	182841990	0		0	0										0			0	0	0			No-ASE
MCF2L2	3	182897920	0		1	0							0	0						0		0	0	No-ASE
YEATS2	3	183432967			0			0	0		0	0	0	0					0	0			0	No-ASE
PARL	3	183547415			0	0			0	0	0	0	0	0		0		0	0			0		No-ASE
ABCC5	3	183665155		0	0				0	0	0		0	0		0				0		0	0	No-ASE
HTR3E	3	183818222		1							0											1		Heterogeneous_ASE
EIF2B5	3	183853307		0		0		0	0	0		0	0	0		0			0		0	0		No-ASE
AP2M1	3	183899559						1					0											Heterogeneous_ASE
ABCF3	3	183904384							0	0		0		0			0							No-ASE
ALG3	3	183960671	0																					No-ASE
ECE2	3	183967677								0	0			0										No-ASE
PSMD2	3	184018215						0			0			0										No-ASE
EIF4G1	3	184035172				0						0	0	0		0						0	0	No-ASE
CLCN2	3	184064408														0						0		No-ASE
CHRD	3	184099050	0					0																No-ASE
MAGEF1	3	184428747		0		0		0				0	0			0			0		0		0	No-ASE
VPS8	3	184542480	0		0	0				0	0	0	0	0		0					0	0	0	No-ASE
C3orf70	3	184800841												0		0								No-ASE
EHHADH	3	184910025												0					0					No-ASE
MAP3K13	3	185146473																					0	No-ASE
LIPH	3	185229464	0	0		0					0			0				0			0		0	No-ASE
SENP2	3	185327128	0			0		0			0	0	0	0					0				0	No-ASE
DNAJB11	3	186301661	0									0	0											No-ASE
AHSG	3	186331138	0											0				0			1			No-ASE
FETUB	3	186358297							0															No-ASE
HRG	3	186383798	0											0							0	0		No-ASE
KNG1	3	186435412	0		0			0	0		0	1	0	0					0		0		1	No-ASE
RTP4	3	187086251	0	1	0			0	0		0		0	0				0	0				0	No-ASE
LOC100131635	3	187444543	0		0						0	0								0				No-ASE
LPP	3	188123957	0	0		0		0	0		0	0	0	0		0			0	0	0	0		No-ASE
CLDN1	3	190026083		0				0						0								0	1	No-ASE
CLDN16	3	190105985	0			1							1					0			0		0	Heterogeneous_ASE
CCDC50	3	191075901	0	0				0				0	0	0		0			0	0	0	0	0	No-ASE
PYDC2	3	191178962																0		1				Heterogeneous_ASE
ATP13A4	3	193120535	0							0		1						0	0		0			No-ASE

OPA1	3	193332522	0	0	0					0	0	0		0				0			0			No-ASE
LOC647323	3	193711182	0			0			0												0			No-ASE
TMEM44	3	194325055	0	0	0	0		0	0	0	0		0	0		0			0	0		0	1	No-ASE
LSG1	3	194362826	0			0			0		0		0	0		0		0	0			0		No-ASE
MUC4	3	195474126			1			1	0		0			0	0	0			0		0	1	1	Heterogeneous_ASE
TNK2	3	195591029	0					0	0											0		0	0	No-ASE
TFRC	3	195778839	0		0	0					0		0			0		0	0	0		0		No-ASE
SLC51A	3	195943590		0	0			0			0	1	0	0				0			0	0	1	No-ASE
TM4SF19-TCTEX1D2	3	196050696		0		0			0	0				0				0		0		0		No-ASE
RNF168	3	196198925		0								0	0	0		0		0	0			0		No-ASE
CEP19	3	196434643	0	0						0		0								0		0		No-ASE
PIGX	3	196454936				0																		No-ASE
PIGZ	3	196673831	0	0		0				0			0			0			0			0	0	No-ASE
DLG1	3	196771513	0			0						0	0			0				0			0	No-ASE
KIAA0226	3	197403779			0					0										0	0			No-ASE
FYTTD1	3	197495334																					1	ASE
LRCH3	3	197544082		0							0									0				No-ASE
IQCG	3	197616551		0		0			0		0									0				No-ASE
LMLN	3	197687102				0			0					0										No-ASE
ZNF732	4	265547			1																			ASE
ABCA11P	4	435522										0												No-ASE
PIGG	4	494188			0						0													No-ASE
ATP5I	4	667134										0												No-ASE
MYL5	4	672459														0								No-ASE
GAK	4	843498				0		0						0							0		0	No-ASE
TMEM175	4	941940				0																		No-ASE
DGKQ	4	954422									0													No-ASE
SLC26A1	4	981602									0												0	No-ASE
IDUA	4	994414				0									0					0	0			No-ASE
FGFRL1	4	1016197									0	0		0	0	0								No-ASE
RNF212	4	1066769			1																1			ASE
SPON2	4	1164219			0			1						0		0								No-ASE
MAEA	4	1305802										0								0			0	No-ASE
UVSSA	4	1341896									0				0									No-ASE
SLBP	4	1695433			0						0	0								0	0			No-ASE
TMEM129	4	1719294		1		0					0			0										No-ASE
TACC3	4	1725159			0	0						0		0						0	0			No-ASE
LETM1	4	1815990																			0			No-ASE
POLN	4	2073958				0		0						0	0									No-ASE
HAUS3	4	2233709				0																		No-ASE
ZFYVE28	4	2273127						0							0									No-ASE
LOC402160	4	2438704						0				0			0									No-ASE
SH3BP2	4	2822423						0			0	0				0					0			No-ASE
ADD1	4	2877658				0									0									No-ASE
MFSD10	4	2932668										0												No-ASE
NOP14-AS1	4	2940916			0						0			0	0					0	0			No-ASE
GRK4	4	2986333			0							0		1	1					1	0		1	Heterogeneous_ASE
HTT	4	3109012				0		0				0		0	0					0	0		0	No-ASE
RGS12	4	3317943				0		0				0												No-ASE
DOK7	4	3475166			0	0		0				0		0	0						0		0	No-ASE
LRPAP1	4	3514715				0		0													0		0	No-ASE
LYAR	4	4269672										0								0				No-ASE
ZBTB49	4	4301786			0			0						0	0					0				No-ASE
EVC	4	5721021										0			0									No-ASE
WFS1	4	6279223			0	0		0						0						0				No-ASE
MAN2B2	4	6577023						1			0	0		0	0					0	0		0	No-ASE
BLOC1S4	4	6718502			0							0		0		0				0			0	No-ASE
TBC1D14	4	6925198			0											0				0	0			No-ASE
SORCS2	4	7398031										0			0									No-ASE
AFAP1	4	7783273			0			0			0													No-ASE
SH3TC1	4	8207058				0																		No-ASE
ACOX3	4	8372686												0									0	No-ASE
TRMT44	4	8443084				0		0			0			0		0				0	0		0	No-ASE
GPR78	4	8582761			0																			No-ASE
SLC2A9	4	9836555			0	0		0			0	0			1	0				0	0		1	No-ASE
WDR1	4	10079377			0						0	0			0	0				0	0			No-ASE
ZNF518B	4	10445207				1					1	0			1						0			Heterogeneous_ASE
BOD1L1	4	13571720				0		0			0	0			0	0				0	0		0	No-ASE
CC2D2A	4	15482360						0															0	No-ASE
FAM200B	4	15688604				0		0							0					0			0	No-ASE
BST1	4	15707179						0						0	0					0	1		0	No-ASE
FGFBP2	4	15964090										0												No-ASE
PROM1	4	15985975																			0			No-ASE
FAM184B	4	17634213				0						0		0							0			No-ASE
DCAF16	4	17805176									0													No-ASE
NCAPG	4	17812750				0		0			0	0		0						0			0	No-ASE
SLIT2	4	20259514			0																0			No-ASE
GPR125	4	22389387				0		0							0									No-ASE
GBA3	4	22737768																					0	No-ASE
PPARGC1A	4	23797521			0																			No-ASE
SOD3	4	24801226						0				0									0		0	No-ASE
CCDC149	4	24810040										0											0	No-ASE
LGI2	4	25005356						0				1												Heterogeneous_ASE
PI4K2B	4	25256846		1	0	0		0				0			0	0				0	0			No-ASE
ZCCHC4	4	25314478										0												No-ASE
ANAPC4	4	25408838			0	0		0				0			0					0	0			No-ASE
SLC34A2	4	25664344		0																				No-ASE
ARAP2	4	36069541						0																No-ASE
KIAA1239	4	37432261			0			0													0		1	No-ASE
C4orf19	4	37591785						0						0							0			No-ASE
TBC1D1	4	37903756				0		1	0		0	0		1		0							0	No-ASE
TLR1	4	38798235			0						0					0								No-ASE
FAM114A1	4	38879818			0	0					0	0									0			No-ASE
KLHL5	4	39064162						1															1	ASE
WDR19	4	39201181							0												0			No-ASE
KLB	4	39408595			0			0							0									No-ASE
UGDH	4	39500514										0		0	0						0			No-ASE
N4BP2	4	40103767									0	0	0		0	1				0			1	Heterogeneous_ASE
RBM47	4	40428010						0							0					0				No-ASE
NSUN7	4	40752736			0						0	0				0				0	0		0	No-ASE
APBB2	4	40818170									0			0		0				0	0			No-ASE
LIMCH1	4	41362924										0			0	0							0	No-ASE
SLC30A9	4	41992715									0		0	0	0								0	No-ASE
GUF1	4	44680658									0		0											No-ASE
ATP10D	4	47514660			0	0		0	0		0	0	0	0	0	0					0		0	No-ASE
CORIN	4	47597749			0			0	1															Heterogeneous_ASE
NFXL1	4	47850197				0		0	0			0		0						0			0	No-ASE
NIPAL1	4	48027129							0		0													No-ASE
TXK	4	48082095						0			0	0			0					0			1	No-ASE
TEC	4	48147513			0			0						0		0					0		1	No-ASE
ZAR1	4	48493237										0												No-ASE
FRYL	4	48502177																		0				No-ASE
CWH43	4	48988450				0			0											0				No-ASE
SPATA18	4	52926657				0		0	0			0												No-ASE
SCFD2	4	53740210			0				0				0			0							0	No-ASE
KIT	4	55561758		0					0															No-ASE
CLOCK	4	56304494							0															No-ASE
KIAA1211	4	57164405			0	0		0	0		0	0	0							0	0			No-ASE

AASDH	4	57204777				0		0	0		0	0	0			0					0			No-ASE
PAICS	4	57307919										0				0								No-ASE
HOPX	4	57514924			1							0		1		1								ASE
REST	4	57777078			0	0			0				0	0										No-ASE
LPHN3	4	62599227				1		1	0					0		1				0				Heterogeneous_ASE
CENPC1	4	68340623				0		0																No-ASE
UBA6	4	68484738						0						0						0	0			No-ASE
FTLP10	4	69078113			0																			No-ASE
TMPRSS11E	4	69337325			0			0																No-ASE
ODAM	4	71063018				0		0	0															No-ASE
CSN3	4	71114793						0																No-ASE
CABS1	4	71200799						0																No-ASE
MUC7	4	71346606												0										No-ASE
RASSF6	4	74442010			0	0		0				0				0				0				No-ASE
CXCL2	4	74964625									0													No-ASE
BTC	4	75673298				0								0							0		0	No-ASE
C4orf26	4	76481300				1										0								Heterogeneous_ASE
USO1	4	76695830				0			0			0		0		0				0	0			No-ASE
NAAA	4	76836110			0	0		0	0			0		0						0	0			No-ASE
SDAD1	4	76871949				0			0		0			0						0		1		No-ASE
NUP54	4	77038912							0		0			0		0				0			0	No-ASE
SCARB2	4	77084386									0			0										No-ASE
FAM47E	4	77184996				0		0			0	0				0				0	0			No-ASE
SHROOM3	4	77357273			0	0		0				0		0		0				0			0	No-ASE
SOWAHB	4	77816631		1							0	0		0						0	0		0	No-ASE
FRAS1	4	79158704			0	0		1	0		0	0		0		0				1	0		0	No-ASE
ANXA3	4	79494343																			0			No-ASE
BMP2K	4	79747281			0															0				No-ASE
GK2	4	80327886																		1				ASE
SCD5	4	83552538				0		0			0	0		0		0					0		0	No-ASE
THAP9	4	83827601			0			0			0			0		0				0			0	No-ASE
HPSE	4	84216580				0						0								0				No-ASE
HELQ	4	84328632			0	0			0			0		0		0				0	0			No-ASE
FAM175A	4	84383735			0				0			0		0		0				0	0			No-ASE
AGPAT9	4	84457903			0																			No-ASE
CDS1	4	85525406							0															No-ASE
ARHGAP24	4	86491702			0																			No-ASE
PTPN13	4	87556416			0	0		0			0	0		0							0			No-ASE
SLC10A6	4	87744883			0									0										No-ASE
C4orf36	4	87808969			0						0													No-ASE
AFF1	4	87967323			0			0												0			0	No-ASE
SPARCL1	4	88400666				0			0		0			0		1					0			No-ASE
DSPP	4	88533290				1																		ASE
DMP1	4	88578209						1																ASE
IBSP	4	88723842												1										ASE
HERC6	4	89300101			0									0		0				0	0		0	No-ASE
HERC5	4	89380540							1												0			Heterogeneous_ASE
HERC3	4	89577147												0						0			0	No-ASE
FAM13A	4	89653153			0									0		0				0	0			No-ASE
TIGD2	4	90034309							0			0								0	0		0	No-ASE
GPRIN3	4	90168572			0	0		0	0		0	0								0	0			No-ASE
MMRN1	4	90816294							1															ASE
CCSER1	4	91229455										0											0	No-ASE
SMARCAD1	4	95129699			0	0		0	0		0	0		0						0	0			No-ASE
PDLIM5	4	95376504			0	0			0		0	0		0		0					0			No-ASE
BMPR1B	4	96025586			0			1																Heterogeneous_ASE
UNC5C	4	96091324										1												ASE
STPG2	4	98480255		0							0	0											0	No-ASE
LOC100507053	4	100045616							0															No-ASE
ADH1C	4	100260783				0			0														1	Heterogeneous_ASE
ADH7	4	100336659																			1			ASE
MTTP	4	100503146			0	0			0		0										0			No-ASE
H2AFZ	4	100869244			0						0													No-ASE
DDIT4L	4	101108057												0		0							0	No-ASE
BANK1	4	102751014			1			0			1	0		0							1		0	Heterogeneous_ASE
MANBA	4	103553221						0	0		0			0						0	0		0	No-ASE
SLC9B2	4	103947631							0											1	0		0	No-ASE
CENPE	4	104027405				0	1							0						0			0	No-ASE
TACR3	4	104510766						0																No-ASE
TET2	4	106155121			0	0		0	0		0	0		0		0							0	No-ASE
PPA2	4	106307762							0		0	0		0		0					0			No-ASE
ARHGEF38	4	106473983														0								No-ASE
NPNT	4	106819089			0				0		0												0	No-ASE
TBCK	4	107016742				0					0	0								0	0			No-ASE
DKK2	4	107842959			0							0												No-ASE
AGXT2L1	4	109663672									1	1												ASE
CCDC109B	4	110581363			0			0	0		0					0				0	0		0	No-ASE
LRIT3	4	110772701			0						0			0									1	No-ASE
EGF	4	110834537			0	0		0	0		0	1		0						0	0		0	No-ASE
ENPEP	4	111397628			0									0										No-ASE
ALPK1	4	113303616				0		0	0		0									0	0		0	No-ASE
C4orf21	4	113460814			0	0			0					0		0					0		0	No-ASE
LARP7	4	113565835						1													0			Heterogeneous_ASE
ANK2	4	114095617			1							0												Heterogeneous_ASE
PRSS12	4	119203216			0	0		0	0			0												No-ASE
SYNPO2	4	119947922			0	1			0		0	0		0		0							1	No-ASE
USP53	4	120166520			0	0		0	0		0									0				No-ASE
FABP2	4	120240720										0											0	No-ASE
PDE5A	4	120422407			0											0				0			0	No-ASE
NDNF	4	121957051																			1			ASE
QRFPR	4	122250537												1										ASE
CCNA2	4	122738801							0		0													No-ASE
KIAA1109	4	123094236			0							0				0				0	0		0	No-ASE
BBS12	4	123663163									0					0					0			No-ASE
FGF2	4	123747942				0		0	0														0	No-ASE
NUDT6	4	123838685			0				0			0								0	0			No-ASE
FAT4	4	126237639							1			0												Heterogeneous_ASE
INTU	4	128554281																		0				No-ASE
HSPA4L	4	128715251			0																		0	No-ASE
PLK4	4	128802306		0		0										0					0		0	No-ASE
MFSD8	4	128841867																			0			No-ASE
LARP1B	4	128998952														0	1						0	Heterogeneous_ASE
SCLT1	4	129805634			0						0										0		0	No-ASE
C4orf33	4	130023884				0		0	0			0		0		0					0		0	No-ASE
PCDH18	4	138442265																			0			No-ASE
MGARP	4	140187879			0																			No-ASE
MGST2	4	140624612			0	0			0												0			No-ASE
SCOC	4	141294814				0																		No-ASE
CLGN	4	141310389															1						1	ASE
UCP1	4	141481151																					0	No-ASE
TBC1D9	4	141543915																			0			No-ASE
IL15	4	142649121												0										No-ASE
INPP4B	4	143007392						0				0				0								No-ASE
GAB1	4	144354745				0			0															No-ASE
FREM3	4	144498768										1												ASE
MMAA	4	146560560																		0				No-ASE
POU4F2	4	147560411							1											1	1			ASE
PRMT10	4	148559696				0			0		0													No-ASE

NR3C2	4	149035319				0		0						0						0				No-ASE
LRBA	4	151186882				0			0		0			0							0		0	No-ASE
FAM160A1	4	152498875																		0				No-ASE
PET112	4	152592396						0															0	No-ASE
TIGD4	4	153690691				0			0			0		0							0			No-ASE
FHDC1	4	153864319				0										0					0			No-ASE
KIAA0922	4	154388363									0			0							0		0	No-ASE
TLR2	4	154624136																			0			No-ASE
RNF175	4	154631543										1												ASE
DCHS2	4	155155797				0					0	1											1	Heterogeneous_ASE
FGB	4	155484174																		1				ASE
MAP9	4	156269027												1										ASE
TDO2	4	156824932							0															No-ASE
TMEM144	4	159136405										0												No-ASE
ETFDH	4	159601676							0														0	No-ASE
PPID	4	159630817				0		0			0	0		0							0		0	No-ASE
FNIP2	4	159747079																					0	No-ASE
NAF1	4	164048199									0			0						0	0		0	No-ASE
TKTL2	4	164393029														0								No-ASE
TRIM61	4	165878311							0															No-ASE
ANXA10	4	169060675												0										No-ASE
DDX60	4	169141828						0	0					0										No-ASE
DDX60L	4	169279300				0		0	0			0		0		0				0	0		0	No-ASE
PALLD	4	169432663							0		0			0									0	No-ASE
CBR4	4	169911312		1		0					0	0		0										No-ASE
SH3RF1	4	170037572				0		0	0		0	0		0										No-ASE
NEK1	4	170322819				0		0								0				0				No-ASE
C4orf27	4	170663235						0				0		0										No-ASE
CEP44	4	175220223						0	0		0	0		0		0					0			No-ASE
WDR17	4	177017678				0																		No-ASE
NEIL3	4	178231223						0			0					0				0	0		0	No-ASE
AGA	4	178352938																			0			No-ASE
WWC2	4	184175037				0			0		0	0		0						1				No-ASE
CLDN24	4	184242959									0										0			No-ASE
CDKN2AIP	4	184366755				0			0											0				No-ASE
TRAPPC11	4	184585165		1				0				0		0		0				0			0	No-ASE
CCDC111	4	185580557										0									0			No-ASE
MLF1IP	4	185616486			1							0									0	1		Heterogeneous_ASE
ACSL1	4	185678110							0		0			0						0	0			No-ASE
KIAA1430	4	186084055				0								0		0					0			No-ASE
SNX25	4	186180187							0											0				No-ASE
ANKRD37	4	186318347				0			0											0				No-ASE
CCDC110	4	186379286						0																No-ASE
SORBS2	4	186508808						0	0											0			0	No-ASE
TLR3	4	186997767				0		0	0			0		0		0		1		0			0	No-ASE
FAM149A	4	187074833				0								0									0	No-ASE
CYP4V2	4	187113041				0		0	0			0				0		1		0	0		0	No-ASE
KLKB1	4	187153290				0		0				1											0	No-ASE
FAT1	4	187509760		1		0		0	0		0	0		0		0		0		0	0		0	No-ASE
PLEKHG4B	5	140474	0		0				1		0	0					0						1	Heterogeneous_ASE
SDHA	5	223624	0			0					0							0						No-ASE
AHRR	5	353885		0		0		0				0		0					0					No-ASE
C5orf55	5	442586	0												0									No-ASE
SLC9A3	5	475076	0								0				0						0			No-ASE
CEP72	5	619113		0	0				0	0	0	0		0	0		0		0		0			No-ASE
TPPP	5	665148		0													0							No-ASE
BRD9	5	878470																		0				No-ASE
NKD2	5	1032318																		0				No-ASE
SLC12A7	5	1052486	0	0	0	0			0	0		0			0			0	0		0			No-ASE
SLC6A19	5	1201806		0				0																No-ASE
SLC6A18	5	1225626				0	0					1						0		0			0	No-ASE
TERT	5	1254614				1																		ASE
IRX4	5	1878325										0												No-ASE
IRX2	5	2748621			1	0			1										1	1				ASE
ADAMTS16	5	5140798	0				0		0		0				0			0		0	0	1	0	No-ASE
KIAA0947	5	5441314	0	0		0			0		0			0		0			0		0			No-ASE
NSUN2	5	6600064		0	0					0				0		1	0	0	0			0	1	No-ASE
ADCY2	5	7520881																0						No-ASE
FASTKD3	5	7861279	0			0					0	0				0		0	0		0			No-ASE
MTRR	5	7869328	0	0	0	0			0	0	0	0		0		0	0	0	0	0	0	0	0	No-ASE
SEMA5A	5	9041924			0	0			0		0	0		0			0		0		0			No-ASE
FAM173B	5	10227566			0	0		0	0		0	0			0			0	0	0		0	0	No-ASE
DNAH5	5	13692131	0	0	0	0		0	0	0	0	0		0		0	0	0	1		0	0	0	No-ASE
TRIO	5	14270975																		0				No-ASE
MYO10	5	16668390			0		0	0	0		0	0		0	0	0	0		0	0	0	0		No-ASE
CDH12	5	21751961															1	0						Heterogeneous_ASE
DROSHA	5	31435971	0	0	0				0	0	0			0		0	0					0		No-ASE
C5orf22	5	31532513						0	0			0			0		0	0				0		No-ASE
PDZD2	5	31799371			0						0	0		0					0	0			0	No-ASE
MTMR12	5	32229904										0					0							No-ASE
ZFR	5	32356015	0			1				0				0	0					0			0	No-ASE
SUB1	5	32601205					0																	No-ASE
NPR3	5	32724806	0																					No-ASE
TARS	5	33441193													0				0	0				No-ASE
SLC45A2	5	33944827							0															No-ASE
AMACR	5	33988445		0	0			0	0	0				0		0		0	0	0			0	No-ASE
RAI14	5	34687817	0	0	0			0	0						0		0	0			0	0	0	No-ASE
TTC23L	5	34840840						0	0	0		0		0			0	0						No-ASE
RAD1	5	34909420										0												No-ASE
PRLR	5	35065328			0				0															No-ASE
SPEF2	5	35641582		0	0	0	0	0	0		0			0				0	0	0	0	0	0	No-ASE
IL7R	5	35861023	1	1		1			0		0	1				0					0	0		Heterogeneous_ASE
SLC1A3	5	36608593																		0				No-ASE
NIPBL	5	36955688							0		0									0	0		0	No-ASE
C5orf42	5	37108520			0			0	0		0	0							0	0	0		0	No-ASE
EGFLAM	5	38338807												0					1	0				Heterogeneous_ASE
LIFR	5	38481703										1												ASE
OSMR	5	38869210				1								1										ASE
RICTOR	5	38942449		0	0	0		0		0	0	0						0	0	0	0	0	0	No-ASE
C9	5	39285311										1			1			0				0		Heterogeneous_ASE
DAB2	5	39376173									0					0	0			0				No-ASE
PTGER4	5	40691893						0						0										No-ASE
CARD6	5	40841512	0		0				0											0	0	0	1	No-ASE
C7	5	40931237		0		0			1		0								1			0		Heterogeneous_ASE
GHR	5	42565967				0																		No-ASE
CCDC152	5	42759230	0	0		0		0			0					1			0			0		No-ASE
ANXA2R	5	43039593			0				0	0		0		0	0			0						No-ASE
ZNF131	5	43161351		0							0								0				0	No-ASE
NNT	5	43609377																		0				No-ASE
PARP8	5	50057670										0		0					0	0			0	No-ASE
PELO	5	52096889	0								0			0					0					No-ASE
ITGA1	5	52160871	0	0	0	0	0				0	1			0					0			0	No-ASE
ITGA2	5	52337983										0		0							0			No-ASE
SNX18	5	53814428	0	1		0		0				0		0	0				0		0		0	No-ASE
CDC20B	5	54410065			0	0	0				0	0		0										No-ASE
SKIV2L2	5	54603822			0		0	1			0													No-ASE
SLC38A9	5	54922404	0			0	0	0		0		0				0			0	0				No-ASE
DDX4	5	55034832					0																	No-ASE

IL6ST	5	55237014					0												0					No-ASE
ANKRD55	5	55396074					0																	No-ASE
MAP3K1	5	56155618				0	0				0	0		0	0						0		0	No-ASE
SETD9	5	56205479	0		0	1	0	0		0	0	0			0				0		1			No-ASE
ZSWIM6	5	60768550												0										No-ASE
FAM159B	5	63986463										1												ASE
SREK1IP1	5	64020316		0																	0			No-ASE
PPWD1	5	64859234		0	0	0	0					0		0					0					No-ASE
TRAPPC13	5	64933563						0																No-ASE
NLN	5	65054513													0									No-ASE
ERBB2IP	5	65288668		1		0	0	0				0			0									No-ASE
MAST4	5	65892523			0	0														0	0			No-ASE
CD180	5	66478729									1	0												Heterogeneous_ASE
PIK3R1	5	67575490	0			0	0	0			0	0			0				0				0	No-ASE
CENPH	5	68487643		0																				No-ASE
CCDC125	5	68578647	0	0		0		0						0									0	No-ASE
RAD17	5	68668039														0								No-ASE
MARVELD2	5	68715249	0	0			0	0			0			0		0			0				0	No-ASE
OCLN	5	68804987																			0			No-ASE
BDP1	5	70751781		0		0						0							0	0	0		0	No-ASE
MAP1B	5	71403533					0					0				0					0		1	No-ASE
MRPS27	5	71516795	0	0		0	0	0							0					0	0			No-ASE
FCHO2	5	72313066		0		0		0																No-ASE
TMEM171	5	72419251	0	0		0		0						0	0				0	1	0		0	No-ASE
TMEM174	5	72469090					0																	No-ASE
FOXD1	5	72742882						0						0										No-ASE
UTP15	5	72864061																	0					No-ASE
ARHGEF28	5	72980709	0	0		0	0	0			0	0		0	0				0	0	0			No-ASE
HEXB	5	73981299		0		1					0	0									0			No-ASE
GFM2	5	74017499		0							0													No-ASE
NSA2	5	74065169		0			0				0													No-ASE
GCNT4	5	74324548				1					0			0							0			No-ASE
ANKRD31	5	74400151		0			0				0									0	0			No-ASE
HMGCR	5	74638536																					0	No-ASE
COL4A3BP	5	74675250	0																					No-ASE
POC5	5	74970024	0	0		0		0			0	0		0					0	0	0			No-ASE
IQGAP2	5	75866423		0		0	0				0	1			0				0					No-ASE
F2RL1	5	76115069																			0			No-ASE
AGGF1	5	76326719				0	0					0							0	0			0	No-ASE
PDE8B	5	76607841				1																		ASE
WDR41	5	76728974	0			0	0	0			0			0	0				0	0	0			No-ASE
ARSB	5	78076276					0				0			0	0					0	0		0	No-ASE
DMGDH	5	78294107	0	1				0				0		0	0					0	0			No-ASE
BHMT	5	78411602	0	0			0					1			0					0				No-ASE
JMY	5	78533410	0	0			0							0	0					0				No-ASE
HOMER1	5	78670802		0			0					0		0							0			No-ASE
CMYA5	5	79024747				0	0				0	0		0	0						0		1	No-ASE
MTX3	5	79279578									0										0		0	No-ASE
THBS4	5	79335944									0	0									0		0	No-ASE
SPZ1	5	79616083					0																	No-ASE
ZFYVE16	5	79732631				0					0								0					No-ASE
FAM151B	5	79797690									0	0								0			0	No-ASE
ANKRD34B	5	79854441				0						1		0										Heterogeneous_ASE
DHFR	5	79950781				0	0				0	0			0								0	No-ASE
MSH3	5	79952308						0						0									0	No-ASE
RNU5E-1	5	80547052				0						0											0	No-ASE
ATG10	5	81283451	0	0		0		0			0	0		0	0				1		0		0	No-ASE
ATP6AP1L	5	81601230												0										No-ASE
VCAN	5	82786067				1	0					1									1		0	Heterogeneous_ASE
EDIL3	5	83239249	1												0									Heterogeneous_ASE
CCNH	5	86695211					0	0			0									0			0	No-ASE
GPR98	5	89910784	0	0		0	0				0	0		0	1								0	No-ASE
KIAA0825	5	93489746	0								0			0							0		0	No-ASE
ANKRD32	5	93966249	0									0		0									0	No-ASE
MCTP1	5	94044279	0									0												No-ASE
TTC37	5	94814011				0						0											0	No-ASE
ARSK	5	94891059				0						0												No-ASE
SPATA9	5	94994467		0															0					No-ASE
RHOBTB3	5	95067622	0			0					0	0			0						0			No-ASE
ELL2	5	95233949										0											0	No-ASE
PCSK1	5	95728710	0					0			0	1			0				0					No-ASE
CAST	5	96031558						0			0			0						0	0			No-ASE
ERAP1	5	96112083		0		0		0			0	0		0	0				0	0	0		0	No-ASE
ERAP2	5	96215503		0		0						1							0	0	0			No-ASE
LNPEP	5	96314935		0		0					0	0							0	0	0			No-ASE
RIOK2	5	96498904				0	0	0			0			0							0			No-ASE
CHD1	5	98192095					0				0	0		0										No-ASE
SLCO4C1	5	101572564				1																	1	ASE
PAM	5	102201978						0												0	0			No-ASE
GIN1	5	102423628		0		0	0	0							0					0				No-ASE
PPIP5K2	5	102490411						0												0				No-ASE
PJA2	5	108672946		0		0									0				0		0		0	No-ASE
MAN2A1	5	109049221				0	0				0								0		0			No-ASE
TMEM232	5	109756436																	0					No-ASE
SLC25A46	5	110074872																	0					No-ASE
WDR36	5	110428060		0		0		0				0								0	0			No-ASE
EPB41L4A	5	111500706					0				0	0											0	No-ASE
APC	5	112090657	0	0		0		0				0		0	0						0			No-ASE
DCP2	5	112312655		0		0	0	0			0			0						0			0	No-ASE
MCC	5	112364689	0			0	0																	No-ASE
YTHDC2	5	112849608	0			0	0	0			0				0					0	0		0	No-ASE
TRIM36	5	114462214													0				0					No-ASE
COMMD10	5	115423282									0	0							0					No-ASE
SEMA6A	5	115782397				0																		No-ASE
DTWD2	5	118176773										0												No-ASE
DMXL1	5	118433785	0																	0			0	No-ASE
HSD17B4	5	118788316		0		0	0	0			0	0		0	0				0	0			0	No-ASE
FAM170A	5	118968565	0			0	0				0	1												No-ASE
PRR16	5	120021817					0																	No-ASE
SRFBP1	5	121297728		0							0								0					No-ASE
ZNF474	5	121487765					0												1	0				Heterogeneous_ASE
LOC100505841	5	121515248					0																	No-ASE
SNCAIP	5	121739518		0											0				0	0				No-ASE
PPIC	5	122359589					0																	No-ASE
CEP120	5	122682248	0			0	0							0					0		0		0	No-ASE
ZNF608	5	123977074		0		0	0				0	0		0						0	0		0	No-ASE
GRAMD3	5	125696118	0	0				0				0								0			0	No-ASE
ALDH7A1	5	125880710					0													0	0			No-ASE
PHAX	5	125939409										0												No-ASE
LMNB1	5	126145905						0			0			0										No-ASE
mrt-03	5	126213944										0												No-ASE
MEGF10	5	126667050				0	0																	No-ASE
CTXN3	5	126993249					0																	No-ASE
SLC12A2	5	127420260	0	0		0													0		0			No-ASE
FBN2	5	127595458					0							0	1								0	No-ASE
SLC27A6	5	128301211					0								1						0			Heterogeneous_ASE
ADAMTS19	5	128797271									0			1										Heterogeneous_ASE
CHSY3	5	129240667										0												No-ASE

RAPGEF6	5	130764598																		0			No-ASE
CSF2	5	131409577	0	0								0												No-ASE
SLC22A4	5	131647935	0			0	0					0									0			No-ASE
IL13	5	131994031						0				0									0			No-ASE
LEAP2	5	132209417																	0					No-ASE
FSTL4	5	132534846		1							1	0		0									1	Heterogeneous_ASE
TCF7	5	133451611				0	0							0						0			0	No-ASE
CATSPER3	5	134303694				0													0					No-ASE
PITX1	5	134364518					0				0										0			No-ASE
C5orf20	5	134782076					0																	No-ASE
SLC25A48	5	135170563																					1	ASE
IL9	5	135228153					0																	No-ASE
LECT2	5	135283122					0																	No-ASE
HNRNPA0	5	137088945										0												No-ASE
FAM13B	5	137277669																	0					No-ASE
BRD8	5	137476419						0						0						0	0			No-ASE
KIF20A	5	137515392	0																					No-ASE
CDC25C	5	137621421	0	0		1	0	0			0								1				0	No-ASE
FAM53C	5	137680573	0			0	0							0					0	0	0			No-ASE
KDM3B	5	137717265												0					0					No-ASE
HSPA9	5	137894331	0																					No-ASE
CTNNA1	5	138147906												0										No-ASE
SLC23A1	5	138707916				0													0					No-ASE
PROB1	5	138728483										0												No-ASE
TMEM173	5	138855862				0	0				0												0	No-ASE
SLC4A9	5	139739898												0										No-ASE
SRA1	5	139930307																					0	No-ASE
APBB3	5	139938237										0			0								0	No-ASE
SLC35A4	5	139946846																	0					No-ASE
TMCO6	5	140021360	0												0									No-ASE
WDR55	5	140044576		0								0			0				0					No-ASE
HARS	5	140054277					0																	No-ASE
PCDHA1	5	140166194		0				0				1		1									1	Heterogeneous_ASE
PCDHB2	5	140474467		0															1					Heterogeneous_ASE
PCDHB3	5	140480270		1			0																	Heterogeneous_ASE
PCDHB4	5	140501710		1		1	0																	Heterogeneous_ASE
PCDHB5	5	140515038													0									No-ASE
PCDHB6	5	140529841					0								0									No-ASE
PCDHB7	5	140552585	0								1													Heterogeneous_ASE
PCDHB8	5	140558064				0	0												1					Heterogeneous_ASE
PCDHB16	5	140562304				0	0							0					1	0				No-ASE
PCDHB9	5	140567264	0				0	0							0				1					No-ASE
PCDHB11	5	140579358				0	0				1													Heterogeneous_ASE
PCDHB12	5	140588502				0	0																	No-ASE
PCDHB13	5	140594389																	0					No-ASE
PCDHB14	5	140603370										0												No-ASE
SLC25A2	5	140682655						1			0	0											1	Heterogeneous_ASE
PCDHGA1	5	140710264		1		0	0	0			0	0		0	0				0	0	0		0	No-ASE
RELL2	5	141018407	0	0		0	0	0				0			0				0	0	0			No-ASE
FCHSD1	5	141021093						0			0													No-ASE
ARAP3	5	141033719					0	0			0	0			0				0		0		0	No-ASE
PCDH1	5	141233707				0									0					0	0			No-ASE
KIAA0141	5	141304176		0				0						0										No-ASE
PCDH12	5	141324954	0	0		0	0	0				0		0	0					0	0			No-ASE
NR3C1	5	142779317												0										No-ASE
HMHB1	5	143200053					0																	No-ASE
YIPF5	5	143539969									0													No-ASE
SH3RF2	5	145317535	0			0	0				0			0	0					0			0	No-ASE
PLAC8L1	5	145464015																	1				0	Heterogeneous_ASE
LARS	5	145493737	0																				0	No-ASE
GPR151	5	145894518					0				0								0	0	0			No-ASE
C5orf46	5	147281210					0																	No-ASE
SPINK5	5	147449923	0	1		1	0	0			1	1		1	0				0	0			0	Heterogeneous_ASE
FBXO38	5	147778585		0		0	0				0									0	0			No-ASE
ADRB2	5	148206440		0			0	1			0	1		0	0				0	0			1	Heterogeneous_ASE
SH3TC2	5	148384455						0				0		0					0	0				No-ASE
PCYOX1L	5	148743614	0	0			0												0				0	No-ASE
ARHGEF37	5	148977407	0	0		0		0			0	0		0	0				0	0			0	No-ASE
PPARGC1B	5	149200008				0								0	0				0					No-ASE
PDE6A	5	149240492						0																No-ASE
SLC26A2	5	149357444		0										0						0				No-ASE
TIGD6	5	149374399		0							0	0							0				0	No-ASE
HMGXB3	5	149384422		0				0			0	0		0										No-ASE
CSF1R	5	149432863		0		0																		No-ASE
PDGFRB	5	149495360																	0					No-ASE
TCOF1	5	149740732		0		0	0	0			0	0		0	0				0	0	0			No-ASE
RPS14	5	149825239	0			0		0						0	0								0	No-ASE
NDST1	5	149900889						0				0		0	0					0	0			No-ASE
IRGM	5	150226722		0																			1	Heterogeneous_ASE
TNIP1	5	150413290	0	0															0					No-ASE
CCDC69	5	150562697	0			0	0	0			0				0				0	0	0		0	No-ASE
GM2A	5	150632832		0	1	0	0				0			0	0				0				0	No-ASE
SLC36A3	5	150657130					0																	No-ASE
FAT2	5	150885263	0	1		0	0	1			0	0			0				0	0	0		0	No-ASE
SPARC	5	151045923													0									No-ASE
NMUR2	5	151771817																		1	1		1	ASE
FAM114A2	5	153372564		0		0		0				0			0					0	0		0	No-ASE
C5orf4	5	154199884						0											0					No-ASE
GEMIN5	5	154267901		0							0	0							0					No-ASE
KIF4B	5	154394302	1	1		1													1		1		1	ASE
HAVCR1	5	156476111					0				1	1					1				0			Heterogeneous_ASE
HAVCR2	5	156514129																	0					No-ASE
CYFIP2	5	156738677	0																0		0			No-ASE
NIPAL4	5	156890119	0	1		0								0										No-ASE
ADAM19	5	156908932	0	0			0				0				0				0		0		0	No-ASE
C5orf52	5	157098725	0											0					0		0			No-ASE
THG1L	5	157158612		0										0					0		0			No-ASE
RNF145	5	158630642				0								1										Heterogeneous_ASE
LOC285626	5	158759900	1	1							0										0			Heterogeneous_ASE
TTC1	5	159437621													0									No-ASE
FABP6	5	159640783	0	0		0		1			0	0								1	0		0	No-ASE
CCNJL	5	159680516	0																					No-ASE
C5orf54	5	159820751						0															0	No-ASE
SLU7	5	159832038						0											0	0			0	No-ASE
ATP10B	5	159992486		0		0					0			0					0		0		0	No-ASE
CCNG1	5	162866351																		0				No-ASE
HMMR	5	162896650						1						0						0	0		0	No-ASE
MAT2B	5	162930428						0						0							0			No-ASE
WWC1	5	167835539	0	0		0	0				0	0		0					0					No-ASE
RARS	5	167913510		0		0					0	0		0							0			No-ASE
SLIT3	5	168093556	0	1		0	0	1						0										Heterogeneous_ASE
SPDL1	5	169015433					0	0				0		0	0					0			0	No-ASE
LCP2	5	169675703					0								0									No-ASE
GABRP	5	170215647										1		0										Heterogeneous_ASE
STK10	5	171471953					0																	No-ASE
UBTD2	5	171638968									0													No-ASE
SH3PXD2B	5	171764665		0		0	0					0							0		0		0	No-ASE
DUSP1	5	172195811										0												No-ASE

CREBRF	5	172517368		0			0																	No-ASE
STC2	5	172744882												0										No-ASE
BOD1	5	173034149		0			0	0			0	0		0					0	0				No-ASE
MSX2	5	174151670					0	0				0							0					No-ASE
NOP16	5	175811233																	0					No-ASE
CDHR2	5	175992370		0								0							0	0			0	No-ASE
HK3	5	176308081												0										No-ASE
UIMC1	5	176332321										0		0										No-ASE
FGFR4	5	176516631	0	0			0							0	0				0	0	0		0	No-ASE
NSD1	5	176562176				0					0												0	No-ASE
MXD3	5	176734179																		0				No-ASE
LMAN2	5	176759170		0															0					No-ASE
SLC34A1	5	176812770					0																	No-ASE
DBN1	5	176885145										0		0					0	0	0			No-ASE
PDLIM7	5	176911101										0		0									1	Heterogeneous_ASE
DOK3	5	176930113										0		0					0	0			1	No-ASE
TMED9	5	177019262		0	0									0							0		0	No-ASE
AGXT2L2	5	177638957																	0					No-ASE
COL23A1	5	177669056					0																	No-ASE
ZNF454	5	178373941					0																	No-ASE
GRM6	5	178408668					1																	ASE
ZNF879	5	178455114	1																		0			Heterogeneous_ASE
ZNF354C	5	178503473	1	1																			1	ASE
ADAMTS2	5	178540953					0																	No-ASE
MAML1	5	179192418		0		0						0		0	0				0				0	No-ASE
MGAT4B	5	179225386				0													0					No-ASE
SQSTM1	5	179248034	0	0							0	0			0					0	0			No-ASE
C5orf45	5	179267889	0			0	0				0				0						0		0	No-ASE
TBC1D9B	5	179290477	0	1		0	0				0	0		0					0	0	0			No-ASE
CNOT6	5	179956302																	0					No-ASE
FLT4	5	180030221				1																		ASE
MGAT1	5	180218668	0	0							0	0								0				No-ASE
ZFP62	5	180275896	0									0								0				No-ASE
BTNL8	5	180326326	1	1		0					0				0				0	0				Heterogeneous_ASE
OR2V2	5	180582003					0																	No-ASE
TRIM7	5	180622167		0								0		0										No-ASE
TRIM41	5	180651231				0																		No-ASE
EXOC2	6	491150													0						0			No-ASE
MYLK4	6	2675282			0									0	0									No-ASE
WRNIP1	6	2765899										0											0	No-ASE
SERPINB6	6	2948627		0	0	0						0			0								0	No-ASE
NQO2	6	3010298				0							0		0					0	0		0	No-ASE
BPHL	6	3123967																	0					No-ASE
PSMG4	6	3259296										0			0									No-ASE
PXDC1	6	3727811	0									0			0				0				0	No-ASE
PRPF4B	6	4031998	0	0											0				0					No-ASE
FAM217A	6	4068932	0											0										No-ASE
C6orf201	6	4087904	0	0	0	0								0					0					No-ASE
ECI2	6	4133855	0	0	0	0																		No-ASE
RPP40	6	4995464													0					0				No-ASE
LYRM4	6	5260936	0	0		0																	0	No-ASE
FARS2	6	5368973		0											0					0			0	No-ASE
F13A1	6	6145863			0																			No-ASE
RREB1	6	7182155	0	0						0		0		0	0								0	No-ASE
SSR1	6	7299004				0						0	0	0							0		0	No-ASE
RIOK1	6	7395322	0																					No-ASE
DSP	6	7542160	0		0								0						0		0			No-ASE
SNRNP48	6	7590624		0	0					0		0		0	0					0				No-ASE
TMEM14B	6	10747996																	0					No-ASE
SYCP2L	6	10894336	0									1												Heterogeneous_ASE
SMIM13	6	11103937													0									No-ASE
NEDD9	6	11185782										0												No-ASE
ADTRP	6	11723611																		0				No-ASE
HIVEP1	6	12120290	0	0								0	0		0				0		0		0	No-ASE
EDN1	6	12290866												0	0				0					No-ASE
SIRT5	6	13584347								0			0											No-ASE
CCDC90A	6	13801615								0			0		0									No-ASE
DTNBP1	6	15523376	0			0																		No-ASE
MYLIP	6	16129603				0				0		0								0			0	No-ASE
GMPR	6	16247181	0		0	0				0		0	0		0								0	No-ASE
CAP2	6	17421824																					0	No-ASE
NUP153	6	17616405			0	0				0		0	0	0	0								0	No-ASE
KIF13A	6	17764360		0										0										No-ASE
TPMT	6	18130918													0								0	No-ASE
DCDC2	6	24175021	1	1		0							1											ASE
MRS2	6	24403275	0		0					0				0	0					0				No-ASE
GPLD1	6	24429297	0	0	0	0						0	1						0		0		0	No-ASE
ALDH5A1	6	24495234	0			0				0			1		0				0	0	0		0	No-ASE
KIAA0319	6	24544967	0			0				1		0	1		0				0	0	0		0	No-ASE
TDP2	6	24651032											0											No-ASE
GMNN	6	24777517																	0					No-ASE
LRRC16A	6	25426768				0						0	0	0					0		0			No-ASE
SLC17A4	6	25762227	0		0							0	0	0	0						0			No-ASE
HIST1H1A	6	26017510			0							0	0	0					0					No-ASE
HIST1H1C	6	26056053													0									No-ASE
HFE	6	26087686																		0			0	No-ASE
HIST1H1T	6	26107774	0	0		0						0	0	0	0						0		0	No-ASE
HIST1H1E	6	26156752				0																		No-ASE
BTN3A2	6	26370616			0	0				0			0		0									No-ASE
BTN2A2	6	26384060			0	0							0								0			No-ASE
BTN3A1	6	26405816			0					0			0		0									No-ASE
BTN3A3	6	26446066																	0					No-ASE
BTN2A1	6	26459861			0	0							0											No-ASE
BTN1A1	6	26502012		0	1	0				0			1											Heterogeneous_ASE
HMGN4	6	26545532																	0					No-ASE
POM121L2	6	27276861				1																		ASE
ZNF184	6	27419389	0		0	0							0	0	0					0			0	No-ASE
HIST1H2BL	6	27775647																			0		0	No-ASE
HIST1H1B	6	27834677																			0			No-ASE
HIST1H4L	6	27840926										0	1										0	Heterogeneous_ASE
ZKSCAN8	6	28116310										0	0											No-ASE
ZKSCAN4	6	28213031				0							0											No-ASE
NKAPL	6	28227156				1																		ASE
ZSCAN26	6	28239741				0							0											No-ASE
PGBD1	6	28251610	0			1						0	0								0			No-ASE
ZSCAN31	6	28293992	0			0						0	0							0	0			No-ASE
ZKSCAN3	6	28327371	0			0						0								0	0			No-ASE
ZSCAN12	6	28358320	1									1	1											ASE
ZNF311	6	28962827																	0					No-ASE
LOC100129636	6	29012067		1																				ASE
OR2J3	6	29079871		1								0												Heterogeneous_ASE
OR14J1	6	29274486										0												No-ASE
OR12D2	6	29364508										0												No-ASE
OR10C1	6	29407800										0												No-ASE
UBD	6	29523670				0				0		0		0							0			No-ASE
GABBR1	6	29570841	0	0						0					0									No-ASE
MOG	6	29625001											1											ASE
ZFP57	6	29640284																	1					ASE

HLA-F	6	29691278								0					0									No-ASE
HLA-F-AS1	6	29696245								0					0									No-ASE
HCG4	6	29759876		1						1		1	1		1									ASE
HLA-H	6	29855978										0												No-ASE
HLA-A	6	29910750	0	0						0			0	0										No-ASE
HCG9	6	29943035	0	0								0			0						0			No-ASE
ZNRD1-AS1	6	30002793	0							0		0							0					No-ASE
ZNRD1	6	30029109										0												No-ASE
RNF39	6	30038352								0		0	0						0					No-ASE
TRIM31	6	30071110	0	0		0				0		0	0	0	0				0		0			No-ASE
TRIM40	6	30104982	0											0	0									No-ASE
TRIM10	6	30120563		0		0						0		0	0				0		0			No-ASE
TRIM15	6	30131546	0	0		0						0	0	0	0									No-ASE
TRIM26	6	30152961		0		0						0												No-ASE
HCG17	6	30231224		0		0						0			0				0		0			No-ASE
TRIM39	6	30297126				0				0		0			0				0		0			No-ASE
TRIM39-RPP21	6	30312958	0	0						0		0	0	0	0						0			No-ASE
HLA-E	6	30457671				0				0					0						0			No-ASE
GNL1	6	30511170				0				0		0			0					0	0			No-ASE
PRR3	6	30529622				0				0		0		0	0					0	0			No-ASE
ATAT1	6	30595648	0			0				0		0			0						0			No-ASE
PPP1R18	6	30645050				0								0					0	0	0			No-ASE
NRM	6	30656463				0																		No-ASE
MDC1	6	30668246		0		0				0		0		0	0				0	0	0			No-ASE
IER3	6	30711031				0				0				0	0					0				No-ASE
DDR1	6	30856555	0	0		0				0														No-ASE
VARS2	6	30882636	0	0		0				0		0			0									No-ASE
SFTA2	6	30899524	0	0						0														No-ASE
DPCR1	6	30916645	0	0		0						0			0									No-ASE
MUC21	6	30951739																			0			No-ASE
MUC22	6	30993377	0	0								1			0							0		No-ASE
C6orf15	6	31079264	0	1	0										1				1	0	0			Heterogeneous_ASE
PSORS1C1	6	31082960	0	0	0	0				0				0	0				0	0	0			No-ASE
CCHCR1	6	31110391	0	0	0	0				0				0	0				0	0	0			No-ASE
TCF19	6	31127322	0	0	0	0				0				0	0				0	0	0			No-ASE
PSORS1C3	6	31141523	0		1	0								1										Heterogeneous_ASE
HCG27	6	31165566			0	0								0	0				0		0			No-ASE
HLA-C	6	31237124	0	0	0	0				0		0		1	0				0	0	0			No-ASE
HLA-B	6	31321685		0	0	0				0				0					0	0	0		1	No-ASE
MICA	6	31378335		0		0				0		0		0	0					0	0		0	No-ASE
HCP5	6	31431691	0	0	0	0				0		0		0	0				0		0			No-ASE
HCG26	6	31440082	0			0													1					Heterogeneous_ASE
MICB	6	31473546		0		0						0		0			1				0		0	No-ASE
MCCD1	6	31496834		0		0					0	0									0			No-ASE
DDX39B	6	31506624				0					0	0			0						0			No-ASE
ATP6V1G2-DDX39B	6	31512720	0												0									No-ASE
NFKBIL1	6	31525912			0								0											No-ASE
AIF1	6	31583111		1						1	1									0			1	ASE
PRRC2A	6	31592275	0	0	0	0				0	0		0							0	0		0	No-ASE
BAG6	6	31608031	0	0							0													No-ASE
C6orf47	6	31626904		0		0				0	0									0	0		0	No-ASE
GPANK1	6	31629638	0	0	0	0					0	0									0		0	No-ASE
CSNK2B	6	31635710	0	0	0	0					0										0		0	No-ASE
LY6G5B	6	31638728	0		0																0			No-ASE
LY6G6F	6	31675283	0		0	0					0			0							0		1	No-ASE
LY6G6C	6	31686497												0	0									No-ASE
C6orf25	6	31691508	0			0						0									0			No-ASE
CLIC1	6	31698714	0		0																		0	No-ASE
MSH5-SAPCD1	6	31707730		0		0						0		0	0				0		0			No-ASE
VWA7	6	31733418			0						0													No-ASE
VARS	6	31746948		0	0			0				0		0	0									No-ASE
HSPA1L	6	31777859	0			0				0	0	0		0	0					0				No-ASE
HSPA1A	6	31783507	0	0		0		0			0	0		0	0								1	No-ASE
HSPA1B	6	31795550	0			0																		No-ASE
C6orf48	6	31807343			0			0																No-ASE
NEU1	6	31827286	0																					No-ASE
SLC44A4	6	31831478		0	0	0					0	0			0					0				No-ASE
C2	6	31868275		0		0								0	0						0			No-ASE
CFB	6	31914179									0								0		0		0	No-ASE
SKIV2L	6	31927839			0	0		0		0											0		0	No-ASE
DOM3Z	6	31937715	0	0		0						0		0	0									No-ASE
TNXB	6	32011639		0	0	0		0		0	0			0	0				1				1	No-ASE
ATF6B	6	32083175	0	0				0		0		0		0									0	No-ASE
FKBPL	6	32097091	0					0				0		0					0				0	No-ASE
LOC100507547	6	32120715						0																No-ASE
PPT2	6	32122386	0		0	0		0				0	0	0					0					No-ASE
PPT2-EGFL8	6	32133976	0		0			0				0	0	0										No-ASE
AGPAT1	6	32136547		0	0			0					0	0										No-ASE
RNF5	6	32147691	0		0			0				0	0	0					0					No-ASE
AGER	6	32149140	0																					No-ASE
NOTCH4	6	32163558		0										0	1									Heterogeneous_ASE
BTNL2	6	32362521										1												ASE
HLA-DRA	6	32407740	0																					No-ASE
HLA-DRB5	6	32487242				0							1											Heterogeneous_ASE
HLA-DRB1	6	32546879	1	0								1	1	0										Heterogeneous_ASE
HLA-DQA1	6	32605284			1			1			0		1						1					ASE
HLA-DQB1	6	32629137	1		1	0		1			1	0	1	1					1	1			1	ASE
HLA-DQA2	6	32712977						1											1					ASE
HLA-DQB2	6	32726693												1										ASE
HLA-DOB	6	32780724		0	0	0		1			0			0	1				0	0				No-ASE
TAP2	6	32790059	0		0			0		0	0		0	0	0				0	0	0		0	No-ASE
PSMB8	6	32809349												0	0					0	0			No-ASE
LOC100507463	6	32813214	0		0	0							0	0									0	No-ASE
TAP1	6	32814909	0		0									0					0	0			0	No-ASE
PSMB9	6	32823948	0		0					0														No-ASE
LOC100294145	6	32862682	0							0				0					0	0	0		0	No-ASE
HLA-DMB	6	32902583	0	0				0		0	0			0						0	0			No-ASE
HLA-DMA	6	32917165		0				0				0	0	0	0				0	0				No-ASE
BRD2	6	32939785	0	0	0			0		0		0	0	0	0					0	0			No-ASE
HLA-DOA	6	32972404				1						0		0	1									Heterogeneous_ASE
HLA-DPA1	6	33033022		0							0			1	0									No-ASE
HLA-DPB1	6	33048628	0	0							0		1	1	0									Heterogeneous_ASE
HLA-DPB2	6	33080359												1										ASE
COL11A2	6	33131501			0																			No-ASE
RXRB	6	33162082																	0		0			No-ASE
SLC39A7	6	33168716			0						0													No-ASE
RING1	6	33179062																			0			No-ASE
HCG25	6	33218180						0													0			No-ASE
VPS52	6	33231323		0	0	0				0			0	0	0				0					No-ASE
WDR46	6	33247085			0			0					0	0							0			No-ASE
RGL2	6	33260036											0											No-ASE
TAPBP	6	33267528		0		0				0					0									No-ASE
ZBTB22	6	33282824		0		0				0		0			0									No-ASE
KIFC1	6	33359716		0	0	0		0		0	0				0						0			No-ASE
PHF1	6	33380066			0									0										No-ASE
CUTA	6	33384473	0					0				0	0								0			No-ASE
SYNGAP1	6	33405980								0														No-ASE
ITPR3	6	33589404	0		0			0		0	0	0	0	0	0					0				No-ASE

C6orf1	6	34214322	0																0	0	0		0	No-ASE
UHRF1BP1	6	34802110	0	0	0			0		0					0					0			0	No-ASE
TAF11	6	34847785		0																				No-ASE
ANKS1A	6	34949644											0	0										No-ASE
SCUBE3	6	35201057		0																				No-ASE
ZNF76	6	35248983	0					0											0					No-ASE
DEF6	6	35278309																					0	No-ASE
FANCE	6	35423528	0											0					0					No-ASE
TEAD3	6	35442397	0																0		0			No-ASE
FKBP5	6	35544889																			0			No-ASE
CLPSL1	6	35748931		0																				No-ASE
SRPK1	6	35803103			0										0									No-ASE
SLC26A8	6	35911696	0	0	0			0		0		0	0	0	0				0				0	No-ASE
BRPF3	6	36168415	0	0	0					0		0	0		0					0	0			No-ASE
PNPLA1	6	36259274	0		0			0					0		0				0	0	0		0	No-ASE
C6orf222	6	36287134			0			0		0				0	0						0		0	No-ASE
ETV7	6	36334748						0					0						0				0	No-ASE
KCTD20	6	36442567	0		0							0	0	0										No-ASE
CDKN1A	6	36651889																	0					No-ASE
PPIL1	6	36823754										0			0								0	No-ASE
C6orf89	6	36867372										0	0	0										No-ASE
PI16	6	36922621						0																No-ASE
PIM1	6	37138904		0																			0	No-ASE
MDGA1	6	37605100																			0			No-ASE
GLO1	6	38649871		0	0	0				0		0	0		0						0		0	No-ASE
DNAH8	6	38702283							1															ASE
GLP1R	6	39024212										0												No-ASE
KCNK16	6	39282806		1																				ASE
MOCS1	6	39876849												0	0									No-ASE
LRFN2	6	40359723													0									No-ASE
UNC5CL	6	40996173	0		0															0			0	No-ASE
APOBEC2	6	41021108				0					0	0		0										No-ASE
TREML2	6	41160572												0						1		0		Heterogeneous_ASE
TREM1	6	41243926									0													No-ASE
NCR2	6	41303831		0											0									No-ASE
USP49	6	41766425										0												No-ASE
BYSL	6	41889323			0															0				No-ASE
C6orf132	6	42072628		0	0			0		0	0		0	0	0						0	0	0	No-ASE
TRERF1	6	42196094	0			0							0							0		0		No-ASE
UBR2	6	42559926		0				0		0	0	0	0	0					0	0		0		No-ASE
PRPH2	6	42666061		0											0				0				1	No-ASE
CNPY3	6	42903349								0			0	0								0		No-ASE
PEX6	6	42932080	1	1	0			0	0	0	1		0	0						0	1	0		Heterogeneous_ASE
MEA1	6	42980218																					0	No-ASE
RRP36	6	42989417						0	0											0		0		No-ASE
CUL7	6	43005443				0		0						0								0		No-ASE
KLC4	6	43029099						0	0													0		No-ASE
PTK7	6	43097978				0		0	0		0			0								1	0	No-ASE
CUL9	6	43152127						0	0		0	0			0				0			0		No-ASE
TTBK1	6	43221052																		0			1	Heterogeneous_ASE
CRIP3	6	43273604						0		1	0	0		0							0	1	1	Heterogeneous_ASE
ZNF318	6	43304912												0					0			0		No-ASE
ABCC10	6	43400202			0	0			0			0	0	0						0			0	No-ASE
DLK2	6	43418453		0				0																No-ASE
YIPF3	6	43479934												0										No-ASE
XPO5	6	43492270						0				0										0		No-ASE
C6orf223	6	43968412						1					0											Heterogeneous_ASE
TMEM63B	6	44103069											0									0		No-ASE
CAPN11	6	44134522	0	0					1															Heterogeneous_ASE
SLC29A1	6	44197398																			0			No-ASE
HSP90AB1	6	44216399						1						1									1	ASE
NFKBIE	6	44227802							0															No-ASE
TCTE1	6	44248003	0								0		1	0						0				No-ASE
SUPT3H	6	44900431								0														No-ASE
CLIC5	6	45870832												0	0					0				No-ASE
ENPP4	6	46107343	0	0	0			0	0		0		0						0		0		0	No-ASE
ENPP5	6	46128688	0	0	0			0	0				0						0	0	0		0	No-ASE
CYP39A1	6	46521532				0						0		0					0	0	0		0	No-ASE
SLC25A27	6	46623690		0																	0			No-ASE
TDRD6	6	46655876																		0	0			No-ASE
PLA2G7	6	46672301				0											1				0			Heterogeneous_ASE
MEP1A	6	46761313		1	0			0				0	0	0	0					0			0	No-ASE
GPR110	6	46975021			0														0					No-ASE
GPR111	6	47646842	0	0					1				1		0				0		0			Heterogeneous_ASE
GPR115	6	47675014		0		0			0			0			0				0				1	No-ASE
MUT	6	49403267	0	0	0				0	0		0	0		0				0	0	0	0		No-ASE
CENPQ	6	49437923	0	0	0				0	0			0		0				0	0	0	0	0	No-ASE
PKHD1	6	51483961	0		0	0				0	0	1		0					0	0	0	0	0	No-ASE
MCM3	6	52129484		0		0						0		0									0	No-ASE
EFHC1	6	52285209	0	0		0									0					0	0			No-ASE
ICK	6	52871155				0						0										0		No-ASE
GCM1	6	52993209																	0					No-ASE
KLHL31	6	53516464		0	0			0													0	0		No-ASE
LRRC1	6	53660101	0																					No-ASE
MLIP	6	53883843																	1					ASE
TINAG	6	54173355	0		0	1			0				0						0	0			0	No-ASE
FAM83B	6	54735051	0	0								0	0	0						0				No-ASE
HCRTR2	6	55039416																				1		ASE
COL21A1	6	55923968												0										No-ASE
RNU6-71	6	56323828	0	0		0		0	0		0	0	0	0	0				0	0	0	0	0	No-ASE
KIAA1586	6	56917508		0		0		0		0		0		0	0						0		0	No-ASE
PRIM2	6	57185345												1	1									ASE
PHF3	6	64389915												0						0				No-ASE
EYS	6	64431505															0		1		0			Heterogeneous_ASE
BAI3	6	69348700										1			0									Heterogeneous_ASE
LMBRD1	6	70386413	0		0	0		0		0		0	0		0		0			0	0		0	No-ASE
FAM135A	6	71162209			0							0	0	0			0			0				No-ASE
C6orf57	6	71276670			0			0	0	0		0					0				0		0	No-ASE
SMAP1	6	71377781				0								1										Heterogeneous_ASE
KHDC1	6	73951280									1													ASE
DDX43	6	74104285						0	1	1														Heterogeneous_ASE
MB21D1	6	74135071						0		1							0				0		1	Heterogeneous_ASE
CD109	6	74407128	0		1					0		0	0		0						1		0	No-ASE
COL12A1	6	75797302	0									0					0							No-ASE
SENP6	6	76331308		0		0				0			0		0		0			0			0	No-ASE
MYO6	6	76527290																	0	0				No-ASE
IMPG1	6	76631823	0		0					0	0	0		0							0			No-ASE
HTR1B	6	78172085													1									ASE
PHIP	6	79655736	0		0			0	0			0		0	0		0			0	0		0	No-ASE
LCA5	6	80196789	0		0	0		0		0		0		0						0				No-ASE
ELOVL4	6	80624530	0									0												No-ASE
TTK	6	80717615																					0	No-ASE
IBTK	6	82882185	0	0	0					0		0		0	0						0		0	No-ASE
UBE3D	6	83667045										0	0											No-ASE
DOPEY1	6	83810533			0							0	0				0							No-ASE
PGM3	6	83878970		0	0	0				0		0	0				0			0				No-ASE
ME1	6	83933509		0																				No-ASE
PRSS35	6	84233570	0					0																No-ASE

NT5E	6	86159933							0					0	0				0	0			1	No-ASE
ZNF292	6	87953322	0		0	0				0			0	0			0		0	0	0		0	No-ASE
GJB7	6	87994015	0	0	0	0							0		0		1				0		1	No-ASE
C6orf165	6	88119600												0										No-ASE
RARS2	6	88224688		0		0								0										No-ASE
ORC3	6	88304122		0											0									No-ASE
AKIRIN2	6	88385579																	0					No-ASE
GABBR1	6	89888668								0							1							Heterogeneous_ASE
GABBR2	6	89967411				1		0		0		0	0		0		0							No-ASE
UBE2J1	6	90039532	0	0		0											0				0		0	No-ASE
ANKRD6	6	90276733										0							0					No-ASE
LYRM2	6	90347511		0		0			0	0			0	0			0			0				No-ASE
MDN1	6	90354735		0	0	0		0	0	0	0	0	0	0	0		0		0	0			0	No-ASE
BACH2	6	90641851															0							No-ASE
MANEA	6	96034328	0																					No-ASE
FHL5	6	97051559								0														No-ASE
NDUFAF4	6	97339088	0		0										0					0	1			No-ASE
KLHL32	6	97414949										0	0											No-ASE
MIR548H3	6	97597803	0	1	0	0			0	0			0		0		0			0	0			No-ASE
FBXL4	6	99323399										0			0		0							No-ASE
COQ3	6	99817491	0						0				0	0	0		0		0		0			No-ASE
PNISR	6	99848453																					0	No-ASE
USP45	6	99883704	0		0	0				0		0	0		0				0	0	0		0	No-ASE
ASCC3	6	100957344	0	1	0			0		0		0	0										0	No-ASE
GRIK2	6	102134167											0											No-ASE
BVES-AS1	6	105606361		1													1							ASE
PREP	6	105726036	0					0				0					0		0					No-ASE
PRDM1	6	106536253			0				0			0			0					0				No-ASE
AIM1	6	106960245	0					0				0	0	0			0		0		0		0	No-ASE
QRSL1	6	107088231				0								0					0		0			No-ASE
OSTM1	6	108370517																	0					No-ASE
LACE1	6	108616303							0															No-ASE
ARMC2	6	109175625																	0					No-ASE
SESN1	6	109313998	0									0	0				0			0				No-ASE
CEP57L1	6	109466452										0												No-ASE
PPIL6	6	109721368				0		0					0		0									No-ASE
SMPD2	6	109763433	0		0				0						0					0	0		0	No-ASE
MICAL1	6	109765453	0		0				0			0			0					0	0		0	No-ASE
ZBTB24	6	109783941	0		0	0			0						0					0	0		0	No-ASE
AKD1	6	109819135	0		0	0		0					0		0						0		0	No-ASE
FIG4	6	110037655				0			0						0						0			No-ASE
WASF1	6	110421361																	0					No-ASE
CDC40	6	110501666																			0			No-ASE
SLC22A16	6	110746146	0		0										0									No-ASE
KIAA1919	6	111583554																	0					No-ASE
REV3L	6	111628626	0					0	0	0		0					0			0			0	No-ASE
TRAF3IP2-AS1	6	111880723			0	0		0					0		0		0			0				No-ASE
FYN	6	111983038								0					0		0				0			No-ASE
WISP3	6	112375532															1				0			Heterogeneous_ASE
TUBE1	6	112393155																	0					No-ASE
LAMA4	6	112430662						0	0		0	0	1	0	0				1		1		1	Heterogeneous_ASE
FRK	6	116263644				0		0		0			0	0	0		0			0	0		0	No-ASE
NT5DC1	6	116429582						0					0	0					0	0	0		0	No-ASE
TSPYL4	6	116573958			0	0		0				0		0			0			1			0	No-ASE
TSPYL1	6	116599859				0				0									0		0			No-ASE
DSE	6	116720448										0		0	0				0					No-ASE
BET3L	6	116818128	0		0																			No-ASE
RSPH4A	6	116937877												0										No-ASE
ZUFSP	6	116967057						0		0				0					0					No-ASE
FAM162B	6	117083172													1									ASE
GPRC6A	6	117113315								1					0									Heterogeneous_ASE
ROS1	6	117609683								0		1												Heterogeneous_ASE
DCBLD1	6	117825028	0																0					No-ASE
CEP85L	6	118786639			0	0			0		0		0		0		0			1	1		0	No-ASE
MCM9	6	119136133			0			0		0		0					0						0	No-ASE
FAM184A	6	119288065	0																				0	No-ASE
C6orf170	6	121401942	0			0		0	0	0		0		0			0		0		0			No-ASE
SERINC1	6	122766138	0						0			0								0	0		0	No-ASE
SMPDL3A	6	123110537			0			0						0									0	No-ASE
RNF217	6	125379112				0													0					No-ASE
NCOA7	6	126136540			0	0		0		0		0		0	0		0		0				0	No-ASE
HINT3	6	126278230	0		0	0			0			0	0	0	0				0				0	No-ASE
TRMT11	6	126307709			0															0			0	No-ASE
CENPW	6	126661502	0		0																		0	No-ASE
SOGA3	6	127765277			0				1			0									0			No-ASE
LAMA2	6	129371201	0			0									0				1	0			0	No-ASE
ARHGAP18	6	129905211	0							0									0		0			No-ASE
TMEM244	6	130152520										1												ASE
L3MBTL3	6	130370485	0						0	0		0		0										No-ASE
SAMD3	6	130465847												0										No-ASE
SMLR1	6	131148737				0			0	0			0	0					0					No-ASE
AKAP7	6	131466537	0		0	0		0	0					0	0									No-ASE
ENPP1	6	132172368						0					0							0				No-ASE
VNN1	6	133004281		0						0														No-ASE
SLC18B1	6	133091438						0																No-ASE
EYA4	6	133789728		1								1												ASE
TBPL1	6	134303785		0																				No-ASE
HBS1L	6	135286230			0							0	0	0					0	0				No-ASE
AHI1	6	135606565		0										0	0				0					No-ASE
BCLAF1	6	136582458				1	1	1	1		1						1	1						ASE
MAP7	6	136667027										0	0											No-ASE
MAP3K5	6	136882715																		0				No-ASE
SLC35D3	6	137243720													0									No-ASE
IL20RA	6	137322820		0				0				0			0								0	No-ASE
TNFAIP3	6	138192646										0							0					No-ASE
PERP	6	138413186	0							0	0	0	0	0							0		0	No-ASE
KIAA1244	6	138491807										0		0					0	0				No-ASE
NHSL1	6	138751629																		0				No-ASE
CCDC28A	6	139094890								0			0	0										No-ASE
ECT2L	6	139134423	0									0		0	0									No-ASE
REPS1	6	139229814		0	0	0				0				0	0					0				No-ASE
GPR126	6	142630763				0		0	0	0					0									No-ASE
HIVEP2	6	143074516	0					0		0				0									0	No-ASE
FUCA2	6	143816863	0	0		0		0	0			0	0		0				0		0			No-ASE
PHACTR2	6	143929450		0																	0			No-ASE
STX11	6	144507790						0																No-ASE
UTRN	6	144743015	0		0				0			0	0	0	0				0	0			0	No-ASE
LOC100507557	6	146121438								0			0	0	0				0	0				No-ASE
STXBP5	6	147556386			0	0						0	0	0	0				0					No-ASE
SASH1	6	148711301						0	0	0			0						0	0				No-ASE
TAB2	6	149699523	0	0		0				0		0	0	0						0			0	No-ASE
ZC3H12D	6	149771932		0		0																		No-ASE
GINM1	6	149893443								0														No-ASE
PCMT1	6	150071059						0					0											No-ASE
LRP11	6	150141767	0		0			0		0			0		0				0					No-ASE
LOC100652739	6	150209717	1	0	1			0		0		0	0	0	0				0	0				No-ASE
IYD	6	150710490				0															0			No-ASE
PLEKHG1	6	151055031		0	0			0						0					0	0				No-ASE

AKAP12	6	151626933	0	0		0		0	0	0	0						0		0		0		0	No-ASE
ZBTB2	6	151686905	0			0			0	0							0		0					No-ASE
C6orf211	6	151789596		0		0				0		0	0								0			No-ASE
CCDC170	6	151857472		1	0	0			1					0	0					1	0		0	Heterogeneous_ASE
SYNE1	6	152443693	0	1		0						0		0	0		0		0				0	No-ASE
FBXO5	6	153292308							0			0	0				0			0				No-ASE
OPRM1	6	154360642			1												0							Heterogeneous_ASE
CNKSR3	6	154727538															0							No-ASE
SCAF8	6	155109136						0													0			No-ASE
TIAM2	6	155450378				0				0				0	0				0	0				No-ASE
TFB1M	6	155579035				0			0	0		0	0	0	0				0					No-ASE
TMEM242	6	157739931																					0	No-ASE
SYNJ2	6	158438287	1							0		0		0	0				0	0				No-ASE
SERAC1	6	158532382																	0					No-ASE
TULP4	6	158735296			0								0	0									0	No-ASE
TMEM181	6	158957764								0									0					No-ASE
SYTL3	6	159084320			0			0	0	0					0				0		0			No-ASE
RSPH3	6	159398620			0			0					0										1	No-ASE
TAGAP	6	159457034			0																			No-ASE
SOD2	6	160106042			0				0	0			0	0	0		0		0		0		0	No-ASE
ACAT2	6	160183960			0				0				0	0	0		0		0	0	0		0	No-ASE
MRPL18	6	160211648						0	0				0		0		0		0		0			No-ASE
IGF2R	6	160412313						0				0		0					0	0	0			No-ASE
SLC22A1	6	160543008			0			0	0			0		0	0		0		0	0	0		1	No-ASE
SLC22A3	6	160819086		0	0								0				0						1	No-ASE
MAP3K4	6	161455328								0							0		0		0		0	No-ASE
PARK2	6	161771240									0	0	1		0					0				No-ASE
T	6	166571840										1												ASE
MPC1	6	166778930													0									No-ASE
RPS6KA2	6	166836744	0		0							0			0				1		0		0	No-ASE
RNASET2	6	167343106									0								0				0	No-ASE
UNC93A	6	167704994		1				1	0	0	0			0	0				0				0	No-ASE
TTLL2	6	167738668	0	0	1			0							0		0		0	1				No-ASE
KIF25	6	168431475																			0			No-ASE
FRMD1	6	168457824								1					0									Heterogeneous_ASE
DACT2	6	168708369										1					0		0					Heterogeneous_ASE
WDR27	6	169982991	0						0	0				0			0		0	0			0	No-ASE
C6orf70	6	170151721	0							0				0	0					0			0	No-ASE
FAM120B	6	170627191			0												0							No-ASE
PSMB1	6	170844432	0	0	0				0	0	0	0			0		0			0	0			No-ASE
FAM20C	7	193245				1					0	1						0					0	Heterogeneous_ASE
HEATR2	7	769344	0	0	0		0	0		0	0	0	0	0	0								0	No-ASE
SUN1	7	872190				0				0	0	0	0		0			0					0	No-ASE
ADAP1	7	938848	0							0								0				0		No-ASE
CYP2W1	7	1022990		0				0																No-ASE
C7orf50	7	1040173	0	0	0			0				0	0									0		No-ASE
MICALL2	7	1474260																		0				No-ASE
INTS1	7	1511214											0											No-ASE
TMEM184A	7	1586617								0			0											No-ASE
MAD1L1	7	1937837	0			0	0	0		0												0		No-ASE
EIF3B	7	2395043			0																			No-ASE
CHST12	7	2472429		0									0											No-ASE
LFNG	7	2552806		0																				No-ASE
BRAT1	7	2577871			0					0														No-ASE
IQCE	7	2606797	0		0	0		0			0	0	0	0				0		0		0	0	No-ASE
SDK1	7	3658736	0							0		0		0				0					1	No-ASE
AP5Z1	7	4820915	0					0					0											No-ASE
RADIL	7	4839025						1										0						Heterogeneous_ASE
RBAK	7	5103404		0			0	0		0	0	0	0	0	0			0	0				0	No-ASE
RBAK-LOC389458	7	5112554																0						No-ASE
TNRC18	7	5353449		0	0	0					0			0	0							0	0	No-ASE
FBXL18	7	5521534	0																					No-ASE
PMS2	7	6026457									0													No-ASE
EIF2AK1	7	6064327				0																		No-ASE
USP42	7	6154984																0					0	No-ASE
FAM220A	7	6370025	0	0							0	0	0					0	0			0		No-ASE
DAGLB	7	6449476		0	0			0		0													0	No-ASE
GRID2IP	7	6547902																					1	ASE
ZDHHC4	7	6621269	0	0		0		0					0	0								0	0	No-ASE
ZNF853	7	6656830																0		0			0	No-ASE
ZNF12	7	6730487		0				0							0									No-ASE
C1GALT1	7	7274054		0																				No-ASE
COL28A1	7	7398301								0			0											No-ASE
PHF14	7	11022095	0	0				0			0	0		0	0						0	0		No-ASE
THSD7A	7	11418786			0						1	0		0	0			0				0	1	No-ASE
TMEM106B	7	12254552						0																No-ASE
VWDE	7	12373125	0		1	0					1	0			1			0					0	Heterogeneous_ASE
SCIN	7	12610578	0		0		1	0		0	1	1										0		Heterogeneous_ASE
ETV1	7	13978809										0		0									0	No-ASE
SOSTDC1	7	16502264			1																			ASE
ANKMY2	7	16640445			0					0			0											No-ASE
AHR	7	17349691			0			0		0													0	No-ASE
PRPS1L1	7	18066561	1	1		1		1		1	1		1					1				1		ASE
HDAC9	7	18535940									0			0					0					No-ASE
MACC1	7	20180717	0	0	0	0		0		0		0	0					0				0	0	No-ASE
ABCB5	7	20666191																0						No-ASE
DNAH11	7	21584693	0	1	1	0	0	1		1	0	1	0	0	0				1			0		Heterogeneous_ASE
RAPGEF5	7	22165242											0											No-ASE
STEAP1B	7	22478374											1											ASE
NUPL2	7	23221811										0												No-ASE
GPNMB	7	23286486			1																			ASE
IGF2BP3	7	23352431								0														No-ASE
FAM221A	7	23724197																					0	No-ASE
STK31	7	23749905	0	0	0			1		0	0	0	0					0				0		No-ASE
DFNA5	7	24738802		0		0				0														No-ASE
C7orf31	7	25175698	0	0		0		0		0	0	0	0	0				0		0				No-ASE
SKAP2	7	26709790			0			0				0		0										No-ASE
HOXA1	7	27134966										0	0									1		Heterogeneous_ASE
HOXA3	7	27147739						0		0		0												No-ASE
HOXA7	7	27195857				1		0				0	0						0					No-ASE
TAX1BP1	7	27797660	0		0					0													0	No-ASE
JAZF1	7	27872466	0																					No-ASE
TRIL	7	28995807						0										0						No-ASE
LOC100506497	7	29035428											0											No-ASE
CPVL	7	29070209	0	0	0	1				0	0	1			0								1	Heterogeneous_ASE
CHN2	7	29234574						0			0				0									No-ASE
PRR15	7	29606160		0		0					0			0	0									No-ASE
WIPF3	7	29918676						1																ASE
SCRN1	7	29966085		0	0						0													No-ASE
NOD1	7	30465283	0							0					0							0		No-ASE
GARS	7	30634548				0									0									No-ASE
INMT	7	30791800		1	1	0							0		0			0				0	0	No-ASE
INMT-FAM188B	7	30818064		0		0				0		0			0			0					0	No-ASE
AQP1	7	30951658						0																No-ASE
CCDC129	7	31609381				0				0					0			0						No-ASE
PDE1C	7	31793046													0									No-ASE
LSM5	7	32525631	0			0						0						0					0	No-ASE
AVL9	7	32582779								0		0						0						No-ASE

BBS9	7	33185898	0					0															0	No-ASE
NPSR1	7	34818113			0	0	0	0			0			1	0			0					1	No-ASE
DPY19L1	7	34979905		0	0						0											0	0	No-ASE
HERPUD2	7	35677978		0		0		1							0									No-ASE
EEPD1	7	36193992								0	0							0						No-ASE
ANLN	7	36435887	0		0			0		0	0	0	0		0							0	0	No-ASE
AOAH	7	36552772	0							0			0										0	No-ASE
SFRP4	7	37947103	0											0	0									No-ASE
EPDR1	7	37960206				1																		ASE
VPS41	7	38765883								0			0	0										No-ASE
YAE1D1	7	39606003	0					0		0		0												No-ASE
CDK13	7	39991072		0				0					0										0	No-ASE
INHBA	7	41729529		0																				No-ASE
GLI3	7	42003792				0																		No-ASE
HECW1	7	43351401	0																					No-ASE
STK17A	7	43635583								0	0		0										0	No-ASE
BLVRA	7	43810764								0	0		1										0	No-ASE
URGCP-MRPS24	7	43916483	0	0		0		0		0	0	0	0	0	0			0						No-ASE
UBE2D4	7	43978084		0	0			0			0	0		0	0			0				0		No-ASE
AEBP1	7	44146151				0				0	0		1		0			0					0	No-ASE
NUDCD3	7	44431912										0												No-ASE
NPC1L1	7	44553204	0		0	0					0	0						0					0	No-ASE
OGDH	7	44663949								0														No-ASE
ZMIZ2	7	44796067			0	0		0				0		0	0			0	0				0	No-ASE
H2AFV	7	44874113	1			1						1	1										1	ASE
MYO1G	7	45002388		0		0	0	0		0	0	0						0				0	1	No-ASE
CCM2	7	45067314		0		0		0			0	0	0		0			0		0		0		No-ASE
NACAD	7	45121241										0						0				1		Heterogeneous_ASE
TBRG4	7	45139949				0		0			0		0											No-ASE
IGFBP3	7	45954465								0														No-ASE
TNS3	7	47319774			0	0					0	0	0	0	0		0	0				0		No-ASE
PKD1L1	7	47832225	0	0	1	0		0		0	0	0	0	0	0		0	0	0			0	1	No-ASE
HUS1	7	48004991								0														No-ASE
C7orf57	7	48080972		1		0					0	1			0			0						Heterogeneous_ASE
UPP1	7	48128850	0	0		0		0			0		0	0				0					0	No-ASE
ABCA13	7	48259057	0	0				0			0	0		0	0			1	0	1		0	1	No-ASE
FIGNL1	7	50513062						0										0						No-ASE
DDC	7	50530946										0					0						0	No-ASE
COBL	7	51092832	0	0	0	0	0	0		0	0		0	0			0					0	0	No-ASE
EGFR	7	55211050				0						0	0		0		0							No-ASE
LANCL2	7	55433884				0					0	0	0	0	0		0						0	No-ASE
SUMF2	7	56136260			0																			No-ASE
ZNF680	7	63982238												1				0						Heterogeneous_ASE
ZNF107	7	64166802		0																			0	No-ASE
ZNF273	7	64363742			0						0	0		0								0	0	No-ASE
ERV3-1	7	64451646	0	0	0		0	0		0	0	0		0	1		0	0				0	0	No-ASE
ZNF92	7	64863392												0			0						0	No-ASE
GUSB	7	65425894	0					0		0				0	0	0	0	0	0	0		0	0	No-ASE
CRCP	7	65599361		0				0		0		0		0	0		0							No-ASE
AUTS2	7	69364311																0					0	No-ASE
TYW1B	7	72093938				0					0	0					0	0				1	0	No-ASE
TBL2	7	72985024			0			0														0		No-ASE
MLXIPL	7	73008233	0		0							0						0						No-ASE
DNAJC30	7	73097221	0		0	0		0				0		0	0			0					0	No-ASE
CLDN4	7	73245592				0																		No-ASE
WBSCR27	7	73249092	0		0	1		0							0	0	0	0		0		0		No-ASE
WBSCR28	7	73275565		0		0		0	0			0	0		0			0				0		No-ASE
ELN	7	73450896					0				0	0	1				0						1	Heterogeneous_ASE
CLIP2	7	73731968			0						0													No-ASE
GTF2IRD1	7	73929751								0														No-ASE
HIP1	7	75167524															0							No-ASE
CCL24	7	75441170						0			0						0							No-ASE
RHBDD2	7	75511203												0										No-ASE
POR	7	75583335	0		0				0	0					0		0					0	0	No-ASE
MDH2	7	75687382										0											0	No-ASE
SRCRB4D	7	76019476	0			0		0														0	0	No-ASE
ZP3	7	76054372	0			0						0		0										No-ASE
DTX2	7	76111835						0						0			0					0		No-ASE
CCDC146	7	76826258						0				0					0							No-ASE
PION	7	76941245			0	0		0		0	0	0		0			0					0	0	No-ASE
PTPN12	7	77166931			0	0			0		0	0	0	0	0		0	0				0		No-ASE
PHTF2	7	77522948	0			0			0			0										0		No-ASE
GNAT3	7	80088014															0							No-ASE
PCLO	7	82387905	0		0			0		0	0	0	0	0	0			0	0			0	1	No-ASE
SEMA3E	7	82997081															0							No-ASE
SEMA3D	7	84628989										1				1								ASE
CROT	7	86978418	0					1																Heterogeneous_ASE
ABCB4	7	87032531	0						1		0				0			0				0	0	No-ASE
ABCB1	7	87135347			0	0					0		0	0				0			0			No-ASE
STEAP4	7	87908744	0					1														0	0	No-ASE
ZNF804B	7	88423502																0						No-ASE
STEAP2	7	89854446		0		0		0	0	0			0	0								0	0	No-ASE
C7orf63	7	89874804	1	0		0		0	0	0			0									0	0	No-ASE
GTPBP10	7	89982260	0	0		0		0		0			0						0			0	0	No-ASE
MTERF	7	91503197			0	0								0	1			0						No-ASE
AKAP9	7	91570454		0		0		0			0	0			0		0	0				0		No-ASE
ANKIB1	7	91936684		0		0			0					0	0		0	0		0		0		No-ASE
RBM48	7	92158135						0																No-ASE
SAMD9	7	92730651	0			0				0		0					0					0		No-ASE
SAMD9L	7	92760738				0						0	0		0							0		No-ASE
CALCR	7	93055724											1		1		0							Heterogeneous_ASE
BET1	7	93623458	0	0					0	0		0		0			0						0	No-ASE
PEG10	7	94293129																0				1	1	Heterogeneous_ASE
PPP1R9A	7	94539540	0		0			0				0					0					1	0	No-ASE
PON1	7	94931560	0		1		0	0		0		0							1					Heterogeneous_ASE
PON2	7	95034775		0	0				0	0					0			0				0		No-ASE
ASNS	7	97482484		0	0			0									0							No-ASE
LMTK2	7	97770840		0	0	0		0	0	0		0	0		0			0		0		0	0	No-ASE
TECPR1	7	97846804		0	0				0	0		0												No-ASE
KPNA7	7	98775592			0			0					0									0		No-ASE
ATP5J2-PTCD1	7	99017607										0												No-ASE
ZNF789	7	99081730			0			0		0				0	0									No-ASE
ZKSCAN5	7	99103684												0										No-ASE
ZSCAN25	7	99217353																	0					No-ASE
OR2AE1	7	99473714	0		0	0	0			0	1		1	0	0							0	1	Heterogeneous_ASE
TRIM4	7	99489571		0	0					0		0												No-ASE
GJC3	7	99521208				0				0														No-ASE
ZSCAN21	7	99654642			0			0		0	0		0		0			0				0	0	No-ASE
ZNF3	7	99668803	0		0																			No-ASE
MCM7	7	99690722			0			0		0	0		0		0	0		0			0	0	0	No-ASE
AP4M1	7	99699581	0	0		0								0			0							No-ASE
GAL3ST4	7	99757553			0					0	0	0	0	0			0					0	0	No-ASE
GATS	7	99817603			0			0			0		0		0			0				0	0	No-ASE
SPDYE3	7	99905325		0										0									0	No-ASE
PILRA	7	99971770				0																		No-ASE
ZCWPW1	7	99998720											0		0			0						No-ASE
TSC22D4	7	100071904																				0		No-ASE
AGFG2	7	100146467				0		0																No-ASE

LRCH4	7	100172783																				0		No-ASE
FBXO24	7	100184265			0	0					0	0												No-ASE
GIGYF1	7	100279376							0		0											0		No-ASE
ZAN	7	100344205			0							0					0	0						No-ASE
TRIP6	7	100465747	1	0				1	0			0	0					0						Heterogeneous_ASE
ACHE	7	100488658				0		0	0								0							No-ASE
MUC12	7	100612961			0						0	0	0	0		0	0	0	0		0		0	No-ASE
MUC17	7	100663420						0	0			0		0				0				0	0	No-ASE
TRIM56	7	100731026									0													No-ASE
FIS1	7	100883020												0										No-ASE
CUX1	7	101713691	0	0				0			0													No-ASE
SH2B2	7	101928495	0	0		0		0			0			0				0				0		No-ASE
LOC100630923	7	102036866									0			0	0									No-ASE
ALKBH4	7	102097966			0												0						0	No-ASE
LRWD1	7	102106444									0			0										No-ASE
FBXL13	7	102453914	0	0											0				0			0		No-ASE
NAPEPLD	7	102743893	0	0											0							0		No-ASE
PMPCB	7	102937914												0								0		No-ASE
DNAJC2	7	102957374								0														No-ASE
PUS7	7	105098241						0			0													No-ASE
RINT1	7	105172803		0		0								0									0	No-ASE
ATXN7L1	7	105250980												0										No-ASE
CDHR3	7	105603809	0	0		0						0	0	0	0			0						No-ASE
PIK3CG	7	106508268											1				0							Heterogeneous_ASE
PRKAR2B	7	106781370		0						1				0				0						No-ASE
COG5	7	106844050		0	0		0			0	0		0	0			0	0		0			0	No-ASE
DUS4L	7	107211657									0				0									No-ASE
SLC26A3	7	107408037							0															No-ASE
LAMB1	7	107564485	0			0																0		No-ASE
LAMB4	7	107671300				0		1	1	1					1		0							Heterogeneous_ASE
NRCAM	7	107790417	0																				0	No-ASE
DOCK4	7	111368454			1																			ASE
TES	7	115874657										0	0									0	0	No-ASE
CAV2	7	116140419	0	0		0		1		0	0			0										No-ASE
CFTR	7	117120179	0	0						0		1	0	0	0			0					1	No-ASE
CTTNBP2	7	117351696													0									No-ASE
CPED1	7	120629916		1	0				0				1					0				0		Heterogeneous_ASE
WNT16	7	120969638		0	0		0			0	0	0			1		0	1				0	0	No-ASE
PTPRZ1	7	121513561	0	1								0												Heterogeneous_ASE
CADPS2	7	121965550			0																			No-ASE
SLC13A1	7	122755609	0																					No-ASE
ASB15	7	123254647										0		0				0						No-ASE
ZNF800	7	127013672							0		0													No-ASE
GCC1	7	127222318											0											No-ASE
SND1	7	127326687									0													No-ASE
RBM28	7	127950857	0							0		0												No-ASE
PRRT4	7	127991133				0		0										0						No-ASE
FAM71F2	7	128312463				0	0		0	0		0					0						1	No-ASE
FAM71F1	7	128355676			0	1		0					1	0	0		1					0	1	Heterogeneous_ASE
CALU	7	128388648						0										0				0		No-ASE
OPN1SW	7	128413726												0										No-ASE
CCDC136	7	128432446																				0		No-ASE
LOC100130705	7	128506470							0													0	0	No-ASE
KCP	7	128517183				0																0		No-ASE
STRIP2	7	129079918						0																No-ASE
ZC3HC1	7	129662352	0		0	0	0	0	0	0	0	0	0				0	0				0		No-ASE
C7orf45	7	129855818					0																	No-ASE
CPA4	7	129938598			0									0										No-ASE
CPA5	7	129984630				1	0							1	0		0			1				Heterogeneous_ASE
COPG2	7	130146607							0		0													No-ASE
FLJ43663	7	130629901		0	0			0	0		0			0	0		0		0				0	No-ASE
PODXL	7	131189107		0		0	0	0	0	0	0		0		0		0					0	0	No-ASE
PLXNA4	7	131815312					0											0						No-ASE
CHCHD3	7	132481247						0																No-ASE
LRGUK	7	133812220									0	0												No-ASE
AKR1B10	7	134215396								0							0							No-ASE
CALD1	7	134617749										0					0	0				1		No-ASE
TMEM140	7	134849213		0						0			0	0	0									No-ASE
WDR91	7	134870998			0				0					0	0		0			0		0	0	No-ASE
CNOT4	7	135047811			0	0					0	0		0	0								0	No-ASE
NUP205	7	135242703																				0		No-ASE
SLC13A4	7	135366424			1					0													0	Heterogeneous_ASE
CREB3L2	7	137567348			0							0		0	0									No-ASE
TRIM24	7	138200030	0																					No-ASE
SVOPL	7	138281268				1						1			1			0					1	ASE
KIAA1549	7	138522671		0				0			0		0		0				0			0		No-ASE
ZC3HAV1L	7	138711291			0																			No-ASE
ZC3HAV1	7	138732434	0		0		0	0	0		0	0	0	0			0			0		0	0	No-ASE
TTC26	7	138822599		0							0		0											No-ASE
UBN2	7	138916654		0																				No-ASE
LUC7L2	7	139026152									0				0							0	0	No-ASE
KLRG2	7	139138437										0												No-ASE
HIPK2	7	139259911										0	0			0	0	0	0				0	No-ASE
TBXAS1	7	139529239										0												No-ASE
PARP12	7	139724496												0								0		No-ASE
JHDM1D	7	139791645	0					0	0	0			0	0				0				0		No-ASE
SLC37A3	7	140035268	0		0	0		0					0	0				0					0	No-ASE
RAB19	7	140111711	0		0							0										0		No-ASE
DENND2A	7	140221699																0						No-ASE
ADCK2	7	140373254		0					0			0	0				0	0						No-ASE
AGK	7	141255283																				0		No-ASE
TAS2R4	7	141478296		0	0			0	0	0				0	0		0	0				0	0	No-ASE
TAS2R5	7	141490219		0	0			0		0				0	0		0	0		0		0	0	No-ASE
PRSS37	7	141536258		0	0			0		0				0	0		0	0				0	0	No-ASE
TAS2R38	7	141672604		0	0			0	0	0		0	0		0			0	0			0	0	No-ASE
TRPV6	7	142569596		0																		0		No-ASE
KEL	7	142638355																0					0	No-ASE
OR6V1	7	142749439																0						No-ASE
PIP	7	142829211	0			0																	0	No-ASE
TAS2R40	7	142919238		0																				No-ASE
CASP2	7	142985603							0															No-ASE
CLCN1	7	143013391	0	1		1							0		1			0						Heterogeneous_ASE
FAM131B	7	143053713				0						0	0		0		0							No-ASE
ZYX	7	143079922																0						No-ASE
EPHA1	7	143088576	0			0		0	0								0	0					0	No-ASE
EPHA1-AS1	7	143140577					0											0						No-ASE
OR2F2	7	143632833		1														0						Heterogeneous_ASE
OR2F1	7	143657104		0		0																		No-ASE
OR2A5	7	143747497		0														0	0					No-ASE
OR2A12	7	143792472	1						1					1									1	ASE
OR2A2	7	143806688										1		1	0			0						Heterogeneous_ASE
OR2A14	7	143826248																1				0		Heterogeneous_ASE
NOBOX	7	144094418	0			1			1	1		1			1		1					1		ASE
C7orf33	7	148288020																0						No-ASE
EZH2	7	148507426																					0	No-ASE
PDIA4	7	148701005				0			0			0					0							No-ASE
ZNF786	7	148767538											0											No-ASE
ZNF425	7	148800715		0		0				0		0			0		0					0		No-ASE

ZNF398	7	148851037							0				0		0									No-ASE
ZNF282	7	148895510												0										No-ASE
ZNF212	7	148947862												0	0									No-ASE
ZNF777	7	149129475	0	0	0	0			0	0		0	0					0				0	0	No-ASE
KRBA1	7	149417162												1				0					0	Heterogeneous_ASE
ZNF467	7	149461895				0						0												No-ASE
SSPO	7	149473146				0	0	1				0					0	0				0	1	No-ASE
ZNF862	7	149541777		0					0			0						0						No-ASE
ACTR3C	7	149981855												0										No-ASE
RARRES2	7	150035721												0										No-ASE
REPIN1	7	150066886	0		0													0						No-ASE
GIMAP2	7	150384150								1			1	0										Heterogeneous_ASE
TMEM176B	7	150489176	0	0	0	0	0	1		0					0			0					0	No-ASE
TMEM176A	7	150499378			0								0		0			0				1		No-ASE
ABP1	7	150553605	0	0		0		0	0	0		0	0	0			0	0						No-ASE
KCNH2	7	150645534	0			0						0			0		0					0		No-ASE
ABCB8	7	150725631	0						0															No-ASE
ASIC3	7	150745974																				0		No-ASE
SLC4A2	7	150761314	0					0				0		0	0			0					0	No-ASE
CHPF2	7	150931237						0				0										0		No-ASE
NUB1	7	151042447										0												No-ASE
GALNTL5	7	151668063		1											1							1		ASE
GALNT11	7	151791436		0																				No-ASE
MLL3	7	151842287						0		0		0					0						0	No-ASE
XRCC2	7	152345762															0							No-ASE
DPP6	7	154561258																			1			ASE
LOC100132707	7	154738409										0					0					0	0	No-ASE
RBM33	7	155531080		0		0					0			0			0					0		No-ASE
RNF32	7	156437221	0	0		0		0	0					0	0		0	0			0			No-ASE
NOM1	7	156742562		0	0			0			0						0				0		0	No-ASE
PTPRN2	7	157333411	0	0		1		0	0					0	0		0		0	0		0	0	No-ASE
ESYT2	7	158528229	0		0						0	0			0	0		0	0					No-ASE
WDR60	7	158663897			0		0			0	0			0	0			0	1					No-ASE
ZNF596	8	190907										0		0										No-ASE
DLGAP2	8	1496924	1																					ASE
ARHGEF10	8	1806227						0							0									No-ASE
MYOM2	8	1993158	0			0								0						0			1	No-ASE
CSMD1	8	2795835												1										ASE
MCPH1	8	6272353	0			0						0		0	0								0	No-ASE
XKR5	8	6668746				0						0			0									No-ASE
SGK223	8	8175717	0			0						0		0	0					0				No-ASE
CLDN23	8	8560021	0											0										No-ASE
ERI1	8	8860620	0			0								0										No-ASE
PRSS55	8	10383105	0												0									No-ASE
RP1L1	8	10464405	0			0	0							0						1			0	No-ASE
PINX1	8	10622930	0									0			0					0				No-ASE
FAM167A	8	11301550					1	0																Heterogeneous_ASE
GATA4	8	11614483						0																No-ASE
FDFT1	8	11666337										0											0	No-ASE
CTSB	8	11702375	0			0		0				0		0						0			0	No-ASE
LONRF1	8	12594292												0										No-ASE
KIAA1456	8	12870279													0								0	No-ASE
DLC1	8	12943382				0		0						0						1				No-ASE
C8orf48	8	13424565													0									No-ASE
VPS37A	8	17125768													0									No-ASE
SLC7A2	8	17396379										0												No-ASE
MTUS1	8	17503316	0			0						0			0									No-ASE
PCM1	8	17793188	0												0					0			0	No-ASE
ASAH1	8	17915087	0	1		0						0			0		1			0			0	No-ASE
NAT1	8	18067463				0		0																No-ASE
NAT2	8	18257583						0						0									0	No-ASE
PSD3	8	18393371	0			0		0							0					0			0	No-ASE
SH2D4A	8	19171316										0											0	No-ASE
CSGALNACT1	8	19263328						1							0									Heterogeneous_ASE
LPL	8	19805708				1									0									Heterogeneous_ASE
SLC18A1	8	20003282										0												No-ASE
XPO7	8	21847855						0																No-ASE
FAM160B2	8	21955034	0	0		0								0	0								0	No-ASE
HR	8	21974516	0					0						0										No-ASE
SFTPC	8	22019351										0												No-ASE
SLC39A14	8	22262246				0		0						0	0					0			0	No-ASE
SORBS3	8	22412114						0						0										No-ASE
C8orf58	8	22458406																					0	No-ASE
KIAA1967	8	22463319												0										No-ASE
BIN3	8	22481483	0																					No-ASE
TNFRSF10B	8	22880284				0		0				0		0	0					0				No-ASE
TNFRSF10C	8	22972220												0										No-ASE
TNFRSF10D	8	22995385	0																				0	No-ASE
TNFRSF10A	8	23049208	0			0						0		0	0		0			0			0	No-ASE
R3HCC1	8	23148940	0			0		0				0			0					0				No-ASE
LOXL2	8	23156435				0		0																No-ASE
SLC25A37	8	23423697													0									No-ASE
DOCK5	8	25101205	0			0																		No-ASE
GNRH1	8	25276976	0			0																		No-ASE
CDCA2	8	25317913						0															0	No-ASE
PNMA2	8	26365201										1												ASE
DPYSL2	8	26481697	0			0						0											0	No-ASE
ADRA1A	8	26623647	0																					No-ASE
PTK2B	8	27255130	0					0				0			0					0			0	No-ASE
EPHX2	8	27358495												0	0									No-ASE
SCARA3	8	27507233				0																		No-ASE
ESCO2	8	27633972	0																					No-ASE
SCARA5	8	27729527										0												No-ASE
NUGGC	8	27880851				1									0					0			1	Heterogeneous_ASE
ELP3	8	27950736				0																		No-ASE
FBXO16	8	28304769										0												No-ASE
KIF13B	8	28928041						0																No-ASE
TEX15	8	30694321										1											1	ASE
WRN	8	30916058	0			0						0		0	0								0	No-ASE
FUT10	8	33230142						0						0									0	No-ASE
PROSC	8	37620221	0			0																		No-ASE
GPR124	8	37688966						0																No-ASE
RAB11FIP1	8	37720462			0			0						0			0			0	0		0	No-ASE
DDHD2	8	38091917	0		0	0									0	0								No-ASE
WHSC1L1	8	38139034											0											No-ASE
FGFR1	8	38271301												0										No-ASE
HTRA4	8	38832564	0																					No-ASE
TM2D2	8	38851112											0	1	0						1			Heterogeneous_ASE
ZMAT4	8	40438757					0																	No-ASE
ANK1	8	41519012					0																1	Heterogeneous_ASE
KAT6A	8	41790141												0										No-ASE
PLAT	8	42033519	0		0									0							0			No-ASE
HGSNAT	8	43002177			0																			No-ASE
PRKDC	8	48690299				0							0						0				0	No-ASE
MCM4	8	48874952		0	0		0		0							0				0				No-ASE
PXDNL	8	52232487					0							0	0	0				1		0		No-ASE
RB1CC1	8	53536363	0	0		0	0						0		0	0			0	0		0		No-ASE
NPBWR1	8	53852583					0					0												No-ASE

RP1	8	55533591	0				0													0				No-ASE
TGS1	8	56686224	0	0									0	0					0					No-ASE
MOS	8	57025644					0																	No-ASE
PLAG1	8	57078933	0			0	0	0							0									No-ASE
NSMAF	8	59496687																		0				No-ASE
TOX	8	59728090																					0	No-ASE
CA8	8	61178512			0								0			0							0	No-ASE
CHD7	8	61654118							0			0				0					0			No-ASE
ASPH	8	62415937				0																		No-ASE
NKAIN3	8	63492175					0		0															No-ASE
GGH	8	63938764				0			0						0									No-ASE
YTHDF3	8	64099225																				0		No-ASE
PDE7A	8	66631598					0																	No-ASE
RRS1	8	67341394						0							0				0					No-ASE
ADHFE1	8	67344767		0			0					0					0							No-ASE
C8orf44-SGK3	8	67590022						0						0		0			0					No-ASE
MCMDC2	8	67786401												0										No-ASE
CPA6	8	68334782				0													0					No-ASE
PREX2	8	68930106					0																	No-ASE
SLCO5A1	8	70585153				0	0		0			1			0	1	0		0	0	0	0		No-ASE
PRDM14	8	70980738					0																	No-ASE
NCOA2	8	71033556																				0		No-ASE
XKR9	8	71593497												1										ASE
LOC100132891	8	72935249																					1	ASE
TRPA1	8	72969228				0		0				0		0									1	No-ASE
SBSPON	8	73979655		0	0			0	1				0			0	0			0		0		No-ASE
STAU2-AS1	8	74333676						0								0								No-ASE
STAU2	8	74507522			0				0															No-ASE
TMEM70	8	74888585	0	0								0					0		0				0	No-ASE
HNF4G	8	76452313		0		0	0	0	0			0	0	0		0								No-ASE
ZFHX4	8	77616519		0										1								0	0	No-ASE
ZNF704	8	81577057																	0				0	No-ASE
SLC10A5	8	82606244		0					0										0					No-ASE
CHMP4C	8	82644869		0		0										0							0	No-ASE
SNX16	8	82713734																					0	No-ASE
LRRCC1	8	86019544						0											0					No-ASE
CA13	8	86171659															0							No-ASE
CA1	8	86240851																	0					No-ASE
CA3	8	86351961			0		0									0						0		No-ASE
SLC7A13	8	87226647		1								1			1									ASE
RMDN1	8	87497181	0	0			0									0			0	0				No-ASE
CPNE3	8	87540832			0		0															0		No-ASE
CNGB3	8	87588042					0												1					Heterogeneous_ASE
RIPK2	8	90775193				0								0										No-ASE
NBN	8	90949286					0		0				0		0	0						0	0	No-ASE
OTUD6B	8	92082574																	0					No-ASE
FAM92A1	8	94717177					0									0				0				No-ASE
RBM12B	8	94745597													0									No-ASE
TMEM67	8	94767139		0	0		0	0						0		0								No-ASE
CDH17	8	95140468	0	0	0	0		0				0	0	0	0		0		0	0	0	0	0	No-ASE
RAD54B	8	95384436			0	0								0	0	0			0	0	0	0		No-ASE
ESRP1	8	95654206						0	0				0			0					0	0		No-ASE
DPY19L4	8	95738586										1												ASE
SDC2	8	97506503	1																				0	Heterogeneous_ASE
MATN2	8	98900369	0			0	0		1			0	0	0		0	0		0	0		0	0	No-ASE
NIPAL2	8	99205289				0	0	0					0	0	0		0					0		No-ASE
VPS13B	8	100108606		0				0	0			0		0	0		0		0					No-ASE
SPAG1	8	101178106		0		0		0					0	0	0				0	0		0	0	No-ASE
SNX31	8	101586125	0		0			0				0					0					0		No-ASE
RRM2B	8	103225061		0																				No-ASE
ODF1	8	103564006															1							ASE
FZD6	8	104312381	0			0	0	0	0			0	0			0	0		0	0		0	0	No-ASE
SLC25A32	8	104412688	0		0		0	0				0	0		0	0						0	0	No-ASE
DCAF13	8	104427541					0					0				0	0			0			0	No-ASE
ZFPM2	8	106431375		0			0									0						1		No-ASE
NUDCD1	8	110255347	0	0			0	0	0			0		0		0	0				0	0	0	No-ASE
PKHD1L1	8	110393640					0					0					0							No-ASE
CSMD3	8	113241021			0									0						0				No-ASE
UTP23	8	117778979		0					0			0							0					No-ASE
RAD21	8	117859924				0			0								0							No-ASE
SLC30A8	8	118159193					0																	No-ASE
TNFRSF11B	8	119936717	0	0	0				0					0			0					0	0	No-ASE
MAL2	8	120233910			0		0		0								0							No-ASE
NOV	8	120428792				0	0	1																Heterogeneous_ASE
TAF2	8	120744399	0		0	0		0	0			0	0	0			0				0			No-ASE
DSCC1	8	120847188							0			0					0							No-ASE
DEPTOR	8	120886100		0	0		0		0			0	0	0	0		0		0		0	0		No-ASE
COL14A1	8	121160097														0								No-ASE
MTBP	8	121457827	0																					No-ASE
SNTB1	8	121551200				0																		No-ASE
ZHX2	8	123963776		0																				No-ASE
DERL1	8	124031444				0								0								0		No-ASE
WDR67	8	124085043				0																		No-ASE
FAM83A	8	124195140	0																					No-ASE
WDYHV1	8	124440174		0	0	0			0			0	0		0						0			No-ASE
FBXO32	8	124516917	0			0		0					0		0		0						0	No-ASE
KLHL38	8	124658193															0					0		No-ASE
ANXA13	8	124693509	0	0	0	0	0		0			0	0	0	0					0	0	0		No-ASE
FER1L6	8	124968243	0	0	0	0		0	0			0	0		0		0		0			0		No-ASE
TRMT12	8	125463211	0	0		0		1				0	0	0		0	0							No-ASE
NDUFB9	8	125555335			0	0						0										0		No-ASE
MTSS1	8	125565328	0	0	0	0			0			0		0		0			0		0	0		No-ASE
ZNF572	8	125987907													0							0		No-ASE
GSDMC	8	130760802		0	0																			No-ASE
ASAP1	8	131072829		0					0			0		0		0					0		1	No-ASE
LRRC6	8	133584558		0				0							0	0							0	No-ASE
TG	8	133880375	0	0	0	0	0	0	0			0	0	0	0	0	0		0	0	0	0	0	No-ASE
WISP1	8	134203425		0																				No-ASE
ZFAT	8	135490774		0		0		0	0			0			0	0	0		0			0	0	No-ASE
TRAPPC9	8	140743304		0	0	0		0	0			0	0	0	0		0		0		0		0	No-ASE
PTK2	8	141678415												0										No-ASE
DENND3	8	142146794		0		0	0	0	0			0		0	0				0					No-ASE
SLC45A4	8	142221626	0	0		0		0	0			0	0	0	0		0				0			No-ASE
GPR20	8	142367014																				0		No-ASE
MROH5	8	142444653	0	0			0	1						1	1	1				0	1			Heterogeneous_ASE
TSNARE1	8	143310853		0	0	0	0		0				0		0	0							0	No-ASE
JRK	8	143740311	0	0	0	0	0	0				0	0		0		0		0		0		0	No-ASE
PSCA	8	143761931	0														1							Heterogeneous_ASE
LY6D	8	143866711														0								No-ASE
C8orf31	8	144124609		0	0	1										0	0					1		Heterogeneous_ASE
GPIHBP1	8	144295183																					1	ASE
GLI4	8	144351610			0			0						0	0		0			0		0		No-ASE
ZNF696	8	144377951							0															No-ASE
TOP1MT	8	144391640	0		0									0			0			0		0		No-ASE
ZC3H3	8	144522247		0	0	0		0	0			0		0	0	0	0		0			0	0	No-ASE
MROH6	8	144650013	0				0	0	0			0		0									0	No-ASE
NAPRT1	8	144657182		0								0							0					No-ASE
EEF1D	8	144661982		0								0					0							No-ASE

TIGD5	8	144681397						0				0					0							No-ASE
PYCRL	8	144687896		0				0	0										0				0	No-ASE
ZNF707	8	144772282		0				0						0										No-ASE
MAPK15	8	144799903												0										No-ASE
FAM83H	8	144808293		0	0							0			0							0		No-ASE
SCRIB	8	144873405	0			0		0				0		0	0					0				No-ASE
EPPK1	8	144940217	0	0	0	0		0				0	0	0	0	0	0		0	0		0	0	No-ASE
PLEC	8	144990587	0	0			0	0	0			0	0	0			0			0	0	0		No-ASE
PARP10	8	145051711	0			0		0				0	0	0						0	0	0		No-ASE
SPATC1	8	145086736	0		0	0														0				No-ASE
OPLAH	8	145106251			0	0		0					0	0			0		0					No-ASE
SHARPIN	8	145154091		0				0						0										No-ASE
MAF1	8	145160983				0		0											0					No-ASE
MROH1	8	145223240				0													0					No-ASE
FBXL6	8	145579649	0									0				0	0			0				No-ASE
ADCK5	8	145603114	0		0			0				0	0			0			0	0	0	0		No-ASE
SLC39A4	8	145638286		0	0	0		0	0						0		0		0		0	0	1	No-ASE
TONSL	8	145655903		0	0	0			0				0		0		0		0		0	0	0	No-ASE
GPT	8	145729710	0			0						0	0	0							0		0	No-ASE
MFSD3	8	145735793																	0					No-ASE
RECQL4	8	145736819	0			0			0			0				0	0				0		0	No-ASE
LRRC14	8	145745251												0					0					No-ASE
ZNF251	8	145947075															0							No-ASE
RPL8	8	146015154													0		0							No-ASE
ZNF517	8	146028316	0		0							0												No-ASE
ZNF7	8	146062841		0		0		0	0					0	0	0	0			0		0		No-ASE
COMMD5	8	146076066		0		0		0							0		0			0		0		No-ASE
ZNF252P	8	146225112			0				0											0	0			No-ASE
C9orf66	9	214517				0	0	1		1	0													Heterogeneous_ASE
DOCK8	9	271729				0		1		1		0	1	0		0							0	Heterogeneous_ASE
KANK1	9	676988				0	0					0	0			0				0	0		0	No-ASE
DMRT3	9	977054																					1	ASE
DMRT2	9	1051758						0		1				0							0		0	No-ASE
KCNV2	9	2717755									0													No-ASE
KIAA0020	9	2804404		1			0			0			0	0		0				0			0	No-ASE
SLC1A1	9	4490715				0		0	0		0	0				0				1				No-ASE
SPATA6L	9	4605429												0										No-ASE
ERMP1	9	5787242												0										No-ASE
KIAA2026	9	5919734				0		0			0									0			0	No-ASE
KDM4C	9	6849628				0	0	0		0	0		0	0							0			No-ASE
PTPRD	9	8319965				0																	1	Heterogeneous_ASE
CER1	9	14720158												1										ASE
FREM1	9	14737506				0	0		0	0	0		0	1		1				0	0		0	No-ASE
TTC39B	9	15175039					0	0		0			0			0				0				No-ASE
SNAPC3	9	15422957											0											No-ASE
CCDC171	9	15564100								0	0	0	0										0	No-ASE
CNTLN	9	17135286				0					0			0										No-ASE
ADAMTSL1	9	18504954																					0	No-ASE
FAM154A	9	18928083					0																	No-ASE
HAUS6	9	19056385									0		0										0	No-ASE
DENND4C	9	19290794											0										0	No-ASE
RPS6	9	19376255								0														No-ASE
FOCAD	9	20715401				0								0							0		0	No-ASE
PTPLAD2	9	21008127												0										No-ASE
MTAP	9	21816758									0	0				0				0				No-ASE
CDKN2B-AS1	9	22003223						0				0								0			1	No-ASE
TUSC1	9	25677912																		0				No-ASE
IFT74	9	26956371		0						0		0	0							0				No-ASE
TEK	9	27109600											0											No-ASE
C9orf72	9	27556664		0		0						0	0	0		0				0				No-ASE
ACO1	9	32405589															0							No-ASE
DDX58	9	32457151										0				0							0	No-ASE
TOPORS	9	32541468											0											No-ASE
SPINK4	9	33240216				0		0		0		0	1										0	No-ASE
NFX1	9	33294549												0									0	No-ASE
UBAP2	9	33922582					0	0		0		0		0		0	0			0			0	No-ASE
DCAF12	9	34093400								0		0												No-ASE
KIF24	9	34254425				0				0			0	0		0				0			0	No-ASE
KIAA1161	9	34371171			0								0			0				0				No-ASE
C9orf24	9	34379084						0			0												1	Heterogeneous_ASE
DNAI1	9	34459043						0																No-ASE
ARID3C	9	34622389				0		0		0	0	0		0						0			0	No-ASE
IL11RA	9	34655629										0												No-ASE
FAM205A	9	34723278		1	0							1		0						0				Heterogeneous_ASE
DNAJB5	9	34996397									0													No-ASE
C9orf131	9	35042268				0		0			0					1					0		1	Heterogeneous_ASE
VCP	9	35056961			0		0			0	0													No-ASE
FANCG	9	35074490																		0				No-ASE
UNC13B	9	35231123										0												No-ASE
RUSC2	9	35546735												0										No-ASE
FAM166B	9	35563062								0														No-ASE
CD72	9	35610691					0				0													No-ASE
CCDC107	9	35660414		0			0			0	0			0		0	0				0			No-ASE
ARHGEF39	9	35661943				0				0	0		0	0		0					0			No-ASE
CA9	9	35674185								0	0		0	0		0								No-ASE
FAM221B	9	35819211			0	0	0					0	0											No-ASE
OR13J1	9	35869555									0			0										No-ASE
HRCT1	9	35906390								1	0	1	1	1		1				0			1	ASE
OR2S2	9	35957288				0										1				0			0	No-ASE
RECK	9	36037065														0								No-ASE
CCIN	9	36169539					0										1			1				Heterogeneous_ASE
PAX5	9	36840623			0		0																	No-ASE
ZBTB5	9	37440620			0		0					0		0						0			0	No-ASE
POLR1E	9	37486109			0			0		0				0		0							0	No-ASE
FRMPD1	9	37692654			0																			No-ASE
EXOSC3	9	37780747								0	0													No-ASE
ALDH1B1	9	38395909								0			0	0		0				0				No-ASE
PIP5K1B	9	71395138						0								0				0			0	No-ASE
TJP2	9	71820148								0		0		0		0				0	0		0	No-ASE
APBA1	9	72071237										0												No-ASE
SMC5	9	72879264			0			0			0			0	0						0			No-ASE
KLF9	9	73028001														0								No-ASE
TRPM3	9	73150873															0							No-ASE
TMEM2	9	74300174				0		0		0	0	0	0	0	0	0				0	0		0	No-ASE
GDA	9	74764545																			0			No-ASE
TRPM6	9	77354770				0	0			0		0		1										No-ASE
PCSK5	9	78547338			0	0	0	1		0	0			0	0		0				1		0	No-ASE
GCNT1	9	79117312			0			0					0			0					0			No-ASE
PRUNE2	9	79244174			0					0	0		0		0	0	0				0		0	No-ASE
VPS13A	9	79824351				0																	0	No-ASE
TLE4	9	82242309															0							No-ASE
RASEF	9	85597642		1	0	0					0	0		0		0	0				0			No-ASE
FRMD3	9	85863069													1	0								Heterogeneous_ASE
IDNK	9	86258357					0				0	0									0		0	No-ASE
UBQLN1	9	86278899									0													No-ASE
KIF27	9	86465079						0				0	0								0			No-ASE
RMI1	9	86615956						0		0	0					0					0			No-ASE
SLC28A3	9	86893201			0					0			0											No-ASE

AGTPBP1	9	88162066														0								No-ASE
ZCCHC6	9	88916274			0	0					0		0	0			0				1		0	No-ASE
DAPK1	9	90252858			0			1				0	0	0							1		0	Heterogeneous_ASE
SPATA31E1	9	90497810				0	0			1	0	1	1										1	Heterogeneous_ASE
LOC286238	9	91262347		1			0																	Heterogeneous_ASE
C9orf47	9	91605923					0														0			No-ASE
S1PR3	9	91616138																			0			No-ASE
SHC3	9	91653075														1					0			Heterogeneous_ASE
SEMA4D	9	91977321			0		0			0	0	0	0		0	0	0				0		0	No-ASE
IARS	9	94973151														0								No-ASE
NOL8	9	95060161				0										0							0	No-ASE
CENPP	9	95094474															0							No-ASE
IPPK	9	95378124														0								No-ASE
FGD3	9	95738810											0											No-ASE
SUSD3	9	95838156								0														No-ASE
WNK2	9	95992044			0	0	0	0		0		0	0	0		0	0				0		0	No-ASE
FAM120AOS	9	96209933			0			0		0	0			0		0								No-ASE
PHF2	9	96398726									0			0			0							No-ASE
ZNF169	9	97055310			0	0		0		0	0	0	0			0	0						0	No-ASE
FBP2	9	97321221					1																	ASE
FBP1	9	97365742										0					0				0			No-ASE
PTCH1	9	98209213									0	0		0		0	0				0		0	No-ASE
ERCC6L2	9	98638306				0							0										0	No-ASE
ZNF510	9	99521119											0											No-ASE
ZNF782	9	99580257						0			0	0	0											No-ASE
LOC100499484-C9ORF174	9	100070367									1	0	1										0	Heterogeneous_ASE
TDRD7	9	100190936									0	0		0			0				0			No-ASE
TMOD1	9	100286484									0													No-ASE
TSTD2	9	100364783			0	0		0				0												No-ASE
NCBP1	9	100405634						0		0														No-ASE
C9orf156	9	100667157					0	0		0	0		0	0	0	0	0				0			No-ASE
NANS	9	100819143								0				0			0				0			No-ASE
TBC1D2	9	100961704									0	0		0	0	0	0							No-ASE
GALNT12	9	101570283			0			0			0										0		0	No-ASE
COL15A1	9	101747885					0					1												Heterogeneous_ASE
ALG2	9	101980335			0	0																		No-ASE
STX17	9	102677552											0			0	0						0	No-ASE
INVS	9	102866870									0		0											No-ASE
LPPR1	9	103947810			0																			No-ASE
BAAT	9	104124952			0			0						0	0		0						0	No-ASE
ZNF189	9	104170241				0						0		0			0				0			No-ASE
ALDOB	9	104187133											1											ASE
GRIN3A	9	104335472				1	0	0																Heterogeneous_ASE
OR13D1	9	107456763			1	1						0	1											ASE
NIPSNAP3A	9	107513256			0	0		0				0				0	0				0		0	No-ASE
NIPSNAP3B	9	107526539			1	0				1						1								ASE
ABCA1	9	107546652			0		0			0	0	0		0										No-ASE
SLC44A1	9	108097888			0			0							0	0					0			No-ASE
FSD1L	9	108230553																					1	ASE
FKTN	9	108337338				0		0			0	0			0	0	0				0			No-ASE
ZNF462	9	109686435				0	0	0		0	0			0	0	0	0						0	No-ASE
ACTL7A	9	111624712																					0	No-ASE
IKBKAP	9	111637237			0	0	0	0	0	0	0		0											No-ASE
FAM206A	9	111696795			0																			No-ASE
CTNNAL1	9	111705049			0	0		0			0		0				0							No-ASE
EPB41L4B	9	111938834												0										No-ASE
PTPN3	9	112141832			0			0			0	0		0	0									No-ASE
PALM2-AKAP2	9	112898528								0	0	0		0	0						0		1	No-ASE
SVEP1	9	113132221			1	0					1	1			1	0								Heterogeneous_ASE
MUSK	9	113449489				0																		No-ASE
KIAA0368	9	114131383												0		0	0							No-ASE
PTGR1	9	114341111		0		0		0				0												No-ASE
C9orf84	9	114449112		0		0						0			0						0			No-ASE
MIR3134	9	114820731		0																				No-ASE
SNX30	9	115598610			0						0			0		0							0	No-ASE
ZFP37	9	115805036		1						0														Heterogeneous_ASE
FKBP15	9	115928396		0	0	0		0			0					0	0				0		0	No-ASE
SLC31A1	9	116025024		0		0		0		0		0	0	0	0		0				0		0	No-ASE
RNF183	9	116059983		0		0				0	1		0								0			No-ASE
WDR31	9	116080766								0														No-ASE
BSPRY	9	116116523								0			0								0		0	No-ASE
HDHD3	9	116135892			0	0				0	0		0	0	0		0						0	No-ASE
ALAD	9	116151277					0	0																No-ASE
C9orf43	9	116175858		0		0															0		0	No-ASE
RGS3	9	116224308		0							0					0							0	No-ASE
ZNF618	9	116750780						0					0				0							No-ASE
KIF12	9	116854714								0				0	0						0		1	No-ASE
COL27A1	9	116929987		0		0	0	1		0	0	0		1			0							No-ASE
AKNA	9	117099420		0		0	0	0			0	0		0	0						0			No-ASE
DFNB31	9	117165093				0	0	0			0	0		0	0		0				0		0	No-ASE
LOC100505478	9	117428960		0	0	0	0				0	0		0		0								No-ASE
TNFSF15	9	117552809		0				0		0	0	0	0								0			No-ASE
TNC	9	117786253		0	0	0	0	0		0	1	0		0	0	0					0		1	No-ASE
37226	9	118163563												1										ASE
PAPPA	9	118949459		1		1					0	1												ASE
ASTN2	9	119188134									0						0							No-ASE
DBC1	9	121929412																					0	No-ASE
CDK5RAP2	9	123152042		0	0			0																No-ASE
TRAF1	9	123673673						0				0												No-ASE
C5	9	123722537		0	0	0	0	0		0	0	0		0		0	0				0		0	No-ASE
CNTRL	9	123850642		0	0	0	0			0	0	0	0	0		0	0				0		0	No-ASE
GSN	9	124064268		0																				No-ASE
STOM	9	124103523											0											No-ASE
DAB2IP	9	124441020																			0		0	No-ASE
TTLL11	9	124584915												0										No-ASE
PTGS1	9	125133479					0			0				0										No-ASE
OR1N1	9	125288720		1													1							ASE
OR1N2	9	125315478		1												1								ASE
OR1Q1	9	125377087		1																				ASE
OR1B1	9	125390874			0		0					0		0							0			No-ASE
OR1L4	9	125486270												0										No-ASE
PDCL	9	125582173						0				0	0		0						0			No-ASE
CRB2	9	126118577		0	0	0						0		0		0	0						0	No-ASE
PSMB7	9	127115893			0			0		0	0	0		0			0							No-ASE
GPR144	9	127213445					0																	No-ASE
OLFML2A	9	127549437			0																			No-ASE
WDR38	9	127615858		0			0	0			0					0								No-ASE
ARPC5L	9	127635985		0	0	0								0	0		0				0			No-ASE
GOLGA1	9	127642825		0				0		0	0					0								No-ASE
SCAI	9	127715169		0						0						0								No-ASE
PPP6C	9	127910307		0		0				0	0			0		0								No-ASE
RABEPK	9	127975654				0					0			0										No-ASE
ZBTB34	9	129642645										0												No-ASE
RALGPS1	9	129728123																			0			No-ASE
SLC2A8	9	130162220			0																			No-ASE
ZNF79	9	130186700		0	0	0	0			0		0	0	0							0		0	No-ASE
LRSAM1	9	130217298		0		0						0				0					0		0	No-ASE
C9orf117	9	130469384			0	0						0	0		0									No-ASE

TTC16	9	130478427			1	0																		Heterogeneous_ASE
TOR2A	9	130494335		0	0	0				0		0	0		0									No-ASE
FPGS	9	130566628				0					0		0	0										No-ASE
AK1	9	130630342			0																			No-ASE
DPM2	9	130698029		0	0	0															0			No-ASE
SLC25A25	9	130854358						0																No-ASE
PTGES2	9	130884723												0			0							No-ASE
CIZ1	9	130928633		0	0	0				0	0		0											No-ASE
GOLGA2	9	131019738														0							0	No-ASE
TRUB2	9	131071839									0													No-ASE
COQ4	9	131088131			0																			No-ASE
SLC27A4	9	131107439										0		0										No-ASE
CERCAM	9	131185203											0	0										No-ASE
ODF2	9	131217494												0										No-ASE
GLE1	9	131267089		0		0									0	0	0				0		0	No-ASE
PKN3	9	131467744											0	0										No-ASE
ZDHHC12	9	131483486		0								0											0	No-ASE
TBC1D13	9	131565554		0		0		0				0	0											No-ASE
C9orf114	9	131585069		0		0	0	0				0	0											No-ASE
NUP188	9	131710017								0							0							No-ASE
FAM73B	9	131804718				0																		No-ASE
DOLPP1	9	131846957		0			0						0		0		0							No-ASE
PPP2R4	9	131891373								0			0				0							No-ASE
C9orf50	9	132374678		0	0					0						0					0			No-ASE
TOR1A	9	132576364									0													No-ASE
USP20	9	132618599			0			0			0		0	0										No-ASE
FNBP1	9	132662721			0						0		0								0			No-ASE
ABL1	9	133729499						0					0											No-ASE
LAMC3	9	133884891				1	0																	Heterogeneous_ASE
NUP214	9	134001105									0	0			0	0								No-ASE
PRRC2B	9	134305632			0			0		0	0		0		0	0					0		0	No-ASE
POMT1	9	134382859									0					1							1	Heterogeneous_ASE
UCK1	9	134401335																			0			No-ASE
SETX	9	135139669			0			0				0		0			0						0	No-ASE
TTF1	9	135251366			0			0				0		0										No-ASE
DDX31	9	135470281		0	0				0	0			0		0						0			No-ASE
TSC1	9	135771835										0		0	0						0			No-ASE
CEL	9	135940433				0																		No-ASE
RALGDS	9	135974054								0				0										No-ASE
GBGT1	9	136029007										0											1	Heterogeneous_ASE
ABO	9	136131069					0	0		0	0		0	0	0	0							0	No-ASE
SURF6	9	136198734									0	0	0	0										No-ASE
SURF2	9	136223541									0	0	0	0										No-ASE
C9orf96	9	136245917				0	0						0											No-ASE
ADAMTS13	9	136287582		0		0	0				0	0	0	0		1	0				0			No-ASE
CACFD1	9	136325282				0											0				0			No-ASE
SLC2A6	9	136337168			0	0				0	0	0		0			0							No-ASE
ADAMTSL2	9	136401892		0			0	1			0												0	No-ASE
DBH	9	136501569												0										No-ASE
SARDH	9	136531857												0		0								No-ASE
VAV2	9	136633569		0		0			0	0		0	1	0		1	0						0	No-ASE
BRD3	9	136898797										0												No-ASE
COL5A1	9	137582841												0									1	Heterogeneous_ASE
FCN2	9	137772678										1												ASE
PPP1R26	9	138376372		0													0						0	No-ASE
MRPS2	9	138392871																					0	No-ASE
OBP2A	9	138439723						1																ASE
GLT6D1	9	138516047										1												ASE
KCNT1	9	138594163																					1	ASE
UBAC1	9	138830083		0			0							0		0							0	No-ASE
NACC2	9	138903547				0																		No-ASE
QSOX2	9	139100608		0	0						0	0		0							0		0	No-ASE
GPSM1	9	139230523		0			0	0								0					0			No-ASE
DNLZ	9	139256468		0							0		0		0									No-ASE
CARD9	9	139258794		0	0	1				0	0		1			1					0			Heterogeneous_ASE
SNAPC4	9	139270830		0	0	0				0	0		0			0	0				0			No-ASE
SDCCAG3	9	139297277									0		0		0									No-ASE
INPP5E	9	139323311			0	0	0		0		0		0			0					0		0	No-ASE
SEC16A	9	139340155			0	0	0				0		0	0	0	0	0				0		0	No-ASE
NOTCH1	9	139390543				0							0	0	0	0					0			No-ASE
EGFL7	9	139563043				0		0															0	No-ASE
LCN10	9	139634458												1										ASE
LCN15	9	139656670				0																	1	Heterogeneous_ASE
KIAA1984	9	139693563		0				0		0		0	0	0			0							No-ASE
RABL6	9	139726219		0								0		0									0	No-ASE
PHPT1	9	139743890						0																No-ASE
MAMDC4	9	139746869				0						0												No-ASE
C8G	9	139839839		0		0						0				1							1	Heterogeneous_ASE
LCN12	9	139847446												0										No-ASE
LCNL1	9	139878950		1																				ASE
ABCA2	9	139902962		0	0	0		0		0				0		0	0				0		0	No-ASE
ENTPD2	9	139943159																			0			No-ASE
UAP1L1	9	139973090		0		0						0			0	0	0				0		0	No-ASE
DPP7	9	140005147			0	0	0				0		0	0		0	0							No-ASE
NDOR1	9	140108261			0								0											No-ASE
SLC34A3	9	140127247			0		0		0	0	0				0	0							1	No-ASE
LOC100129722	9	140146219		0	0																			No-ASE
EXD3	9	140201415				0			0	0	0	0	0				0						0	No-ASE
NOXA1	9	140320715							0		0			0			0						0	No-ASE
ENTPD8	9	140329373		0										0			0							No-ASE
PNPLA7	9	140355188				0		0				0		0	0		0				0			No-ASE
ARRDC1	9	140507366															0							No-ASE
EHMT1	9	140605429												0			0							No-ASE
CACNA1B	9	140772613			1				1			1												ASE
GTPBP4	10	1034433					0											0						No-ASE
IDI2	10	1065603			0		0	0						0		0		0						No-ASE
ADARB2	10	1229282																0						No-ASE
PFKP	10	3143161		0			0					0	0			0	0	0	0	0	0			No-ASE
PITRM1	10	3180227	0	0	0		0			0	0	0	0	0		0	0	0	0	0	0		0	No-ASE
AKR1E2	10	4872930		1				0				0	0			0	1			0				Heterogeneous_ASE
AKR1C2	10	5040936	0																					No-ASE
AKR1C3	10	5136654						0								0					0			No-ASE
AKR1C4	10	5247754							1			1						0						Heterogeneous_ASE
UCN3	10	5415798					0	0									1	0						No-ASE
CALML5	10	5541032						0				1												Heterogeneous_ASE
FAM208B	10	5762725	0	0				0	0		0	0				0	0	0		0				No-ASE
ANKRD16	10	5920121																	0	0			0	No-ASE
IL15RA	10	5995147			0		0	0			0	0	0	0		0		0						No-ASE
PRKCQ	10	6470255					0											0						No-ASE
ITIH5	10	7605101																		0				No-ASE
TAF3	10	7866419	0		0				0			0		0			0				0		0	No-ASE
ECHDC3	10	11789355		1																			1	ASE
UPF2	10	11998413						0				0		0		0	0			0			0	No-ASE
DHTKD1	10	12123525	0		0		0	0				0	0	0			0	0			0		0	No-ASE
CAMK1D	10	12595329	0																					No-ASE
MCM10	10	13213015		0	0			0	0			0		0			0	0			0			No-ASE
UCMA	10	13264107									0													No-ASE
PHYH	10	13320327	0					0	0							0				0	0		0	No-ASE

DCLRE1C	10	14950496																0			0			No-ASE
MEIG1	10	15008475																				1		ASE
C10orf111	10	15138387			0											1			0	0				No-ASE
RPP38	10	15145336			0					0	0		0	0		0			0	0				No-ASE
FAM171A1	10	15255056	0								0			0			0					0	0	No-ASE
CUBN	10	16867012	0	0				1		0	0	0	0	0			0	0			0	0	0	No-ASE
TRDMT1	10	17191129		0	0							0	0				0				0			No-ASE
PTPLA	10	17636242			0			0			0	0		0		0	0		1		0			No-ASE
MRC1	10	17865421	0				0		0					0		1						0	0	No-ASE
SLC39A12	10	18242311	0				0							0				0						No-ASE
ARL5B	10	18964097					0										0			0				No-ASE
PLXDC2	10	20106075	0	0	0				0			0						0						No-ASE
NEBL	10	21074455	0	0	0		0	0	0		0	0	0	0			0			0				No-ASE
EBLN1	10	22498209																0						No-ASE
COMMD3	10	22605398							0						0									No-ASE
PIP4K2A	10	22830757		0						0	0	0	0	0	0								0	No-ASE
ARMC3	10	23248457										1						0						Heterogeneous_ASE
PTF1A	10	23481467					0																	No-ASE
KIAA1217	10	24498169		0			0		1		0			0	0	0		0		0			0	No-ASE
ENKUR	10	25273675																0						No-ASE
GPR158	10	25509981	0				0	0	0		0		0	0	0		0	0			0	0	0	No-ASE
MYO3A	10	26224759					0								0									No-ASE
ABI1	10	27044667														0								No-ASE
ANKRD26	10	27301840		0								0				1	0	0	0	0			0	No-ASE
YME1L1	10	27406513																	0	0				No-ASE
MASTL	10	27444425																		0				No-ASE
ACBD5	10	27493435					0																	No-ASE
PTCHD3	10	27687225																0						No-ASE
RAB18	10	27798805																	0					No-ASE
MKX	10	27964273									1													ASE
ARMC4	10	28101455									1	1						1						ASE
MPP7	10	28343067		0	0			0								0				0		0		No-ASE
SVIL	10	29747261		0	0			0	0	0	0	0			0	0		0	0			0	0	No-ASE
KIAA1462	10	30315041	0	0			0	0		0		0				0	0	0			0	0	0	No-ASE
MTPAP	10	30602551							0							0	0			0	0	0		No-ASE
ZNF438	10	31134010								0		0												No-ASE
ARHGAP12	10	32099648		0				0					0											No-ASE
EPC1	10	32560779								0			0									0		No-ASE
CCDC7	10	32740611		0		0		0				0						0				0		No-ASE
C10orf68	10	32974954		0								0										0		No-ASE
ITGB1	10	33195991										0												No-ASE
NRP1	10	33469144	0										0				0	0				0		No-ASE
PARD3	10	34408649									0													No-ASE
ZNF248	10	38121242						0									0						0	No-ASE
ZNF37BP	10	43014780	0		0								0			0		0		0	0			No-ASE
ZNF33B	10	43088356	0		0	0												0	0					No-ASE
BMS1	10	43281013	0					0			0	0	0	0						0	0		0	No-ASE
RET	10	43595999					0						0											No-ASE
ZNF239	10	44052395	0		0								1			0		0	0	0	0		0	No-ASE
ZNF485	10	44104090	0	0		0				0	0								0	0			0	No-ASE
RASSF4	10	45465678			0			0				0				0								No-ASE
C10orf25	10	45495863		0			0	0	0	0	0	0	0					0					0	No-ASE
ZNF22	10	45498875						0				0	0											No-ASE
ALOX5	10	45877946				0																		No-ASE
mrt-08	10	45953767				0						0		0		0				0	0		0	No-ASE
ZFAND4	10	46111039												0										No-ASE
GPRIN2	10	46998955																	0					No-ASE
PPYR1	10	47086901						0				0												No-ASE
ZNF488	10	48370644			0								0							0	0		0	No-ASE
FRMPD2	10	49392828					0											0						No-ASE
WDFY4	10	49917865				0	0											0			0			No-ASE
VSTM4	10	50224766					0																	No-ASE
FAM170B-AS1	10	50339754					0											0						No-ASE
C10orf128	10	50369666													0									No-ASE
C10orf71	10	50530621						0										0						No-ASE
ERCC6	10	50666949				0					0		0	0		0			0		0	0	0	No-ASE
CHAT	10	50818874					0											0						No-ASE
C10orf53	10	50901917					0											0						No-ASE
OGDHL	10	50943323						1		1		0		1				0					0	Heterogeneous_ASE
NCOA4	10	51568378	0	0	0	0					0		0					0	0	0			0	No-ASE
TIMM23	10	51592523				0								0						0			0	No-ASE
SGMS1	10	52103414	0				0				0		0		0				0		0	0		No-ASE
A1CF	10	52566611												0										No-ASE
MBL2	10	54528016					0	0										0						No-ASE
PCDH15	10	55566406																0						No-ASE
MTRNR2L5	10	57359697			1					1	1	0									1		1	ASE
ZWINT	10	58118339																				0		No-ASE
BICC1	10	60273034									1			1	0						0			Heterogeneous_ASE
SLC16A9	10	61412559				0		0				0			0	0						0		No-ASE
CCDC6	10	61552502	0									0								0				No-ASE
ANK3	10	61802453		0		0		0			0	0		0				0				0	1	No-ASE
RTKN2	10	63957780			0	0	0	0				0		0		0				0	0			No-ASE
ZNF365	10	64136038	0					0				0		1		0		0		0	0		0	No-ASE
ADO	10	64564892			0			0			0		0	0							0			No-ASE
JMJD1C	10	64927823	0	0	0		0	0		0	0	0		0							0	0	0	No-ASE
CTNNA3	10	67680203										1						0						Heterogeneous_ASE
SIRT1	10	69644820																					0	No-ASE
MYPN	10	69881254	0		0		0	0						0	0	0					0			No-ASE
PBLD	10	70044031	0		0							0	0	0										No-ASE
HNRNPH3	10	70098948				0					0		0		0						0	0		No-ASE
TET1	10	70332271		0	0		0	1		0		0											0	No-ASE
STOX1	10	70641860	0				0	0						1	0	0								No-ASE
SUPV3L1	10	70940052																	0					No-ASE
HKDC1	10	70986996	0		0		0			0				0	0	0		0		0	0		0	No-ASE
TACR2	10	71164595	0				0				0								1					No-ASE
NEUROG3	10	71332204											1					0						Heterogeneous_ASE
COL13A1	10	71562415	0																					No-ASE
AIFM2	10	71873987					0					0											0	No-ASE
TYSND1	10	71899693	0					0		0				0				0			0			No-ASE
NPFFR1	10	72014967	0																0					No-ASE
NODAL	10	72192820				0	0			0		0		0	0			0	0		0			No-ASE
PALD1	10	72285816	0	0	0		0			0	0		0			0					0		0	No-ASE
ADAMTS14	10	72434374		0									0		0			0	1					No-ASE
TBATA	10	72531154										0												No-ASE
SGPL1	10	72604246					0			0													0	No-ASE
UNC5B	10	72972771		0															0	0				No-ASE
SLC29A3	10	73082563	0	0	0	0		0		0	0	0	0	0		0		0				0	0	No-ASE
CDH23	10	73199595	0	0	0	0		1			0	1	1	0	0	1		0	0	0	0	0	1	Heterogeneous_ASE
ECD	10	74894375																	0					No-ASE
DNAJC9-AS1	10	75010635																	0					No-ASE
TTC18	10	75037991																	1					ASE
MSS51	10	75184364											0						0					No-ASE
PPP3CB	10	75197051								0														No-ASE
SEC24C	10	75519510	0											0										No-ASE
C10orf55	10	75671356																	0					No-ASE
VCL	10	75832578																					0	No-ASE
DUSP13	10	76854462	0				0					0						0				0		No-ASE
C10orf11	10	77795801										1												ASE

KCNMA1	10	78646536																				0	No-ASE
DLG5	10	79552206	0	0	0	0					0		0	0	0			0	0		0	0	0	No-ASE
POLR3A	10	79737283																				0		No-ASE
RPS24	10	79799959																	0					No-ASE
SFTPD	10	81697697	0		0	0						0						0		0		0		No-ASE
ANXA11	10	81917431				0					0		0		0			0		0		0		No-ASE
DYDC2	10	82122269																1						ASE
SH2D4B	10	82298106		1							0			0					1	0				Heterogeneous_ASE
CDHR1	10	85955312			0		0								0				0					No-ASE
CCSER2	10	86130899		0			0	0										0		0		0		No-ASE
GRID1	10	87373271	1				0																1	Heterogeneous_ASE
WAPAL	10	88206141		0							0				0							0		No-ASE
OPN4	10	88414554					0											0						No-ASE
LDB3	10	88428502				0																		No-ASE
MMRN2	10	88696622			0		0						0			0		0		0				No-ASE
MINPP1	10	89264860											0											No-ASE
PAPSS2	10	89468953												0					0					No-ASE
RNLS	10	90034737					0						0			0				0			0	No-ASE
LIPM	10	90562705					0						0	0						1		0	0	No-ASE
ANKRD22	10	90582744																	0					No-ASE
STAMBPL1	10	90665270	0			0					0			0									0	No-ASE
FAS	10	90762801																	0					No-ASE
LIPA	10	90974776	0	0							0	0		0		0			0	0		0	0	No-ASE
IFIT2	10	91065949	0	0	0		0	0			0		0		0			0		0		0	0	No-ASE
IFIT1	10	91162040				0																		No-ASE
SLC16A12	10	91193081												1										ASE
PANK1	10	91359190		0										0										No-ASE
KIF20B	10	91465118	0	0	0	0		0			0	0	0	0		0		0	0	0		0	0	No-ASE
RPP30	10	92631775				0												0						No-ASE
LOC100188947	10	93170250										0												No-ASE
FGFBP3	10	93667966				0	0					0		0	0				0	0				No-ASE
CPEB3	10	93811989		0	0																			No-ASE
EXOC6	10	94594565		0	0		0				0			0				0		0				No-ASE
CYP26C1	10	94821204		0																				No-ASE
MYOF	10	95069867	0	0				0				0		0					0				0	No-ASE
CEP55	10	95259878		0	0	0	0	0				0							0			0		No-ASE
FFAR4	10	95326619												1										ASE
RBP4	10	95353604									0													No-ASE
PDE6C	10	95372685					0																	No-ASE
FRA10AC1	10	95430585	0	0				0			0	0	0		0	0				0		0	0	No-ASE
PLCE1	10	95791030	0				0	0			0	0			0	0				0			0	No-ASE
NOC3L	10	96093965	0								0	0	0		0	0			0			0	0	No-ASE
CYP2C18	10	96447920									1	0			0								0	No-ASE
CYP2C8	10	96797033		0										1										Heterogeneous_ASE
C10orf129	10	96954252	0	1		0	0					0	0	0					0				0	No-ASE
SORBS1	10	97074865																				0		No-ASE
ALDH18A1	10	97366572											0											No-ASE
TCTN3	10	97440282									0													No-ASE
LOC728558	10	97687049	0											0						0				No-ASE
ZNF518A	10	97916272		0	0									0								0		No-ASE
BLNK	10	97959905																	0					No-ASE
TLL2	10	98127968											1	0		0								Heterogeneous_ASE
PIK3AP1	10	98362062	0	0	0	0		0				0	0			0				0		0	0	No-ASE
C10orf12	10	98741206		0			0	0								0			0	0				No-ASE
SLIT1	10	98761008																0						No-ASE
ARHGAP19-SLIT1	10	99003741				0					0													No-ASE
RRP12	10	99116903	0		0	0					0	0	0		0	0		0	0	0		0		No-ASE
MMS19	10	99218456	0		0	0							0	0	0					0			0	No-ASE
HOGA1	10	99344645			0	0					0							0				0	1	No-ASE
PI4K2A	10	99400525			0																			No-ASE
AVPI1	10	99439454	0	0	0						0	0	0			0			0	0		0		No-ASE
ZFYVE27	10	99498234	0				0				0			0		0				0		0	0	No-ASE
R3HCC1L	10	99967935	0	0	0	0	0					0	0	0				0		0		0		No-ASE
LOXL4	10	100008694	0	0	1	0	0					0				0				1			0	No-ASE
PYROXD2	10	100143572				0	0	0			0	0	1	0	1				1			0	0	Heterogeneous_ASE
HPS1	10	100177358		0		0						0		0					0	0			0	No-ASE
HPSE2	10	100219341		0			0															1		Heterogeneous_ASE
CNNM1	10	101090123				1																		ASE
ENTPD7	10	101421279									0				0	0			0	0		0	0	No-ASE
COX15	10	101473218		0		0		0				0		0		0		0	0	0			0	No-ASE
ABCC2	10	101544387										0		0		0						0		No-ASE
DNMBP	10	101637089			0	0	0					0		0		0		0	0	0		0	0	No-ASE
ERLIN1	10	101911920	0			0		0			0					0		0		0			0	No-ASE
CHUK	10	101950636	0	0	0	0		0			0							0		0			0	No-ASE
CWF19L1	10	101993024		0							0													No-ASE
PKD2L1	10	102048184		0	1													0					1	Heterogeneous_ASE
SCD	10	102107997			0			0				0	0	0		0			0	0		0	1	No-ASE
SEC31B	10	102247408	0				0					0	0	0				0						No-ASE
HIF1AN	10	102295836	0					0					0	0		0							0	No-ASE
SEMA4G	10	102732739		0		0	0	0			0	0	0		0	0		0					0	No-ASE
C10orf2	10	102748040											0									0		No-ASE
PDZD7	10	102768234					0					0		0					0			0	1	No-ASE
KAZALD1	10	102822575		0	0	0		0			0	0			0								1	No-ASE
POLL	10	103339424		0							0		0					0		0			0	No-ASE
DPCD	10	103360560		0							0		0		0			0					0	No-ASE
NPM3	10	103541595																0						No-ASE
PPRC1	10	103899047			0																		0	No-ASE
NOLC1	10	103916792			0	0		0			0	1	0						0	0			1	No-ASE
ELOVL3	10	103986319		0									0			1								Heterogeneous_ASE
GBF1	10	104117875		0		0									0								0	No-ASE
SUFU	10	104263921		0																				No-ASE
ARL3	10	104436641														0								No-ASE
WBP1L	10	104536151	0			0		0				0					0	0		0		0	0	No-ASE
C10orf32-AS3MT	10	104622644	0	1								0						0						No-ASE
NT5C2	10	104849450				0		0																No-ASE
LOC729020	10	105005891			1																			ASE
PDCD11	10	105160184	0				0	0						0	0	0		0	0	0				No-ASE
CALHM2	10	105207196	0			0																		No-ASE
CALHM1	10	105215062		0																				No-ASE
CALHM3	10	105233110	0		0																	0		No-ASE
NEURL	10	105254197																				0		No-ASE
SH3PXD2A	10	105361679		0				0			0		0	0				0		0			1	No-ASE
OBFC1	10	105642449					0				0					0			0				0	No-ASE
SLK	10	105727558	0			0																0		No-ASE
COL17A1	10	105792021	0	0	0	0	0				0		0	0	0			0	0	0		0		No-ASE
SFR1	10	105882765											0									0		No-ASE
WDR96	10	105891927	0											0						0				No-ASE
GSTO1	10	106014706			0	0					0			0	0					0				No-ASE
GSTO2	10	106034982			0	0	0				1			0	0	0			1					No-ASE
CCDC147	10	106118360													0							0		No-ASE
RBM20	10	112540884																0						No-ASE
PDCD4	10	112641053												0		0								No-ASE
GPAM	10	113913222	0	0		0	0	0						0	0	0		0	0			0	0	No-ASE
ACSL5	10	114136154		0		0													0					No-ASE
VTI1A	10	114220306	0															0						No-ASE
NRAP	10	115349462	0	1	1	0	0	0				0			0			0	0			1		Heterogeneous_ASE
CASP7	10	115451818		0				0			0		0		0					0		0		No-ASE
PLEKHS1	10	115527229		0	0		0						0						0					No-ASE

DCLRE1A	10	115594990		0			0						0	0								0		No-ASE
NHLRC2	10	115614750			0		0	0			0		0		0	0		0				0		No-ASE
C10orf118	10	115884927									0	0												No-ASE
VWA2	10	116008464	0	0	0						0	0	0	0	0			0					0	No-ASE
AFAP1L2	10	116056852	0			0													0					No-ASE
ABLIM1	10	116196048	0	0	0	0										0				0			0	No-ASE
FAM160B1	10	116590688																				0		No-ASE
TRUB1	10	116698112		0	0		0	0			0		0						0	0			0	No-ASE
PNLIPRP1	10	118351375	0																	1				Heterogeneous_ASE
PNLIPRP2	10	118385551				0													1					Heterogeneous_ASE
PDZD8	10	119042867	0				0				0											0	0	No-ASE
FAM45A	10	120863692																	0					No-ASE
GRK5	10	121086097	0	0								0		0									0	No-ASE
BAG3	10	121411212	0	0		0					0			0				0		0		0	0	No-ASE
INPP5F	10	121551143	0		0	0					0			0	0	0		0	0	0				No-ASE
SEC23IP	10	121652427	0		0	0					0			0	0	0		0	0	0				No-ASE
MIR5694	10	122610998	0					0								0		0						No-ASE
TACC2	10	123809983	0	0	0	0	0	0			0	0	0	0	0			0	0	0		0	0	No-ASE
BTBD16	10	124034607	0		1	0	0				0	0		0		0		0	0			0	0	No-ASE
PLEKHA1	10	124166131	0									0			0	0			0	0			0	No-ASE
DMBT1	10	124320300																	0					No-ASE
CUZD1	10	124591832		0							0											0		No-ASE
FAM24B-CUZD1	10	124608914	0					0			0		0						1	1		0	0	No-ASE
PSTK	10	124740113	0					0			0									0		0	0	No-ASE
ACADSB	10	124793921				0		0												0				No-ASE
CHST15	10	125769672						0					0	0				0				0	0	No-ASE
LHPP	10	126172721		0		0							0											No-ASE
CTBP2	10	126678241	0		0						0	0	0	0	0	0			0	0		0		No-ASE
TEX36	10	127344489					0																	No-ASE
C10orf137	10	127408432				0					0			0				0						No-ASE
MMP21	10	127455290					0																	No-ASE
BCCIP	10	127512136													0									No-ASE
DOCK1	10	128788749	0		0	0	0	0				0	0			0		0	0	0		0	0	No-ASE
CLRN3	10	129676520											0										0	No-ASE
MKI67	10	129899523	0	0		0		0				0	0	0	0	0	0	0	0	0			0	No-ASE
MGMT	10	131334541			0			0				0				0		0						No-ASE
GLRX3	10	131959150		0		0										0		0						No-ASE
FLJ46300	10	133608237					0											0						No-ASE
PPP2R2D	10	133748056	0			0	0				0	0	0	0				0						No-ASE
JAKMIP3	10	133930971				1																		ASE
STK32C	10	134021526						0			1													Heterogeneous_ASE
LRRC27	10	134147120		0	0	0		0						0		0							0	No-ASE
PWWP2B	10	134218492	0					0																No-ASE
INPP5A	10	134459388				0	0				0	0				0				0				No-ASE
TTC40	10	134622002		1		0	0	1				1	1	1				0					1	Heterogeneous_ASE
KNDC1	10	134974003		0	0						0			0				0					1	No-ASE
ADAM8	10	135076676				0		0			0	1		0		0			0				1	No-ASE
PRAP1	10	135164930																	0					No-ASE
ECHS1	10	135180455				0								0		0		0	0	0				No-ASE
PAOX	10	135193551										0							1					Heterogeneous_ASE
CYP2E1	10	135342034																	0					No-ASE
SYCE1	10	135367823												0										No-ASE
LOC653486	11	193146																				0		No-ASE
ODF3	11	197350															0							No-ASE
SIRT3	11	218848		0			0	0	0			0					0		0			0	0	No-ASE
PSMD13	11	237087	0			0					0		0	0	0					0	0		0	No-ASE
ATHL1	11	290494						0	0															No-ASE
B4GALNT4	11	372138	0	0		0			0		0			0		0			0	0				No-ASE
SIGIRR	11	405958	0					0	0			0		0		0								No-ASE
ANO9	11	418421	0	0	0	0		0			0			0	0		0			0				No-ASE
RNH1	11	494731															0							No-ASE
LRRC56	11	540719	0	0										0		0				0	0			No-ASE
C11orf35	11	555075	0											0										No-ASE
RASSF7	11	561787							0											0		0		No-ASE
PHRF1	11	587343	0				0	0	0			0		0	0					0		0		No-ASE
IRF7	11	613087												0						0	0			No-ASE
CDHR5	11	617452	0					0						0	0					0	0			No-ASE
TMEM80	11	700181				0																		No-ASE
EPS8L2	11	709567														0								No-ASE
PIDD	11	800186			0	0		0	0			0		0									0	No-ASE
PNPLA2	11	821676		0											0									No-ASE
EFCAB4A	11	828764		0		0					0								0	0				No-ASE
CHID1	11	870141		0	0	0					0		0	0									0	No-ASE
AP2A2	11	959457	0		0		0	0			0		0	0		0	0		0		0		0	No-ASE
MUC6	11	1013929	0					0	0								1						0	No-ASE
MUC2	11	1074927				0	0						0	0	0	0			0	0	0	0	0	No-ASE
MUC5B	11	1246941	0			0		0	0	1	1	0	0			1	0			0				Heterogeneous_ASE
MOB2	11	1491427	0									0	0						0				0	No-ASE
SYT8	11	1855821	0	0	0	0		0				0							0	0	0		0	No-ASE
LSP1	11	1874388		1																		0		Heterogeneous_ASE
MRPL23	11	1972143																					0	No-ASE
INS-IGF2	11	2154293					0																	No-ASE
TH	11	2185482					0	0	0						0									No-ASE
TSPAN32	11	2323396										0												No-ASE
TRPM5	11	2426215	0		0	0	0				0			0		0				0	0		0	No-ASE
SLC22A18AS	11	2909513	0	0			0	0	0			0		0		0					0	0	0	No-ASE
SLC22A18	11	2929502						0	0		0									0		0		No-ASE
CARS	11	3022456				0																		No-ASE
OSBPL5	11	3109546				0		0														0	0	No-ASE
MRGPRE	11	3249219					0																	No-ASE
ZNF195	11	3380855		0																				No-ASE
ART5	11	3659982				0																		No-ASE
CHRNA10	11	3687429						0						0										No-ASE
NUP98	11	3697438						0						0	0					0				No-ASE
OR52K1	11	4510186				0								0										No-ASE
OR51D1	11	4661190																	0					No-ASE
OR51E1	11	4673788			0												1							Heterogeneous_ASE
OR51F1	11	4790214		1									0		1									Heterogeneous_ASE
OR51F2	11	4842656																0						No-ASE
OR51A7	11	4928621					0																	No-ASE
OR51G2	11	4935975				0	0																	No-ASE
OR51G1	11	4944620					0																	No-ASE
OR51L1	11	5020241																				1		ASE
OR52J3	11	5067816		1																				ASE
OR52E2	11	5080059			1				1															ASE
OR51B4	11	5322249	1	1		1								1	0		0					0	1	Heterogeneous_ASE
OR51B2	11	5344592																		1				ASE
OR51B5	11	5364013	1	1	0	1	0	0	0		0			0	0	0	1		1	1		0	1	Heterogeneous_ASE
UBQLN3	11	5528864																				0		No-ASE
UBQLNL	11	5536279				0			0							0	0					0	0	No-ASE
OR52H1	11	5565906	0				0	0			1											0		No-ASE
OR52B6	11	5602135	0	0	0	0	0	0	0		0			0			0		0				1	No-ASE
TRIM6	11	5624495																					1	ASE
TRIM6-TRIM34	11	5653978		0								0				0							0	No-ASE
TRIM5	11	5686085		0	0				0			0			0	0	0						0	No-ASE
TRIM22	11	5717482			0			0				0				0						0		No-ASE
OR52N4	11	5776044		0	0				0								0					0		No-ASE
OR52N2	11	5841650							1															ASE

OR56B4	11	6129082					0																	No-ASE
OR52B2	11	6190634		0													0							No-ASE
OR52W1	11	6220605		0	0	0	0					0		0			0			0		0	0	No-ASE
FAM160A2	11	6232752		0	0			0					0	0	0					0		0	0	No-ASE
CNGA4	11	6261307												0		0				0				No-ASE
PRKCDBP	11	6340416			0		0				0													No-ASE
SMPD1	11	6411836		0	0		0	0	0				0	0						0				No-ASE
DNHD1	11	6519453	0	0	0	0	0	0	0		0	0	0	0	0	0	0			0			0	No-ASE
RRP8	11	6622162			0								0											No-ASE
ILK	11	6625566					0																	No-ASE
DCHS1	11	6643110		1	1	1			1		0	1	1	0			1					0		Heterogeneous_ASE
OR2AG2	11	6789257	0	0					0		0	0		0		0	0		0	1		0		No-ASE
OR2AG1	11	6806366		1					0			1		0						1				Heterogeneous_ASE
OR6A2	11	6816036	0	1				0					0		0	1	0							Heterogeneous_ASE
OR10A2	11	6891368	0	1				0	0		0	1	0	0	1		0		1				0	Heterogeneous_ASE
OR2D2	11	6912827	0	1	1			0	0		0	1	1	0	0		0		1	1		0	0	Heterogeneous_ASE
OR2D3	11	6942395	0	1		0	0				0	0		0	0	1	1		1			0	0	Heterogeneous_ASE
ZNF215	11	6953597	0	1	1	0					0	0	0	0	0		0		1	0		0	0	No-ASE
ZNF214	11	7021115	0			0	0				0		0		0		0		1				0	No-ASE
NLRP14	11	7059825				0					0				1							0		No-ASE
SYT9	11	7324581					0																	No-ASE
OLFML1	11	7507122		0		0						0					0			0		0	0	No-ASE
CYB5R2	11	7686612	0	0				0							0	0			0					No-ASE
OVCH2	11	7712523										0												No-ASE
OR10A6	11	7949293														0								No-ASE
RIC3	11	8129296										0												No-ASE
STK33	11	8414061	0									1		0			1							Heterogeneous_ASE
TRIM66	11	8639200	0			0	0	0				0	0	0		0	0		0			0		No-ASE
ST5	11	8718154				0		0	0			0	0		0	0	0			0	0	0		No-ASE
AKIP1	11	8933004	0					0				0	0				0		0		0			No-ASE
C11orf16	11	8942867										1		0	1		1		0					Heterogeneous_ASE
ASCL3	11	8959255						0							0									No-ASE
SCUBE2	11	9047261	0			0	0					0		0			0			0				No-ASE
ZNF143	11	9494245	0														0							No-ASE
SWAP70	11	9685741	0	0				0	0			0											0	No-ASE
SBF2	11	9834123		0				0	0		0									0	0			No-ASE
AMPD3	11	10477939									0		0	0						0			0	No-ASE
RNF141	11	10536298		0		0																0	0	No-ASE
MRVI1	11	10622544																		0		0		No-ASE
CTR9	11	10785697																	0					No-ASE
ZBED5	11	10874853		0							0									0				No-ASE
GALNT18	11	11292768				0																		No-ASE
USP47	11	11906050	0		0			0			0	0			0				0		0		0	No-ASE
DKK3	11	11986051	0	0	0										0						0			No-ASE
MICAL2	11	12183737									0			0										No-ASE
MICALCL	11	12313734		0	0	0		0			0					0			0	0			1	No-ASE
TEAD1	11	12903443	0	0		0			0					0		0	0		0					No-ASE
RASSF10	11	13031880											0											No-ASE
SPON1	11	14096939		1		0	0	0	0		0	1			0		0						1	Heterogeneous_ASE
INSC	11	15134023				0		0			0				0									No-ASE
PLEKHA7	11	16810755		0					0			0		0			0		0	0				No-ASE
PIK3C2A	11	17111340	0			0								0										No-ASE
NUCB2	11	17317668	0	0				0				0			0		0		0	0	0	0		No-ASE
NCR3LG1	11	17378308			0	0	0		0		0		0			0	0		0	0		0	0	No-ASE
KCNJ11	11	17408485	0	0		0		0			0	0			0				0	0	0	0		No-ASE
ABCC8	11	17414570				1		1			0	0			1				0			1		Heterogeneous_ASE
USH1C	11	17515883	0						0		0		0	0		0			0	0	0	0		No-ASE
SERGEF	11	17809598																				0		No-ASE
SAAL1	11	18102014			0	0		0																No-ASE
MRGPRX3	11	18158958	0	0		0									0						0	0		No-ASE
SAA4	11	18253084									0													No-ASE
SAA2-SAA4	11	18267005																					1	ASE
HPS5	11	18303533	0									0					0							No-ASE
GTF2H1	11	18357384		0																				No-ASE
UEVLD	11	18553921											0										0	No-ASE
SPTY2D1	11	18633897		0												0						0		No-ASE
IGSF22	11	18727448	0	0	0	0						0			1	1	0		0	0	0	0	0	No-ASE
MRGPRX2	11	19076980	0																					No-ASE
ZDHHC13	11	19164670	0			0	0		0					0			0			0	0	0	0	No-ASE
CSRP3	11	19204263	0																					No-ASE
NAV2	11	19735396	0	0	0	0	0	0					0			0	0		0	0	0	0	0	No-ASE
SLC6A5	11	20622728					0																	No-ASE
NELL1	11	20699576															0							No-ASE
ANO5	11	22239808				0									1		1						0	Heterogeneous_ASE
FANCF	11	22646270											0											No-ASE
BBOX1	11	27078769													0									No-ASE
CCDC34	11	27360457	0	0				0			0	0	0								0		0	No-ASE
LGR4	11	27389449								0		0	0							0				No-ASE
BDNF-AS	11	27679649			0					0		0			0		0			0				No-ASE
BDNF	11	27720937																					1	ASE
KIF18A	11	28042799			0		0		0	0				0		0	0						0	No-ASE
METTL15	11	28129982																	0					No-ASE
DCDC5	11	30900214										0										1		Heterogeneous_ASE
PRRG4	11	32852167			0	0	0		0	0		0	0	0			0		0	0	0	0	0	No-ASE
QSER1	11	32953516			0	0		0					0	0										No-ASE
DEPDC7	11	33047270										0			0									No-ASE
TCP11L1	11	33065394	0	0	0	0	0		0		0			0	0	0	0				0	0		No-ASE
HIPK3	11	33308079																		0				No-ASE
KIAA1549L	11	33564038		0	0	0		0	0	0	0			0	0					0	0		0	No-ASE
FBXO3	11	33763476	0	0									0											No-ASE
ABTB2	11	34174032								0		0								0		0		No-ASE
CAT	11	34460472						0				0		0	0	0					0		0	No-ASE
EHF	11	34664266									0		0											No-ASE
APIP	11	34909910	0	0	0	0	0		0			0	0	0	0	0	0			0	0	0	0	No-ASE
PDHX	11	34938249		0		0														0				No-ASE
CD44	11	35198244				0			0	0	0		0	0			0			0	0			No-ASE
SLC1A2	11	35282334										0												No-ASE
PAMR1	11	35454022	0											0					0					No-ASE
PRR5L	11	36422679												0	0				0	0		0		No-ASE
RAG1	11	36594855			0				0	0						0	0				0			No-ASE
TTC17	11	43380651												0										No-ASE
HSD17B12	11	43772505	0	0					0	0				0	0		0					0		No-ASE
ALKBH3	11	43904221	0		0			0			0			0		0		0		1		0	0	No-ASE
ACCS	11	44089221		0	0	0	0	0	1					0	0	0		0	0			0		No-ASE
EXT2	11	44129270		0																		0		No-ASE
ALX4	11	44286523		0			0		0															No-ASE
CD82	11	44621780		0	0	0				0	0				0	0	0	0	0					No-ASE
TSPAN18	11	44931280		0	1		0	0		0	0			0	1	0		0						No-ASE
PRDM11	11	45117405			0			0					0					0						No-ASE
SYT13	11	45264277											1			1		0	0					Heterogeneous_ASE
CHST1	11	45671241													0									No-ASE
SLC35C1	11	45827578										0												No-ASE
GYLTL1B	11	45944542				0		0																No-ASE
PHF21A	11	45955550									0			0									0	No-ASE
CREB3L1	11	46321657			0														0					No-ASE
DGKZ	11	46367120					0				1										0		0	No-ASE
AMBRA1	11	46419263																0						No-ASE
ATG13	11	46665854		0	0	0	0			0				0		0					0			No-ASE

ZNF408	11	46722632												0										No-ASE
F2	11	46741363								0														No-ASE
LRP4-AS1	11	46880592	0	0	0	0			0	0		0		0		0	0			0	0			No-ASE
LRP4	11	46896484	0	0	0	0				0			0	0	0	0	0			0	0			No-ASE
ARFGAP2	11	47187041							0															No-ASE
ACP2	11	47261762	0		0					0		0	0	0	0	0	0				0	0	0	No-ASE
MADD	11	47290984		0	0					0	0	0	0	0	0	0	0	0	0		0		0	No-ASE
MYBPC3	11	47353626								0	0	0	0	0	0	0					0		0	No-ASE
SLC39A13	11	47431703		0	0					0		0				0	0							No-ASE
KBTBD4	11	47595131																				0		No-ASE
MTCH2	11	47640429						0		0	0		0							0			0	No-ASE
AGBL2	11	47681762	0		0		0	0	0		0					0	0	0		0	0	0		No-ASE
FNBP4	11	47741561			0					0		0		0			0							No-ASE
NUP160	11	47801897					0	0	0	0							0	0		0	0			No-ASE
PTPRJ	11	48134520		0				0	0	0	0	0	0	0		0	0			0			0	No-ASE
OR4B1	11	48238455		1										1				0						Heterogeneous_ASE
OR4X2	11	48266660							0		1		1				1			1	1		0	Heterogeneous_ASE
OR4X1	11	48285558						1										0	0					Heterogeneous_ASE
OR4C3	11	48346495				1				1		0												Heterogeneous_ASE
OR4A16	11	55110761		1			0					1					1	0						Heterogeneous_ASE
OR4C15	11	55321823																0						No-ASE
OR4C16	11	55339605		1	1		0		1								1	0						Heterogeneous_ASE
OR4C6	11	55432646			1				1		1													ASE
OR5D14	11	55563035					0											0						No-ASE
OR5L1	11	55579078																0						No-ASE
OR5F1	11	55761251																0						No-ASE
OR8J1	11	56127898																0						No-ASE
OR8U8	11	56143103												0			1	0			0			No-ASE
TNKS1BP1	11	57068494	0						0		0	0							0		0		1	No-ASE
P2RX3	11	57114074					0											0						No-ASE
SLC43A3	11	57175272															0							No-ASE
SLC43A1	11	57254616															0					0	0	No-ASE
SMTNL1	11	57310327				0			0									0						No-ASE
SERPING1	11	57365768																0						No-ASE
ZDHHC5	11	57456075																0						No-ASE
TMX2-CTNND1	11	57480167																0	0					No-ASE
OR9Q1	11	57798473				0			1		1	1						0						Heterogeneous_ASE
OR1S1	11	57982229		0	1																	1		Heterogeneous_ASE
OR10Q1	11	57995396				0	0						1											Heterogeneous_ASE
OR10W1	11	58034457																					0	No-ASE
OR5B17	11	58125600					0																	No-ASE
OR5B12	11	58206703																0						No-ASE
ZFP91	11	58377389										0												No-ASE
FAM111A	11	58916425		0													0							No-ASE
MPEG1	11	58978258			0						0				0			0		0		0		No-ASE
OR5A1	11	59210646													1					1				ASE
OR4D6	11	59224464			0				0						0									No-ASE
OR10V1	11	59480448													0									No-ASE
TCN1	11	59620464		0							0	0				0	1							No-ASE
MS4A6A	11	59939307																0						No-ASE
MS4A4A	11	60059810															0							No-ASE
MS4A6E	11	60102384															0							No-ASE
MS4A14	11	60164180															0							No-ASE
MS4A12	11	60264849				0			1															Heterogeneous_ASE
MS4A15	11	60531180													0									No-ASE
MS4A10	11	60557840													0							1		Heterogeneous_ASE
TMEM109	11	60687275							0					0										No-ASE
TMEM132A	11	60695216		0								0	0	0		0		0		0		0	0	No-ASE
SLC15A3	11	60704714											0										1	Heterogeneous_ASE
CD6	11	60739372				0	0										0	0		0				No-ASE
CD5	11	60870066	0		0		0	0	0		0	0			0	0			0	1		0	0	No-ASE
VPS37C	11	60899476			0			0	0		0	0								0		0	0	No-ASE
DDB1	11	61081819		0												0								No-ASE
DAK	11	61105458																	0				0	No-ASE
TMEM216	11	61161430										0			0				0				0	No-ASE
CPSF7	11	61179392			0																			No-ASE
PPP1R32	11	61249390				0																		No-ASE
MYRF	11	61533183				0																		No-ASE
FADS1	11	61569830					0				0				0						1			No-ASE
BEST1	11	61722627						0				0				0				0			0	No-ASE
INCENP	11	61895638		0	0	0	0		0		0	0	0	0				0	0	0	0		0	No-ASE
AHNAK	11	62259222							0		0		0				0			0		0		No-ASE
TUT1	11	62342678					0										0							No-ASE
GANAB	11	62393291		0													0							No-ASE
C11orf48	11	62433898			0									0	0									No-ASE
BSCL2	11	62457861				0	0		0				0	0		0	0							No-ASE
TMEM223	11	62559301		0	0	0		0	0								0							No-ASE
STX5	11	62591966											0											No-ASE
SLC22A24	11	62847453																0						No-ASE
SLC22A25	11	62931484					0															1		Heterogeneous_ASE
MIR3680-1	11	63057658		1			0												1			0		Heterogeneous_ASE
RTN3	11	63449125		0		0					0		0		0		0		0		0	0		No-ASE
TRPT1	11	63991581			0	0		0					0	0						0			0	No-ASE
FKBP2	11	64009879											0											No-ASE
PLCB3	11	64021930					0					0					0						0	No-ASE
GPR137	11	64053988							0															No-ASE
KCNK4	11	64060583					0																	No-ASE
TEX40	11	64067889				1		0					0	0										No-ASE
CCDC88B	11	64109118						0	0		0		0		0								1	No-ASE
SLC22A11	11	64323476		0																				No-ASE
NRXN2	11	64402743										0												No-ASE
RASGRP2	11	64496352		0			0																	No-ASE
MEN1	11	64572104							0			0				0								No-ASE
CDC42BPG	11	64594586		0				0				0	0	0	0	0				0			0	No-ASE
EHD1	11	64622073			0			0				0		0				0					0	No-ASE
ATG2A	11	64662575		0		0													0					No-ASE
BATF2	11	64756977				0								0										No-ASE
ARL2-SNX15	11	64795017				0							0											No-ASE
SAC3D1	11	64809044				0		0				0				0					0			No-ASE
NAALADL1	11	64812777		0					0													0		No-ASE
ZFPL1	11	64854012											0											No-ASE
TM7SF2	11	64880743			0	0		0	0		0	0	0	0	0				0	0		0	0	No-ASE
CAPN1	11	64950393			0	0	0							0			0			0				No-ASE
SLC22A20	11	64981366		0		0		0				0	0	0	0	0	0		0	0	0			No-ASE
POLA2	11	65029776						0					0							0				No-ASE
LTBP3	11	65307048											0						0					No-ASE
LOC254100	11	65336819											0											No-ASE
EHBP1L1	11	65346846			0				0			0	0	0			0			0		0		No-ASE
PCNXL3	11	65383859				0		0	0		0	0	0	0		0			0		0	0	0	No-ASE
AP5B1	11	65545610			0		0	0			0	0	0		0	0	0		0	0	0	0	0	No-ASE
MUS81	11	65628334		0					0			0					0				0		0	No-ASE
C11orf68	11	65685126										0							0					No-ASE
TSGA10IP	11	65713207		0		0	0		0					0	0		0		0	0			0	No-ASE
EIF1AD	11	65766041										0							0		0			No-ASE
CATSPER1	11	65784563	0				0																	No-ASE
KLC2	11	66029387											0	0	0	0			0					No-ASE
YIF1A	11	66052197			0																			No-ASE
RIN1	11	66099764			0																			No-ASE

BRMS1	11	66105194		0	0	0	0						0		0	0	0	0		0	0			No-ASE
SLC29A2	11	66130917									0													No-ASE
PELI3	11	66235631		0	0			0					0			0	0	0		0		0		No-ASE
DPP3	11	66249676		0	0	0		0					0			1	0			0		0		No-ASE
BBS1	11	66281880		0	0	0		0					0			0	0	0		0		0		No-ASE
ZDHHC24	11	66311349										0												No-ASE
ACTN3	11	66314514	0			0			1									0				0		No-ASE
SPTBN2	11	66453406						0						0						0				No-ASE
C11orf80	11	66555733				0		0				0	0	0						0				No-ASE
ANKRD13D	11	67059166											0											No-ASE
SSH3	11	67072333									0													No-ASE
RPS6KB2	11	67197026									0		0										0	No-ASE
CORO1B	11	67205869											0											No-ASE
AIP	11	67250655																	0					No-ASE
PITPNM1	11	67259518													0			0						No-ASE
CABP2	11	67287311					0																	No-ASE
GSTP1	11	67351964	0	0				0				0	0					0	0	0				No-ASE
TBX10	11	67400475						0			0								1	0	0		1	Heterogeneous_ASE
ACY3	11	67410314		1				0						0									0	No-ASE
ALDH3B2	11	67430720				0						0					0							No-ASE
ALDH3B1	11	67782795		0		0	0		0				0	0								0		No-ASE
NDUFS8	11	67799622									0													No-ASE
TCIRG1	11	67808746						0				0												No-ASE
SUV420H1	11	67925546			0																			No-ASE
C11orf24	11	68029285		0		0	0	0	0						0	0	0	0	0	0		0	0	No-ASE
LRP5	11	68115486			0			0						0		0	0			0				No-ASE
PPP6R3	11	68312310												0										No-ASE
MTL5	11	68475966										0												No-ASE
CPT1A	11	68527037		0					0														0	No-ASE
IGHMBP2	11	68673577			0	0	0	0	0		0		0	0		0	0		0	0			0	No-ASE
MRGPRF	11	68772955		0	0		0	0						0						0		0	1	No-ASE
TPCN2	11	68821507		0	0	0	0	0	0		0	0		0			0		0	0		0		No-ASE
MYEOV	11	69062880			0	0	0									0			0				1	No-ASE
CCND1	11	69456169		0		0	0	0	0		0		0						0		0			No-ASE
ORAOV1	11	69486584																	0					No-ASE
FGF4	11	69588175							1															ASE
ANO1	11	69924755		0	0	0		1				0		0			0						0	No-ASE
PPFIA1	11	70118365		0	0	0			0			0		0			0			0			0	No-ASE
CTTN	11	70260735																				0		No-ASE
SHANK2	11	70319049			0			0		0	0	0	0		0	0		0		0		0		No-ASE
NADSYN1	11	71164364			0		0		0			0	0	0	0		0	0	0		0	0	0	No-ASE
ALG1L9P	11	71524861					0																	No-ASE
RNF121	11	71640167																			0		0	No-ASE
NUMA1	11	71715129								0												0	0	No-ASE
FOLR3	11	71850130									1	1	1					0		1				ASE
PDE2A	11	72288437		1				0																Heterogeneous_ASE
ARAP1	11	72397101		0				0	0		0		0			0				0	0	0	0	No-ASE
ATG16L2	11	72527854											0			0								No-ASE
P2RY2	11	72945254			0	0	0	0	0	0	0	0	0									0	1	No-ASE
ARHGEF17	11	73020390		0	0	0		0			0		0	0										No-ASE
RELT	11	73100204																					0	No-ASE
PAAF1	11	73598401				0												0					0	No-ASE
DNAJB13	11	73662133																					1	ASE
UCP2	11	73686548			0							0	0		0			0	0	0	0			No-ASE
C2CD3	11	73748676	0		0	0		0		0	0	0	0	0	0		0	0	0	0			0	No-ASE
P4HA3	11	73978324												0	1		1	1						ASE
PGM2L1	11	74047743			0						0	0	0	0			0	0		0		0	0	No-ASE
LIPT2	11	74203256		0			0	0	0	0	0	0								0				No-ASE
POLD3	11	74329638																	0					No-ASE
XRRA1	11	74554472	0	0		0								0		0	0			0	0	0	0	No-ASE
NEU3	11	74705572									0	0	0		0									No-ASE
SLCO2B1	11	74873726			0	0								0						1				No-ASE
KLHL35	11	75133703		0								0									0			No-ASE
GDPD5	11	75148014		0							0	0		0						0			0	No-ASE
MAP6	11	75298327									0													No-ASE
MOGAT2	11	75428947				0													0	0			0	No-ASE
LRRC32	11	76370661																	0					No-ASE
TSKU	11	76506691		0				0	0		0	0	0				0						0	No-ASE
ACER3	11	76637651				0			0	0		0	0	0			0	0		0			0	No-ASE
CAPN5	11	76795984				0																	0	No-ASE
MYO7A	11	76853775		0	1	1	0	0	0		0	0			1	0	0	0	0	0	0		1	No-ASE
GDPD4	11	76928356					0																	No-ASE
AQP11	11	77301121		0							0			0				0		0	0		0	No-ASE
RSF1	11	77378210		0				0			0	0		0		0		0	0	0	0		0	No-ASE
AAMDC	11	77553595								0	0			0						0			0	No-ASE
RNU6-83	11	77812075				0		0	0	0	0	0	0			0			0	0	0			No-ASE
KCTD21	11	77882393				0				0		0		0	0	0		0	0		0			No-ASE
USP35	11	77907961		0	0			0			0			0				0					0	No-ASE
NARS2	11	78177004			0					0	0					0		0			0			No-ASE
TENM4	11	78369332																0					1	Heterogeneous_ASE
PRCP	11	82536108			0											0								No-ASE
C11orf82	11	82625793		0					0		0	0	0	0	0	0	0		0		0	0		No-ASE
PCF11	11	82875309								0		0	0						0					No-ASE
CCDC90B	11	82972960						0		0						0							0	No-ASE
SYTL2	11	85406316			0	0	0								0	0		0	0		0	0	0	No-ASE
CCDC83	11	85576197		0																				No-ASE
CCDC81	11	86103702		0																				No-ASE
ME3	11	86157366		0	0		0		0		0		0	0		0	0	0				0		No-ASE
FZD4	11	86662209														0								No-ASE
CTSC	11	88027209				0		0	0								0	0		0	0			No-ASE
CHORDC1	11	89935586		0								0												No-ASE
FAT3	11	92086264					0		1									0			0			No-ASE
CCDC67	11	93088656									0						0						1	Heterogeneous_ASE
KIAA1731	11	93400902			0	0		0				0						0	0	0			0	No-ASE
VSTM5	11	93554242														0								No-ASE
HEPHL1	11	93754679																	0					No-ASE
PANX1	11	93862493	0		0	0	0	0						0	0	0		0	0				0	No-ASE
KDM4D	11	94730711				0																		No-ASE
SRSF8	11	94800853										0												No-ASE
ENDOD1	11	94861754		0		0	0	0	0				0		0									No-ASE
CEP57	11	95523887		0					0		0	0	0	0		0				0			0	No-ASE
MAML2	11	95712527			0	0			0		0		0				0							No-ASE
CCDC82	11	96092186												0										No-ASE
ARHGAP42	11	100558520		0							0	0												No-ASE
TMEM133	11	100863055																0						No-ASE
KIAA1377	11	101815106		0													0						1	Heterogeneous_ASE
TMEM123	11	102272400						0						0										No-ASE
MMP7	11	102394024			0			0	0			0								1			1	Heterogeneous_ASE
MMP20	11	102449808			0						0					0		0		0				No-ASE
MMP27	11	102562537					0																	No-ASE
MMP8	11	102584096					0																	No-ASE
MMP10	11	102641531												0										No-ASE
WTAPP1	11	102661164																	0					No-ASE
MMP12	11	102736642							1					0										Heterogeneous_ASE
DYNC2H1	11	102984387		0	0	0			0							0	0		1				1	No-ASE
PDGFD	11	103779025				0																		No-ASE
CASP5	11	104866515				0		0			0	0		0	0					0				No-ASE
CASP1	11	104897049																	0					No-ASE

CWF19L2	11	107197640			0	0	0	0			0	0	0	0		0	0			0				No-ASE
ALKBH8	11	107375422		0										0										No-ASE
ACAT1	11	107992346			0	0						0			0	0	0	0		0				No-ASE
NPAT	11	108031926	0	0	0	0	0	0	0		0	0	0			0	0	0	0	0			0	No-ASE
ATM	11	108098555		0												0		0		0				No-ASE
KDELC2	11	108345575													0									No-ASE
EXPH5	11	108380281		0	0	0		0	0		0	0		0	0	0	0	0	0				0	No-ASE
C11orf88	11	111385534							0															No-ASE
PPP2R1B	11	111597693																	0				0	No-ASE
ALG9	11	111657184		0				0									0			0	0			No-ASE
FDXACB1	11	111745702										0	0											No-ASE
HSPB2-C11orf52	11	111783582		0		0		0									0			0	0			No-ASE
DLAT	11	111896242		0		0		0									0		0	0	0			No-ASE
PIH1D2	11	111941312			0							0												No-ASE
C11orf34	11	112119652		0		0		0							0	1	0	0		0				No-ASE
NCAM1	11	112832353				1						0			1			0			0			Heterogeneous_ASE
TTC12	11	113186986		0			0	0	0		0		0	0	0	0		0	0	0				No-ASE
ANKK1	11	113258719	0			0	0				0			0			0	0	1					No-ASE
DRD2	11	113283337										0												No-ASE
TMPRSS5	11	113560969			0	0	0	0					0	0						0	0		1	No-ASE
NXPE1	11	114392694					0	0	0			0		0			0		1		0		1	No-ASE
NXPE4	11	114441788			1		0		0			0	0	0			0	0			0		1	No-ASE
NXPE2	11	114568942				0		1	0		0	0		0			0						0	No-ASE
BUD13	11	116619073		0	0			0	0						0									No-ASE
ZNF259	11	116649645		0	0																			No-ASE
APOA4	11	116691621					0						1					0						Heterogeneous_ASE
PAFAH1B2	11	117038251		0		0												0			0			No-ASE
BACE1	11	117156893									0			0	0									No-ASE
CEP164	11	117222592		0		0		0	0		0	0	0	0		0	0	0	0	0	0		0	No-ASE
DSCAML1	11	117299079																0						No-ASE
TMPRSS13	11	117776363			0						0		0											No-ASE
IL10RA	11	117859104		0	1		0	0	0		0				0					0				No-ASE
TMPRSS4	11	117969787						0												0				No-ASE
AMICA1	11	118065081			0	0	0						0	0				0	0		0		1	No-ASE
MPZL3	11	118100634																	0					No-ASE
CD3G	11	118215171					0											0						No-ASE
TMEM25	11	118402869				1							1											ASE
IFT46	11	118422526									0								0		0			No-ASE
PHLDB1	11	118477367	0	0							0					1								No-ASE
TREH	11	118529017		0																				No-ASE
BCL9L	11	118769303				0						0												No-ASE
UPK2	11	118827082				1																		ASE
SLC37A4	11	118895624			0																			No-ASE
VPS11	11	118940182		0					0		0						0	0	0	0				No-ASE
DPAGT1	11	118967758	0	0			0		0			0	0		0		0		0	0			0	No-ASE
C2CD2L	11	118978479										0			0		0		0					No-ASE
HINFP	11	118997800		0																				No-ASE
NLRX1	11	119042123		0			0	0	0		0	0	0		0	0	0		0				0	No-ASE
PDZD3	11	119056865		0	0		0		0		0	0	0	0	0	1	0						0	No-ASE
CCDC153	11	119060963		0							0		0		0				0				0	No-ASE
RNF26	11	119205987				0			0		0	0	0			0	0		0					No-ASE
MFRP	11	119210380					0											0						No-ASE
USP2	11	119227916				0								0	0				0					No-ASE
PVRL1	11	119509441															0							No-ASE
TRIM29	11	119983133			0																			No-ASE
OAF	11	120097534		0			0	0					0	0	0				0	0	0		0	No-ASE
POU2F3	11	120117184									1							0						Heterogeneous_ASE
ARHGEF12	11	120280115														0								No-ASE
TECTA	11	120973430		0			0	0			0					0	0				0		1	No-ASE
SORL1	11	121323072		0					0					0				0						No-ASE
UBASH3B	11	122650402																	1					ASE
CLMP	11	122944357																					1	ASE
GRAMD1B	11	123476108			0						0						0	0	0	0			0	No-ASE
ZNF202	11	123595927		0	0			0				0	0	0		0			0				0	No-ASE
OR6M1	11	123676231																0						No-ASE
OR8D4	11	123777215							0				1		0									Heterogeneous_ASE
OR10S1	11	123847412				1			0					1									1	ASE
VWA5A	11	123988253																		0				No-ASE
OR8G2	11	124095414					0											0						No-ASE
OR8G1	11	124120819					0																	No-ASE
OR8D1	11	124179857					0											0						No-ASE
OR8B4	11	124293857																0						No-ASE
OR8A1	11	124440043				1												0						Heterogeneous_ASE
SIAE	11	124506947		0							0	0							0					No-ASE
VSIG2	11	124617442				0								0			0							No-ASE
ESAM	11	124623558			0																		0	No-ASE
ROBO3	11	124735519																0						No-ASE
ROBO4	11	124755072																0						No-ASE
HEPN1	11	124789755																0						No-ASE
CCDC15	11	124824804			0				0						0		0							No-ASE
SLC37A2	11	124933322					0				0	0				1		0						No-ASE
FEZ1	11	125322254												0										No-ASE
EI24	11	125445237					0	0	0		0	0	0		0			0	1				0	No-ASE
CHEK1	11	125503100			0									0										No-ASE
PATE1	11	125617606		0	1		0				0	1		0			0	0						No-ASE
PATE4	11	125703297						0																No-ASE
HYLS1	11	125763746	0	0			0	0			0	0	0	0	0	0	0	0	0	0	0			No-ASE
CDON	11	125828007	0	0	0	0	0	0	0		0	0	0	0	0	0	0		0	0	0		0	No-ASE
RPUSD4	11	126073336				0		0	0				0		0		0				0			No-ASE
TIRAP	11	126160814		0		0		0					0		0						0			No-ASE
DCPS	11	126173982						1																ASE
ETS1	11	128328959			0			0	0														1	No-ASE
TP53AIP1	11	128805502					0								0		0							No-ASE
ARHGAP32	11	128838845											0											No-ASE
BARX2	11	129306648			0																			No-ASE
TMEM45B	11	129722378			0			0				0		0		0	0		0	0	0		0	No-ASE
NFRKB	11	129734633									0													No-ASE
PRDM10	11	129775534			0		0	0			0				0			0	0	0	0			No-ASE
ST14	11	130058057																	0					No-ASE
ADAMTS8	11	130275533				0					0							0	0				0	No-ASE
SNX19	11	130750592		0		0		0			0			0	0			0	0	1	0		0	No-ASE
SPATA19	11	133714148					0																	No-ASE
IGSF9B	11	133788992		0			0	0	0	0	0							0	0				0	No-ASE
NCAPD3	11	134022867		0						0							0							No-ASE
GLB1L2	11	134201992						0		0	0				0	0	0			0				No-ASE
IQSEC3	12	234929					1										1							ASE
SLC6A12	12	299715		0																			1	Heterogeneous_ASE
KDM5A	12	401960		0		0	0	0					0		0									No-ASE
CCDC77	12	520944	0	0			0					0			0		0		0	0			0	No-ASE
B4GALNT3	12	644337	0	0		0	0	0				0			0				0	0		0	0	No-ASE
WNK1	12	863286		0			0	0	0				0	0				0	0	0		0	0	No-ASE
ERC1	12	1137117							0						0				0				0	No-ASE
FBXL14	12	1675943							0															No-ASE
ADIPOR2	12	1863625																	0					No-ASE
CACNA2D4	12	1902879							1															ASE
DCP1B	12	2055401					0								0				0	0				No-ASE
CACNA1C	12	2224411						0											0	0				No-ASE
LOC100507424	12	2967949							0					0					0				0	No-ASE

FOXM1	12	2970470				0																		No-ASE
TULP3	12	3018708					0	0				0							0	0		0		No-ASE
TEAD4	12	3104098																					0	No-ASE
EFCAB4B	12	3724583		0		0	1	0	0				0		0			0	0	0		0	0	No-ASE
CCND2	12	4388084	0	0			0				0			0	0			0		0		0	0	No-ASE
DYRK4	12	4700380																	1					ASE
AKAP3	12	4724934				1	1						0							0				Heterogeneous_ASE
GALNT8	12	4829956	0	0		0	0	0				0	0	0					0	0				No-ASE
KCNA1	12	5020680					0																	No-ASE
NTF3	12	5603540							1			1		1										ASE
ANO2	12	5672475				0												1						Heterogeneous_ASE
VWF	12	6058245					0	0							0			1		0		0	1	Heterogeneous_ASE
PLEKHG6	12	6421395		0		0		0						0									0	No-ASE
SCNN1A	12	6457062		0		0	0						0						0					No-ASE
CD27-AS1	12	6554628	0			0	1					0	0		0				0			0	0	No-ASE
TAPBPL	12	6562260	0			0		0	0				0	0	0			1		0		0	0	No-ASE
MRPL51	12	6601475																				0		No-ASE
NCAPD2	12	6604269	0			0		0	0				0	0	0					0		0	0	No-ASE
CHD4	12	6688050				0						0		0	0				0	0		0	1	No-ASE
LAG3	12	6882372	0	0		0																	1	No-ASE
LEPREL2	12	6939030		0		0		0					0	0				0	0	0		1	0	No-ASE
GNB3	12	6952177	0	0																				No-ASE
LRRC23	12	7015075		0																		0		No-ASE
LPCAT3	12	7086343																	0	0				No-ASE
C1S	12	7169861										0										0		No-ASE
C1R	12	7187854		0										0						0		0		No-ASE
RBP5	12	7277251																	0					No-ASE
CLSTN3	12	7283255										0												No-ASE
ACSM4	12	7456988																					1	ASE
CD163L1	12	7507726		1					1			1	0	0	0					0			0	Heterogeneous_ASE
C3AR1	12	8211373												0						0				No-ASE
CLEC4A	12	8276536		0					0			1		0	0				1				0	Heterogeneous_ASE
RIMKLB	12	8902470																					0	No-ASE
A2ML1	12	8975801	0			0								0									0	No-ASE
A2M	12	9222373															1							ASE
PZP	12	9301572										1						0						Heterogeneous_ASE
KLRB1	12	9750669										1												ASE
CLEC2D	12	9822387				1			0			0		0	1			0						Heterogeneous_ASE
KLRK1	12	10532326																		1			1	ASE
TAS2R10	12	10978112										0												No-ASE
PRR4	12	10999708	0												0				0	0			0	No-ASE
PRH1-PRR4	12	11060991			0			0	0		0			0	0			1	0	0			0	No-ASE
TAS2R42	12	11338603													0			0		0				No-ASE
LRP6	12	12277521				0		0	0			0		0	0				0				0	No-ASE
MANSC1	12	12483019	0									0		0	0			0	0				0	No-ASE
LOH12CR1	12	12514203												0										No-ASE
DUSP16	12	12629972							0			0												No-ASE
GPR19	12	12814190						1	0										0				0	No-ASE
DDX47	12	12966314		0		0			0					0	0								0	No-ASE
GSG1	12	13238121				0																		No-ASE
EMP1	12	13364476										0			0									No-ASE
GRIN2B	12	13715799				0								0	0							1	0	No-ASE
ATF7IP	12	14577193	0			0		0												0				No-ASE
PLBD1	12	14656732	0			0		0	0			0		0	0				0			1		No-ASE
GUCY2C	12	14766058												0										No-ASE
C12orf60	12	14958960							0						0									No-ASE
ART4	12	14982352																				0		No-ASE
MGP	12	15035081											0											No-ASE
PTPRO	12	15637092																	0					No-ASE
EPS8	12	15774350													0					0				No-ASE
PDE3A	12	20522252				1													1				0	Heterogeneous_ASE
SLCO1C1	12	20858967											1											ASE
SLCO1B3	12	20963638		1				0	0	1		1		1					1					Heterogeneous_ASE
SLCO1B7	12	21172285				0		0	0			1	0							0		0		No-ASE
SLCO1B1	12	21325669												1										ASE
SLCO1A2	12	21422492										0		0								1		Heterogeneous_ASE
RECQL	12	21624504																	0					No-ASE
GYS2	12	21689928										0												No-ASE
LDHB	12	21788602																	0					No-ASE
LRMP	12	25232193											0				0			0				No-ASE
CASC1	12	25261702										0		0								0		No-ASE
SSPN	12	26377285	0																					No-ASE
ITPR2	12	26493117		0					0										0					No-ASE
TM7SF3	12	27126968		0					0													0		No-ASE
C12orf71	12	27234184							0			0		0								0	1	No-ASE
ARNTL2	12	27521258		0																		0		No-ASE
C12orf70	12	27623691		0									0		0							0		No-ASE
MRPS35	12	27863792	0										0				0						0	No-ASE
MANSC4	12	27915699	0	0				0				0	0		0		0					0	0	No-ASE
CCDC91	12	28412372		0		0		0	0				0	0	0				0	0		0		No-ASE
LOC101055625	12	29592280	0														0		0					No-ASE
TMTC1	12	29659798						0																No-ASE
CAPRIN2	12	30863255		0		0		0					0		0		0			0		0		No-ASE
DENND5B	12	31540619	0			0						0		0								0	0	No-ASE
METTL20	12	31815095	0									0					0		0	0				No-ASE
KIAA1551	12	32133908		0		0		0	0			0		0	0		0		0	0		0	0	No-ASE
FGD4	12	32735037												0										No-ASE
DNM1L	12	32860302											0	0										No-ASE
YARS2	12	32903808											0	0										No-ASE
PKP2	12	32945612		0				0	0					0			0						0	No-ASE
KIF21A	12	39695319																		0				No-ASE
SLC2A13	12	40223917	0																					No-ASE
GXYLT1	12	42481607							0					0					0					No-ASE
PUS7L	12	44124184									0		0	0			0			0			0	No-ASE
IRAK4	12	44161927									0									0				No-ASE
TWF1	12	44189545									0													No-ASE
TMEM117	12	44238723												0										No-ASE
DBX2	12	45410134										0										0		No-ASE
ANO6	12	45610152																	0					No-ASE
ARID2	12	46205208												0					0					No-ASE
SCAF11	12	46316805	0			0		0	0								0		0	0		0		No-ASE
SLC38A4	12	47168900		0								1												Heterogeneous_ASE
RAPGEF3	12	48131387		0								0					0			0			1	No-ASE
HDAC7	12	48177628																	0					No-ASE
VDR	12	48238704		0		0		0	0		0	0							0	0		0		No-ASE
TMEM106C	12	48358023															0							No-ASE
COL2A1	12	48367327	0								0	1	0									0		No-ASE
PFKM	12	48501161		0		0						0	0	0	0		0		0			0		No-ASE
OR10AD1	12	48596241		1		1						1			0		1							ASE
H1FNT	12	48723108	0			0					1				0					0			1	Heterogeneous_ASE
ZNF641	12	48736791	0						0			0	0						0	0			0	No-ASE
ANP32D	12	48866481		1													1							ASE
C12orf54	12	48879982																				0		No-ASE
OR8S1	12	48919428	0									0					1					0		No-ASE
KANSL2	12	49047912						0			0	0	0	0	0				0	0		0	0	No-ASE
CCDC65	12	49298799		0		0		0				0			0		0			0		0	1	No-ASE
DDN	12	49390677	0						0		0		0		0		0					0	0	No-ASE
PRKAG1	12	49396691													0									No-ASE

MLL2	12	49418717				0						0							0	0		0		No-ASE
SPATS2	12	49878411		0																				No-ASE
FAM186B	12	49981468				0			0															No-ASE
NCKAP5L	12	50186598							0			0												No-ASE
FAIM2	12	50263148				0							0				0							No-ASE
CERS5	12	50524345				0						0							0	0		0	0	No-ASE
FAM186A	12	50724422	0									0		0						0		0	0	No-ASE
LARP4	12	50821551									0													No-ASE
ATF1	12	51189699	0			0						0		0	0		0		0			0	0	No-ASE
TMPRSS12	12	51236802												0										No-ASE
SLC11A2	12	51382124				0					0													No-ASE
LETMD1	12	51442209	0						0				0	0					0			0	0	No-ASE
BIN2	12	51681903	0																					No-ASE
CELA1	12	51722363										0												No-ASE
GALNT6	12	51745834	0						0		0									0				No-ASE
SLC4A8	12	51856227				0							1	0	0					0		0	0	No-ASE
ACVRL1	12	52306898				0																		No-ASE
KRT7	12	52629029	0					0									0							No-ASE
KRT83	12	52708420	0					0	0			0		0	0									No-ASE
KRT84	12	52771933		1							0											1	1	ASE
KRT75	12	52818377	0									0		0	0								1	No-ASE
KRT5	12	52908794									0	1								0				Heterogeneous_ASE
KRT71	12	52938320	0					1				1		0	1					0				Heterogeneous_ASE
KRT72	12	52979803						0			1	1					1			0				Heterogeneous_ASE
KRT73	12	53002080				1																	1	ASE
KRT2	12	53039003				0		0					1	0						0				No-ASE
KRT1	12	53069000	0																				0	No-ASE
KRT77	12	53086340	0																	0			0	No-ASE
KRT76	12	53162528				1		1															1	ASE
KRT3	12	53185104	0						1											0				Heterogeneous_ASE
KRT4	12	53200921							0		0								1	0				No-ASE
KRT78	12	53232935	0					0			1				0					0				No-ASE
EIF4B	12	53412610									0	0							0					No-ASE
SOAT2	12	53497402		0																				No-ASE
CSAD	12	53552336																	0					No-ASE
ZNF740	12	53578736									0													No-ASE
RARG	12	53605545						0	0		0			0	0		0		0	0		0		No-ASE
ESPL1	12	53662624	0			0			0		0			0					0				0	No-ASE
SP7	12	53722232						0																No-ASE
CALCOCO1	12	54105889		0				0											0				0	No-ASE
HOTAIR	12	54367413						0				0												No-ASE
HOXC6	12	54422504						0																No-ASE
HOXC4	12	54448022				0		0																No-ASE
OR9K2	12	55523587																0						No-ASE
OR6C74	12	55641075																0						No-ASE
OR10P1	12	56030812																0						No-ASE
METTL7B	12	56075594							0									0						No-ASE
ITGA7	12	56078933												0					0	0		0		No-ASE
MMP19	12	56230846											0											No-ASE
DGKA	12	56331777																	0					No-ASE
PMEL	12	56349118																	0					No-ASE
SUOX	12	56396395						0																No-ASE
ESYT1	12	56522122				0																		No-ASE
RNF41	12	56600432																				0		No-ASE
COQ10A	12	56660905	0																					No-ASE
PAN2	12	56713694																	0					No-ASE
STAT2	12	56737251												0					0					No-ASE
TIMELESS	12	56811549	0						0		0							1	0	0		1		Heterogeneous_ASE
GLS2	12	56865338						0	0								0			0			0	No-ASE
BAZ2A	12	56992559									0													No-ASE
NACA	12	57107359		0				0	0		0		0				0		0			0	0	No-ASE
PRIM1	12	57132236				0					0	0			0		0			0				No-ASE
GPR182	12	57389151						0					0						0				0	No-ASE
ZBTB39	12	57396637											0											No-ASE
MYO1A	12	57422576		0				0					0	0								0	0	No-ASE
TMEM194A	12	57453694																					0	No-ASE
LRP1	12	57532348						0			0	0			0		0	1		0				No-ASE
R3HDM2	12	57648644										0												No-ASE
GLI1	12	57857485		0		0												0						No-ASE
ARHGAP9	12	57866317																1					0	Heterogeneous_ASE
MARS	12	57883087				0																		No-ASE
PIP4K2C	12	57985185																	0					No-ASE
ARHGEF25	12	58004386	0									0	1				0							No-ASE
AGAP2	12	58120775													0									No-ASE
METTL1	12	58162739		0					0		0						0					0		No-ASE
AVIL	12	58197143																	0					No-ASE
XRCC6BP1	12	58335626	0	0		0			0		0	0		0	0					0		0		No-ASE
SLC16A7	12	60098664													0		1							Heterogeneous_ASE
FAM19A2	12	62104191																				0		No-ASE
MON2	12	62861043	0						0					0	0					0		0		No-ASE
C12orf61	12	62996793																	0					No-ASE
DPY19L2	12	63954300										0												No-ASE
TMEM5	12	64173757		0					0								0					0		No-ASE
SRGAP1	12	64377913																		0				No-ASE
C12orf66	12	64587633																	0					No-ASE
C12orf56	12	64660970												1				0						Heterogeneous_ASE
TBC1D30	12	65224220									0				0				0					No-ASE
LEMD3	12	65563471																		0				No-ASE
HMGA2	12	66308896	0						0		0	0	0	0								0		No-ASE
TMBIM4	12	66531745		0				0	0			0	0							0			0	No-ASE
HELB	12	66696547				0		0	0		0	0			0		0	0	0			0	0	No-ASE
GRIP1	12	66742817				1													0					Heterogeneous_ASE
DYRK2	12	68050928																		0				No-ASE
MDM1	12	68689075		0				0	0		0	0	0		0		0		0	0		0		No-ASE
NUP107	12	69082824																					0	No-ASE
LYZ	12	69742198	0														0							No-ASE
BEST3	12	70048824												0										No-ASE
PTPRB	12	70925853	0	0		0		0			0	0		0			0		0	0			0	No-ASE
PTPRR	12	71050533				0					0			0	0				0	0				No-ASE
TSPAN8	12	71519116	0											0	0		0			0			0	No-ASE
LGR5	12	71833933	0																					No-ASE
TBC1D15	12	72274318	0			0		0						0								0		No-ASE
CAPS2	12	75676045	0	0																		0		No-ASE
KRR1	12	75895733	0	0					0		0	0					0	1				0	0	No-ASE
BBS10	12	76740029													0					1				Heterogeneous_ASE
ZDHHC17	12	77199150		0				0	0		0													No-ASE
E2F7	12	77417795	0			0			0		0				0				0			0		No-ASE
NAV3	12	78225245		0										0	0					0				No-ASE
OTOGL	12	80605733		0					1						1									Heterogeneous_ASE
CCDC59	12	82747063													0									No-ASE
METTL25	12	82780723												0										No-ASE
TMTC2	12	83250899																	0					No-ASE
RASSF9	12	86198535										1							0				1	Heterogeneous_ASE
C12orf50	12	88376891																				1		ASE
C12orf29	12	88439500	0	0		0					0			0	0			1		0		0	0	No-ASE
CEP290	12	88444154																	0	0				No-ASE
KITLG	12	88890671	0									0			0			1						No-ASE
DUSP6	12	89743140	0	0		0			0		0	0		0				1		0			0	No-ASE

POC1B	12	89814934	0			0		0					0	0				0	0	0				No-ASE
CCER1	12	91347378	0																					No-ASE
DCN	12	91539870																					1	ASE
PLEKHG7	12	93134648									0		0	0				1				0	0	No-ASE
EEA1	12	93169795	0	0									0	0				1				0		No-ASE
CRADD	12	94072653	0								0		0	0	0							0		No-ASE
PLXNC1	12	94543526																					0	No-ASE
CCDC41	12	94702659												0										No-ASE
FGD6	12	95478283		0				0															0	No-ASE
VEZT	12	95645827									0	0	0	0	0			1		0		0	0	No-ASE
NTN4	12	96077310									0											0		No-ASE
CCDC38	12	96260886		0								1												Heterogeneous_ASE
HAL	12	96368014							0		0		0	0	0									No-ASE
CDK17	12	96674555						0						0	0				0					No-ASE
TMPO	12	98921742				0													0					No-ASE
IKBIP	12	99007411	0	0		0		0			0			0						0			0	No-ASE
APAF1	12	99042208	0										0											No-ASE
ANKS1B	12	99638164										0												No-ASE
UHRF1BP1L	12	100431425										0							0	0				No-ASE
DEPDC4	12	100656056																	0	0				No-ASE
SCYL2	12	100706346						0				0	0											No-ASE
GAS2L3	12	100994160													0									No-ASE
SLC5A8	12	101551065				1																		ASE
UTP20	12	101674913	0	0							0			0	0				0					No-ASE
MYBPC1	12	102008303						0																No-ASE
CHPT1	12	102091758		0								0		0										No-ASE
GNPTAB	12	102141003																	0					No-ASE
C12orf42	12	103695960													1					0				Heterogeneous_ASE
STAB2	12	103981288	0	0							0		0		0				0					No-ASE
NT5DC3	12	104171654																	0					No-ASE
HSP90B1	12	104324309		0		0																		No-ASE
C12orf73	12	104345326							0															No-ASE
TDG	12	104370728						0	0									1						Heterogeneous_ASE
TXNRD1	12	104609594										0												No-ASE
C12orf45	12	105380152		0				0	0				0	0						0			0	No-ASE
KIAA1033	12	105501598										0			0				0					No-ASE
APPL2	12	105568139						0	0		0	0	0										0	No-ASE
NUAK1	12	106460681				0							0	0	0					0				No-ASE
TCP11L2	12	106704948							0		0							1	0				0	No-ASE
POLR3B	12	106751671	0	0				0																No-ASE
BTBD11	12	107712865				0													0	0			0	No-ASE
PWP1	12	108082253						0																No-ASE
WSCD2	12	108589662				0																		No-ASE
CMKLR1	12	108685629												0										No-ASE
FICD	12	108910756							0			0												No-ASE
SART3	12	108918108	0												0		0		0					No-ASE
ISCU	12	108962663				0											0							No-ASE
SELPLG	12	109016906	0																					No-ASE
SSH1	12	109181849											0	0										No-ASE
ALKBH2	12	109526067									0								0					No-ASE
ACACB	12	109577338	0	0		0					0	0	0		0		0						0	No-ASE
MYO1H	12	109826634	0	0		0									0		0		0					No-ASE
UBE3B	12	109921448	0	0		0					0				0		0	1	0					No-ASE
MMAB	12	109994870		0		0									0		0		0					No-ASE
MVK	12	110012641												0			0						0	No-ASE
TCHP	12	110340895		0					0				0		0			1	0					No-ASE
GIT2	12	110368201				0					0		0	0					0					No-ASE
IFT81	12	110565193				0																		No-ASE
HVCN1	12	111088061																					0	No-ASE
CUX2	12	111652019											0											No-ASE
SH2B3	12	111856506	0			0		0					0		0				0					No-ASE
ATXN2	12	111902514												0										No-ASE
ACAD10	12	112130610											0											No-ASE
TMEM116	12	112369505	0	0							0	0												No-ASE
NAA25	12	112471090	0									0							0					No-ASE
TRAFD1	12	112572637									0		0											No-ASE
HECTD4	12	112601928	0	0							0								0				0	No-ASE
RPH3A	12	113266169	0																					No-ASE
OAS1	12	113344983	0	0				0	0		0	0	1	0	0				0				1	No-ASE
OAS3	12	113376388	0	0				0	0		0		0	0	0								0	No-ASE
OAS2	12	113416428	0			0		0	0				0		0								0	No-ASE
RASAL1	12	113537834	0			0		0											1					No-ASE
DDX54	12	113599081																					0	No-ASE
TPCN1	12	113659260																	1					ASE
SLC24A6	12	113737661																	0					No-ASE
PLBD2	12	113796430									0			0	0									No-ASE
RBM19	12	114282474	0	0					0			0	0	0	0				0					No-ASE
C12orf49	12	117157653		0	1			0	0									0						No-ASE
FBXW8	12	117383245									0	0								0				No-ASE
VSIG10	12	118504457	0			0		0				0	0	0	0				0			0		No-ASE
CCDC60	12	119772991		1									1		0			1		0				Heterogeneous_ASE
CIT	12	120135468	0	0		0		0			0								0			0		No-ASE
RAB35	12	120533965						0					0						0					No-ASE
GCN1L1	12	120568455																	0					No-ASE
PXN	12	120651676				0							0									0		No-ASE
COQ5	12	120941645				0																0		No-ASE
RNF10	12	120984266																	0					No-ASE
UNC119B	12	121151093																	0					No-ASE
ACADS	12	121164829						0				0	0	0									0	No-ASE
SPPL3	12	121206182																	0					No-ASE
HNF1A	12	121416650	0	0							0	0		0					0	0		0	0	No-ASE
P2RX7	12	121570787		0		1							0							0		0		No-ASE
P2RX4	12	121648031		0		0					0											0		No-ASE
CAMKK2	12	121678567		0		0								0								0		No-ASE
MORN3	12	122091018				0							0											No-ASE
TMEM120B	12	122186239		0		0		0					0		0				0			0		No-ASE
SETD1B	12	122247782						0																No-ASE
HPD	12	122277891				0						0			0							0		No-ASE
PSMD9	12	122326793				0						0	0	0	0		0		0			0		No-ASE
WDR66	12	122359236		0		0		0	0			1	0	0					0			0	0	No-ASE
BCL7A	12	122468687											0											No-ASE
MLXIP	12	122615411	0					0				0	0	0								0		No-ASE
LRRC43	12	122657198				0								0										No-ASE
B3GNT4	12	122689181		0				0												0				No-ASE
DIABLO	12	122692978													0									No-ASE
CLIP1	12	122763670		0		0					0		0	0						0		0		No-ASE
KNTC1	12	123014687						0			0	0			0								0	No-ASE
HCAR3	12	123200227	0																					No-ASE
CCDC62	12	123262080																	1					ASE
HIP1R	12	123333380																	0					No-ASE
ABCB9	12	123414616				0																		No-ASE
ARL6IP4	12	123464956																	0					No-ASE
MPHOSPH9	12	123641421				0																		No-ASE
C12orf65	12	123738265										0										0	0	No-ASE
SBNO1	12	123780585											0	0									0	No-ASE
RILPL2	12	123915089				0								0									0	No-ASE
DDX55	12	124090527										0							0					No-ASE
ATP6V0A2	12	124209215		0		0		0			0	0	0						0					No-ASE

DNAH10	12	124252160	0	0				1						0	0				1			0	1	Heterogeneous_ASE
CCDC92	12	124421759		0					0		0	0							0			0		No-ASE
ZNF664	12	124496864		1					1		1								1					ASE
ZNF664-FAM101A	12	124798797																					0	No-ASE
NCOR2	12	124810093		0				0				0	0									0		No-ASE
UBC	12	125396218																				0		No-ASE
DHX37	12	125432380	0	0		0		0			0	0	0	0	0		0					0	0	No-ASE
AACS	12	125550207		0					0		0						0							No-ASE
TMEM132C	12	128899673													0				1					Heterogeneous_ASE
SLC15A4	12	129278745						0							0									No-ASE
STX2	12	131276464									0				0								0	No-ASE
GPR133	12	131439063									0													No-ASE
SFSWAP	12	132199423																		0				No-ASE
MMP17	12	132322801	1			1					1	0		0	1									Heterogeneous_ASE
ULK1	12	132393686		0							0		0		0									No-ASE
EP400	12	132445421				0													0	0			0	No-ASE
DDX51	12	132624220	0			0					0	0	0	0	0		0		0					No-ASE
GALNT9	12	132681748									0			1										Heterogeneous_ASE
FBRSL1	12	133084818																				0		No-ASE
POLE	12	133201324		0		0		0					0		0		0		0	0		0		No-ASE
PXMP2	12	133272470															0					0		No-ASE
PGAM5	12	133291473				0													0					No-ASE
ANKLE2	12	133303836	0	0		0		0	0				0		0		0		0	0				No-ASE
GOLGA3	12	133349749											0	0	0	1	0			0		0	0	No-ASE
CHFR	12	133420708				1		0					1	0								1	0	Heterogeneous_ASE
ZNF605	12	133498358						1					0						0					Heterogeneous_ASE
ZNF84	12	133633717																		0				No-ASE
ZNF268	12	133764464						1				0	1	0	0		1		0	0				Heterogeneous_ASE
ZMYM5	13	20398669		0			0					0	0			0								No-ASE
ZMYM2	13	20567327										0												No-ASE
IFT88	13	21148610	0	0			0	0																No-ASE
XPO4	13	21361123		0		0															0			No-ASE
LATS2	13	21549202														0						0		No-ASE
SKA3	13	21729252		0	1		0	1						0			0	0				1		Heterogeneous_ASE
MRP63	13	21751052					0							0		0								No-ASE
MIPEPP3	13	21955582		0	0																	0		No-ASE
FGF9	13	22246093			0															0				No-ASE
SGCG	13	23777841		0																				No-ASE
SACS	13	23903622	0	0	0	0	0				0	0		0						0		0		No-ASE
TNFRSF19	13	24167505	0									0				0							1	No-ASE
MIPEP	13	24304584				0	0	0											0			0	0	No-ASE
SPATA13	13	24797177		0	0							0				0						0	0	No-ASE
C1QTNF9	13	24890157							0															No-ASE
PARP4	13	25000617	0	0	0			0			0		0			0			0	0	0	0	0	No-ASE
ATP12A	13	25255785					0																0	No-ASE
CENPJ	13	25457410	0								0	0		0						0			0	No-ASE
MTMR6	13	25823441		0		0	0						0									0	0	No-ASE
NUPL1	13	25876011	0	0								0	0	0						0				No-ASE
RNF6	13	26788151		0																				No-ASE
GTF3A	13	28001305	0	0		0	0	0														0		No-ASE
LNX2	13	28122502	0	0	0		0													0		0		No-ASE
POLR1D	13	28197314	0	0		0					0							0				0		No-ASE
CDX2	13	28537284		0																0				No-ASE
PRHOXNB	13	28562605											0								0			No-ASE
FLT3	13	28578209	0																					No-ASE
USPL1	13	31195291	0	0	0			0					0							0	0	0	0	No-ASE
TEX26-AS1	13	31480670					0																	No-ASE
B3GALTL	13	31821992	0													0				0	0			No-ASE
FRY	13	32606002	0			0					0	0				0					0	0		No-ASE
BRCA2	13	32893271	0	0			0	0				0	0							0			0	No-ASE
N4BP2L2	13	33016603		0	0			0														0		No-ASE
KL	13	33590971			1						0					0								Heterogeneous_ASE
STARD13	13	33684035		0	0	0	0	0			0	0	0			0		0		0				No-ASE
NBEA	13	35517220			0																		1	Heterogeneous_ASE
CCDC169-SOHLH2	13	36743177				1			1			0												Heterogeneous_ASE
EXOSC8	13	37580090	0																					No-ASE
SUPT20H	13	37583812	0		0	0			0			0	0	0						0		0	0	No-ASE
CSNK1A1L	13	37678394			1	1												0						Heterogeneous_ASE
FREM2	13	39261590	0									1				0				0		1	0	Heterogeneous_ASE
PROSER1	13	39585591		0									0								0			No-ASE
NHLRC3	13	39612819						0																No-ASE
COG6	13	40229891	0	0		0	0	0				0			0			0	0	0	0	0	0	No-ASE
MRPS31	13	41303643		0	0															0				No-ASE
SLC25A15	13	41373284	0																				0	No-ASE
ELF1	13	41507579	0	0	0				0			0								0				No-ASE
MTRF1	13	41791267					0									0								No-ASE
VWA8	13	42142362		0	0			0	0		0	0				0							0	No-ASE
DGKH	13	42701612	0								0					0					0		0	No-ASE
AKAP11	13	42866585				0																		No-ASE
EPSTI1	13	43462422			0			0																No-ASE
DNAJC15	13	43597772	0																		1		1	Heterogeneous_ASE
CCDC122	13	44411421		0		0																		No-ASE
LACC1	13	44455233				0																		No-ASE
TSC22D1	13	45008787	0		0																		0	No-ASE
NUFIP1	13	45517616			0																			No-ASE
COG3	13	46039206	0						0													0	0	No-ASE
SPERT	13	46287373					0																	No-ASE
ZC3H13	13	46538118	0								0	0							0		0	0	0	No-ASE
CPB2-AS1	13	46627762	0																			0		No-ASE
LCP1	13	46704998	0					0	0				0							0				No-ASE
KIAA0226L	13	46917631				0	0						0											No-ASE
LRCH1	13	47127796					0				0					0								No-ASE
ESD	13	47345630		0					0														0	No-ASE
SETDB2	13	50050620		0		0	0	0	0		0	0		0	0									No-ASE
PHF11	13	50080827				0	0	0			0				0							0		No-ASE
RCBTB1	13	50108364							0		0							0	0			0	0	No-ASE
ARL11	13	50204594		1							0											1		Heterogeneous_ASE
KPNA3	13	50279776			0																			No-ASE
FAM124A	13	51819493					0														0		0	No-ASE
SERPINE3	13	51915440		0																				No-ASE
DHRS12	13	52343391	0	0			0	0								0						0		No-ASE
CCDC70	13	52439534					0																	No-ASE
ATP7B	13	52508989	0	0		0	0		0		0	0			0				0			0	0	No-ASE
ALG11	13	52586598	0	0		0	0				1	0			0						0		0	No-ASE
UTP14C	13	52603838	0	0					0			0								0				No-ASE
NEK5	13	52639588	0	0		0	0														0			No-ASE
THSD1	13	52951802																				0		No-ASE
CKAP2	13	53030706																				0		No-ASE
PCDH8	13	53418747																				0		No-ASE
OLFM4	13	53602999													0				0					No-ASE
BORA	13	73303197												0										No-ASE
DIS3	13	73333935	0				0	0	0				0	0	0	0				0	0			No-ASE
PIBF1	13	73357636		1		0	0	0								0							0	No-ASE
TBC1D4	13	75860990	0			0	0		0					0		0		0	0	0			0	No-ASE
LMO7	13	76195833	0		0		0	0	0		0	0	0	0	0	0							0	No-ASE
CLN5	13	77566090										0											0	No-ASE
MYCBP2	13	77632432										0												No-ASE
SCEL	13	78130761						0						0										No-ASE

NDFIP2	13	80094993			0									0					0					No-ASE
GPC5	13	92101021												0										No-ASE
GPC6	13	93879719													0									No-ASE
GPR180	13	95257726			0							0												No-ASE
ABCC4	13	95673837			0		0													1				Heterogeneous_ASE
DZIP1	13	96238288					0		0															No-ASE
UGGT2	13	96485204	0		0	0		0	0			0	0	0	0				0					No-ASE
HS6ST3	13	96743177														0							1	Heterogeneous_ASE
FARP1	13	98828727	0				0					0	0		0	0				0				No-ASE
SLC15A1	13	99337039	0													0								No-ASE
DOCK9	13	99449468					0				0	0		0		0			0					No-ASE
CLYBL	13	100425097				0		0	1				0	0	0	1				0	0			No-ASE
PCCA	13	100755176	0			0																		No-ASE
GGACT	13	101184421	0						0														0	No-ASE
TMTC4	13	101257309				0			0		0					0				0	0		0	No-ASE
ITGBL1	13	102105247																	1					ASE
CCDC168	13	103382660					0																	No-ASE
KDELC1	13	103438652	0		0		0		0		0									0				No-ASE
BIVM	13	103459624	0																					No-ASE
BIVM-ERCC5	13	103498672	0		0		0		0		0		0	0		0				0				No-ASE
SLC10A2	13	103701648	0				0																	No-ASE
DAOA-AS1	13	106119446		0		1	0							1			1				1			Heterogeneous_ASE
LIG4	13	108861048					0	0					0											No-ASE
ABHD13	13	108881639																					0	No-ASE
MYO16	13	109365050													0	0							1	Heterogeneous_ASE
IRS2	13	110434418	0									0							0				0	No-ASE
COL4A1	13	110815830				0	0	0	0			1	0			0					0		0	No-ASE
CARS2	13	111293898																	0				0	No-ASE
ANKRD10	13	111532174				0															0			No-ASE
TEX29	13	111973269				0																		No-ASE
ATP11A	13	113439549			0		0	0	0		0	0	0			0		0	0	0			0	No-ASE
MCF2L	13	113669142	0		0		0					0			0					0			0	No-ASE
F7	13	113765018					0																	No-ASE
PROZ	13	113813005			0																			No-ASE
CUL4A	13	113873295			0		0	0				0	0		0	0			0					No-ASE
ADPRHL1	13	114077172					0							0		0								No-ASE
TMCO3	13	114149919			0	0									0				0		0			No-ASE
ATP4B	13	114303693																					1	ASE
TMEM255B	13	114469204				0							0											No-ASE
RASA3	13	114748792										0												No-ASE
CHAMP1	13	115089811				0	0						0											No-ASE
OR4K1	14	20404046					0																	No-ASE
OR4K15	14	20443751					0																	No-ASE
OR11G2	14	20665530										1	1	1				0						ASE
OR11H6	14	20691881					0						1		1									Heterogeneous_ASE
TTC5	14	20757803					0	0	0	0							0	0		0			0	No-ASE
CCNB1IP1	14	20779741			0		0		0	0		0	0	0	0		0	0		0				No-ASE
PARP2	14	20811843										0					0							No-ASE
TEP1	14	20836609			0		0	0	0	0	0	0	0	0	0		0	0		0			0	No-ASE
KLHL33	14	20897183					0				0	0											0	No-ASE
APEX1	14	20923826						0			0	0												No-ASE
PNP	14	20940468												0	0			0						No-ASE
OR6S1	14	21108896										1						1						ASE
RNASE4	14	21167576					0				0						0							No-ASE
EDDM3A	14	21215746					0																	No-ASE
RNASE6	14	21249869								0	0			0	0					0				No-ASE
RNASE3	14	21359987					0											1		0				Heterogeneous_ASE
TPPP2	14	21498771					0											0						No-ASE
RNASE7	14	21511212			0		0											0						No-ASE
RNASE8	14	21526079					0																	No-ASE
ARHGEF40	14	21541382			0					0				0	0								0	No-ASE
OR5AU1	14	21623118					0											0						No-ASE
LINC00641	14	21671277					0			0		0					0	1						No-ASE
RPGRIP1	14	21756209					0	0										1						Heterogeneous_ASE
SALL2	14	21990897					0			0		0					0						0	No-ASE
OR10G3	14	22038001																0						No-ASE
OR10G2	14	22102362			1			0										1						Heterogeneous_ASE
OR4E2	14	22133330					0								1									Heterogeneous_ASE
OXA1L	14	23235810								0								1						Heterogeneous_ASE
SLC7A7	14	23242828			0			1	0					0	0		0	0						No-ASE
MRPL52	14	23299135			0			0		0				0	0									No-ASE
MMP14	14	23310723			0				0			1		0	0			0					0	No-ASE
REM2	14	23353895										0		0										No-ASE
RBM23	14	23371055			0		0			0	0			0	0			0		0				No-ASE
C14orf93	14	23456518												0										No-ASE
PSMB5	14	23495387							0															No-ASE
CDH24	14	23517629																					0	No-ASE
ACIN1	14	23528421			0			0	0	0	0	0		0	0		0			0			0	No-ASE
HOMEZ	14	23744806			0		0	0	0			0					0	0						No-ASE
EFS	14	23826539					0																	No-ASE
MYH6	14	23851739													0									No-ASE
THTPA	14	23992742									0												0	No-ASE
AP1G2	14	24029037										0					0			0				No-ASE
DHRS4-AS1	14	24423002						0																No-ASE
DHRS4L2	14	24458162					0	0										1					1	Heterogeneous_ASE
DHRS4L1	14	24506975									0												1	Heterogeneous_ASE
LRRC16B	14	24522938			1		0										0	0						No-ASE
PCK2	14	24566139										0												No-ASE
DCAF11	14	24586496						0				0		0			0						0	No-ASE
EMC9	14	24608235						0																No-ASE
IPO4	14	24649911												0				0		0			0	No-ASE
TM9SF1	14	24658776																0		0			0	No-ASE
CHMP4A	14	24679139			0			0		0		0		0				1		0			1	No-ASE
NEDD8-MDP1	14	24683304									0													No-ASE
GMPR2	14	24702413																0		0				No-ASE
TGM1	14	24718568												0										No-ASE
RABGGTA	14	24736961						0							0								0	No-ASE
DHRS1	14	24760198													0									No-ASE
NOP9	14	24769180						0				0					0	0		0			0	No-ASE
ADCY4	14	24788316						0																No-ASE
RIPK3	14	24805463										0			0		0							No-ASE
NYNRIN	14	24877468			0		0	0									0	1		0			0	No-ASE
KHNYN	14	24900115						0						0	0									No-ASE
CMA1	14	24974790										0												No-ASE
CTSG	14	25042955																0						No-ASE
GZMB	14	25100282												1				0						Heterogeneous_ASE
MIR548AI	14	30046460									0													No-ASE
SCFD1	14	31099759																		1			0	Heterogeneous_ASE
COCH	14	31346813												0								0	0	No-ASE
STRN3	14	31364948			0					0		0											0	No-ASE
HECTD1	14	31572251						0														0		No-ASE
HEATR5A	14	31762552			0				0		0			0				1		0				No-ASE
AKAP6	14	32902715			0			0		0	0	0			0		0			0		0	0	No-ASE
NPAS3	14	34243570								0														No-ASE
EAPP	14	34985658										0												No-ASE
BAZ1A	14	35222760			0				0		0												0	No-ASE
KIAA0391	14	35592756			0									0	0								0	No-ASE
RALGAPA1	14	36041924												0										No-ASE

MBIP	14	36777304										0												No-ASE
FOXA1	14	38060646			0			0		0	0			0			0	0						No-ASE
SEC23A	14	39509974		1																			0	Heterogeneous_ASE
PNN	14	39645745																					0	No-ASE
MIA2	14	39709768		1													0					0		Heterogeneous_ASE
CTAGE5	14	39736680									0												0	No-ASE
FSCB	14	44973719															1							ASE
FAM179B	14	45431637																					0	No-ASE
PRPF39	14	45564466																0						No-ASE
FANCM	14	45605287						0	0		0	0					0	0				0		No-ASE
MIS18BP1	14	45673341			0															0		0		No-ASE
RPS29	14	50044392								0				0									0	No-ASE
LRR1	14	50069088			0				0			0	0									0	0	No-ASE
DNAAF2	14	50092393			0				0	0	0	0	0	0	0			0				0	0	No-ASE
KLHDC1	14	50175886												0										No-ASE
C14orf182	14	50472338						0									0						1	Heterogeneous_ASE
METTL21D	14	50581011												0								0	0	No-ASE
L2HGDH	14	50732095								0														No-ASE
ATP5S	14	50779742			0				0		0	0	0		0		0			0		0		No-ASE
CDKL1	14	50796881			0				0		0	0	0		0		0			0		0		No-ASE
MAP4K5	14	50895394							0				0							0				No-ASE
NIN	14	51188341			0				0		0	0	0	0				0		0		0		No-ASE
ABHD12B	14	51345537										0		0									0	No-ASE
PYGL	14	51372120						1																ASE
TRIM9	14	51446216			0							0	1				1	0				1	0	Heterogeneous_ASE
NID2	14	52473280						1					1	0									0	Heterogeneous_ASE
TXNDC16	14	52899036			0						0	0		0			0	0				0		No-ASE
GPR137C	14	53020155									0	0	0	0	0			0				0	0	No-ASE
BMP4	14	54417117			0			0	0	0					0		0	0					1	No-ASE
WDHD1	14	55408252						0																No-ASE
LGALS3	14	55604109						0			0	0	0		0		0	0					0	No-ASE
DLGAP5	14	55618479						0				0												No-ASE
FBXO34	14	55817352						0				0	0		0		0	0					0	No-ASE
C14orf101	14	57046768			0			0			0									0				No-ASE
EXOC5	14	57672715						0										0				0		No-ASE
AP5M1	14	57736048										0								0				No-ASE
C14orf37	14	58471800							0					1				0						Heterogeneous_ASE
ARID4A	14	58768299		1	0						0	0		0	0		0	0		0		0	0	No-ASE
TOMM20L	14	58863051															0						0	No-ASE
DACT1	14	59105180							0								0						0	No-ASE
L3HYPDH	14	59939704			0										0		0					0	0	No-ASE
CCDC175	14	59988324		1	0							1			0							1	0	Heterogeneous_ASE
PCNXL4	14	60581809																		0			0	No-ASE
SIX4	14	61180193		1										0										Heterogeneous_ASE
TMEM30B	14	61745417						0	0		0						0					0		No-ASE
PRKCH	14	61788973									0													No-ASE
HIF1A	14	62187212						0				0		0			0							No-ASE
SNAPC1	14	62229285																		0				No-ASE
SYNE2	14	64375888			0			0			0	0			0		0	0				0	0	No-ASE
MTHFD1	14	64867522		1	0			0						0	0			0		0		0	0	No-ASE
AKAP5	14	64935148																		0				No-ASE
ZBTB1	14	64988814			0				0											0		0	0	No-ASE
PPP1R36	14	65016720			0							0		0						0		0		No-ASE
PLEKHG3	14	65194379						0			0													No-ASE
SPTB	14	65216718			0	0		0	0					0			0	0		0			0	No-ASE
CHURC1-FNTB	14	65406238			0										0			0		0				No-ASE
FUT8	14	66082779						0														0		No-ASE
ATP6V1D	14	67807223			0																			No-ASE
PLEK2	14	67854986																					0	No-ASE
PLEKHH1	14	68022572			0			0	0		0	0		0	0					0		0	0	No-ASE
RDH11	14	68156965													0			0						No-ASE
RDH12	14	68191206							0									1					1	Heterogeneous_ASE
ZFYVE26	14	68213744			0			0			0	0			0		0	0		0		0	0	No-ASE
ACTN1	14	69341650										0												No-ASE
SLC8A3	14	70512889																0						No-ASE
ADAM20	14	70989325									0												1	Heterogeneous_ASE
SIPA1L1	14	72054629						0	0									0				0		No-ASE
DPF3	14	73137882									0	0			0					0				No-ASE
DCAF4	14	73404680			0							0		0			0							No-ASE
ZFYVE1	14	73437732						0			0				0		0							No-ASE
PAPLN	14	73711394						0			0	0		0	0			0		0			0	No-ASE
NUMB	14	73743458															0							No-ASE
HEATR4	14	73945346			0			0			0	0		0	0			0		0		0		No-ASE
ACOT4	14	74060508										0						0						No-ASE
ELMSAN1	14	74188007							0			0		0			0							No-ASE
PTGR2	14	74318645													0		1							Heterogeneous_ASE
ZNF410	14	74360573			0			0						0	0		0					0		No-ASE
FAM161B	14	74401030						0				0		0	0		0					0		No-ASE
COQ6	14	74417180						0						0	0		0					0		No-ASE
CCDC176	14	74489695		0	0			0						0	0		0	1		0		0		No-ASE
ALDH6A1	14	74533575																0						No-ASE
ABCD4	14	74753420			0			0	0		0	0		0	0		0			0				No-ASE
YLPM1	14	75230643			0			0				0												No-ASE
RPS6KL1	14	75375560			0				0								0	0		0		0		No-ASE
EIF2B2	14	75470349										0												No-ASE
MLH3	14	75483796						0	0					0			0	0		0				No-ASE
ZC2HC1C	14	75537322			0				0					0			0	0		0		0		No-ASE
NEK9	14	75551321			0				0					0			0	0		0				No-ASE
JDP2	14	75904615			0			0			0	0			0		0	0				0		No-ASE
FLVCR2	14	76045362										0		0				1						Heterogeneous_ASE
TTLL5	14	76129532			0				0		0	0			0					0				No-ASE
IFT43	14	76452145			0							0		0	0		0			0		0		No-ASE
ANGEL1	14	77255665			0						0	0						0		0		0		No-ASE
C14orf166B	14	77294654									0													No-ASE
IRF2BPL	14	77491837							0															No-ASE
POMT2	14	77744798																0						No-ASE
GSTZ1	14	77787706			0			0	0					0			0			0			0	No-ASE
SAMD15	14	77843814			0			0	0			0		0	0		0	0		0				No-ASE
ISM2	14	77942225										0		1				0		0				No-ASE
SPTLC2	14	77978621																		0				No-ASE
ALKBH1	14	78140190			0				0			0			0			0					0	No-ASE
DIO2	14	80669091																0						No-ASE
CEP128	14	80963829			0			0	0		0	0		0	0		0							No-ASE
TSHR	14	81422124										0		0										No-ASE
STON2	14	81736917							0					0				0				0	0	No-ASE
SEL1L	14	81945991																0						No-ASE
FLRT2	14	86088177																0						No-ASE
GALC	14	88401213			1							1						0				1	1	ASE
GPR65	14	88477577																0						No-ASE
KCNK10	14	88651913						0				0		0										No-ASE
SPATA7	14	88862529							0		0	0		0			0			0				No-ASE
PTPN21	14	88934443							0		0	0		0			0			0				No-ASE
EML5	14	89084617			1				0		1	0		0			0							Heterogeneous_ASE
NRDE2	14	90744776			0												0	0					0	No-ASE
TTC7B	14	91007828												0										No-ASE
C14orf159	14	91626666									0	0	0	0	0			0		0		0	0	No-ASE
CCDC88C	14	91739075			0			0							0					0			0	No-ASE
SMEK1	14	91925103						0									0						0	No-ASE

TRIP11	14	92436057							0				0		0		0						0	No-ASE
ATXN3	14	92537364							0				0		0		0						0	No-ASE
SLC24A4	14	92792313									0													No-ASE
RIN3	14	93022148							0			0	0	0				0		0			0	No-ASE
LGMN	14	93170994											0											No-ASE
GOLGA5	14	93263964											0		0		0			0		0	0	No-ASE
CHGA	14	93392980							0			0	0									0		No-ASE
MOAP1	14	93649713															0							No-ASE
UBR7	14	93673655									0												0	No-ASE
BTBD7	14	93708759															0							No-ASE
UNC79	14	93813621																0				0		No-ASE
ASB2	14	94401051																					1	ASE
IFI27L1	14	94563193									0	0		0			1	0				1	1	Heterogeneous_ASE
SERPINA10	14	94750336										1					0	0						Heterogeneous_ASE
SERPINA6	14	94770808							0									1						Heterogeneous_ASE
SERPINA1	14	94844843			0				0					0	0			0		0		0	1	No-ASE
SERPINA11	14	94909130																1						ASE
SERPINA5	14	95053772											0											No-ASE
SERPINA3	14	95080786						0	0		1	0	0									0	1	Heterogeneous_ASE
DICER1	14	95556961																					0	No-ASE
CLMN	14	95657901																		0				No-ASE
SYNE3	14	95884218			0			0	0			0	0	0	0					0			0	No-ASE
GLRX5	14	96010424							0													0		No-ASE
TCL1B	14	96152821															1							ASE
BDKRB2	14	96703484			0							0			0			0				0		No-ASE
ATG2B	14	96752219			0			0				0					0	0				0		No-ASE
AK7	14	96864529							0			0	0	0			0					1	0	No-ASE
VRK1	14	97304113						0	0			0	0					0					0	No-ASE
EML1	14	100331926			1						1	0			0							0	0	Heterogeneous_ASE
DEGS2	14	100615546						0									0							No-ASE
SLC25A47	14	100792126			0				0				0				0	0					0	No-ASE
WDR25	14	100847281			0				0						0			0		0				No-ASE
RTL1	14	101347090																0				0		No-ASE
PPP2R5C	14	102323220			0									0	0								0	No-ASE
DYNC1H1	14	102445720						0		0										0				No-ASE
HSP90AA1	14	102548006			0								0	0	0									No-ASE
MOK	14	102695693			0			0		0	0		0	0	0					0				No-ASE
ZNF839	14	102792323			0			0	0		0	0	0		0		0	0		0		0	0	No-ASE
CINP	14	102814997										0			0		0			0				No-ASE
TECPR2	14	102843111			0				0		0				0		0							No-ASE
TRAF3	14	103336572							0		0		0				0						0	No-ASE
EXOC3L4	14	103566644											0		0			0						No-ASE
LINC00605	14	103654939											1		1									ASE
KLC1	14	104120903			0				0			0	0	0						0			0	No-ASE
ZFYVE21	14	104193136													0		0							No-ASE
C14orf2	14	104381411						0					0									0	0	No-ASE
ASPG	14	104552159			0				0									0		0		0		No-ASE
KIF26A	14	104618593			1			0																Heterogeneous_ASE
INF2	14	105167739						0					0							0			0	No-ASE
SIVA1	14	105221996						0														0		No-ASE
ZBTB42	14	105267925						0												0				No-ASE
AHNAK2	14	105404534			0			0	0			0	0		0		0	0		0			0	No-ASE
C14orf79	14	105452770						0																No-ASE
GPR132	14	105517420			0																			No-ASE
JAG2	14	105609043						0					0		0					0				No-ASE
NUDT14	14	105639582			0																	0		No-ASE
PACS2	14	105814891																				0		No-ASE
MTA1	14	105915724									0							0				0	0	No-ASE
CRIP1	14	105954687						0			0	0	0		0		0	0		0				No-ASE
TUBGCP5	15	22833543			1																			ASE
CYFIP1	15	22933860			0					0		0	0										0	No-ASE
NIPA1	15	23043896	0		0				0	0		0								0	0	0	0	No-ASE
MAGEL2	15	23889200					1																	ASE
ATP10A	15	25924498								0														No-ASE
OCA2	15	28000551						0																No-ASE
HERC2	15	28356991																		1				ASE
TJP1	15	30000773						0		0		0									0			No-ASE
FAN1	15	31196867			0			0		0			0	0						0				No-ASE
OTUD7A	15	31776558								0														No-ASE
FMN1	15	33091015			0	0								0	0					0	0		0	No-ASE
AVEN	15	34158888				0																		No-ASE
PGBD4	15	34394926										0												No-ASE
C15orf55	15	34640188				0				0											0			No-ASE
C15orf41	15	36936489								0		0	0	0						0			0	No-ASE
THBS1	15	39874396						0					0	0										No-ASE
FSIP1	15	39909953											0	0	0									No-ASE
EIF2AK4	15	40231779			0	0		0					0	0						0			0	No-ASE
BUB1B	15	40457337	0							0				0							0			No-ASE
PAK6	15	40544493								0				0	0						0		0	No-ASE
C15orf52	15	40627384						0																No-ASE
DISP2	15	40655827	0					0		0		0		0							0		0	No-ASE
KNSTRN	15	40675061						0				0												No-ASE
BAHD1	15	40750677						0				0		0						0	0		0	No-ASE
C15orf57	15	40845952	0			0		0				0		0						0				No-ASE
CASC5	15	40898604	0	0				0				0		0			0			0				No-ASE
DNAJC17	15	41060221											0							0	0			No-ASE
ZFYVE19	15	41099819			0			0		0										0	0			No-ASE
DLL4	15	41222183																			0			No-ASE
INO80	15	41272426												0										No-ASE
NDUFAF1	15	41679668	0		0					0		0	0	0						0				No-ASE
LTK	15	41796352						0																No-ASE
RPAP1	15	41810019			0			0					0	0						0	0		0	No-ASE
TYRO3	15	41853402		1					1													1		ASE
MGA	15	41961113	0			0		1				0	1	0	0					0				No-ASE
MAPKBP1	15	42103137	0										0	0						0			0	No-ASE
JMJD7-PLA2G4B	15	42120372						0						0										No-ASE
SPTBN5	15	42142100	0	0				0					0	0	0						0		1	No-ASE
EHD4	15	42192923	0			0		0				0	0							0	0			No-ASE
PLA2G4E	15	42278162											0											No-ASE
PLA2G4D	15	42360950	0		0					0		0	0	0						0	0			No-ASE
PLA2G4F	15	42434202			0	0						0	0		0									No-ASE
GANC	15	42568547			0	0		0		0			0	0							0			No-ASE
CAPN3	15	42652013						0						0										No-ASE
ZFP106	15	42710122						0					0	0										No-ASE
STARD9	15	42877759											0	0										No-ASE
CDAN1	15	43017376											0	0						0				No-ASE
TTBK2	15	43038006						0						0						0				No-ASE
UBR1	15	43237575						0		0				0										No-ASE
LCMT2	15	43620707				0							0											No-ASE
ADAL	15	43627351	0					0																No-ASE
ZSCAN29	15	43653305	0			0		0					0							0				No-ASE
TUBGCP4	15	43668300												0										No-ASE
RNU6-28	15	43705365	0			0		0				0	0		0					0				No-ASE
CATSPER2	15	43924420	0																					No-ASE
WDR76	15	44119235				0		0					0							0				No-ASE
CASC4	15	44581508						0																No-ASE
SPG11	15	44855395			0	0		0		0	0	0	0	0	0					0	0			No-ASE
TRIM69	15	45047125			0	0		0		0	0													No-ASE

DUOX2	15	45386358									0		0	0										No-ASE
DUOX1	15	45424212	0			0		1					0	0						0	0		0	No-ASE
SHF	15	45464510	0					0			0			0									0	No-ASE
SLC28A2	15	45545437										1	0	1									0	Heterogeneous_ASE
GATM	15	45656996	0								0	0		0										No-ASE
SQRDL	15	45951224			0			0			0		0	0										No-ASE
SEMA6D	15	48052588																			0			No-ASE
CEP152	15	49030795											0							0				No-ASE
SECISBP2L	15	49284452	0																				0	No-ASE
ATP8B4	15	50152401	0		0	0					0	0		0						0				No-ASE
SLC27A2	15	50474766		0				0		0											0			No-ASE
USP8	15	50757285	1									0		1							0			Heterogeneous_ASE
TRPM7	15	50853900		1								0								0	0			No-ASE
AP4E1	15	51201108	0		0			0					0											No-ASE
TNFAIP8L3	15	51350090									0		1	0							0			No-ASE
CYP19A1	15	51503159											1											ASE
GLDN	15	51675988	0									1	1	0										Heterogeneous_ASE
DMXL2	15	51741195	0		0			0				0	0	0							0			No-ASE
GNB5	15	52425618						0				0		0										No-ASE
MYO5C	15	52486169									0	0		0										No-ASE
MYO5A	15	52606328				0						1												Heterogeneous_ASE
FAM214A	15	52874488						0						0										No-ASE
WDR72	15	53815417	0		0									1	0					1			0	Heterogeneous_ASE
RAB27A	15	55497811																					0	No-ASE
PIGB	15	55611603				0								0						0			0	No-ASE
CCPG1	15	55651926			0								0							0				No-ASE
DYX1C1-CCPG1	15	55700981				0		0				0	0	0						0				No-ASE
PRTG	15	55912234	0																					No-ASE
NEDD4	15	56122778		1	0			0		0	0			0							0			No-ASE
MNS1	15	56756243	0			0					0	0	0										0	No-ASE
ZNF280D	15	56923700	0	1							0										0		0	No-ASE
TCF12	15	57484419			0																			No-ASE
CGNL1	15	57730466	0					0		1	0										0			No-ASE
MYZAP	15	57896482						0						0										No-ASE
ALDH1A2	15	58254226																		0				No-ASE
AQP9	15	58430844										0												No-ASE
LIPC	15	58724298	0		1	0						1			0						0			Heterogeneous_ASE
RNF111	15	59323048	0		0	0		0		0			0	0							0			No-ASE
MYO1E	15	59430501		0	0			0		0	0			0			1			0	0			No-ASE
GCNT3	15	59910440									0													No-ASE
BNIP2	15	59961125	0											0							0			No-ASE
RORA	15	60781513								0				0						0	0		0	No-ASE
VPS13C	15	62147087				0					0	0	0	0										No-ASE
TLN2	15	62939598			0						0		0							0	0			No-ASE
LACTB	15	63414083	0					0		0				0							0			No-ASE
LOC100130855	15	63889602	0							0				0									0	No-ASE
HERC1	15	63915986	0			0		0		0		0	0	0	0					0	0		0	No-ASE
SNX1	15	64388275	0			0				0			0	0						0	0		0	No-ASE
PIF1	15	65108136			0	0					0			0						0				No-ASE
PLEKHO2	15	65140807	0	0								0											1	No-ASE
ANKDD1A	15	65208022	0			0				0	0										0		0	No-ASE
CILP	15	65489128				0					0													No-ASE
IGDCC3	15	65621257										0												No-ASE
IGDCC4	15	65676366	0		0	0	0	0				0	0		1						1		0	No-ASE
SLC24A1	15	65916465									0				0									No-ASE
MEGF11	15	66191095	0							0											0			No-ASE
DIS3L	15	66601052	0							0	0	0								0	0		0	No-ASE
TIPIN	15	66629354			0			0			0													No-ASE
ZWILCH	15	66797725	0							0		0		0							0		0	No-ASE
LCTL	15	66845422	0											0										No-ASE
AAGAB	15	67495926			0	0				0		0		0						0	0		0	No-ASE
IQCH	15	67553690	0																					No-ASE
ITGA11	15	68596162	0		1	1		1		1		0		1						0	1			Heterogeneous_ASE
MIR548H4	15	69238145				0					0			0							0			No-ASE
GLCE	15	69548338	0		0	0		0			0									0				No-ASE
KIF23	15	69708397					0							0										No-ASE
NR2E3	15	72103835	0											0						1				Heterogeneous_ASE
MYO9A	15	72119057	0				1					0											0	No-ASE
GRAMD2	15	72454690				0		0																No-ASE
PKM	15	72492096						0																No-ASE
BBS4	15	72987530			0	0															0			No-ASE
ADPGK	15	73044594				0					0	0	0								0		0	No-ASE
PML	15	74315485	0		0	0		0		0	0		0	0						0	0		0	No-ASE
STRA6	15	74472455	0								0	0	0							0			1	No-ASE
CCDC33	15	74536356								0	0										1			Heterogeneous_ASE
CYP1A1	15	75012894						0			0	0												No-ASE
ULK3	15	75129584			0	0		0		0		0	0										1	No-ASE
MPI	15	75182424	0			0		0				0	0										0	No-ASE
C15orf39	15	75498447	0		0	0					0		0							0	0		0	No-ASE
NEIL1	15	75641367																		0				No-ASE
MAN2C1	15	75648541	0																				0	No-ASE
CSPG4	15	75968078	0		0							0		0							0			No-ASE
ODF3L1	15	76016583	0			0		1			0	0		0						0	0			No-ASE
C15orf27	15	76426621	0								0		0							0				No-ASE
SCAPER	15	76643618	0								0		0	0						0	0			No-ASE
PEAK1	15	77406507	0		0	0		0		0		0		0										No-ASE
HMG20A	15	77750775	0		0						0	0	0	0										No-ASE
TBC1D2B	15	78290630	0								0	0											0	No-ASE
SH2D7	15	78386540								0														No-ASE
IDH3A	15	78441758				0																		No-ASE
ACSBG1	15	78466028			0							0		0									0	No-ASE
IREB2	15	78757624	0			0		0			0									0			0	No-ASE
AGPHD1	15	78805635			0								0							0				No-ASE
CHRNA5	15	78873272	0			0					0	0	0							0				No-ASE
CTSH	15	79214498			0					0			0								0			No-ASE
RASGRF1	15	79254524										0												No-ASE
KIAA1024	15	79748576	0			0					0	0									0			No-ASE
ST20-MTHFS	15	80137560	0			0		0			0	0	0											No-ASE
IL16	15	81517772						1						0							0		0	No-ASE
TMC3	15	81624768						1		0	0										0		0	No-ASE
MEX3B	15	82336141											1	0						0				Heterogeneous_ASE
EFTUD1	15	82422800	0					0		0	0													No-ASE
FAM154B	15	82555242	0							0	0	0	0							0			1	No-ASE
FSD2	15	83428126				0						0									0			No-ASE
WHAMM	15	83481859						1		0	0	0												No-ASE
C15orf40	15	83674432	0			0				0	0	0	0	0						0	0			No-ASE
TM6SF1	15	83781599											1											ASE
SH3GL3	15	84257447										1												ASE
ADAMTSL3	15	84324527	0																					No-ASE
ZSCAN2	15	85147499	0					0							0					0				No-ASE
WDR73	15	85186712										0								0				No-ASE
NMB	15	85198599	0					0			0	0	0										0	No-ASE
ZNF592	15	85326313				0				0	0												0	No-ASE
ALPK3	15	85360252	0			0		0			0	0	0	0						0	0		0	No-ASE
SLC28A1	15	85430976	0			0						1		0						0			1	Heterogeneous_ASE
PDE8A	15	85607620	0																		0			No-ASE
AKAP13	15	86064750	0			0		0		0		0	0	0	0						0		0	No-ASE
KLHL25	15	86311276	0										0											No-ASE

AGBL1	15	86687049	0			0								0										No-ASE
AEN	15	89169484										0	0										0	No-ASE
ACAN	15	89379448	0									1												Heterogeneous_ASE
HAPLN3	15	89421212			0																			No-ASE
MFGE8	15	89442723	0		0						0	0		0	0								0	No-ASE
ABHD2	15	89659716				0								0										No-ASE
RLBP1	15	89753595				0																		No-ASE
FANCI	15	89803944				0		0		0		0	0							0	0			No-ASE
POLG	15	89861812			0								0										0	No-ASE
RHCG	15	90016059			0			0						0									1	No-ASE
TICRR	15	90118867	0			0				0	0		0	0	0					0	0			No-ASE
KIF7	15	90171613	0			0				0	0		0	0						0	0		0	No-ASE
WDR93	15	90245033	0									0									0		0	No-ASE
ANPEP	15	90328681	0			0		0		0	0			0						1	0		0	No-ASE
ZNF710	15	90610517											0											No-ASE
SEMA4B	15	90760712				0														0				No-ASE
GDPGP1	15	90784165	0		0	0				0	0		0							0	0			No-ASE
TTLL13	15	90794031									0				0						0			No-ASE
NGRN	15	90809534						0			0													No-ASE
ZNF774	15	90902097	0		0					0	0	0		0							0			No-ASE
BLM	15	91290630				0							0											No-ASE
MAN2A2	15	91448591				0								0						0	0			No-ASE
UNC45A	15	91475018	0																					No-ASE
SLCO3A1	15	92459303						0													0			No-ASE
RGMA	15	93588374			1																			ASE
MCTP2	15	94841430	0			0		0			0	0		0						0	0			No-ASE
SPATA8	15	97327391																		0				No-ASE
ARRDC4	15	98504151	0	1	0	0		0		0			0							0	0		0	No-ASE
SYNM	15	99645541	0					0		0	1		1							1	1		0	Heterogeneous_ASE
TTC23	15	99678324																			0			No-ASE
LYSMD4	15	100269341	0	0		0		0												0	0			No-ASE
ADAMTS17	15	100514614	0			0		0			0	0	0	0	0					1			0	No-ASE
LINS	15	101109598						0				0		0						0	0			No-ASE
LRRK1	15	101464847	0									0	0								0		0	No-ASE
CHSY1	15	101717599	0			0		0		0		0	0							0	0			No-ASE
PCSK6	15	101845469			0	0		0		0	0	0	1	0							0		1	No-ASE
TARSL2	15	102194813						0		0				0							0			No-ASE
RGS11	16	319511									0		0							1				Heterogeneous_ASE
PDIA2	16	333229	0	0			0	1					0	0		0		0			0		0	No-ASE
AXIN1	16	337691				0	0																	No-ASE
MRPL28	16	417691	0			0																	0	No-ASE
TMEM8A	16	422085		0	0			0						0						0	0		0	No-ASE
PIGQ	16	624108	0	0	0	0		0			0			0				0		0	0			No-ASE
C16orf13	16	684561		0																				No-ASE
WDR90	16	700622	0	0		0		0		0	0	0		0					0		0			No-ASE
RHOT2	16	718533		0		0		0			0	0		0					0		0			No-ASE
CCDC78	16	772978		0		0		0						0				0						No-ASE
MSLN	16	812700		1		0																		Heterogeneous_ASE
CHTF18	16	838713	0	0		0		0					0	0					0	0				No-ASE
PRR25	16	855591		0																				No-ASE
LMF1	16	904551														0								No-ASE
SSTR5	16	1129010	0			0	0	0		0	0			1				0						No-ASE
CACNA1H	16	1245057		0				0		0		0						0	1				0	No-ASE
TPSG1	16	1271890					0			1		0				1		0						Heterogeneous_ASE
BAIAP3	16	1384711								0				0				0		0				No-ASE
UNKL	16	1416362			0	0																		No-ASE
CCDC154	16	1484455												0				0						No-ASE
CLCN7	16	1497403														0			0	0				No-ASE
PTX4	16	1535950				0	0			1	1												1	Heterogeneous_ASE
TELO2	16	1544302				0	0			0	0					0			0	0			0	No-ASE
IFT140	16	1560953														0							0	No-ASE
CRAMP1L	16	1675986								0														No-ASE
MAPK8IP3	16	1793383	0			0	0				0	0	0							0				No-ASE
MRPS34	16	1822237								0		0				0			0				0	No-ASE
EME2	16	1823740		0																				No-ASE
IGFALS	16	1840711									0													No-ASE
HAGH	16	1859310									1													ASE
FAHD1	16	1877272				0						0	0					0		0				No-ASE
MEIOB	16	1891843										1						0						Heterogeneous_ASE
HS3ST6	16	1961647				1												0						Heterogeneous_ASE
RNF151	16	2017335												0		0								No-ASE
TBL3	16	2024204									0												0	No-ASE
NOXO1	16	2029498			0					0													0	No-ASE
GFER	16	2034463								0	0			0					0	0				No-ASE
SYNGR3	16	2042650										0												No-ASE
ZNF598	16	2048437		0	0		0	0	0	0	0		0	0		0				0			0	No-ASE
NPW	16	2070164					0	0										0						No-ASE
SLC9A3R2	16	2086353								0								0						No-ASE
PKD1	16	2139969	0	0		0				0			0			0				0			0	No-ASE
BRICD5	16	2259442			0	0		0			0		0	0				0						No-ASE
PGP	16	2263822														0							0	No-ASE
ECI1	16	2289990			0							0												No-ASE
ABCA3	16	2326689														0								No-ASE
CCNF	16	2481277		0																				No-ASE
SRRM2	16	2807922	0	0		0	0	0								0			0				0	No-ASE
TCEB2	16	2821486	0	0		0								0										No-ASE
PRSS41	16	2848610				0	0	1																Heterogeneous_ASE
PRSS22	16	2903942						0																No-ASE
PAQR4	16	3021200									0													No-ASE
HCFC1R1	16	3072916	0				0				0												0	No-ASE
IL32	16	3117378																					1	ASE
MEFV	16	3293241		0		1		0				0		0				0			0			No-ASE
ZNF263	16	3333907		0								0							0				0	No-ASE
ZNF75A	16	3367229		0								0							1				1	Heterogeneous_ASE
ZSCAN32	16	3432913			0								0	0										No-ASE
CLUAP1	16	3556398					0																	No-ASE
NLRC3	16	3593426				0					0													No-ASE
SLX4	16	3632347			0	0													0					No-ASE
DNASE1	16	3705380									0												0	No-ASE
TRAP1	16	3708130				0		0		0	0	0								0				No-ASE
ADCY9	16	4015870		0		0					0	0				0		0	1	0			0	No-ASE
PAM16	16	4390324	0							0							1							Heterogeneous_ASE
CORO7-PAM16	16	4407245	0			0	0			0	0		0										0	No-ASE
DNAJA3	16	4475905	0								0							0	0	0			0	No-ASE
NMRAL1	16	4511873						0			0			0				0						No-ASE
ANKS3	16	4747039				0						0				0								No-ASE
C16orf71	16	4786548				0				0	0					0			0					No-ASE
ROGDI	16	4847466												0										No-ASE
UBN1	16	4902950			0							0							0					No-ASE
PPL	16	4933396	0	0	0		0	0		0	0	0	0	0		0				0				No-ASE
NAGPA	16	5075526				0						0		0		0				0	0			No-ASE
C16orf89	16	5097882									1	1		1		0								ASE
METTL22	16	8719577	0	0	0	0		0		0				0		0		0			0		0	No-ASE
ABAT	16	8829621		0	0	0		0				0		0		0		0					0	No-ASE
TEKT5	16	10721476		1		0	0	0			0		0			0				0				No-ASE
NUBP1	16	10837728																					0	No-ASE
CIITA	16	10971206		0	1											0							1	Heterogeneous_ASE
TNP2	16	11362728		0		1				1		1						0						Heterogeneous_ASE

PRM3	16	11367154					0																No-ASE
LITAF	16	11647492		0		0		0		0			0	0		0		0	0					No-ASE
SNN	16	11769948	0					0					0							0	0		0	No-ASE
TXNDC11	16	11773222	0	0		0																		No-ASE
ZC3H7A	16	11846619	0	0	0			0					0											No-ASE
RSL1D1	16	11930337			0	0	0							0		0		0	0					No-ASE
SNX29	16	12136831			0						0													No-ASE
CPPED1	16	12758816														0								No-ASE
SHISA9	16	13002359																0						No-ASE
ERCC4	16	14014141						0															0	No-ASE
BFAR	16	14738258		0						0				0						0			0	No-ASE
PDXDC1	16	15098057	0	0	0	0		0	0	0		0	0			0								No-ASE
NTAN1	16	15131962	0	0	0	0		0	0	0			0			0								No-ASE
RRN3	16	15155630										0												No-ASE
C16orf45	16	15609204	0																					No-ASE
NDE1	16	15761154									0			0										No-ASE
MYH11	16	15820787						0	1		0		1	0		1				0	0		1	Heterogeneous_ASE
ABCC1	16	16101793		0		0		0		0			0					0						No-ASE
ABCC6	16	16244628		0	0	0	0	0	0	0				0		1		0		0	0		0	No-ASE
SMG1	16	18823314									0													No-ASE
TMC7	16	19020590	0	0		0	0				0		0					0	0					No-ASE
COQ7	16	19079036	0	0		0	0	0	0		0							0	0					No-ASE
SYT17	16	19194864															0							No-ASE
TMC5	16	19451418							0	0	0	0		0		0			0				0	No-ASE
CCP110	16	19539306		0	0			0	0	0	0	0	0	0		0	0		0		0			No-ASE
C16orf88	16	19718333		0			0	0											0				0	No-ASE
IQCK	16	19729629				0										0							0	No-ASE
GPRC5B	16	19883228						0																No-ASE
ACSM1	16	20634846								0														No-ASE
THUMPD1	16	20748246	0						0														0	No-ASE
ACSM3	16	20781366	0			0	0		0		1									0			0	No-ASE
ERI2	16	20809303			0	0	0	0								0		0		0	0			No-ASE
DNAH3	16	20944511					0	0	0		0	0		0		1	0		1					No-ASE
TMEM159	16	21181846		0		0		0			0		0	0		0	0	0		0				No-ASE
ZP2	16	21208893				0	0																	No-ASE
ANKS4B	16	21245101				0		0			0		0	0										No-ASE
UQCRC2	16	21973828																	0					No-ASE
C16orf52	16	22092067												1							0			Heterogeneous_ASE
VWA3A	16	22111624	0	0					0									0		0			0	No-ASE
EEF2K	16	22237118																	0				0	No-ASE
POLR3E	16	22319517	0		0							0	0			0							0	No-ASE
USP31	16	23079408				0			0	0	0	0	0	0		0			0				0	No-ASE
SCNN1B	16	23360101																	0					No-ASE
GGA2	16	23489711						0	0	0						0		0					0	No-ASE
EARS2	16	23535717						0	0	0						0		0					0	No-ASE
PALB2	16	23614833							0														0	No-ASE
PLK1	16	23690573												0			0							No-ASE
ERN2	16	23702180						0																No-ASE
TNRC6A	16	24788549	0			0					0						0	0					0	No-ASE
SLC5A11	16	24873990	1	1			0							1									0	Heterogeneous_ASE
AQP8	16	25228368																0					0	No-ASE
ZKSCAN2	16	25251202	0	0	0					0		0		0		0	0				0			No-ASE
KDM8	16	27221524			0																			No-ASE
NSMCE1	16	27236495		0	0	0			0		0	0						0	0					No-ASE
IL4R	16	27353529		0	0	0	0	0	0		0			0				0	0	0			0	No-ASE
IL21R	16	27445682					1																	ASE
GTF3C1	16	27472706	0			0				0		0		0				0			0		0	No-ASE
KIAA0556	16	27585263	0	0				0		0		0											0	No-ASE
SH2B1	16	28877684				0																		No-ASE
CD19	16	28944271					0																	No-ASE
SPNS1	16	28990587			0	0					0												0	No-ASE
QPRT	16	29706444		0			0		0		0							0						No-ASE
CDIPT	16	29871906																	0					No-ASE
SEZ6L2	16	29883758				0				0		0												No-ASE
TAOK2	16	29989619					0		0		0					0		0	0				0	No-ASE
YPEL3	16	30104062											0	0										No-ASE
CD2BP2	16	30364712		0				0	0					0						0				No-ASE
TBC1D10B	16	30369434	0		0	0																		No-ASE
37135	16	30389757											0	0										No-ASE
ZNF48	16	30407177			0	0		0	0				0			0			0					No-ASE
ITGAL	16	30490515					0											0						No-ASE
PRR14	16	30663143		0			0				0		0	0					0				0	No-ASE
C16orf93	16	30772988																		0				No-ASE
FBXL19	16	30936081		0			0		0		0													No-ASE
SETD1A	16	30970202																					0	No-ASE
HSD3B7	16	30997074		0	0		0	0	0	0	0	0		0		0	0						0	No-ASE
ZNF646	16	31087733		0	0	0	0	0	0	0	0	0	0	0		0	1		0				0	No-ASE
PRSS53	16	31095115		0	0		0		0		0	0											0	No-ASE
KAT8	16	31138409			0			0		0		0	0			0					0			No-ASE
ITGAM	16	31273118												0										No-ASE
ITGAX	16	31366590	0								0												1	Heterogeneous_ASE
ARMC5	16	31471188	0	0	0	0		0		0	0									0				No-ASE
ZNF720	16	31765105				0																		No-ASE
ZNF267	16	31885289	0		0	0	0							0									0	No-ASE
PHKB	16	47497874								0		0		0										No-ASE
ABCC12	16	48121912							1															ASE
ABCC11	16	48201223	0		0	0	0					0		0				0			1			No-ASE
ZNF423	16	49525190			0	0												0						No-ASE
CNEP1R1	16	50059580							0		0					0								No-ASE
HEATR3	16	50100302					0																	No-ASE
ADCY7	16	50322149	0	0									0											No-ASE
SNX20	16	50703016																	0					No-ASE
NOD2	16	50731225				0		0				0		0		0		0	1				0	No-ASE
TOX3	16	52473564				0																		No-ASE
CHD9	16	53190992	0	0	0	0	0	0	0	0	0			0		0			0				0	No-ASE
RPGRIP1L	16	53639438		0						0	0							0						No-ASE
IRX3	16	54318158											0										0	No-ASE
IRX5	16	54967040					0	0										0						No-ASE
LPCAT2	16	55559453	0	0		0			0	0		0		0				0	0				0	No-ASE
CES5A	16	55880411					0											0						No-ASE
GNAO1	16	56374792																0						No-ASE
BBS2	16	56519510		0							0												0	No-ASE
MT4	16	56601720												0		1								Heterogeneous_ASE
SLC12A3	16	56899228																0						No-ASE
HERPUD1	16	56969148	0	0							0		0											No-ASE
CETP	16	56995908		0		0		0	0		0					0			0					No-ASE
NLRC5	16	57059280	0	0	0	0	0	0	0	0	0	0	0	0		0				0			0	No-ASE
CCL22	16	57392733																0						No-ASE
CIAPIN1	16	57465112																					0	No-ASE
COQ9	16	57484957					0						0											No-ASE
GPR114	16	57596058							1															ASE
GPR56	16	57684213				0		0	0	0		0		0										No-ASE
CCDC135	16	57732012	0	0					1		1					0		0					0	Heterogeneous_ASE
KIFC3	16	57792815							0															No-ASE
CNGB1	16	57921800				0	0									0		0		0				No-ASE
TEPP	16	58010422					0																	No-ASE
USB1	16	58036453		0		0				0	0													No-ASE
CCDC113	16	58287973	0								0													No-ASE

PRSS54	16	58318518				0												0		1				Heterogeneous_ASE
SLC38A7	16	58705081		0	0	0		0	0		0			0		0							0	No-ASE
GOT2	16	58742190		0					0	0		0	0	0							0			No-ASE
CDH11	16	64981824								1		1		0						0			1	Heterogeneous_ASE
CDH5	16	66413243			0		0					0	0	0		0								No-ASE
BEAN1	16	66511561																0						No-ASE
CMTM2	16	66613560		0									0											No-ASE
CDH16	16	66942338							1					0										Heterogeneous_ASE
CES3	16	66997136								0														No-ASE
CES4A	16	67034867							0					0		0								No-ASE
C16orf70	16	67144115		0					0					0										No-ASE
EXOC3L1	16	67218810							0					0		0			0		0			No-ASE
ELMO3	16	67233243	0			0		0	0		0	0		0		0			0	0			0	No-ASE
FHOD1	16	67264026							0					0		0								No-ASE
PLEKHG4	16	67314243							0					0					0		0			No-ASE
KCTD19	16	67324822						0																No-ASE
ZDHHC1	16	67432123							0					0										No-ASE
FAM65A	16	67572604							0					0		0					0			No-ASE
ACD	16	67691516		0		0		0	0				0	0				0					0	No-ASE
GFOD2	16	67708897			0				0					0										No-ASE
EDC4	16	67911257			0						0			0										No-ASE
CTRL	16	67963925			0						0			0										No-ASE
SLC12A4	16	67978795			0						0			0									0	No-ASE
DDX28	16	68055496				0		0																No-ASE
NFATC3	16	68119591																	0					No-ASE
SLC7A6OS	16	68336371	0			0	0			0			0	0		0		0						No-ASE
PRMT7	16	68349938						0																No-ASE
ZFP90	16	68597030								0		0												No-ASE
CDH3	16	68710362	0	0			0	0	0	0		0		0				0			0		0	No-ASE
TMED6	16	69377350	0											0										No-ASE
TERF2	16	69390938							0															No-ASE
NQO1	16	69744897	0								0	0				0		0					0	No-ASE
NOB1	16	69776426	0									0				0		0	0	0			0	No-ASE
PDPR	16	70154486	0	0	0	0	0		0			0		0	0	0				0	0		0	No-ASE
EXOSC6	16	70284990				1		1						1										ASE
AARS	16	70286631	0		0	0						0		0		0				0	0			No-ASE
DDX19B	16	70348839							0															No-ASE
FUK	16	70497136									0			0										No-ASE
COG4	16	70515032			0											0								No-ASE
IL34	16	70688459				0																		No-ASE
MTSS1L	16	70697927																	0					No-ASE
HYDIN	16	70841632	1	1		1	0	0				1		0		1		0						Heterogeneous_ASE
FTSJD1	16	71317518	0	0		0		0	0		0			0		0		0						No-ASE
CALB2	16	71406122						0					0											No-ASE
ZNF23	16	71482072		0			0	0	0	0						0							0	No-ASE
ZNF19	16	71509114		0				0	0	0		0											0	No-ASE
TAT	16	71602131	0			1					0													Heterogeneous_ASE
MARVELD3	16	71660310	0	0		0	0	0	0	0		0	0	0		0					0			No-ASE
PHLPP2	16	71682803				0					0		0	0					0				1	No-ASE
AP1G1	16	71773190	0	0		0								0					0				0	No-ASE
ATXN1L	16	71884209		0	0		0	0	0			0				0								No-ASE
PKD1L3	16	71967927	0			0	0		0			0	0	0										No-ASE
DHODH	16	72042682		0		0		0		0		0		0		0				0			0	No-ASE
DHX38	16	72130103										0												No-ASE
PMFBP1	16	72153820	0			0	0	0	0	0		0	0	0		0			0				0	No-ASE
ZFHX3	16	72821110		0		0		0	0	1	0					0		0			0		0	No-ASE
GLG1	16	74481327												0										No-ASE
RFWD3	16	74657852	0			0	0		0	0	0			0		0			0		0			No-ASE
MLKL	16	74708858			0	0	0	0																No-ASE
BCAR1	16	75263440				0		0	0	0			0	0				0	0	0	0			No-ASE
TMEM170A	16	75485625								0														No-ASE
CHST5	16	75563237									0									1				Heterogeneous_ASE
TMEM231	16	75573915				0		0																No-ASE
ADAT1	16	75634232	0		0			0		0									0	0				No-ASE
MON1B	16	77225440						0																No-ASE
SYCE1L	16	77245139	0			0		0			0							1						No-ASE
ADAMTS18	16	77317891					0											0	1					Heterogeneous_ASE
NUDT7	16	77756501	0	0	0	1				0	0					1								Heterogeneous_ASE
WWOX	16	78148935	0	0	0	0	0	1	0	0		0		0				0	0	0	0			No-ASE
CDYL2	16	80641994				0		0	0	1	0			0				0	0					No-ASE
CMC2	16	81010073			0									0									0	No-ASE
CENPN	16	81045614			0									0					0				0	No-ASE
ATMIN	16	81075056			0																			No-ASE
C16orf46	16	81087679		0	0		0	0											0					No-ASE
PKD1L2	16	81134859	0	1	0	0	0	0	1	1	0	1		0				0						Heterogeneous_ASE
BCMO1	16	81279208	0					0	0	1	0												0	No-ASE
PLCG2	16	81819605				0				0														No-ASE
SDR42E1	16	82032745	0			0			0		0							0			0			No-ASE
MPHOSPH6	16	82182368								0		1						0		0			0	No-ASE
MLYCD	16	83940604				0													0					No-ASE
OSGIN1	16	83991313														0							0	No-ASE
NECAB2	16	84012104								0				0									0	No-ASE
HSDL1	16	84158248	0		0	0	0	0	0							0		0	0					No-ASE
DNAAF1	16	84182628						0						0		0		0		0			0	No-ASE
TAF1C	16	84212597						0						0		0			0	0			0	No-ASE
ADAD2	16	84224856					0											0						No-ASE
KCNG4	16	84255963					0																	No-ASE
WFDC1	16	84328590				0	0																	No-ASE
ATP2C2	16	84438759	0	0	0	0		0	0		0					0			0		0			No-ASE
KIAA1609	16	84513560	0	0		0	0	0	0		0	0		0						0			0	No-ASE
CRISPLD2	16	84872225		0	0		0	0	0		0	0		0		0			1	0	0		0	No-ASE
GINS2	16	85711861																	0					No-ASE
COX4I1	16	85834818										0												No-ASE
MTHFSD	16	86565823	0					0			0			0				0	0		0			No-ASE
FOXL1	16	86613074	0								0													No-ASE
C16orf95	16	87339390	0		0	0	0	0	0	0	0			0		0			0				0	No-ASE
ZCCHC14	16	87445125			0		0		0	0						0			0	0			0	No-ASE
KLHDC4	16	87741992	0			0	0	0		0								0		0			0	No-ASE
BANP	16	88014652			0											0			0					No-ASE
ZNF469	16	88494947		1		0	0																0	No-ASE
ZFPM1	16	88552442		0															0					No-ASE
ZC3H18	16	88643695				0	1																	Heterogeneous_ASE
CYBA	16	88713182	0	0				0	0	0		0		1		0		0						No-ASE
CTU2	16	88773584	0	0	0	0	0	0		0	0	0		0		0		0	0	0				No-ASE
PIEZO1	16	88782198	0	0	0	0	0	0		0	0	0		0		0		0	0	0				No-ASE
CDT1	16	88871016			0	0	0			0						0		0	0	0			0	No-ASE
GALNS	16	88884459	0					0								0								No-ASE
ACSF3	16	89167094				0		0						0					0					No-ASE
ZNF778	16	89287559			0	0	0					0							0					No-ASE
ANKRD11	16	89341253				0	0					0								0				No-ASE
SPG7	16	89576947					0		0			0		0									0	No-ASE
DPEP1	16	89696828		0	0	0										0			0					No-ASE
C16orf55	16	89724661		0																				No-ASE
CDK10	16	89753146																					0	No-ASE
VPS9D1	16	89774288	0		0									0						0				No-ASE
ZNF276	16	89789073	0		0	0				0		0		0				0	0		0			No-ASE
FANCA	16	89809329	0		0	0		0		0		0		0		0		0	0	0			0	No-ASE
SPIRE2	16	89911747	0	0	0							0												No-ASE

TCF25	16	89940166																	0					No-ASE
MC1R	16	89985733	0		0	0	0			0	0			0										No-ASE
DEF8	16	90015976																0						No-ASE
CENPBD1	16	90037828	0	0		0				0	0							0	0	0				No-ASE
AFG3L1P	16	90066936	0	0	1	0				0	0							0	0	0				No-ASE
GAS8	16	90094071																0	0		0			No-ASE
RPH3AL	17	63683																					0	No-ASE
FAM101B	17	292985												0									0	No-ASE
VPS53	17	436105										0												No-ASE
FAM57A	17	641096										0		0										No-ASE
GEMIN4	17	648156										0		0									0	No-ASE
RNMTL1	17	685640		1								0		0										Heterogeneous_ASE
ABR	17	915979																		0			0	No-ASE
TUSC5	17	1183302				0						0												No-ASE
MYO1C	17	1371342				0																		No-ASE
PITPNA	17	1437427										0		0										No-ASE
SCARF1	17	1538083				0		0				0		0									1	No-ASE
RILP	17	1549888										0												No-ASE
WDR81	17	1629080				0		0				0		0									0	No-ASE
SERPINF2	17	1648294				0																		No-ASE
SERPINF1	17	1673212				0		0												1			0	No-ASE
SMYD4	17	1684605				0		0				0		0						0			0	No-ASE
RPA1	17	1782605				0		0						0									0	No-ASE
SMG6	17	1968369				0		0				0								0			0	No-ASE
TSR1	17	2228713										0								0				No-ASE
SGSM2	17	2240986						0												0			0	No-ASE
METTL16	17	2323437												0									0	No-ASE
CLUH	17	2595180						0				0												No-ASE
RAP1GAP2	17	2867232												0						0			1	Heterogeneous_ASE
SPATA22	17	3343611			1																			ASE
TRPV3	17	3417263										1		0						0			0	No-ASE
TRPV1	17	3470167				0		0						0									0	No-ASE
SHPK	17	3513856						0																No-ASE
CTNS	17	3550792				0		0						0						0				No-ASE
P2RX5-TAX1BP3	17	3567049				0						0		0						1			1	Heterogeneous_ASE
ITGAE	17	3619993						0												0			0	No-ASE
CAMKK1	17	3765508										0												No-ASE
ZZEF1	17	3912197						0						0						0				No-ASE
SPNS3	17	4337267				0		0				1								0			0	No-ASE
SPNS2	17	4434030										0												No-ASE
MYBBP1A	17	4443236												0						0			0	No-ASE
GGT6	17	4461467				0								0									0	No-ASE
SMTNL2	17	4495660						0						0									0	No-ASE
ALOX15	17	4535035																					0	No-ASE
PELP1	17	4574751				0						0												No-ASE
CXCL16	17	4638395			0	0																	0	No-ASE
VMO1	17	4688682				0		0				0		0										No-ASE
GLTPD2	17	4692323										0												No-ASE
PLD2	17	4711170				0								0									0	No-ASE
MINK1	17	4794285				0						0											0	No-ASE
CHRNE	17	4802111				0						0											1	Heterogeneous_ASE
GP1BA	17	4835895				0						0												No-ASE
ENO3	17	4855135				0														0			0	No-ASE
CAMTA2	17	4872066				0						0											0	No-ASE
INCA1	17	4892176				0														0				No-ASE
KIF1C	17	4903559						0																No-ASE
SLC52A1	17	4936370																					1	ASE
USP6	17	5033705		1							1									1				ASE
ZNF594	17	5085168				0		0																No-ASE
LOC100130950	17	5114223																		0			0	No-ASE
RABEP1	17	5241350												0										No-ASE
NUP88	17	5289546																		0				No-ASE
RPAIN	17	5326089				0		0				0		0						0			0	No-ASE
DHX33	17	5347599										0								0				No-ASE
NLRP1	17	5418093										1		0						1				Heterogeneous_ASE
PITPNM3	17	6367539						0						0									0	No-ASE
KIAA0753	17	6493107						0				0								0			0	No-ASE
LOC100506713	17	6900166																		0			0	No-ASE
RNASEK-C17orf49	17	6915911																		0				No-ASE
ELP5	17	7155861																		0			0	No-ASE
CLDN7	17	7163702				0								0									0	No-ASE
SLC2A4	17	7186603																					0	No-ASE
EIF5A	17	7215142				0																		No-ASE
GPS2	17	7216434				0																	0	No-ASE
NEURL4	17	7219552				0																	0	No-ASE
C17orf61-PLSCR3	17	7293715																					0	No-ASE
C17orf74	17	7329134												0										No-ASE
ZBTB4	17	7363088				0								0						0			0	No-ASE
POLR2A	17	7401414				0								0						0			0	No-ASE
TNFSF12-TNFSF13	17	7454275				0								0									0	No-ASE
SENP3	17	7466802																		0				No-ASE
CD68	17	7483200												1										ASE
MPDU1	17	7487223												0										No-ASE
ATP1B2	17	7557394				0								0									0	No-ASE
WRAP53	17	7591997				0		0															0	No-ASE
DNAH2	17	7623092												0										No-ASE
CNTROB	17	7836399				0						1		1						1				ASE
GUCY2D	17	7907178				0						0												No-ASE
ALOX15B	17	7942516				0								0									1	Heterogeneous_ASE
ALOX12B	17	7979002																		0				No-ASE
ALOXE3	17	7999967						0																No-ASE
PER1	17	8045136				0								0										No-ASE
AURKB	17	8108331												0						0				No-ASE
CTC1	17	8131548										0		0										No-ASE
PFAS	17	8157310				0		0				0		0						0				No-ASE
ODF4	17	8243371										1												ASE
MYH10	17	8378932				0																	0	No-ASE
SPDYE4	17	8656662										1												ASE
MFSD6L	17	8700719						0						0						1			0	No-ASE
STX8	17	9395230																		0				No-ASE
DHRS7C	17	9674858										1											1	ASE
GLP2R	17	9729445						1																ASE
MYH13	17	10206595				0																		No-ASE
MYH4	17	10346774				0																		No-ASE
MYH3	17	10533033										0												No-ASE
ADPRM	17	10608307				0		0				0								0			0	No-ASE
DNAH9	17	11511480				1																		ASE
ZNF18	17	11881356																		0				No-ASE
ELAC2	17	12896212				0		0												0			0	No-ASE
COX10	17	13977679				0						0		0						0				No-ASE
TEKT3	17	15207417				0																		No-ASE
TVP23C-CDRT4	17	15341161												0										No-ASE
CDRT1	17	15496727						0				0											1	Heterogeneous_ASE
TRIM16	17	15532095						0																No-ASE
TRPV2	17	16321176				0								0									0	No-ASE
C17orf76-AS1	17	16347193																		0			0	No-ASE
ZNF287	17	16455475										1												ASE
ZNF624	17	16525651																					0	No-ASE

FLCN	17	17118314						0																No-ASE
PEMT	17	17409125				0		0						0						0			0	No-ASE
RAI1	17	17696393				0						0												No-ASE
SREBF1	17	17715101				0		0				0												No-ASE
LRRC48	17	17881007				0																	0	No-ASE
MYO15A	17	18022823				0						0		0						0			1	No-ASE
ALKBH5	17	18111823				0								0										No-ASE
LLGL1	17	18133277						0				0		0									0	No-ASE
SMCR7	17	18164475						0				0		0										No-ASE
SMCR8	17	18219425				0		0				0		0						0			0	No-ASE
EVPLL	17	18284262				0		1				1								1			1	ASE
ZNF286B	17	18584142												1										ASE
PRPSAP2	17	18775900				0						0								0				No-ASE
EPN2	17	19186967																		0			0	No-ASE
MFAP4	17	19290088																		0				No-ASE
SLC47A2	17	19582075				0														0				No-ASE
ALDH3A1	17	19642348				0						0		0										No-ASE
ULK2	17	19683789										0								0				No-ASE
AKAP10	17	19812541						0				0								0			0	No-ASE
SPECC1	17	20000098		1		0						0				0				0			0	No-ASE
C17orf103	17	21144074						0						0						0			0	No-ASE
KCNJ12	17	21318671						1															1	ASE
WSB1	17	25628820	0			0			0				0			0	0			0			0	No-ASE
KSR1	17	25909701				0		0			0	0									0			No-ASE
LGALS9	17	25958304				0		0			0	0		0							0			No-ASE
NOS2	17	26086005				0	0				0			0										No-ASE
NLK	17	26490610				0	0																	No-ASE
SEBOX	17	26691310						0							0		0				0			No-ASE
VTN	17	26694784			0			0			0						0			0	0			No-ASE
SLC13A2	17	26800821	0				0						0					0						No-ASE
FOXN1	17	26851007										0				0								No-ASE
SPAG5-AS1	17	26937711											0	0									0	No-ASE
KIAA0100	17	26948488														0								No-ASE
SUPT6H	17	27008293											0										0	No-ASE
DHRS13	17	27224799	0										0					0			0			No-ASE
PHF12	17	27233221															0							No-ASE
SEZ6	17	27283248			0		0	0										0		0	0			No-ASE
MYO18A	17	27400983						0												0				No-ASE
ABHD15	17	27889602				0		0				0		0	0		0			0	0		0	No-ASE
TP53I13	17	27896342									0												0	No-ASE
CORO6	17	27942757														0								No-ASE
SSH2	17	27957950						0	0				0										0	No-ASE
EFCAB5	17	28268817			0						0		1		0									No-ASE
NSRP1	17	28443834																		0				No-ASE
BLMH	17	28576076					0	0					0		0					0				No-ASE
CRLF3	17	29111368							0				0	0									0	No-ASE
ATAD5	17	29161202			0						0			0			0			0			0	No-ASE
RNF135	17	29298304											0											No-ASE
NF1	17	29483047			0														0					No-ASE
UTP6	17	30195117			0				0			0	0				0			0				No-ASE
RHOT1	17	30521023										0												No-ASE
ZNF207	17	30687707			0								0					0						No-ASE
MYO1D	17	30821812	0																					No-ASE
SPACA3	17	31318972																		0				No-ASE
CCT6B	17	33255068	0				0		0								0							No-ASE
ZNF830	17	33288637			0								0											No-ASE
RAD51L3-RFFL	17	33427976											0	0									0	No-ASE
FNDC8	17	33448777				0																		No-ASE
NLE1	17	33460431				0						0		0							0		0	No-ASE
SLFN5	17	33585712	0		0	0					0		0	0		0	0	0		1	0			No-ASE
SLFN11	17	33679518				0	0					0				0		0						No-ASE
SLFN12	17	33738463					0																	No-ASE
SLFN13	17	33767943					0	1			1										1			ASE
SLFN12L	17	33802153					0	1				0	1								1			Heterogeneous_ASE
SLFN14	17	33875262	0			0	0				0							0						No-ASE
PEX12	17	33903070															0						0	No-ASE
GAS2L2	17	34071968	0		1		0	0	0									0						No-ASE
MMP28	17	34093663				0					0		0	0						0				No-ASE
C17orf66	17	34182092									0										0			No-ASE
RDM1	17	34245404		0			0																	No-ASE
CCL15-CCL14	17	34310899			1											0							0	Heterogeneous_ASE
CCL23	17	34340284	0												1								0	Heterogeneous_ASE
MYO19	17	34854121	0		0				0		0	0	0							0	0		0	No-ASE
PIGW	17	34893056										0									0			No-ASE
MRM1	17	34958256										0												No-ASE
TADA2A	17	35771468	0		0	0		0									0				0			No-ASE
SYNRG	17	35896125				0	0				0			0				0						No-ASE
DDX52	17	35979871				0	0				0			0										No-ASE
MRPL45	17	36455348					0																	No-ASE
GPR179	17	36482628					0																	No-ASE
SOCS7	17	36521269												0										No-ASE
SRCIN1	17	36700069												0										No-ASE
CISD3	17	36889541						0	0		0				0		0			0	0			No-ASE
PIP4K2B	17	36922196	0			0	0		0		0	0		0	0	0	0				0		0	No-ASE
C17orf98	17	36991482					0				1													Heterogeneous_ASE
LASP1	17	37054772					0	0	0		0		0		0	0					0			No-ASE
LOC100131347	17	37223126	0			0		0									0							No-ASE
ARL5C	17	37316988	0					0																No-ASE
STARD3	17	37809912	0				0		0		0	0			0		0	0		0				No-ASE
ERBB2	17	37863269					0						0	0		0				0			0	No-ASE
IKZF3	17	37922198	0						0				0					0		0	0		0	No-ASE
GSDMB	17	38061072	0						0				0	0				0			0		0	No-ASE
GSDMA	17	38122095	0			0					0			0		0	0	0			0		0	No-ASE
PSMD3	17	38140666										0												No-ASE
MED24	17	38176038							0															No-ASE
CDC6	17	38445811				0					0			0							0			No-ASE
TOP2A	17	38545193			0	0	0				0									0				No-ASE
TNS4	17	38633854			0		0	0	0			0		0		0		0		0				No-ASE
CCR7	17	38711073				0																		No-ASE
KRT26	17	38922790					0																	No-ASE
KRT27	17	38933291					0																	No-ASE
KRT28	17	38948714					0																	No-ASE
KRT10	17	38974733			0																			No-ASE
TMEM99	17	38990780			0		0	0				0		0		0				0	0		0	No-ASE
KRT12	17	39019462											0											No-ASE
KRT20	17	39036190							0				0											No-ASE
KRT23	17	39081614			0				0		0			0		0				0			1	No-ASE
KRT39	17	39114897			0								1		0						0			No-ASE
KRT40	17	39134541	1		0	1		0					1			1		1			0			Heterogeneous_ASE
KRT37	17	39577180	0		0			1	0		0		1	0			1			0				Heterogeneous_ASE
KRT32	17	39616430							0		0			0						0	0		0	No-ASE
KRT35	17	39634732									0				0	0				0			1	No-ASE
KRT36	17	39643230																		0			0	No-ASE
KRT13	17	39657519						1			0		1	0						0				Heterogeneous_ASE
KRT15	17	39670912				0		0	0				0			0	0			0			0	No-ASE
KRT19	17	39680084				0																		No-ASE
HAP1	17	39880545	0		0	0	0	0	0		0			0	0	0	0	0		1	0		1	No-ASE
JUP	17	39912027															0							No-ASE

LEPREL4	17	39965958	0		0			0	0		0		0		0	0				0			0	No-ASE
NT5C3B	17	39983700		1			0	1								0	0			0	0		0	No-ASE
ACLY	17	40039454	0				0									0	0			0	0		0	No-ASE
TTC25	17	40086978									0													No-ASE
DHX58	17	40253856						0				0		0		0	0							No-ASE
HSPB9	17	40274873										0					0				0			No-ASE
GHDC	17	40341789											0											No-ASE
STAT3	17	40466092							0			0	0		0	0	0			0	0		0	No-ASE
ATP6V0A1	17	40612929				0																		No-ASE
COASY	17	40714478		1		0																	0	Heterogeneous_ASE
MLX	17	40719173				0			0				0		0	0	0							No-ASE
FAM134C	17	40733952										0												No-ASE
TUBG2	17	40817712																					0	No-ASE
AOC2	17	40996645						0																No-ASE
AOC3	17	41003381							0								0							No-ASE
PTGES3L-AARSD1	17	41103839						0	0															No-ASE
RUNDC1	17	41132622										0		0										No-ASE
IFI35	17	41158985												0									0	No-ASE
BRCA1	17	41215385	0				0	0	0		0	0	0	0		0	0	0					0	No-ASE
NBR1	17	41329986					0					0	0	0		0	0						0	No-ASE
ETV4	17	41606033			0																			No-ASE
MPP3	17	41879074											0											No-ASE
CD300LG	17	41925962					0																	No-ASE
PYY	17	42030528			1				1					0			0							Heterogeneous_ASE
HDAC5	17	42155892	0														0							No-ASE
C17orf53	17	42225250	0				0						0		0		0							No-ASE
ASB16	17	42248212					0				0		0											No-ASE
SLC4A1	17	42327861														0								No-ASE
SLC25A39	17	42397386			0	0							0	0	0	0								No-ASE
C17orf104	17	42743946										0					0						0	No-ASE
DBF4B	17	42786711					0																	No-ASE
HIGD1B	17	42925537	0		0	0	0		0		0	0						0			0			No-ASE
GFAP	17	42985442					0																	No-ASE
DCAKD	17	43101382				0					0	0		0	0						0		0	No-ASE
NMT1	17	43159028			0	0								0									0	No-ASE
PLCD3	17	43190256															0							No-ASE
ACBD4	17	43212963					0		0						0								0	No-ASE
MAP3K14-AS1	17	43332512			0	0							0	0			0				0		0	No-ASE
ARHGAP27	17	43473587															0							No-ASE
PLEKHM1	17	43513441			0	0	0																	No-ASE
CRHR1	17	43714850			0	0	0				0													No-ASE
MAPT-AS1	17	43922348		1	0		0				0													No-ASE
MAPT	17	44039717				0	0				0													No-ASE
KANSL1	17	44108906	0	0	0	0	0		0		0	0			0			0		0	0		0	No-ASE
NSF	17	44791322	0		0	0					0													No-ASE
WNT9B	17	44949890										0												No-ASE
GOSR2	17	45006896	0			0	0				0		0		0									No-ASE
EFCAB13	17	45412575															0							No-ASE
MRPL10	17	45901633	0			0			0		0	0		0			0						0	No-ASE
SCRN2	17	45915364				0					0						0							No-ASE
SP2	17	45993622												0							0			No-ASE
CDK5RAP3	17	46050894				0					0													No-ASE
SKAP1	17	46239844		1	0		0									0					0			No-ASE
HOXB3	17	46627884													0									No-ASE
HOXB9	17	46700352			0		1																	Heterogeneous_ASE
HOXB13	17	46804175			0																			No-ASE
TTLL6	17	46846430					0		0		0												1	No-ASE
CALCOCO2	17	46925777	0								0				0			0			0		0	No-ASE
UBE2Z	17	47005193					0					0	0		0		0				0			No-ASE
GIP	17	47036073					0								0			0						No-ASE
IGF2BP1	17	47075135					0																	No-ASE
B4GALNT2	17	47210469					0					0						0						No-ASE
ABI3	17	47293906	0																					No-ASE
NGFR	17	47579431	0																					No-ASE
DLX4	17	48046960	0														0							No-ASE
ITGA3	17	48141461										0												No-ASE
PPP1R9B	17	48216860											0	0										No-ASE
XYLT2	17	48431021			0						0	0		0		0	0	0					0	No-ASE
EME1	17	48452584	0		0		0				0	0		0		0	0	0			0		0	No-ASE
RSAD1	17	48557326	0									0		0	0	0		0			0			No-ASE
MYCBPAP	17	48594690	0	0		0					0	0		0	0	0	0	0			0		0	No-ASE
SPATA20	17	48625667	0									0	0	0				0						No-ASE
ABCC3	17	48733349			0		0								0		0							No-ASE
WFIKKN2	17	48913321	0			0	0						0	0									0	No-ASE
KIF2B	17	51900500																0						No-ASE
STXBP4	17	53046176			0	0							0	0									0	No-ASE
ANKFN1	17	54305317												0			0						0	No-ASE
C17orf67	17	54872439			0				0															No-ASE
COIL	17	55023857				0																		No-ASE
AKAP1	17	55182878												0			0				0			No-ASE
MRPS23	17	55916641	0		0				0			0	0			0								No-ASE
CUEDC1	17	55944723	0																				0	No-ASE
OR4D1	17	56232675												0										No-ASE
OR4D2	17	56247029	0		1						0	1		0			1							Heterogeneous_ASE
EPX	17	56270425	0				0							0									1	No-ASE
MPO	17	56348106				1						1		0				0						Heterogeneous_ASE
BZRAP1	17	56382477	0			0			0			0	0	0	0			0			0		0	No-ASE
RNF43	17	56435046	0		0	0			0		0	0	0	0		0		0			0		0	No-ASE
HSF5	17	56536257					0					0												No-ASE
MTMR4	17	56569083	0		0		0					0	0					0					0	No-ASE
38231	17	56597842	0								0			0										No-ASE
C17orf47	17	56619253			0		0		0			0			0								1	No-ASE
TEX14	17	56636922					0					0	1		0		0	0					0	No-ASE
RAD51C	17	56770018									0													No-ASE
PRR11	17	57262419																					0	No-ASE
SMG8	17	57287425									0													No-ASE
PTRH2	17	57775172	0																					No-ASE
TUBD1	17	57941133	0		0	0			0				0	0	0									No-ASE
RPS6KB1	17	57970574							0				0	0										No-ASE
HEATR6	17	58120931			0		0																	No-ASE
BCAS3	17	58786625												0										No-ASE
NACA2	17	59667898		1	1				1			1		1	1						1		1	ASE
BRIP1	17	59760963					0						0	0	0	0								No-ASE
MRC2	17	60741917					0		1			0					0							No-ASE
TANC2	17	61278229	0				0											0						No-ASE
ACE	17	61556410	0			0					0		0	0	0	0					0			No-ASE
KCNH6	17	61601563			0		0											0						No-ASE
DCAF7	17	61628111												0										No-ASE
DDX42	17	61869806										0									0			No-ASE
FTSJ3	17	61897218	0						0					0			0	0			0			No-ASE
PSMC5	17	61905516			0	0	0		0					0			0							No-ASE
SCN4A	17	62016704	0				0											0						No-ASE
C17orf72	17	62079123				0					0	0			0	0	0	0					0	No-ASE
PECAM1	17	62401118			0	0						1												Heterogeneous_ASE
POLG2	17	62476451			0				0		0													No-ASE
GNA13	17	63010416																					0	No-ASE
AXIN2	17	63526198	0								0		0		0	0	0				0			No-ASE
CEP112	17	63637082	0		0	0						0		0	0		1	0			0		0	No-ASE

APOH	17	64208285																0						No-ASE
PRKCA	17	64683255													0									No-ASE
HELZ	17	65074702										0	0					0			0			No-ASE
C17orf58	17	65988049				0						0	0	0			0							No-ASE
AMZ2	17	66246353			0				0		0		0		0	0							0	No-ASE
ARSG	17	66267008	0		1	0	0				0	0			0		0	0			0		0	No-ASE
WIPI1	17	66422292										0	0	0			0							No-ASE
PRKAR1A	17	66526468	0			0	0					0	0		0	0					0		0	No-ASE
ABCA6	17	67075361	0				0					1					0							No-ASE
ABCA10	17	67145188			0												0							No-ASE
ABCA5	17	67247900	0		0		0		0		0		0	0	0	0	0	0					0	No-ASE
SLC39A11	17	70645012												0			0							No-ASE
COG1	17	71189266	0						0		0	0	0			0	0				0			No-ASE
FAM104A	17	71205787				0							0											No-ASE
C17orf80	17	71231868	0		0						0	0	0	0	0						0			No-ASE
SDK2	17	71334757											1				0	0						Heterogeneous_ASE
TTYH2	17	72227032			0	0	0		0			0			0						0			No-ASE
KIF19	17	72338037					0										1	0						Heterogeneous_ASE
GPRC5C	17	72428206														0								No-ASE
CD300LD	17	72584662	0						1		0		0					0						No-ASE
C17orf77	17	72588388	0																					No-ASE
CD300E	17	72610089			0						1	0	0		0			0						No-ASE
RAB37	17	72691263	0									0												No-ASE
NAT9	17	72768191										0												No-ASE
TMEM104	17	72781693										0	0	0				0					0	No-ASE
GRIN2C	17	72838645					0					0						0						No-ASE
OTOP2	17	72920927	0		0		0					0												No-ASE
OTOP3	17	72931912	0				0								1			0						No-ASE
KCTD2	17	73049110											0											No-ASE
NT5C	17	73126662														0								No-ASE
GGA3	17	73233599				0					0		0			0								No-ASE
CASKIN2	17	73497583				0																		No-ASE
TSEN54	17	73513281					0		0		0							0			0			No-ASE
LLGL2	17	73539559			0	0	0		0				0		0	0		0			0		0	No-ASE
RECQL5	17	73623542												0				0						No-ASE
ITGB4	17	73720794	0		0				0		0	0	0										0	No-ASE
UNC13D	17	73824982	0		0	0			0			0	0				0				0			No-ASE
TRIM65	17	73886888	0		0	0			0		0		0								0			No-ASE
FBF1	17	73908358	0		0	0	1		0		0		0				0				0			No-ASE
ACOX1	17	73944496	0		0				0		0	0	0				0				0			No-ASE
EVPL	17	74003204			0		0										0							No-ASE
ZACN	17	74075408		0	0		0		0		0	0	0		0	0							1	No-ASE
RNF157	17	74154496														0								No-ASE
QRICH2	17	74271936			0	0						0	0		0		0	0					0	No-ASE
SPHK1	17	74381555					0					0						0						No-ASE
UBE2O	17	74387150	0				0		0					0				0					0	No-ASE
RHBDF2	17	74467762											0											No-ASE
ST6GALNAC2	17	74562292										0												No-ASE
ST6GALNAC1	17	74621447			0																			No-ASE
MGAT5B	17	74868836					0																	No-ASE
SEC14L1	17	75186930									0	0												No-ASE
TNRC6C	17	76045264			0																			No-ASE
TMC6	17	76109263			0											0	0	0						No-ASE
TMC8	17	76128532	0				0		0		0			0	0	0		0						No-ASE
C17orf99	17	76157044	0			0					0		0	0				0						No-ASE
AFMID	17	76187091	0		0	0																		No-ASE
BIRC5	17	76210422									0													No-ASE
TMEM235	17	76230693					0																	No-ASE
PGS1	17	76395430	0			0	0		0		0		0		0	0	0							No-ASE
DNAH17	17	76421535	0		0	0	0		0		0	0	1	0	0	0		0			0		1	No-ASE
USP36	17	76794506			0						0	0	0	0		0								No-ASE
LGALS3BP	17	76967674															0							No-ASE
ENGASE	17	77073901													0									No-ASE
CBX8	17	77768654			0								0											No-ASE
TBC1D16	17	77914743									0		0	0										No-ASE
CCDC40	17	78011924										0	0	0										No-ASE
GAA	17	78078417	0			0			0		1			0	0	0	0	0					0	No-ASE
CARD14	17	78155422	0			0	0		0		0	0	0	0	0			0			0		0	No-ASE
SGSH	17	78184298	0						0		0	0		0	0									No-ASE
RNF213	17	78247045	0		0	0			0			0	0	0		0	0	0			0		0	No-ASE
ENDOV	17	78389020					0										0				0			No-ASE
RPTOR	17	78599592							0								0						0	No-ASE
BAIAP2	17	79060247				0			0			0						0						No-ASE
AATK	17	79093324			0									0										No-ASE
AZI1	17	79163758				0			0		0		0			0	0							No-ASE
ENTHD2	17	79202732	0																					No-ASE
SLC38A10	17	79219621	0																					No-ASE
BAHCC1	17	79405446				0	0						0	0			0				0			No-ASE
C17orf70	17	79508395				0	0				0	0	0	0		0		0					0	No-ASE
NPLOC4	17	79526379	0									0			0			0						No-ASE
TSPAN10	17	79612022	0																				1	Heterogeneous_ASE
PDE6G	17	79620262																					1	ASE
CCDC137	17	79634774			0								0	0										No-ASE
HGS	17	79653380			0						0		0											No-ASE
MRPL12	17	79671332			0						0		0					0						No-ASE
MYADML2	17	79898714	0						0									0						No-ASE
ASPSCR1	17	79941516							0								0						0	No-ASE
LRRC45	17	79983359																0						No-ASE
RAC3	17	79991701																					0	No-ASE
DCXR	17	79993919											0											No-ASE
RFNG	17	80006602			0	0	0		0		0										0		0	No-ASE
FASN	17	80037253	0				0																	No-ASE
CCDC57	17	80059637			0		0		0					0	0			0					0	No-ASE
CD7	17	80273225				1							0											Heterogeneous_ASE
SECTM1	17	80280054	1			0	0				0	0		0	0	0		0					0	No-ASE
OGFOD3	17	80350284			0	0					0	0	0		0						0		0	No-ASE
FOXK2	17	80477918	0										0											No-ASE
WDR45B	17	80573830				0	0														0			No-ASE
RAB40B	17	80615788	0																					No-ASE
FN3KRP	17	80674717			0		0				0	0	0	0			0							No-ASE
FN3K	17	80693643			0	0						0											0	No-ASE
TBCD	17	80724159	0		0		0				0		0	0	0	0	0	0					0	No-ASE
B3GNTL1	17	80904844			0	0					0													No-ASE
METRNL	17	81042978				0						0									0			No-ASE
USP14	18	163340							0			0				0								No-ASE
ENOSF1	18	674325						0				0								0				No-ASE
YES1	18	724385		0																				No-ASE
METTL4	18	2539016										0				0							0	No-ASE
NDC80	18	2573067						0				0											0	No-ASE
SMCHD1	18	2656248		0		0		0		0		0		0		0	0			0	0		0	No-ASE
EMILIN2	18	2847890		0					0															No-ASE
MYOM1	18	3067357		0					0			0		1		0	0			1	0		1	Heterogeneous_ASE
TGIF1	18	3452030				0											0						0	No-ASE
DLGAP1	18	3534199		0				0				1		0		1				0	0		0	No-ASE
EPB41L3	18	5395098				0								0										No-ASE
L3MBTL4	18	5956224																					0	No-ASE
LAMA1	18	6942160														0				0			1	Heterogeneous_ASE
SOGA2	18	8718522		0		0		0						0			0			0			1	No-ASE

ANKRD12	18	9195598						0				0		0									0	No-ASE
RALBP1	18	9517221				0																		No-ASE
PPP4R1	18	9547898						0				0		0		0	0						0	No-ASE
VAPA	18	9931945						0												0				No-ASE
PIEZO2	18	10671666							1											1				ASE
TUBB6	18	12308316		0				0						0										No-ASE
AFG3L2	18	12329611				0																		No-ASE
PSMG2	18	12674711		0		0				0		0											0	No-ASE
SEH1L	18	12948134		0		0			0	0		0				0	0			0			0	No-ASE
CEP192	18	12999533				0			0	0		0		0		0	0			0			0	No-ASE
FAM210A	18	13671940		0		0																		No-ASE
MC5R	18	13825871		0												0								No-ASE
ZNF519	18	14104957		0										0		0	0							No-ASE
POTEC	18	14543057				0																		No-ASE
ESCO1	18	19153303				0						0								0			0	No-ASE
RBBP8	18	20548849				0																		No-ASE
TMEM241	18	20889671				0		0		0													1	No-ASE
NPC1	18	21112182						0				0					0			0			0	No-ASE
LAMA3	18	21294020						0		0														No-ASE
CABYR	18	21723194								0		0		0									0	No-ASE
OSBPL1A	18	21743216										0		0										No-ASE
IMPACT	18	22008833				0		0												0				No-ASE
TAF4B	18	23807184								0				0										No-ASE
KCTD1	18	24035739						0		0				0						0				No-ASE
DSC3	18	28574152		0		0				0		0		0									0	No-ASE
DSC2	18	28648100										0								0				No-ASE
DSC1	18	28710560																		0				No-ASE
DSG1	18	28898294				0		0				0		0						0			0	No-ASE
DSG4	18	28956904						0		0														No-ASE
DSG3	18	29038537								1														ASE
DSG2	18	29098211				0		0				0											0	No-ASE
TRAPPC8	18	29410750		1		0		0						0						0				No-ASE
RNF125	18	29617167										0												No-ASE
MEP1B	18	29782888										0											1	Heterogeneous_ASE
GAREM	18	29848028																					0	No-ASE
ASXL3	18	31187565				0		1															1	Heterogeneous_ASE
ZNF397	18	32822472						0												0			0	No-ASE
ZNF24	18	32917274			1									0									0	Heterogeneous_ASE
C18orf21	18	33552862						0				0												No-ASE
ELP2	18	33709977												0										No-ASE
MOCOS	18	33775295						0				0								0				No-ASE
FHOD3	18	34156464										0		0										No-ASE
KIAA1328	18	34465613										0												No-ASE
SETBP1	18	42281472												0						0				No-ASE
SLC14A1	18	43307246										0		0										No-ASE
SIGLEC15	18	43405725																		0				No-ASE
EPG5	18	43435600						0				0								0			0	No-ASE
C18orf25	18	43795986						0																No-ASE
KATNAL2	18	44559399				0																	0	No-ASE
ZBTB7C	18	45555783												0										No-ASE
SMAD7	18	46447801				0																		No-ASE
LIPG	18	47088754												0										No-ASE
MYO5B	18	47352892						0				0								0				No-ASE
CCDC11	18	47753755						0				0		0						0				No-ASE
MBD1	18	47796089						0															0	No-ASE
SKA1	18	47908542						0						0						0				No-ASE
MAPK4	18	48190329				0																	0	No-ASE
MRO	18	48326426				0						0											1	Heterogeneous_ASE
MBD2	18	51681244				0								0									0	No-ASE
POLI	18	51795960				0						0		0									0	No-ASE
C18orf54	18	51887025						0																No-ASE
CCDC68	18	52571597				0		0						0									1	No-ASE
FECH	18	55221518										0		0									0	No-ASE
LOC100505549	18	55315751																					0	No-ASE
NEDD4L	18	55711905				0								0										No-ASE
ALPK2	18	56149099										0		0										No-ASE
ZNF532	18	56586019												0						0				No-ASE
LMAN1	18	56998735				1																	0	Heterogeneous_ASE
PIGN	18	59713102																					0	No-ASE
TNFRSF11A	18	60015458				0								0										No-ASE
SERPINB5	18	61151704				0														0			0	No-ASE
SERPINB13	18	61256025																		0				No-ASE
SERPINB11	18	61377495																		0				No-ASE
SERPINB7	18	61449746																					1	ASE
SERPINB10	18	61582751										1												ASE
SERPINB8	18	61645628				0						0		0										No-ASE
CDH7	18	63476982																					0	No-ASE
DSEL	18	65178235												0									1	Heterogeneous_ASE
CCDC102B	18	66504176												1										ASE
RTTN	18	67673697				0						0												No-ASE
TIMM21	18	71816278																		0				No-ASE
CYB5A	18	71920841				0						0		0										No-ASE
CNDP1	18	72201918												0										No-ASE
ZNF407	18	72343156				0																	0	No-ASE
TSHZ1	18	72997677										0											0	No-ASE
ZNF516	18	74082537				0																		No-ASE
SALL3	18	76753726						0																No-ASE
ATP9B	18	76829468				0																		No-ASE
NFATC1	18	77170454				0		0															0	No-ASE
CTDP1	18	77455227										0												No-ASE
HSBP1L1	18	77728135						0				0		0										No-ASE
RBFA	18	77797400				0														0				No-ASE
ADNP2	18	77891027				0						0											0	No-ASE
PPAP2C	19	281405	0	0				0	0	0		0			0					0			0	No-ASE
MIER2	19	307163			0																			No-ASE
THEG	19	362283		1												0								Heterogeneous_ASE
C2CD4C	19	407900		0																				No-ASE
MADCAM1	19	498782																		0				No-ASE
BSG	19	572671										0	0											No-ASE
POLRMT	19	617466						0		0														No-ASE
PALM	19	727021	0		1	0						0							0	1			0	Heterogeneous_ASE
PRTN3	19	844020		0				0																No-ASE
MED16	19	868108	0	0					0	0		0												No-ASE
WDR18	19	984370			0							0		0									0	No-ASE
GRIN3B	19	1003172	0	0				0	0	0		0		0	0								1	No-ASE
TMEM259	19	1010755						0																No-ASE
ABCA7	19	1041843		0	0				0	0		0	0	0	0				0	0			1	No-ASE
HMHA1	19	1068428							0			0	0							0				No-ASE
MUM1	19	1357048	0	0				0		0			0		0	0			0				0	No-ASE
NDUFS7	19	1388538	0	0				0							0	0			0	0			0	No-ASE
PCSK4	19	1481787	0		0																			No-ASE
ADAMTSL5	19	1506135																					0	No-ASE
TCF3	19	1611720				0									0								0	No-ASE
ATP8B3	19	1783027	0	1	0			0		0		0	1		0					0			0	No-ASE
REXO1	19	1816124		0	0	0				0			0		0				0	0				No-ASE
FAM108A1	19	1880904	0			0				0														No-ASE
ADAT3	19	1912165						0																No-ASE
BTBD2	19	1986931							0															No-ASE

MKNK2	19	2039727		0								0	0	0									0	No-ASE
AP3D1	19	2102198		0					0				0											No-ASE
DOT1L	19	2191238		0	0			0	0							0								No-ASE
AMH	19	2249384												1									1	ASE
JSRP1	19	2252525																		1				ASE
SPPL2B	19	2337520		0	0							0								0			0	No-ASE
TMPRSS9	19	2389793	0	0	0							0							0	0			1	No-ASE
SLC39A3	19	2732781														0								No-ASE
ZNF554	19	2823043	0											0		1							0	No-ASE
ZNF555	19	2851485	0			0						0		0	0								0	No-ASE
ZNF57	19	2915619	0	0	0				0				0	0						0				No-ASE
ZNF77	19	2933698										0												No-ASE
TLE2	19	2997866								0														No-ASE
MFSD12	19	3539203	0		0			0		0			0	0	0				0					No-ASE
CACTIN	19	3618958				0																	0	No-ASE
TJP3	19	3728464	0		0	0				0			0		0	0				0			0	No-ASE
APBA3	19	3751082	0		0	0				0			0	0	0	0				0				No-ASE
MRPL54	19	3762705	0		0				0	0					0				0				0	No-ASE
EEF2	19	3979842			0									0										No-ASE
ANKRD24	19	4199923	0							0		0								0			0	No-ASE
EBI3	19	4233301		0																				No-ASE
CCDC94	19	4247167						0																No-ASE
SHD	19	4280332	0			0			0	0				0	0									No-ASE
TMIGD2	19	4292621																					1	ASE
MPND	19	4345965													0									No-ASE
CHAF1A	19	4409141	0		0							0		0									0	No-ASE
UBXN6	19	4445541	0		0							0		0									1	No-ASE
PLIN4	19	4504551							0					0										No-ASE
LRG1	19	4538013		1	0							0								0				No-ASE
SEMA6B	19	4546231				0																		No-ASE
TNFAIP8L1	19	4652038																		0				No-ASE
C19orf10	19	4660006	0		0					0			0							0			0	No-ASE
PLIN3	19	4839274	0	0	0	0							0						0	0			0	No-ASE
UHRF1	19	4910889	0	0				0		0			0	0										No-ASE
KDM4B	19	5041262													0									No-ASE
PTPRS	19	5208046				0																		No-ASE
ZNRF4	19	5455515		1					1			1												ASE
LONP1	19	5692087		0				0				0	0	0									0	No-ASE
FUT3	19	5844400			0	0		0							0				0				0	No-ASE
FUT5	19	5867247			0																			No-ASE
VMAC	19	5908969										0												No-ASE
LOC100128568	19	5994913						0																No-ASE
RFX2	19	6026209			0	0				0		0		0	0	0			0	0				No-ASE
ACSBG2	19	6141573																	0					No-ASE
MLLT1	19	6218024				0																		No-ASE
ALKBH7	19	6374554											0	0		0							0	No-ASE
PSPN	19	6375505				0																		No-ASE
GTF2F1	19	6380372										0												No-ASE
KHSRP	19	6418004										0			0									No-ASE
SLC25A41	19	6426528																		1				ASE
DENND1C	19	6467589	0	0	0	0			0			0	0								0			No-ASE
C3	19	6678030	0						0	0		0	0			0								No-ASE
TRIP10	19	6741289						0				0			0									No-ASE
SH2D3A	19	6752671	0		0			0						0		0			0					No-ASE
VAV1	19	6822299								0			0											No-ASE
EMR1	19	6887624				1									1									ASE
ZNF557	19	7075115	0	0					0				0	0	0				0				0	No-ASE
INSR	19	7117197								0														No-ASE
ARHGEF18	19	7505163										0								0			0	No-ASE
PEX11G	19	7542168			0			0						0									0	No-ASE
C19orf45	19	7565735						0	0					0		0			0	0			0	No-ASE
ZNF358	19	7584223	0	0		0																	0	No-ASE
MCOLN1	19	7589856	0	0		0														0			0	No-ASE
PNPLA6	19	7605870		0		0																		No-ASE
STXBP2	19	7703623	0		0	1		0		0				0	0	0				0				No-ASE
TRAPPC5	19	7747293	0	0	0	0		0				0		0						0				No-ASE
FLJ22184	19	7933962	0	0				0		0										0				No-ASE
SNAPC2	19	7986372	0	0		0						0		0	0					0			0	No-ASE
TIMM44	19	7992091	0												0								0	No-ASE
CCL25	19	8117967				0							1		1									Heterogeneous_ASE
FBN3	19	8130867	0	0		0							0		0									No-ASE
CERS4	19	8316043			1														0				0	Heterogeneous_ASE
CD320	19	8367424																	0					No-ASE
KANK3	19	8387803				0						0		0						1				No-ASE
ANGPTL4	19	8429323			0			0		0						0								No-ASE
mrt-02	19	8486882				0						0	0		0	0				0				No-ASE
PRAM1	19	8555109	0																					No-ASE
MUC16	19	8959693	0	0	0	0			1			1	1	0	1	0				0			1	Heterogeneous_ASE
OR7G1	19	9225562						1		1														ASE
ZNF317	19	9267302		0	0			0					0											No-ASE
ZNF559	19	9449235																		0				No-ASE
ZNF426	19	9639062				0													0	0				No-ASE
ZNF561	19	9720904													0									No-ASE
ZNF846	19	9868152																				0		No-ASE
OLFM2	19	9965332	0						0	0		0	0		0					1		0		No-ASE
COL5A3	19	10071227						1					0							0				Heterogeneous_ASE
RDH8	19	10124190												0	1									Heterogeneous_ASE
PPAN-P2RY11	19	10218508	0	0	0					0		0	0	0		0						0	0	No-ASE
DNMT1	19	10247806		0		0													0					No-ASE
S1PR2	19	10334725																					0	No-ASE
ICAM1	19	10385517		0	0	0				0		0	0		0				0			0	1	No-ASE
ICAM5	19	10402720		0		0						0										0	0	No-ASE
ZGLP1	19	10415781											0							0		0		No-ASE
RAVER1	19	10428162										0	0											No-ASE
ICAM3	19	10444558	0	0				0					1							0				No-ASE
TYK2	19	10461514	0		0	0			0			0		0	0								0	No-ASE
PDE4A	19	10541683	0																				0	No-ASE
KRI1	19	10664632			0	0		0					0	0					0	1		0		No-ASE
AP1M2	19	10685094						0					0						0	0		0		No-ASE
SLC44A2	19	10736346	0			0			0			0	0	0	0									No-ASE
ILF3	19	10782188		0	0	0						0	0		0									No-ASE
LDLR	19	11210912				0		0				0												No-ASE
KANK2	19	11275139		0	0	0			0			0		0	0				0				0	No-ASE
DOCK6	19	11311464		0	0	0			0			0	0	0	0				0	0		0	0	No-ASE
CCDC159	19	11460637										0												No-ASE
RGL3	19	11505122		0	0	0			0			0	1	0	0	0			1	0		0	1	No-ASE
CCDC151	19	11531272																		0				No-ASE
PRKCSH	19	11546269				0							0	0	0								0	No-ASE
ZNF653	19	11597866			0														0					No-ASE
ECSIT	19	11617079																	0					No-ASE
ACP5	19	11685943							0					0		0								No-ASE
ZNF823	19	11832649	0																					No-ASE
ZNF441	19	11890983	0	0								0	0	0	0					0				No-ASE
ZNF491	19	11916890				0																		No-ASE
ZNF700	19	12058083	0		0							0	0							0				No-ASE
ZNF878	19	12154684											0							0				No-ASE
ZNF844	19	12186272								1									0					Heterogeneous_ASE
ZNF44	19	12383526	0	0						0														No-ASE

ZNF563	19	12429912			0																			No-ASE
ZNF442	19	12460517		0		0																	0	No-ASE
ZNF443	19	12541214				0							0											No-ASE
ZNF490	19	12691484	0	0	0				0	0				0						0		0	0	No-ASE
MAN2B1	19	12757477	0		0	0		0	0	0		0		0	0	0			0	0	0		0	No-ASE
FBXW9	19	12800212																	0					No-ASE
MAST1	19	12954416										0		1	0	0						0		No-ASE
GCDH	19	13002779		0				0		0		0				0	1					0	0	No-ASE
SYCE2	19	13010874			0																			No-ASE
FARSA	19	13033677	0					0						0	0									No-ASE
RAD23A	19	13059006			0			0				0												No-ASE
DAND5	19	13080720																		0				No-ASE
IER2	19	13264338										0								0		0		No-ASE
CACNA1A	19	13318811																				0		No-ASE
MRI1	19	13875421								0														No-ASE
C19orf53	19	13885484	0	0				0														0		No-ASE
ZSWIM4	19	13915630										0				0			0	0		0		No-ASE
C19orf57	19	13993619										0	0			0			0	0		0	0	No-ASE
CC2D1A	19	14023342			0				0			0			0	0				0			0	No-ASE
RFX1	19	14074441	0	0	0			0				0		0	0									No-ASE
IL27RA	19	14153272				0		0	0	0		0								0		0		No-ASE
PALM3	19	14164629				0							0		0								1	No-ASE
PKN1	19	14551107			0	0			0	0			0	0		0			0					No-ASE
GIPC1	19	14589378							0					0					0					No-ASE
ZNF333	19	14805849		0	0	0				0				0	0							0	0	No-ASE
EMR2	19	14854232		0				0				0		0	1					0		0	0	No-ASE
OR7C1	19	14910199		1											1									ASE
OR7A10	19	14951762																	1					ASE
OR7A17	19	14991491				0																		No-ASE
OR1I1	19	15197898	0				0	1																Heterogeneous_ASE
ILVBL	19	15226199				0			0															No-ASE
NOTCH3	19	15271630			0	0		0						0	0	0						1	0	No-ASE
BRD4	19	15349554		0																				No-ASE
PGLYRP2	19	15579491	1		1	1		1					1	1		1								ASE
CYP4F8	19	15726546		1						0				0	0					0		1		Heterogeneous_ASE
CYP4F12	19	15784370	0	0	0	0							0		0	0			0				0	No-ASE
OR10H3	19	15852223		0	0	0									1	1								Heterogeneous_ASE
OR10H1	19	15918349											0		0	0			1					No-ASE
CYP4F2	19	15990223	1	0		0		1	0			1								0		1	0	Heterogeneous_ASE
CYP4F11	19	16024583	1	1	0	0			0	1		1	1	0		0				0			0	Heterogeneous_ASE
MED26	19	16686864								0														No-ASE
NWD1	19	16831060	0	0	0	0		0	0	0		0	0	0	0	1			0	0		0	1	No-ASE
SIN3B	19	16942368																		0				No-ASE
F2RL3	19	17000414		1		1							1						1					ASE
CPAMD8	19	17003789				0				0														No-ASE
HAUS8	19	17160720			0								0			0				0		0	0	No-ASE
MYO9B	19	17212570	0	0	0	0		0	0			0	0		0				0				0	No-ASE
OCEL1	19	17337066	0						0											0				No-ASE
BABAM1	19	17382463		0																				No-ASE
ANKLE1	19	17392894		0		0		0		0		1		0	0							0	1	No-ASE
ABHD8	19	17403508	0	0		0																	0	No-ASE
GTPBP3	19	17445855							0			0		0	0				0			0		No-ASE
MVB12A	19	17531171								0														No-ASE
FAM129C	19	17638121										0		0						1		0	1	Heterogeneous_ASE
UNC13A	19	17731515		0																				No-ASE
MAP1S	19	17831774										0				0			0	0				No-ASE
FCHO1	19	17873644										0												No-ASE
B3GNT3	19	17918650						0				0			0									No-ASE
JAK3	19	17937659		0																				No-ASE
ARRDC2	19	18112361		0		0			0	0				0	0							0	0	No-ASE
IL12RB1	19	18170727		0	1				1						0		1			1			1	Heterogeneous_ASE
MAST3	19	18232582	0	0	0	0			0						0				0	0			0	No-ASE
PIK3R2	19	18266699	0							0										0			0	No-ASE
IFI30	19	18285869	0	0	0	0			0			0			0								0	No-ASE
MPV17L2	19	18304123		0	0	0			0						0									No-ASE
RAB3A	19	18308430								0			0											No-ASE
PDE4C	19	18321797						1												1				ASE
KIAA1683	19	18367995	0		0	0		0	0	0		0			0	0			0	0			0	No-ASE
PGPEP1	19	18468332																				0		No-ASE
GDF15	19	18497141										0	0							0		0		No-ASE
ELL	19	18557180		0																				No-ASE
KXD1	19	18672894	0		0			0								0						0		No-ASE
CRTC1	19	18864361										0			0					0		0		No-ASE
UPF1	19	18976484			0																			No-ASE
COPE	19	19014109			0	0							0											No-ASE
SUGP2	19	19106080	0	0					0			0	0	0	0	0			0			0	0	No-ASE
TMEM161A	19	19230816										0												No-ASE
RFXANK	19	19304850							0				0											No-ASE
SUGP1	19	19407915	0	0	0			0																No-ASE
GATAD2A	19	19576204												0										No-ASE
LPAR2	19	19735174			0																			No-ASE
ATP13A1	19	19756268																				0		No-ASE
ZNF101	19	19789579			0																			No-ASE
ZNF14	19	19822451			0	0										0								No-ASE
ZNF682	19	20116906		1								0												Heterogeneous_ASE
ZNF85	19	21117803								1			1										0	Heterogeneous_ASE
ZNF714	19	21300054			1	1				1		0										0		Heterogeneous_ASE
ZNF431	19	21326358				0				0												0		No-ASE
ZNF708	19	21477325		0	0	0		0				0	0		0	0			0			0		No-ASE
ZNF493	19	21588604																					0	No-ASE
ZNF429	19	21713459			0							0										0		No-ASE
ZNF91	19	23543107																	0					No-ASE
ZNF681	19	23927522		0								0												No-ASE
ZNF254	19	24270119				0		0				0	0											No-ASE
UQCRFS1	19	29704010																		0		0		No-ASE
POP4	19	30097231		0																				No-ASE
C19orf12	19	30193632						0																No-ASE
URI1	19	30496559																		0				No-ASE
TSHZ3	19	31767465				0																		No-ASE
ANKRD27	19	33089155	0	0	0	0								0	0							0	0	No-ASE
SLC7A9	19	33321545		0		0				1		1	1	0						0				Heterogeneous_ASE
CEP89	19	33370070				0		0	0	0		0	0	0	0	0				0		0		No-ASE
C19orf40	19	33464181													0							0		No-ASE
GPATCH1	19	33579109						0		0				0						0		0	0	No-ASE
WDR88	19	33628589			0										0							0	0	No-ASE
LRP3	19	33693755						0												1				Heterogeneous_ASE
PEPD	19	33878269				0				0		0												No-ASE
KIAA0355	19	34791476																		1				ASE
PDCD2L	19	34895647		0																				No-ASE
ZNF302	19	35173793				0				1				0										Heterogeneous_ASE
ZNF181	19	35225951				0										0								No-ASE
ZNF599	19	35250046		0					0															No-ASE
ZNF30	19	35422771	0	0	0	0			0	0				0	0	0						0		No-ASE
ZNF792	19	35448919	0	0		0			0	0				0	0	0						0		No-ASE
GRAMD1A	19	35500343		0	0	0				0			0	0		1				0		0		No-ASE
SCN1B	19	35524824												0										No-ASE
FXYD5	19	35648330	0	0	0			0	0				0	0	0	0				0				No-ASE
FAM187B	19	35715930		0	0			0		0			0	0	0							1		No-ASE

LSR	19	35739852		0		0														0				No-ASE
CD22	19	35826971	0	0												0							1	No-ASE
FFAR2	19	35940752								0														No-ASE
DMKN	19	35989621		0					1	0		0								0			1	Heterogeneous_ASE
SBSN	19	36015624	0	0										0	0									No-ASE
HAUS5	19	36104777								0		0	0	0								0		No-ASE
ETV2	19	36133528										1												ASE
UPK1A	19	36157725				0				0		0	0			0				0		0	0	No-ASE
ZBTB32	19	36206050	0							0						0								No-ASE
MLL4	19	36210703		0		0			0			0	0			0				0		0		No-ASE
IGFLR1	19	36230240	0						0	0						0								No-ASE
C19orf55	19	36250780		0						0			0									0		No-ASE
NFKBID	19	36387656										0								0				No-ASE
TYROBP	19	36398158										1												ASE
THAP8	19	36526362			0	0														0			0	No-ASE
WDR62	19	36545883	0		0				0	0		0		0	0					0				No-ASE
ZNF565	19	36674305	0							0		0	0		0	0							0	No-ASE
ZNF566	19	36940510		0																				No-ASE
ZNF529	19	37038010										1		0										Heterogeneous_ASE
ZNF382	19	37100817		0																				No-ASE
ZNF461	19	37129806		0						0														No-ASE
ZNF568	19	37428070		0																				No-ASE
ZNF585B	19	37676134	0		1	0			1			1	0			0				1		0	0	Heterogeneous_ASE
HKR1	19	37835658		0	0	0				0		0				0				0		0	0	No-ASE
ZNF527	19	37870148		0						0						0						0	0	No-ASE
LOC100507433	19	38055534												0										No-ASE
ZNF607	19	38188941		0	0	0				0					1	0						0	0	No-ASE
ZNF573	19	38229926						0						0										No-ASE
WDR87	19	38375666		1																		0		Heterogeneous_ASE
SIPA1L3	19	38572304			0	0																		No-ASE
SPINT2	19	38774311				0														0		0	0	No-ASE
YIF1B	19	38795554											0			0						0		No-ASE
KCNK6	19	38817264											0			0								No-ASE
CATSPERG	19	38827960											0			0								No-ASE
GGN	19	38875159																				0	1	Heterogeneous_ASE
FAM98C	19	38894307																					0	No-ASE
RASGRP4	19	38900728				0																	1	Heterogeneous_ASE
RYR1	19	38931418				0																		No-ASE
CAPN12	19	39221785	0																			0	0	No-ASE
ECH1	19	39306529	0		0	0		0	0	0		0	0		0	0							0	No-ASE
RINL	19	39361302		0						0		0		0									0	No-ASE
SIRT2	19	39369369			0			0		0				0	0	0						0	0	No-ASE
SARS2	19	39406322																		0				No-ASE
IFNL1	19	39787143		0				0																No-ASE
LRFN1	19	39804773										0												No-ASE
PLEKHG2	19	39905650	0		0	0		0		0		0	0	0	0	0						0	0	No-ASE
RPS16	19	39923978						0				0								0		0	0	No-ASE
EID2	19	40030018	0			0				0						0				0			0	No-ASE
CLC	19	40224952											1											ASE
FCGBP	19	40354448						0				0								0				No-ASE
CNTD2	19	40729096						0																No-ASE
HIPK4	19	40885586						0						0										No-ASE
PRX	19	40899893		0					0			0	1	0	0					0		0		No-ASE
SERTAD1	19	40929204	0							0			0	0		0						0		No-ASE
SERTAD3	19	40947076	0	0								0		0	0	0								No-ASE
SPTBN4	19	40978640	0						0	0		0		0		0				0		0		No-ASE
LTBP4	19	41105395	0	0	0	0		0		0		0	0	0	0							0		No-ASE
ADCK4	19	41198211		0				0	0			0				0						0	0	No-ASE
RAB4B-EGLN2	19	41306568								0														No-ASE
CYP2A7	19	41382476																		0				No-ASE
CYP2F1	19	41622108	0						1	1			0	0									1	Heterogeneous_ASE
AXL	19	41727077													0									No-ASE
B9D2	19	41860909				0		0	0	0				0	0					0		0		No-ASE
EXOSC5	19	41892573	0			0		0		0			0	0	0	0				0		0		No-ASE
BCKDHA	19	41916549												0									0	No-ASE
ATP5SL	19	41938222	0			0		0		0			0	0	0	0				0		0		No-ASE
CEACAM21	19	42083552	0			1								0	0						1	0		Heterogeneous_ASE
CEACAM5	19	42213944						0	0					0						0		0		No-ASE
ZNF526	19	42728635				0				0				0		0								No-ASE
LOC100996307	19	42906838	0												0							0		No-ASE
PSG8	19	43262353							0						0							1		Heterogeneous_ASE
PSG6	19	43406575										1												ASE
PSG11	19	43519270	0													0				0			0	No-ASE
PSG2	19	43575905							0											1			1	Heterogeneous_ASE
CD177	19	43857873			0															1				Heterogeneous_ASE
PHLDB3	19	43979629										0	0									0		No-ASE
XRCC1	19	44047554	0			0				0				0								0		No-ASE
IRGQ	19	44090195	0		0	0				0		0	0		0	0						0	0	No-ASE
ZNF428	19	44116609	0		0				0	0			0		0	0						0	1	No-ASE
CADM4	19	44128304										0												No-ASE
PLAUR	19	44153044			0					0					0	0				0				No-ASE
SMG9	19	44237580						0																No-ASE
LYPD5	19	44302609	0			0			0				1		0	0				0			0	No-ASE
ZNF283	19	44351276	0					0	0	0		0	0	0	0					0				No-ASE
ZNF404	19	44376789							1						0									Heterogeneous_ASE
ZNF45	19	44417544				0		0	0	0			0	0	0	0				0				No-ASE
ZNF221	19	44469387	0							0					0								0	No-ASE
ZNF230	19	44513250																				0	0	No-ASE
ZNF222	19	44530227				1		0	0			0			0					0		0	0	No-ASE
ZNF223	19	44564980				1		0	0			1			0					0		0	0	No-ASE
ZNF224	19	44610665	0			0		0				0				0				0				No-ASE
ZNF225	19	44619988																				0		No-ASE
ZNF234	19	44652954	0	0				0					0		0					0		0		No-ASE
ZNF226	19	44669960								0			0	0	0	0				0		0	0	No-ASE
ZNF233	19	44777053							0				0	0		0				0			0	No-ASE
ZNF235	19	44791489							0				0	0		0				0		0	0	No-ASE
ZFP112	19	44831658							0				0	0						1		0	0	No-ASE
ZNF285	19	44890727							0			1	1	1									1	ASE
ZNF229	19	44932521													1							1		ASE
ZNF180	19	44980752		1	0	0			0			0	0		0							0	0	No-ASE
CEACAM20	19	45015106	0			0			1			1				1				1		0	1	Heterogeneous_ASE
PVR	19	45150522	0			0							0		0					0		0		No-ASE
CEACAM19	19	45175950			0																			No-ASE
CBLC	19	45281375											0		0					0		0		No-ASE
BCAM	19	45314515	0					0					0	0	0								0	No-ASE
PVRL2	19	45368740			0				0	0			0		0					0				No-ASE
APOC4-APOC2	19	45445541						0				1												Heterogeneous_ASE
RELB	19	45506208																					0	No-ASE
BLOC1S3	19	45682571				0																		No-ASE
CKM	19	45810035						0		0			1	0									1	Heterogeneous_ASE
ERCC2	19	45854910	0			0				0		0	0									0	0	No-ASE
CD3EAP	19	45910365				0														0			0	No-ASE
ERCC1	19	45916813	0		0	0		0				0		0								0	0	No-ASE
PPM1N	19	46002007								1														ASE
EML2	19	46119772	0			0						0				0				0		0	0	No-ASE
GIPR	19	46173929	0						0			0	0	0	0					0		0		No-ASE
FBXO46	19	46216104				0						0												No-ASE
DMPK	19	46273846	0									0												No-ASE

SYMPK	19	46318864								0													0	No-ASE
MYPOP	19	46393885	0						0			0								0			0	No-ASE
CCDC61	19	46506423								0			1							0				Heterogeneous_ASE
IGFL4	19	46543422											0							0				No-ASE
HIF3A	19	46811488	0			0																	0	No-ASE
CCDC8	19	46914459																		1				ASE
SLC1A5	19	47278771			0								0											No-ASE
ZC3H4	19	47569003	0			0		0	0	0		0		0						0		0	0	No-ASE
DHX34	19	47856324								0														No-ASE
GLTSCR1	19	48182946			0			0	0			0	0	0		0								No-ASE
ELSPBP1	19	48511909							1															ASE
PLA2G4C	19	48551619						0	0			0												No-ASE
LIG1	19	48618941							0				0											No-ASE
CARD8	19	48715032						0		0		0	0							0				No-ASE
CCDC114	19	48800301				0		0																No-ASE
EMP3	19	48830797								0														No-ASE
TMEM143	19	48845855								0														No-ASE
SYNGR4	19	48869139			1	1		1	1													1		ASE
FAM83E	19	49104466						0	0			0								0				No-ASE
SEC1P	19	49164944																				0		No-ASE
FUT2	19	49206286			0				0	0		0	0		0	0				0				No-ASE
IZUMO1	19	49244220			1			0		0		0				1				1			0	Heterogeneous_ASE
FUT1	19	49253517														0								No-ASE
BCAT2	19	49298715												0										No-ASE
HSD17B14	19	49316458	1			0			1											0				Heterogeneous_ASE
PLEKHA4	19	49340746	1		0			0	1													0		Heterogeneous_ASE
PPP1R15A	19	49376582	0		0			0	0			0								0		0	0	No-ASE
TULP2	19	49384272	0					1												0				Heterogeneous_ASE
DHDH	19	49436980				0				0		0			0					0			0	No-ASE
C19orf73	19	49621964	0					0															0	No-ASE
HRC	19	49654561			0			0				1			0					0		0		No-ASE
TRPM4	19	49671207																		0				No-ASE
DKKL1	19	49867899				0		0		0		0	0			0								No-ASE
PIH1D1	19	49949652	0		0			0				0	0			0				0		0		No-ASE
ALDH16A1	19	49956613	0		0			0				0		0	0	0						0		No-ASE
RPL13A	19	49993519																				0		No-ASE
RCN3	19	50031894				0				0		0												No-ASE
NOSIP	19	50059652												0										No-ASE
PRRG2	19	50086540				0			0	0				0		0								No-ASE
PRR12	19	50097746						0				0												No-ASE
SCAF1	19	50148336														0				0		0		No-ASE
IRF3	19	50162953												0	0									No-ASE
CPT1C	19	50195648													0							0		No-ASE
PTOV1	19	50360233										0		0										No-ASE
TBC1D17	19	50383591	0		0					0		0	0			0						0	0	No-ASE
IL4I1	19	50392966			0			0		0					0					0				No-ASE
ATF5	19	50435753						0												0				No-ASE
VRK3	19	50479999				0			0	0			0											No-ASE
ZNF473	19	50542478	0																					No-ASE
MYH14	19	50714016						0																No-ASE
NAPSA	19	50861884				0		0														0		No-ASE
POLD1	19	50902164	0					0					0											No-ASE
MYBPC2	19	50938418				1								0								1		Heterogeneous_ASE
EMC10	19	50983413												0										No-ASE
ASPDH	19	51015030				0														0				No-ASE
ACPT	19	51293678			0					0		0										0	0	No-ASE
C19orf48	19	51301395			0					0		0										0	0	No-ASE
KLK1	19	51322484			0				0	0		0	0	0	0	0				0		0	0	No-ASE
KLK3	19	51359497			0							1	0							0				No-ASE
KLK2	19	51378022										0	0							1				Heterogeneous_ASE
KLK7	19	51480829			1				0							0				0			0	No-ASE
KLK10	19	51517731	0		0	0			0	0			0	0								0	0	No-ASE
KLK11	19	51525890			0															1		0		Heterogeneous_ASE
KLK14	19	51581368	0		0	0						0	0							0				No-ASE
CTU1	19	51607507						0		0					0					0				No-ASE
CD33	19	51728567																		0			1	Heterogeneous_ASE
VSIG10L	19	51837487			0	0		0				0	0	0										No-ASE
ETFB	19	51848546	0			0		0	0				0	0		0								No-ASE
CEACAM18	19	51981792	0		1			0						0								0		No-ASE
SIGLEC12	19	51994897				1				1				1								1	1	ASE
SIGLEC6	19	52023419							1													1		ASE
ZNF175	19	52076628			1	0																		Heterogeneous_ASE
HAS1	19	52216563												0										No-ASE
FPR1	19	52249211	1		0	0		0						0		0				0		1	0	No-ASE
ZNF577	19	52375785	0					1	0			0		0	0								0	No-ASE
ZNF350	19	52468109							0						0	0								No-ASE
ZNF615	19	52496149			0							0			0	0								No-ASE
ZNF614	19	52519099	0		0				0			0			0	0								No-ASE
ZNF432	19	52537004			0							0		0										No-ASE
ZNF841	19	52568422			0							0		0										No-ASE
ZNF616	19	52618124		0		0																		No-ASE
ZNF836	19	52658231										0	0											No-ASE
ZNF766	19	52772880								0														No-ASE
ZNF480	19	52819102			1	0														0			0	No-ASE
ZNF610	19	52852469				0			1														1	Heterogeneous_ASE
ZNF880	19	52877677	1	1					0					0		1								Heterogeneous_ASE
ZNF528	19	52909795												0										No-ASE
ZNF701	19	53085635	0										0	0									0	No-ASE
ZNF83	19	53116331				0						0		1		0				0		1		Heterogeneous_ASE
ZNF468	19	53344556								0		0		1						0			0	No-ASE
ZNF816-ZNF321P	19	53453091		0	0	0			0	0			0		0							0	0	No-ASE
ZNF160	19	53571679			0	0						0		1		0						0		No-ASE
ZNF415	19	53611719			0				0													0		No-ASE
ZNF347	19	53644456	1																				1	ASE
ZNF665	19	53667726	0	1						1		0	1	1	0	0				1			0	Heterogeneous_ASE
BIRC8	19	53792954																				1		ASE
ZNF525	19	53884412								1		0										1	0	Heterogeneous_ASE
ZNF761	19	53958104	0	1				0		0			0	0										No-ASE
VSTM1	19	54544231																				0		No-ASE
LENG1	19	54659488							0															No-ASE
TMC4	19	54664209	0		0	0		0		0		0			0	0						0		No-ASE
TTYH1	19	54930392				0																		No-ASE
LENG8	19	54962467	0		0				0				0	0	0								0	No-ASE
LENG9	19	54973280	0		0	0		0	0			0	0	0	0								0	No-ASE
LILRB1	19	55142951										1												ASE
NCR1	19	55418054				0									0									No-ASE
NLRP7	19	55441871								1		1											1	ASE
NLRP2	19	55481394				0			1	1		1											1	ASE
GP6	19	55525508			0			0		0		0	0			0						0		No-ASE
RDH13	19	55556527								0				0		0							0	No-ASE
EPS8L1	19	55587822	0		0				0			0	0	0		0				0		0	0	No-ASE
TNNI3	19	55663250								0												1		Heterogeneous_ASE
DNAAF3	19	55670467				0														1			0	Heterogeneous_ASE
PTPRH	19	55693244	0		0	0		0	0	0		0	0	0	0	0				0			0	No-ASE
TMEM86B	19	55738568			0	0						0	0									0		No-ASE
PPP6R1	19	55742176												0										No-ASE
TMEM150B	19	55824328	0		0			1	0	0		0		0	0								0	No-ASE
FAM71E2	19	55869601							0															No-ASE

IL11	19	55879672				0								0										No-ASE
RPL28	19	55897956							0				0											No-ASE
NAT14	19	55998262			0	0				0		0		0		0						0	0	No-ASE
SSC5D	19	56001026			0																			No-ASE
CCDC106	19	56160641	0																					No-ASE
EPN1	19	56188968				0																		No-ASE
NLRP4	19	56369040										1		0										Heterogeneous_ASE
NLRP13	19	56407347	0																					No-ASE
NLRP5	19	56515090							1						1									ASE
ZSCAN5B	19	56701233			1			1		1		1	1									1		ASE
ZSCAN5A	19	56732947	0		0	0			0														0	No-ASE
ZFP28	19	57050521				0			1			1				0							0	Heterogeneous_ASE
ZNF470	19	57085769			1	0			0			0			0	1				1			0	Heterogeneous_ASE
ZNF71	19	57132677						0				0												No-ASE
SMIM17	19	57158953								0														No-ASE
ZNF264	19	57705243			1	0		0	0	0			0	0	0					1				No-ASE
ZNF805	19	57760018			0	0		0	0	0			0	0	0					0			0	No-ASE
ZNF543	19	57838018	0			0		0	0	0		0	0	1						1		0		No-ASE
ZNF304	19	57863052											1											ASE
ZNF548	19	57905559										0												No-ASE
ZNF17	19	57929280	0			0				0				0						0				No-ASE
ZNF749	19	57953262	0		0			0		0			0		0	0						0	0	No-ASE
VN1R1	19	57966926							0				0			0				0			1	No-ASE
ZNF772	19	57984968	1		0			0		0		1	1		0					0				Heterogeneous_ASE
ZNF419	19	58004398														0								No-ASE
ZNF773	19	58011461	1									1	1							1				ASE
ZNF550	19	58058351				0						0	0		0	0				1				No-ASE
ZNF530	19	58115733							0				0		0					1				No-ASE
ZNF134	19	58131576	1										1									1		ASE
ZNF211	19	58144740	0	0	0	0		0		1		0	0	0	0	0						0		No-ASE
ZNF551	19	58196753												0										No-ASE
ZNF154	19	58213007								0		0								0				No-ASE
ZNF671	19	58231890												0										No-ASE
ZNF135	19	58571371							1															ASE
ZNF274	19	58718140			0							1	1	1								1	0	Heterogeneous_ASE
ZNF544	19	58755399			1	1		0	0								0						0	Heterogeneous_ASE
ZNF8	19	58797098				0														0				No-ASE
ZSCAN22	19	58846211				0																		No-ASE
ZNF497	19	58867567	0			0				0					0	0				0		0	0	No-ASE
ZNF837	19	58879118			0																			No-ASE
ZNF584	19	58921310	0					0					0	0	0							0		No-ASE
ZNF132	19	58944778	0		0			0					0	0	0	0				0		0	0	No-ASE
ZNF324	19	58980631																					0	No-ASE
ZNF446	19	58989014	0			0		0					0	0								0		No-ASE
SLC27A5	19	59009949												0										No-ASE
TRIM28	19	59056881											0											No-ASE
LOC100131691	19	59073495	0		0	0		0				0	0	0	0					0		0		No-ASE
DEFB129	20	209937																0						No-ASE
DEFB132	20	238421																0						No-ASE
C20orf96	20	251883						0			0		0		0		0	0		0			0	No-ASE
NRSN2	20	330408							0															No-ASE
TRIB3	20	368739								0	0			0									1	No-ASE
TBC1D20	20	419483																	0					No-ASE
SLC52A3	20	741699				0		0			0				0									No-ASE
FAM110A	20	825607		0																				No-ASE
PSMF1	20	1099523		0			0					0		0	0			0					0	No-ASE
TMEM74B	20	1161558																					0	No-ASE
RAD21L1	20	1210647					0							1										Heterogeneous_ASE
FKBP1A-SDCBP2	20	1292989		0		0		0	0	0		0		0			0	0		0				No-ASE
NSFL1C	20	1424531						0	0		0	0			0				0					No-ASE
SIRPB2	20	1456897																0						No-ASE
SIRPD	20	1515086					0		1									0						Heterogeneous_ASE
SIRPB1	20	1546802					0											0						No-ASE
SIRPG	20	1615934		0							0							0						No-ASE
SIRPA	20	1896100		0			0	1			1			0	0								0	Heterogeneous_ASE
TGM3	20	2290333		0											0				0					No-ASE
TGM6	20	2375142																0						No-ASE
ZNF343	20	2464066			0																			No-ASE
TMC2	20	2517923				0					0		0		0			0		0			0	No-ASE
NOP56	20	2635212		0																				No-ASE
PCED1A	20	2816191		0																				No-ASE
PTPRA	20	2945759							0					0			0		0					No-ASE
GNRH2	20	3025077		0		0	0		0	0	0			1				0						No-ASE
UBOX5-AS1	20	3096055																	0					No-ASE
DDRGK1	20	3171337		0		0		0	0	0				0										No-ASE
ITPA	20	3193842				0						0												No-ASE
SLC4A11	20	3209011		0		0		0			0		0	0				0						No-ASE
C20orf194	20	3234459							0					0	0									No-ASE
ATRN	20	3452041				0																		No-ASE
ADAM33	20	3649619																0						No-ASE
SIGLEC1	20	3670249																0						No-ASE
HSPA12B	20	3721510						0																No-ASE
CDC25B	20	3777365		0									0											No-ASE
MAVS	20	3838355		0			0	0	0				0	0	0				0	0				No-ASE
PANK2	20	3869878											0		0									No-ASE
PRND	20	4705274					0											0						No-ASE
SLC23A2	20	4835338		0					0				0						0	0				No-ASE
C20orf196	20	5753579		0		0				0			0				0		0	0				No-ASE
CHGB	20	5896996		0		0		0	1	1	0		1				0	0		0				Heterogeneous_ASE
TRMT6	20	5923204							0	0									0					No-ASE
MCM8	20	5932770				0			0	0									0					No-ASE
CRLS1	20	6011934		0											0			0						No-ASE
FERMT1	20	6057860		0						0								0		0				No-ASE
BMP2	20	6750882												0										No-ASE
TMX4	20	7962707					0			0			0					0	0	0			0	No-ASE
PLCB1	20	8113326		1		0	0		1			1			0			0		1	0			Heterogeneous_ASE
PLCB4	20	9288522						0						0	0				0					No-ASE
PAK7	20	9520135									1	1	1	1										ASE
SNAP25-AS1	20	10018954						0	0	0	0		0	0	0		0	0		0			0	No-ASE
MKKS	20	10385919						0			0	0			0		0	0					0	No-ASE
SLX4IP	20	10536882												0			0	0			1			No-ASE
SPTLC3	20	12989981							0					0										No-ASE
ESF1	20	13695607		0		0									0				0					No-ASE
MACROD2	20	14066276		0		0		0	0	0	0	0	0		0		0						0	No-ASE
KIF16B	20	16293011					0					0	0	0					0	0				No-ASE
BFSP1	20	17474749		0																				No-ASE
RRBP1	20	17595399					0	0	0	0	0	0		1	0		0	0	0	0			0	No-ASE
MGME1	20	17950545												0					0	0				No-ASE
CSRP2BP	20	18139832						0																No-ASE
ZNF133	20	18295730							0		0			0	0									No-ASE
DZANK1	20	18365673							0			0						0						No-ASE
SEC23B	20	18491519				0	0		0				0				0		0	0				No-ASE
SLC24A3	20	19261606					0	1				0			0									No-ASE
RIN2	20	19867343		0			0		0	0	0		0	0	0		1	0	0	0				No-ASE
NAA20	20	20006368												0										No-ASE
CRNKL1	20	20016856					0	0		0	0	0							0	0				No-ASE
C20orf26	20	20037320							0		0	0					0	0	0				0	No-ASE
RALGAPA2	20	20453542											0	0					0					No-ASE

PLK1S1	20	21117104		0		0			0	0		0						0	0	0			0	No-ASE
FOXA2	20	22562543								0			0					0						No-ASE
SSTR4	20	23016706																0	1					Heterogeneous_ASE
THBD	20	23028631							1															ASE
GZF1	20	23345243		0		0		0		0	0		0	0	0			0		0				No-ASE
CSTL1	20	23421059		0			0		0	0	0		0	1	0			0					0	No-ASE
CST9L	20	23545658		1														0						Heterogeneous_ASE
CST9	20	23584259			0			0					1	0				0						No-ASE
CST1	20	23731406		0	0		0		0	1					0			0		0				No-ASE
CST5	20	23856833													0			0						No-ASE
APMAP	20	24944454				0				0	0			0				0						No-ASE
VSX1	20	25056940				1														0				Heterogeneous_ASE
ENTPD6	20	25187213		0		0	0		0	0	0				0		0			0			0	No-ASE
PYGB	20	25228902		0						0	0		0					0		0			0	No-ASE
ABHD12	20	25282864				0			0															No-ASE
GINS1	20	25394454				0			0	0			0				0	0						No-ASE
NINL	20	25434139				0	0	0	1	0					0		1	0						No-ASE
ZNF337	20	25655765													0									No-ASE
DEFB119	20	29965123															1							ASE
ID1	20	30193201																0						No-ASE
COX4I2	20	30231212	0	0			0							0				0		0			0	No-ASE
TTLL9	20	30458965										0		0										No-ASE
TM9SF4	20	30729301													0			0						No-ASE
POFUT1	20	30797976										0												No-ASE
KIF3B	20	30898259																	0					No-ASE
ASXL1	20	31019199			0	0		0					0				0		0				0	No-ASE
BPIFB2	20	31596430					0																	No-ASE
BPIFB6	20	31619500				0						0						0						No-ASE
BPIFB3	20	31644401										1						0						Heterogeneous_ASE
BPIFB4	20	31671181						0	0	0		0			0	0	0	0			0			No-ASE
BPIFA3	20	31805408					1											0						Heterogeneous_ASE
BPIFB1	20	31873994																0						No-ASE
CBFA2T2	20	32162037	0																					No-ASE
NECAB3	20	32245789				0													0					No-ASE
PXMP4	20	32295525	0	0	0	1			0		0	0	0	0		0		0		0				No-ASE
ZNF341	20	32328797				0																		No-ASE
RALY	20	32659935	0																					No-ASE
PIGU	20	33148734				0					0													No-ASE
ACSS2	20	33470659			0	0	0			0				0	0	0	0	0	0	0				No-ASE
MYH7B	20	33565466	0	0	0	0						0			0			0	0		0		1	No-ASE
EDEM2	20	33703317	0												0			0			0			No-ASE
PROCR	20	33762696	0		0				0			0	0											No-ASE
UQCC	20	33891824		0	0	0								0	0	0	0	0	0		0			No-ASE
GDF5	20	34022004																0						No-ASE
CEP250	20	34050241	0			0																		No-ASE
C20orf173	20	34116282	0	0					0		0	0												No-ASE
CPNE1	20	34214173	0	1		0					0	0	0	0	0	0							0	No-ASE
NFS1	20	34257560																					0	No-ASE
PHF20	20	34446298		0		0					0		0	0		0								No-ASE
CNBD2	20	34556714		0		0					0			0	1	0								No-ASE
LINC00657	20	34635441		0		0					0		0	0										No-ASE
AAR2	20	34827932																	0					No-ASE
DSN1	20	35381262																	0					No-ASE
SOGA1	20	35414898						0											0					No-ASE
C20orf118	20	35506344			0								0											No-ASE
SAMHD1	20	35526858																			0			No-ASE
RBL1	20	35632140											0											No-ASE
MROH8	20	35731181	0	0	0	0	0				0		1	0	0				0	0	0			No-ASE
RPN2	20	35812669	0		0	0			0		0		0		0		0							No-ASE
TTI1	20	36624780						0																No-ASE
KIAA1755	20	36841536			1		0					1				0		0						Heterogeneous_ASE
BPI	20	36932627									1				0			0						Heterogeneous_ASE
ARHGAP40	20	37252018				1																		ASE
ACTR5	20	37378721	0	0			0	0	0	0	0	0	0	0	0	0		0	0					No-ASE
DHX35	20	37597822				0		0		0	0				0					0			0	No-ASE
PLCG1	20	39791176	0	0					0	0	0	0	0		0		0			0	0			No-ASE
ZHX3	20	39831021	0	0								0	0			0					0			No-ASE
LPIN3	20	39974453					0			0	0	0	0	0				0			0			No-ASE
EMILIN3	20	39990159										0						0						No-ASE
CHD6	20	40033295				0						0				0					0			No-ASE
L3MBTL1	20	42142225																	1					ASE
IFT52	20	42223350									0										0			No-ASE
MYBL2	20	42310449						0		0	0						0		0		0			No-ASE
GTSF1L	20	42355002			1																			ASE
C20orf111	20	42826132		0	0	0	0	0				0							0	0	0			No-ASE
LOC100505783	20	42854134										0	0											No-ASE
GDAP1L1	20	42885803																					1	ASE
R3HDML	20	42965811			0		0								0		0	0			0			No-ASE
HNF4A	20	43042364	0													0								No-ASE
ADA	20	43249032				0	0	0		0		0	0	0			0		0	0				No-ASE
KCNK15	20	43374694	0		0						0		0	0							0		0	No-ASE
PABPC1L	20	43541307	0		0			0	0	0	0		0	0	0	0		0						No-ASE
STK4	20	43600730	0	0			0				0	0							0	0	0			No-ASE
KCNS1	20	43723569	0		0	0			0			0		0	0	0		0	0					No-ASE
WFDC5	20	43739100	0			0									0	0								No-ASE
PI3	20	43803606	0		0	0		0		0	0	0			0	0					0			No-ASE
SEMG1	20	43836015	0					0		1						0					0			No-ASE
MATN4	20	43922428				0			1					1			1		1		1		1	ASE
SDC4	20	43956017	0				0	0						0	0		0	0			0		0	No-ASE
SYS1-DBNDD2	20	44005936		0	0	0				0	0		0			0		0			0		0	No-ASE
SPINT3	20	44141331																0						No-ASE
EPPIN-WFDC6	20	44171347																	1					ASE
WFDC8	20	44180670																0	1					Heterogeneous_ASE
WFDC9	20	44237357	0															0						No-ASE
SPINT4	20	44351095	0							0					0			0	0					No-ASE
WFDC3	20	44404084	0					0		0		0	0	0	0			0			0		0	No-ASE
SNX21	20	44462565											0											No-ASE
ZSWIM3	20	44505437	0	0					0			0		0		0			0		0		0	No-ASE
ZSWIM1	20	44511254							0															No-ASE
PCIF1	20	44569202	0	0				0		0				0	0								0	No-ASE
ZNF335	20	44577623											0			0	0			0				No-ASE
MMP9	20	44637624					0											0						No-ASE
TP53RK	20	45315721			0				0		0		0		0	0	0							No-ASE
SLC2A10	20	45353992											1						0					Heterogeneous_ASE
ZMYND8	20	45839488				0				0									0				0	No-ASE
NCOA3	20	46254213			0			0	0						0	0							0	No-ASE
SULF2	20	46290517		0									0					0			0		0	No-ASE
PREX1	20	47244472					0							0		0	0		0				0	No-ASE
ARFGEF2	20	47558417						0																No-ASE
CSE1L	20	47679880						0				0	0		0				0		0			No-ASE
DDX27	20	47838020						0	0		0		0	0	0	0	0		0	0	0			No-ASE
ZNFX1	20	47863958					0	0	0	0	0	0			0	0	0			0	0			No-ASE
B4GALT5	20	48252914		0								0												No-ASE
SLC9A8	20	48429485		0			0																	No-ASE
SNAI1	20	48600631			0			0								0								No-ASE
PTPN1	20	49127119			0			0																No-ASE
FAM65C	20	49203789	0		0			0	0		0				0	0			1		0		0	No-ASE
MOCS3	20	49575473										0												No-ASE

SALL4	20	50400863	0								0		0				1							No-ASE
ZFP64	20	50701128		0		0		0	0				0	0										No-ASE
TSHZ2	20	51870106	0	0	0																		1	No-ASE
ZNF217	20	52188363								0	0		0	0	0			0			0			No-ASE
BCAS1	20	52561469	0			0		0					0	0	0	0		0	0	0	0		1	No-ASE
CYP24A1	20	52771261	0	1	0	0							1	0	0					0	0		0	No-ASE
MC3R	20	54823993									0			0	0			0						No-ASE
FAM210B	20	54941140								0								0			0			No-ASE
AURKA	20	54945542	0	0		0	0		0			0												No-ASE
CASS4	20	54987530												0										No-ASE
RTFDC1	20	55048429		0								0											0	No-ASE
FAM209B	20	55108510			1	1	0						1	0	1			0	1		1		0	Heterogeneous_ASE
BMP7	20	55750060							0															No-ASE
CTCFL	20	56073644					0																	No-ASE
PCK1	20	56136474				0	0	0	0			0			0	0	0							No-ASE
ZBP1	20	56179808														0				0				No-ASE
APCDD1L	20	57035940		0	0				0															No-ASE
STX16-NPEPL1	20	57268803	0	0			0		0		0	0		0		0	0			0	0		0	No-ASE
NELFCD	20	57561221												0					0				1	Heterogeneous_ASE
TUBB1	20	57594590							0		0			0				0	0				1	No-ASE
ZNF831	20	57766405					0											0						No-ASE
SYCP2	20	58440630	1										1											ASE
CDH26	20	58545197											1											ASE
CDH4	20	59829904		0		0		0			0				0				1				1	Heterogeneous_ASE
GTPBP5	20	60768573				0			0			0												No-ASE
ADRM1	20	60878683				0																		No-ASE
LAMA5	20	60884427	0	0		0	0					0	0		0	0	0	0	0	0	0		1	No-ASE
CABLES2	20	60966318			0	0										0		0						No-ASE
RBBP8NL	20	60985999	0	0									0	0	0		0	0	0		0			No-ASE
C20orf166	20	61162228					0																	No-ASE
SLCO4A1	20	61287879		0	0		0		0		0				0		0	0			0			No-ASE
NTSR1	20	61340732		0		0		0			1			1			0			0	0			No-ASE
OGFR	20	61438896	0	0					0															No-ASE
COL9A3	20	61449904	0													0		0					1	No-ASE
TCFL5	20	61477034						0																No-ASE
DIDO1	20	61511775	0	0	0	0	0	0			0	0	0	0	0	0	0		0	0				No-ASE
SLC17A9	20	61588202			0	1			0		0			0	0	0			0					No-ASE
NKAIN4	20	61872136					0											0						No-ASE
COL20A1	20	61929348												0										No-ASE
CHRNA4	20	61981048					0											0						No-ASE
KCNQ2	20	62038277																0						No-ASE
EEF1A2	20	62119777													0									No-ASE
SRMS	20	62172179	0	0	0			0											0					No-ASE
HELZ2	20	62190628	0		0	0	0	0				0	0	0	0	0	0	0		0			1	No-ASE
RTEL1	20	62292793					0	0			0		0	0	0	0	0	0		0	0		1	No-ASE
ZGPAT	20	62339942	0				0						0		0	0				0				No-ASE
LIME1	20	62369392	0										0		0	0					0			No-ASE
SLC2A4RG	20	62372798	0					0			0		0	0							0		0	No-ASE
ZBTB46	20	62378335					0				0	0	0	0	0		0			0				No-ASE
ZNF512B	20	62591279	0				0						0	0	0		1	0						No-ASE
SAMD10	20	62607130						0																No-ASE
MYT1	20	62836912																	0					No-ASE
LIPI	21	15481365					0				1			1	0						1			Heterogeneous_ASE
RBM11	21	15591968				0	0								0	0					0			No-ASE
NRIP1	21	16337102				0	0		0		0	0												No-ASE
C21orf91	21	19165833		0	0	0	0		0			0	0	0						0	0	0	0	No-ASE
TMPRSS15	21	19642342	1																					ASE
MRPL39	21	26958045							0				0		0						0			No-ASE
ADAMTS1	21	28209989	1				0																	Heterogeneous_ASE
ADAMTS5	21	28296484													0									No-ASE
N6AMT1	21	30250573	0																					No-ASE
LTN1	21	30308902		0	0		0		0		0	0		0	0	0				0	0		0	No-ASE
USP16	21	30408670							0		0	0			0	0								No-ASE
CCT8	21	30433636		0																				No-ASE
GRIK1	21	30909640				0																		No-ASE
CLDN8	21	31587597					0																	No-ASE
KRTAP24-1	21	31654504					0																	No-ASE
KRTAP26-1	21	31691792					0																	No-ASE
KRTAP13-4	21	31802603	1	1		1																	1	ASE
KRTAP7-1	21	32201835			1		0		0			1												Heterogeneous_ASE
KRTAP11-1	21	32253365					0																	No-ASE
TIAM1	21	32492757					0					1	1		1									ASE
URB1	21	33689199							0			0											0	No-ASE
TCP10L	21	33949136					0													0				No-ASE
C21orf59	21	33976523									0													No-ASE
SYNJ1	21	34003354			0		0		0		0	0	0		0					0	0			No-ASE
IFNAR2	21	34614250	0	0			0							0	0	0					0			No-ASE
IL10RB	21	34640722		0		0	0				0										0		0	No-ASE
IFNGR2	21	34787294		0							0		0		0									No-ASE
GART	21	34876833				0								0										No-ASE
SON	21	34921869	0	0		0					0	0		0							0			No-ASE
CRYZL1	21	34963513												0										No-ASE
ITSN1	21	35147404			0																			No-ASE
ATP5O	21	35275907					0					0									0			No-ASE
MRPS6	21	35467645	0						0						0					0	0			No-ASE
CBR3-AS1	21	37507547	0				0							0									0	No-ASE
DOPEY2	21	37537097					0		0						0						0		0	No-ASE
CHAF1B	21	37758516	0				0														0			No-ASE
SIM2	21	38081480	0	0		0	0		0				0	0	1					1	0			No-ASE
HLCS	21	38126554					0					0		0										No-ASE
PIGP	21	38437907	0			0					0	0			0	0					0			No-ASE
TTC3	21	38455936	0		0	0	0				0	0	0	0	0						0			No-ASE
KCNJ15	21	39671199			0									0									1	Heterogeneous_ASE
BRWD1	21	40559229	0				0				0	0	0	0							0		0	No-ASE
LCA5L	21	40777900	0				0		0		0	0		0						0	0		0	No-ASE
SH3BGR	21	40823836	0			0	0		0		0		0	0						0			0	No-ASE
B3GALT5	21	41032740	0								0				0					0	0			No-ASE
IGSF5	21	41137507	0		0	0	0		0		0			0	0	0				1	0		0	No-ASE
FAM3B	21	42694870		1							0	1											1	ASE
MX1	21	42803977	0		0	0			0				0		0	0				0				No-ASE
TMPRSS2	21	42839661		0	0	0					0		0			0				0				No-ASE
RIPK4	21	43161014	0	0	0	0	0		0			0		0		0							0	No-ASE
PRDM15	21	43221483				0	0						0								0			No-ASE
C2CD2	21	43309411				0								0							0			No-ASE
ZBTB21	21	43411171	0		0		0				0			0	0								0	No-ASE
UMODL1	21	43496247			0	0	0					1	0										0	No-ASE
TFF2	21	43767595																			0			No-ASE
TMPRSS3	21	43792873	0		0		0														0			No-ASE
UBASH3A	21	43824076					0																	No-ASE
RSPH1	21	43892939	0	0					0				0										0	No-ASE
SLC37A1	21	43938572							0				0											No-ASE
WDR4	21	44270175			0	0	0		0		0	0	0	0		0				0	0		0	No-ASE
NDUFV3	21	44313454		0		0	0		0		0	0	0	0		0				0				No-ASE
SIK1	21	44836747			0		0																	No-ASE
RRP1B	21	45079654					0		0					0							0			No-ASE
RRP1	21	45209559	0	0								0		0							0			No-ASE
AGPAT3	21	45379653	0																					No-ASE
TRAPPC10	21	45483543	0	0																			0	No-ASE

PWP2	21	45528919	0	0																			0	No-ASE
C21orf33	21	45553596	0	0																			0	No-ASE
ICOSLG	21	45647421				0	0					0		0	0					0	0			No-ASE
DNMT3L	21	45668924	0	0										0	0									No-ASE
PFKL	21	45732896					0																	No-ASE
C21orf2	21	45750112										0												No-ASE
TRPM2	21	45773590		1			0					0												Heterogeneous_ASE
TSPEAR	21	45919682	0	0	0		0				0	1												No-ASE
ITGB2	21	46306709		0										1										Heterogeneous_ASE
COL18A1	21	46875500	0	0		1					1		0		0	0							0	No-ASE
SLC19A1	21	46935756		0		0	0		0			0				0					0			No-ASE
COL6A1	21	47402620					0					0		0						1	0		1	Heterogeneous_ASE
COL6A2	21	47531965	0	1		1							1	0		0					0		1	Heterogeneous_ASE
FTCD	21	47556899					0					0												No-ASE
SPATC1L	21	47581411		0													1						0	Heterogeneous_ASE
LSS	21	47611799	0	0	0	0					0	0	0	0						0	0		0	No-ASE
MCM3AP-AS1	21	47655294	0			0								0										No-ASE
YBEY	21	47706997	0	0		0								0										No-ASE
C21orf58	21	47722489				0			0		0	0	0	0							0		0	No-ASE
PCNT	21	47746375	0	0		0			0		0	0	0	0		0				0	0		0	No-ASE
PRMT2	21	48063447	0	0		0	0		0				0	0										No-ASE
XKR3	22	17264565															1	0						Heterogeneous_ASE
IL17RA	22	17577965			0							0												No-ASE
CECR5	22	17618937						0				0								0			0	No-ASE
CECR1	22	17662444												0										No-ASE
CECR2	22	17976462				0					0							0						No-ASE
BCL2L13	22	18166021										0												No-ASE
MICAL3	22	18273560			0			0			0			0				0						No-ASE
PEX26	22	18562701			0																			No-ASE
PRODH	22	18900755		1	0	0												0		0			0	No-ASE
DGCR2	22	19026403		0				0			0	0		0						0			0	No-ASE
DGCR14	22	19118992			0	0								0						0			0	No-ASE
CLTCL1	22	19168288		1	0	0		0				0		0				0					0	No-ASE
MRPL40	22	19420090		0	0	0		0				0						0						No-ASE
UFD1L	22	19445594				0																		No-ASE
CLDN5	22	19511229																0						No-ASE
TBX1	22	19747082						0				0												No-ASE
GNB1L	22	19776240			0			0				0								0			0	No-ASE
TXNRD2	22	19864669			0	0					0	0						0						No-ASE
COMT	22	19950143		0		0						0						0		0			0	No-ASE
ARVCF	22	19958768			0	0		0				0						0						No-ASE
DGCR8	22	20073518				0		0																No-ASE
RTN4R	22	20229332				0																		No-ASE
SCARF2	22	20780296		0																0				No-ASE
PI4KA	22	21073040		0										0										No-ASE
SNAP29	22	21213449												0										No-ASE
CRKL	22	21288083		0	0	0					0	0						0		0			0	No-ASE
AIFM3	22	21327589			0																			No-ASE
LZTR1	22	21342308										0												No-ASE
THAP7	22	21354503			0							0												No-ASE
P2RX6	22	21369606		1																				ASE
SLC7A4	22	21383420																		0				No-ASE
YDJC	22	21982892			0				0					0				0					0	No-ASE
CCDC116	22	21988330				0		0			0			0	0	0		0		0				No-ASE
PPM1F	22	22277485									0													No-ASE
TOP3B	22	22312879									0													No-ASE
RTDR1	22	23401668									0													No-ASE
RAB36	22	23487575									0			0	0					0			1	No-ASE
BCR	22	23523285																0						No-ASE
GUSBP11	22	24034288			0	0								0	0			0		0				No-ASE
C22orf15	22	24106336						0	0															No-ASE
DERL3	22	24177094				0					0							0						No-ASE
SLC2A11	22	24199704						0	0			0			0			0		0				No-ASE
LOC284889	22	24237074									0													No-ASE
CABIN1	22	24432578			0													0		0				No-ASE
GGT5	22	24616059										0		0										No-ASE
SPECC1L	22	24709420						0																No-ASE
GGT1	22	24982005																0						No-ASE
SGSM1	22	25243618																0						No-ASE
CRYBB3	22	25597371			0	0		0	0		0	0												No-ASE
MYO18B	22	26157068				1			1					0										Heterogeneous_ASE
ASPHD2	22	26829679										0												No-ASE
HPS4	22	26849285		1	0				0			0								0			0	No-ASE
TTC28-AS1	22	28378351			0							0						0						No-ASE
XBP1	22	29196494				0					0	0						0						No-ASE
ZNRF3	22	29383125				0					0	0		0				0					0	No-ASE
C22orf31	22	29454819			0							0												No-ASE
KREMEN1	22	29490381																					0	No-ASE
EMID1	22	29611019						0																No-ASE
RHBDD3	22	29656431									0													No-ASE
GAS2L1	22	29704361				0					0													No-ASE
THOC5	22	29904509				0		0	0		0	0								0			0	No-ASE
NIPSNAP1	22	29956807				0		0				0								0				No-ASE
UQCR10	22	30163410										0								0				No-ASE
ASCC2	22	30189592						0				0						0						No-ASE
MTMR3	22	30375036						0				0												No-ASE
TBC1D10A	22	30688488																					0	No-ASE
CCDC157	22	30762123													0									No-ASE
RNF215	22	30775729						0			0												0	No-ASE
SEC14L2	22	30793137				0					0	0		0	0					0			0	No-ASE
SEC14L3	22	30856091			1	1								1				0		1			1	ASE
SEC14L4	22	30886156				1												0						Heterogeneous_ASE
GAL3ST1	22	30951208						0	0		0			0	0								0	No-ASE
PES1	22	30975745												0										No-ASE
TCN2	22	31006860				0		0			0	1		0				0						No-ASE
MORC2	22	31328662				0						0			0			0						No-ASE
SMTN	22	31479234				0					0	0		0									0	No-ASE
PLA2G3	22	31531788				0		0	0		0	0		0	0			0					0	No-ASE
LIMK2	22	31621792			0	0		0																No-ASE
PIK3IP1	22	31679110			0	0			0			0			0			0		0			0	No-ASE
SFI1	22	31924729			0	0		0	0		0	0			0			0		0			0	No-ASE
PRR14L	22	32100678									0	0												No-ASE
DEPDC5	22	32161029									0	0												No-ASE
C22orf24	22	32330271												0										No-ASE
SLC5A1	22	32439303			0	0					0													No-ASE
RFPL2	22	32587251				0																		No-ASE
SLC5A4	22	32614536			1									0										Heterogeneous_ASE
BPIFC	22	32810378		1		0												0		0				No-ASE
FBXO7	22	32871356						0			0	0		0	0			0		0			0	No-ASE
ISX	22	35463162			0	0						0		0				0					1	No-ASE
HMGXB4	22	35658407				0		0						0						0			0	No-ASE
MCM5	22	35796514																		0				No-ASE
APOL5	22	36116662												0										No-ASE
APOL3	22	36537327			0	0												0		0				No-ASE
APOL4	22	36587154				1					1				0			0		1				Heterogeneous_ASE
APOL2	22	36623628																0		0				No-ASE
APOL1	22	36651030			0	0												0					1	No-ASE
MYH9	22	36678719									0			0						0				No-ASE

FOXRED2	22	36886060						0			0	0											0	No-ASE
CSF2RB	22	37318309												0										No-ASE
TEX33	22	37387257												1										ASE
TST	22	37407109													0									No-ASE
TMPRSS6	22	37462155				0					0	0		0				0						No-ASE
IL2RB	22	37524140													1								1	ASE
C1QTNF6	22	37578337				0		0							0			0		0				No-ASE
SSTR3	22	37602602																0						No-ASE
CARD10	22	37887241			0	0						0		0						0				No-ASE
SH3BP1	22	38038616				0						0		0						0				No-ASE
PDXP	22	38061576				0																		No-ASE
TRIOBP	22	38106521		0		0						0											0	No-ASE
GCAT	22	38205989			0	0									0			0		0			0	No-ASE
EIF3L	22	38259334				0																	0	No-ASE
MICALL1	22	38308394			0										0			0						No-ASE
SLC16A8	22	38474406				0		0							0			0		0				No-ASE
BAIAP2L2	22	38481311													0									No-ASE
KDELR3	22	38864287						0						0						0				No-ASE
FAM227A	22	38993302				0																		No-ASE
SUN2	22	39132369						0												0				No-ASE
DNAL4	22	39176976									0													No-ASE
APOBEC3F	22	39440176			0	0					0			0				0					0	No-ASE
APOBEC3H	22	39493294																0						No-ASE
SYNGR1	22	39760219			1	1						0											0	Heterogeneous_ASE
MGAT3	22	39883517			0	0																		No-ASE
FAM83F	22	40415226			0	0		0												0				No-ASE
TNRC6B	22	40552119				0								0										No-ASE
MKL1	22	40813413			0															0				No-ASE
EP300	22	41513412			0			0																No-ASE
RANGAP1	22	41647049																0						No-ASE
ZC3H7B	22	41721792						0						0										No-ASE
C22orf46	22	42089623						0			0													No-ASE
CENPM	22	42336172									0			0	0			0						No-ASE
WBP2NL	22	42394835																0						No-ASE
FAM109B	22	42473586										0												No-ASE
NDUFA6	22	42482252									0	0												No-ASE
TCF20	22	42557363									0	0		0				0						No-ASE
RRP7A	22	42908938		0		0						0			0		1			0				No-ASE
A4GALT	22	43089044			0									0										No-ASE
ARFGAP3	22	43195045			0	0		0				0								0			0	No-ASE
MCAT	22	43529061			0	0																		No-ASE
TSPO	22	43557156			0										0			0		0			0	No-ASE
TTLL12	22	43564050			0							0			0					0			0	No-ASE
EFCAB6	22	43933285		0		0						0		0				0						No-ASE
PNPLA5	22	44277512																0						No-ASE
PNPLA3	22	44319936			0			0			0							0		0			0	No-ASE
SAMM50	22	44359215			0			0			0			0	0			0		0			0	No-ASE
PARVB	22	44395434			1			1				0		0	0			0		0			0	No-ASE
PRR5	22	45075720				0														0			0	No-ASE
PRR5-ARHGAP8	22	45182361				0		0			0			0	0					0				No-ASE
UPK3A	22	45683071						0				0		0				0					1	No-ASE
FAM118A	22	45719183			0			0				0								0			0	No-ASE
SMC1B	22	45740462				1		0																Heterogeneous_ASE
RIBC2	22	45810273		0	1	0		0															1	Heterogeneous_ASE
FBLN1	22	45914595				0																		No-ASE
WNT7B	22	46318879										0												No-ASE
C22orf26	22	46447714									0							0						No-ASE
CDPF1	22	46640993											0										0	No-ASE
PKDREJ	22	46652516						0															0	No-ASE
TTC38	22	46664412												0										No-ASE
TRMU	22	46731689									0													No-ASE
CELSR1	22	46759949				0					0	0		0	1			0		0			1	No-ASE
CERK	22	47085983						1																ASE
ZBED4	22	50277333						0				0		0						0			0	No-ASE
ALG12	22	50297526										0								0			0	No-ASE
CRELD2	22	50313441						0				0		0						0			0	No-ASE
PIM3	22	50354783				0					0							0		0				No-ASE
IL17REL	22	50435480				1		0				0						0		0			1	Heterogeneous_ASE
MLC1	22	50502491																0						No-ASE
MOV10L1	22	50528569												0										No-ASE
SELO	22	50644783									0													No-ASE
TUBGCP6	22	50656236			0			0			0			0	0								0	No-ASE
HDAC10	22	50684515										0												No-ASE
PLXNB2	22	50713409			0			0			0			0	0			0		0			0	No-ASE
DENND6B	22	50750573			0			0						0	0			0					0	No-ASE
PPP6R2	22	50845173															1							ASE
LMF2	22	50941924			0			0										0		0			0	No-ASE
NCAPH2	22	50956201			0							0		0						0				No-ASE
SCO2	22	50964207										0		0										No-ASE
ODF3B	22	50969172						0			0													No-ASE
CPT1B	22	51007772			0							0		0						0				No-ASE
MAPK8IP2	22	51041663										0						0						No-ASE
ARSA	22	51063758			0						0	0		0				0					0	No-ASE
SHANK3	22	51117580			0									0	0									No-ASE
GTPBP6	X	221792																	0					No-ASE
CRLF2	X	1314931									0													No-ASE
CSF2RA	X	1404785																			0			No-ASE
ASMTL	X	1536976																					0	No-ASE
DHRSX	X	2139168						0			0			0		0			0				0	No-ASE
CD99P1	X	2575151						0								0			0					No-ASE
XG	X	2688612						0																No-ASE
GYG2	X	2772079									0								0					No-ASE
ARSD	X	2825403														0			0	1			1	Heterogeneous_ASE
MXRA5	X	3227866																	1					ASE
PRKX	X	3530357									0			0										No-ASE
HDHD1	X	6975752						0			0			0		0			0					No-ASE
VCX2	X	8138080																	0					No-ASE
GPR143	X	9693808					0													1				Heterogeneous_ASE
SHROOM2	X	9841734												1		1			1					ASE
WWC3	X	10035443																	1					ASE
MID1	X	10417412						1																ASE
HCCS	X	11130185														1								ASE
ARHGAP6	X	11157154															1							ASE
TCEANC	X	13680941						0											0		0			No-ASE
FANCB	X	14861792						1								1								ASE
PIGA	X	15339663						1																ASE
PIR-FIGF	X	15364358																	1					ASE
TXLNG	X	16804627																	0					No-ASE
RBBP7	X	16881125									0			1										Heterogeneous_ASE
NHS	X	17653697																	0					No-ASE
GPR64	X	19014186																	1					ASE
SH3KBP1	X	19555842												0										No-ASE
MAP7D2	X	20030588						0																No-ASE
RPS6KA3	X	20173571						1																ASE
LOC100873065	X	22291205												1							1			ASE
DMD	X	31140021																			1			ASE
FAM47B	X	34960959							1															ASE
MAGEB16	X	35820321						1						0										Heterogeneous_ASE

SYTL5	X	37913566						0						0										No-ASE
RPGR	X	38128962									1								1					ASE
OTC	X	38226603																	1					ASE
TSPAN7	X	38533570																	1					ASE
BCOR	X	39911528												0										No-ASE
ATP6AP2	X	40450585														1								ASE
DUSP21	X	44703415												1										ASE
KDM6A	X	44920641									0													No-ASE
UBA1	X	47061812																	0		0			No-ASE
ZNF41	X	47306860																	1		1			ASE
ARAF	X	47425058						1								1			1					ASE
ZNF81	X	47705674																	1					ASE
ZNF630	X	47918194																	1		1			ASE
TBC1D25	X	48418084		1	0						1			1		1			1		1			ASE
PRAF2	X	48929620									0													No-ASE
CCDC22	X	49093644									1													ASE
AKAP4	X	49955614																	1					ASE
FGD1	X	54472607									1													ASE
ZXDB	X	57622607												1										ASE
AMER1	X	63410516												1					1					ASE
HEPH	X	65382685						0			0					1								Heterogeneous_ASE
EDA2R	X	65819397						0						1										Heterogeneous_ASE
OPHN1	X	67272395												1										ASE
PJA1	X	68381264									1			1							1			ASE
IGBP1	X	69353856																	1					ASE
P2RY4	X	69478417																			1			ASE
GDPD2	X	69646306														1			1					ASE
NHSL2	X	71349700												1					0					Heterogeneous_ASE
FLJ44635	X	71379689						0						1		0					1			Heterogeneous_ASE
PIN4	X	71401607						1													1			ASE
SLC16A2	X	73641569						1																ASE
ABCB7	X	74284934																	1					ASE
ATRX	X	76937107									1			1					1		1			ASE
COX7B	X	77158147																	1					ASE
ATP7A	X	77244108						1			1			1							1			ASE
TAF9B	X	77387192												1										ASE
HDX	X	83591879												1										ASE
SATL1	X	84349207												0					1					Heterogeneous_ASE
POF1B	X	84560861						0													0			No-ASE
TGIF2LX	X	89177673												1										ASE
DIAPH2	X	95993666					1				1													ASE
TSPAN6	X	99885830									1					1								ASE
SYTL4	X	99931059						1											1					ASE
DRP2	X	100486731									1													ASE
ARMCX4	X	100753863									1			1										ASE
RAB40A	X	102755551									1			1										ASE
SERPINA7	X	105277497																	1					ASE
MORC4	X	106185196									1					1			1		1			ASE
NUP62CL	X	106396402									1								1		1			ASE
MID2	X	107084385																	1					ASE
COL4A6	X	107400354																	1					ASE
GUCY2F	X	108619395												1										ASE
ZCCHC16	X	111698032												1										ASE
DOCK11	X	117676910									1													ASE
SLC25A43	X	118540475						1						1										ASE
C1GALT1C1	X	119760356																			1			ASE
XIAP	X	123020195												1		1								ASE
TENM1	X	123514429																	1					ASE
BCORL1	X	129139268									1			1		1					1			ASE
OR13H1	X	130678124									1													ASE
HS6ST2	X	131762541									1													ASE
TFDP3	X	132351204											1										1	ASE
GPC4	X	132437337									1			1		1					1			ASE
FAM122C	X	133941729												1										ASE
FHL1	X	135289301									1													ASE
MAP7D3	X	135301798						1			1			1		1					1			ASE
GPR101	X	136112335									1													ASE
MAGEC3	X	140967121						0																No-ASE
MAGEC1	X	140993216						1								1	1							ASE
FMR1	X	147010263									1								1					ASE
IDS	X	148564440																	1					ASE
MAGEA8	X	149013071																	1					ASE
MAMLD1	X	149613805																			1			ASE
MTMR1	X	149896168																	1					ASE
GPR50	X	150345354														1								ASE
GABRE	X	151123258						1						1		1								ASE
CSAG1	X	151909156						1																ASE
ZNF185	X	152083059												1		1								ASE
PLXNB3	X	153030991			1			0						0		1								Heterogeneous_ASE
RENBP	X	153201025																			0			No-ASE
HCFC1	X	153216900																	1		0			Heterogeneous_ASE
TMEM187	X	153247544																			0			No-ASE
FLNA	X	153577234																	1					ASE
DNASE1L1	X	153631119						1																ASE
PLXNA3	X	153688579																			1			ASE
SPRY3	X	155009856					0												1	1			0	Heterogeneous_ASE
TTTY14	Y	21154466					1																1	ASE

Supplementary table 2: Stability of *DLX5* ASE during tumorigenesis

	Normal mucosa	Colorectal cancer	ASE during tumorigenesis
ET14018	Mono1	pMA2	**Allelic switching**
ET14057	Mono1	pMA1	Stable ASE
ET14008	pMA1	Mono2	**Allelic switching**
ET14086	pMA1	BiA	Loss of ASE
ET14014	pMA1	BiA	Loss of ASE
ET14041	Unb1	BiA	Loss of ASE
ET14063	Unb1	Unb1	Stable ASE
ET14074	BiA	Mono2	ASE acquired
ET14059	BiA	pMA2	ASE acquired
ET14088	BiA	BiA	Stable bi-allelic
ET14075	BiA	Unb1	ASE acquired
ET14038	BiA	Unb1	ASE acquired
ET14025	BiA	Unb1	ASE acquired
ET14028	BiA	pMA1	ASE acquired
ET14031	BiA	pMA1	ASE acquired
ET14054	BiA	pMA1	ASE acquired
ET14001	Unb2	pMA2	Stable ASE
ET14067	Unb2	BiA	Loss of ASE
ET14042	Unb2	BiA	Loss of ASE
ET14087	pMA2	Mono2	Stable ASE
ET14090	pMA2	BiA	Loss of ASE
ET14020	pMA2	BiA	Loss of ASE
ET14009	Mono2	Mono2	Stable ASE
ET14071	Mono2	Mono2	Stable ASE
ET14081	Mono2	Mono2	Stable ASE
ET14027	Mono2	Mono2	Stable ASE
ET14043	Mono2	Mono2	Stable ASE
ET14052	Mono2	Unb2	Stable ASE
ET14015	Mono2	BiA	Loss of ASE
ET14089	Mono2	BiA	Loss of ASE
ET14029	Mono2	Mono1	**Allelic switching**

ASE call legend:

Mono1	Mono-allelic expression of 8-G-allele
pMA1	Predominantly mono-allelic expression of 8-G-allele
Unb1	Unbalanced *DLX5* expression, mostly of 8-G-allele
BiA	Bi-allelic *DLX5* expression
Unb2	Unbalanced *DLX5* expression, mostly of 7-G-allele
pMA2	Predominantly mono-allelic expression of 7-G-allele
Mono2	Mono-allelic expression of 7-G-allele

Supplementary table 3: PRPS1L1 KASPar genotyping results in paired normal and cancer samples.

Sample	T-DNA	N-DNA	T-cDNA	N-cDNA
**ET14001**	Allele 2	Allele 2	Allele 1	Allele 1
**ET14002**	Allele 1	Allele 1	Allele 1	Allele 1
**ET14003**	Heterozygote	Heterozygote	Allele 1	Heterozygote
**ET14004**	Allele 2	Allele 2	Heterozygote	Allele 1
**ET14005**	Heterozygote	Heterozygote	Allele 1	Allele 1
**ET14006**	Heterozygote	Heterozygote	Heterozygote	Allele 1
**ET14007**	Heterozygote	Heterozygote	Allele 1	Allele 1
**ET14008**	Heterozygote	Heterozygote	Heterozygote	Allele 1
**ET14009**	Allele 1	Allele 1	Allele 1	Allele 1
**ET14010**	Allele 1	Allele 1	Allele 1	Allele 1
**ET14011**	Allele 1	Allele 2	Allele 1	Heterozygote
**ET14012**	Heterozygote	Heterozygote	Heterozygote	Allele 1
**ET14013**	Allele 1	Allele 1	Allele 1	Allele 1
**ET14014**	Allele 2	Allele 2	Heterozygote	Allele 1
**ET14015**	Heterozygote	Heterozygote	Allele 1	Allele 1
**ET14016**	Allele 1	Allele 1	Allele 1	Allele 1
**ET14017**	Heterozygote	Heterozygote	Allele 1	Allele 1
**ET14018**	Allele 1	Allele 1	Allele 1	Allele 1
**ET14019**	Heterozygote	Heterozygote	Heterozygote	Allele 1
**ET14020**	Allele 1	Allele 1	Allele 1	Allele 1
**ET14021**	Heterozygote	Heterozygote	Heterozygote	Allele 1
**ET14023**	Heterozygote	Heterozygote	Allele 1	Allele 1
**ET14024**	Allele 2	Allele 2	Heterozygote	Heterozygote
**ET14025**	Allele 1	Allele 1	Allele 1	Allele 1
**ET14026**	Heterozygote	Heterozygote	Heterozygote	Allele 1
**ET14027**	Heterozygote	Heterozygote	Allele 1	Allele 1
**ET14028**	Heterozygote	Heterozygote	Heterozygote	Heterozygote
**ET14029**	Heterozygote	Heterozygote	Allele 1	Allele 1
**ET14030**	Allele 2	Allele 2	Heterozygote	Allele 1
**ET14031**	Heterozygote	Heterozygote	Heterozygote	Allele 1
**ET14033**	Allele 2	Allele 2	Allele 1	Allele 1
**ET14034**	Heterozygote	Heterozygote	Allele 1	Allele 1
**ET14035**	Allele 1	Allele 1	Allele 1	Allele 1
**ET14036**	Allele 1	Allele 1	Allele 1	Allele 1
**ET14037**	Heterozygote	Heterozygote	Allele 1	Heterozygote
**ET14038**	Allele 1	Allele 1	Allele 1	Heterozygote
**ET14039**	Allele 1	Allele 1	Allele 1	Allele 1
**ET14040**	Heterozygote	Heterozygote	Allele 1	Heterozygote
**ET14041**	Heterozygote	Heterozygote	Heterozygote	Allele 1

**ET14042**	Allele 1	Allele 1	Allele 1	Allele 1
**ET14043**	Allele 1	Allele 1	Allele 1	Allele 1
**ET14044**	Allele 2	Allele 2	Heterozygote	Allele 1
**ET14045**	Heterozygote	Heterozygote	Allele 1	Allele 1
**ET14046**	Heterozygote	Heterozygote	Heterozygote	Heterozygote
**ET14047**	Heterozygote	Heterozygote	Heterozygote	Heterozygote
**ET14048**	Heterozygote	Heterozygote	Allele 1	Allele 1
**ET14049**	Allele 2	Allele 2	Heterozygote	Allele 1
**ET14050**	Heterozygote	Heterozygote	Allele 1	Allele 1
**ET14051**	Heterozygote	Heterozygote	Allele 1	Allele 1
**ET14052**	Allele 1	Allele 1	Allele 1	Allele 1
**ET14053**	Heterozygote	Heterozygote	Heterozygote	Allele 1
**ET14054**	Allele 1	Allele 1	Allele 1	Allele 1
**ET14055**	Heterozygote	Heterozygote	Heterozygote	Allele 1
**ET14056**	Heterozygote	Heterozygote	Allele 1	Allele 1
**ET14057**	Allele 1	Allele 1	Allele 1	Allele 1
**ET14058**	Allele 1	Allele 1	Allele 1	Allele 1
**ET14059**	Heterozygote	Heterozygote	Heterozygote	Heterozygote
**ET14060**	Heterozygote	Heterozygote	Allele 1	Allele 1
**ET14061**	Allele 1	Allele 1	Allele 1	Allele 1
**ET14062**	Heterozygote	Heterozygote	Allele 1	Allele 1
**ET14063**	Allele 2	Allele 2	Undetermined	Heterozygote
**ET14064**	Allele 2	Allele 2	Allele 1	Allele 1
**ET14065**	Heterozygote	Heterozygote	Allele 1	Allele 1
**ET14066**	Allele 1	Allele 1	Allele 1	Heterozygote
**ET14067**	Heterozygote	Heterozygote	Allele 1	Heterozygote
**ET14068**	Heterozygote	Heterozygote	Allele 1	Allele 1
**ET14069**	Allele 1	Heterozygote	Heterozygote	Heterozygote
**ET14070**	Allele 1	Allele 1	Allele 1	Allele 1
**ET14071**	Heterozygote	Heterozygote	Heterozygote	Allele 1
**ET14072**	Allele 2	Allele 2	Heterozygote	Allele 1
**ET14073**	Allele 2	Allele 2	Heterozygote	Allele 1
**ET14074**	Heterozygote	Heterozygote	Allele 1	Allele 1
**ET14075**	Allele 1	Allele 1	Allele 1	Allele 1
**ET14076**	Heterozygote	Heterozygote	Heterozygote	Allele 1
**ET14077**	Heterozygote	Heterozygote	Allele 1	Allele 1
**ET14078**	Allele 1	Allele 1	Allele 1	Allele 1
**ET14079**	Allele 1	Allele 1	Allele 1	Heterozygote
**ET14080**	Allele 1	Allele 1	Allele 1	Allele 1
**ET14081**	Heterozygote	Heterozygote	Allele 1	Allele 1
**ET14082**	Heterozygote	Heterozygote	Allele 1	Heterozygote
**ET14083**	Allele 2	Allele 2	Allele 1	Allele 1
**ET14084**	Allele 1	Allele 1	Allele 1	Allele 1
**ET14085**	Allele 1	Allele 1	Allele 1	Allele 1

**ET14086**	Allele 2	Allele 2	Heterozygote	Heterozygote
**ET14087**	Heterozygote	Heterozygote	Heterozygote	Allele 1
**ET14088**	Heterozygote	Heterozygote	Allele 1	Heterozygote
**ET14089**	Allele 1	Allele 1	Allele 1	Allele 1
**ET14090**	Heterozygote	Heterozygote	Heterozygote	Heterozygote
**ET14091**	Allele 2	Allele 2	Heterozygote	Heterozygote

Supplementary table 4: Samples used in this study.

```
Sample,Sample_type,Gender
JVE015,Cell line,F
JVE017,Cell line,F
JVE044,Cell line,F
JVE059,Cell line,M
JVE109,Cell line,F
JVE127,Cell line,M
JVE192,Cell line,F
JVE207,Cell line,M
JVE222,Cell line,F
JVE241,Cell line,M
JVE367,Cell line,F
JVE528,Cell line,F
JVE774,Cell line,M
KP363T,Cell line,M
KP7038T,Cell line,M
JVE103,Cell line,M
JVE114,Cell line,M
JVE187,Cell line,F
JVE253,Cell line,F
JVE371,Cell line,M
KP283T,Cell line,F
ET14001,Primary tissue,M
ET14002,Primary tissue,M
ET14003,Primary tissue,M
ET14004,Primary tissue,M
ET14005,Primary tissue,M
ET14006,Primary tissue,M
ET14007,Primary tissue,F
ET14008,Primary tissue,M
ET14009,Primary tissue,F
ET14010,Primary tissue,M
ET14011,Primary tissue,F
ET14012,Primary tissue,M
ET14013,Primary tissue,F
ET14014,Primary tissue,F
ET14015,Primary tissue,F
ET14016,Primary tissue,M
ET14017,Primary tissue,M
ET14018,Primary tissue,F
ET14019,Primary tissue,M
ET14020,Primary tissue,M
ET14021,Primary tissue,M
ET14023,Primary tissue,M
ET14024,Primary tissue,M
ET14025,Primary tissue,F
ET14026,Primary tissue,F
ET14027,Primary tissue,F
ET14028,Primary tissue,F
ET14029,Primary tissue,F
ET14030,Primary tissue,M
ET14031,Primary tissue,F
ET14033,Primary tissue,F
```

ET14034,Primary tissue,F
ET14035,Primary tissue,F
ET14036,Primary tissue,F
ET14037,Primary tissue,F
ET14038,Primary tissue,M
ET14039,Primary tissue,M
ET14040,Primary tissue,F
ET14041,Primary tissue,F
ET14042,Primary tissue,M
ET14043,Primary tissue,M
ET14044,Primary tissue,F
ET14045,Primary tissue,M
ET14046,Primary tissue,F
ET14047,Primary tissue,F
ET14048,Primary tissue,M
ET14049,Primary tissue,F
ET14050,Primary tissue,M
ET14051,Primary tissue,M
ET14052,Primary tissue,M
ET14053,Primary tissue,M
ET14054,Primary tissue,M
ET14055,Primary tissue,M
ET14056,Primary tissue,M
ET14057,Primary tissue,F
ET14058,Primary tissue,F
ET14059,Primary tissue,F
ET14060,Primary tissue,M
ET14061,Primary tissue,F
ET14062,Primary tissue,F
ET14063,Primary tissue,M
ET14064,Primary tissue,F
ET14065,Primary tissue,M
ET14066,Primary tissue,M
ET14067,Primary tissue,M
ET14068,Primary tissue,F
ET14069,Primary tissue,F
ET14070,Primary tissue,F
ET14071,Primary tissue,M
ET14072,Primary tissue,M
ET14073,Primary tissue,F
ET14074,Primary tissue,F
ET14075,Primary tissue,M
ET14076,Primary tissue,M
ET14077,Primary tissue,F
ET14078,Primary tissue,M
ET14079,Primary tissue,F
ET14080,Primary tissue,M
ET14081,Primary tissue,F
ET14082,Primary tissue,F
ET14083,Primary tissue,F
ET14084,Primary tissue,M
ET14085,Primary tissue,M
ET14086,Primary tissue,M
ET14087,Primary tissue,M
ET14088,Primary tissue,F
ET14089,Primary tissue,M
ET14090,Primary tissue,M
ET14091,Primary tissue,F
